# Dichotomy of platinum(II) and gold(III) carbene intermediates switching from *N*- to *O*-selectivity

**DOI:** 10.1038/s41467-022-29326-0

**Published:** 2022-03-30

**Authors:** Hongming Jin, Wen-Yan Tong, Jing Zhang, Matthias Rudolph, Frank Rominger, Xu Shen, Shuanglin Qu, A. Stephen K. Hashmi

**Affiliations:** 1 School of Pharmacy, Experiment Center for Science and Technology, Nanjing University of Chinese Medicine, Nanjing, China; 2 Organisch-Chemisches Institut, Im Neuenheimer Feld 270, Universität Heidelberg, Heidelberg, Germany; 3 College of Chemistry and Chemical Engineering, Hunan University, Changsha, China; 4 Jiangsu Key Laboratory for Pharmacology and Safety Evaluation of Chinese Materia Medica, State Key Laboratory Cultivation Base for TCM Quality and Efficacy, Nanjing University of Chinese Medicine, Nanjing, China

**Keywords:** Homogeneous catalysis, Reaction mechanisms

## Abstract

Pt(II) and Au(III)-mediated intermolecular divergent annulations of benzofurazans and ynamides highlighted the *N*- to *O*-selectivity of tunable metal carbene intermediates. PtCl_2_ with a bulky phosphite ligand resulted in the specific synthesis of six-membered quinoxaline *N*-oxides and successfully suppressed the in-situ deoxygenation of *N*-oxides. On the other hand, an unique gold(III) catalyst (2,6-di-MeO-PyrAuCl_3_) led to the five-membered ring products, benzimidazoles. A broad scope of functional groups was well compatible, delivering better yields and selectivities in contrast to conventional gold(I) catalysts. The different behavior of presumed platinum(II) and gold(III) carbenes with respect to chemoselectivity was intensively examined by experiments and DFT calculations. A detailed mechanistic study, based on DFT calculations, revealed that the highly electrophilic carbocation-like gold(III) carbene triggers an oxophilic cyclization, followed by a cascade ring contraction and acyl migration. On the contrary, the Pt carbene species is less cationic, favoring the formation of the six-membered ring via *N*-attack.

## Abstract (web summary)

Benzofurazan, a cyclic heterocycle, can form open-chain metal carbene species in the presence of suitable catalysts. Here the authors show divergent reactivity when using gold(III) and platinum(II) catalysts, and perform computational and experimental mechanistic studies to explain the differing reactivity

## Introduction

**Fig. 1:**
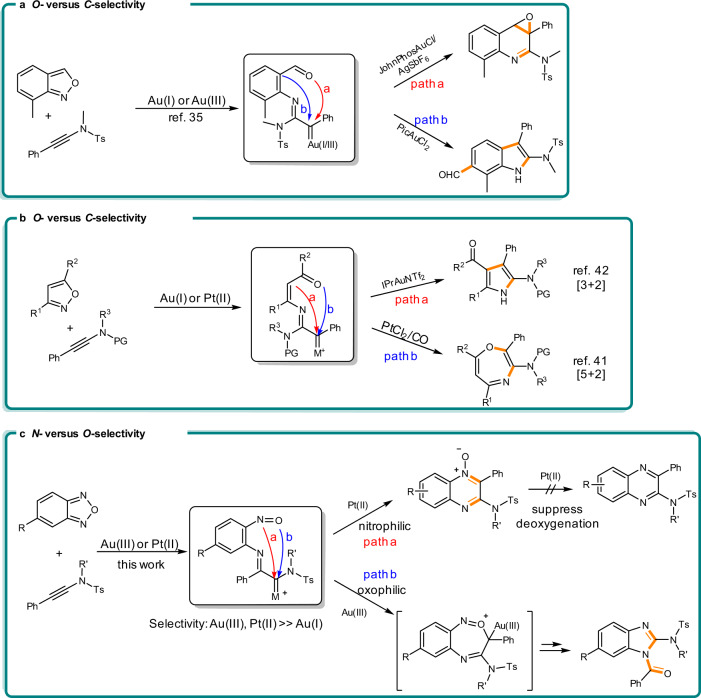
Tunable gold and platinum carbene annulations. **a** Selectivity of gold(I) and gold(III) carbene. **b** Selectivity of platinum(II) and gold(I) carbene. **c** This work: Selectivity of platinum(II) and gold(III) carbene.

Gold-catalyzed intermolecular formal cycloadditions have attracted enormous attention as robust, flexible tool for the construction of heterocyclic frameworks^1–6^. Here, gold carbenes usually serve as the key electrophilic intermediates^7–17^. Recently, the intermolecular access to α-imino gold(I) carbenes via direct nitrene transfer to alkynes has significantly expanded the synthetic possibilities of gold catalysis^18–34^. However, the study of gold(III) carbene analogues that behave and react distinctly to gold(I) carbenes has been realized seldom. Very recently, in the reaction of ynamides with 7-methylanthranil the cyclization pathway of gold carbene intermediate depends on ligands but also the oxidation state of gold^35^. While gold(I) carbenes were facilely trapped by the oxygen atom of aldehyde (Fig. 1a, path a), leading to the epoxidation product, presumed gold(III) carbenes favored to react with the aryl moiety of anthranil, delivering the indole framework after 1,4-acyl migration (Fig. 1a, path b).

Gold- and platinum catalysts only occasionally have been systematically compared in chemo-divergent reactions of the same substrates^36–40^, especially in the field of carbene intermediates. Ye’s group speculated on the involvement of α-imino platinum carbenes in the reaction of isoxazoles with ynamides^41^. Different from the carbophilic gold(I) carbene, furnishing a [3 + 2] annulation (Fig. 1b, path a)^42^, the platinum(II) carbene turned out to be more oxophilic, giving rise to a [5 + 2] cyclization pattern (Fig. 1b, path b). Given the significance with relevance to the synthetic flexibility and catalytic efficiency, the switching of chemo-selectivity by fine-tunable metal catalysts remains highly desirable.

Gold(I)-catalyzed formal [4 + 2] cycloaddition of benzofurazan with ynamides afforded a raw protocol to the synthesis of quinoxaline *N*-oxides^43^, a frequent substructure of pharmaceutical compounds. However, the inevitable gold(I)-catalyzed in situ deoxygenation of quinoxaline *N*-oxide by reacting with residual ynamide delivered a mountain of quinoxaline by-product^43,44^, decreasing the feasibility of this strategy in practice. Moreover, in comparison with the distinct nucleophilicity of carbon and oxygen atom in previous work, the nucleophilicity of nitrogen and oxygen atom is much closer. Thus, the promotion of tunable *N*- and *O*-selective annulations of nitroso with metal carbenes still remains a challenge.

**Fig. 2:**
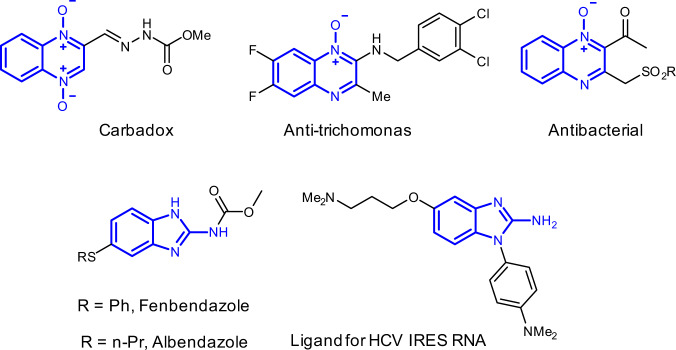
Related bioactive molecules. The pharmaceutical molecules containing quinoxaline *N*-oxide or 2-aminobenzimidazole skeleton.

In this work, Pt(II) and Au(III) catalysts are examined in order to overcome above problems (Fig. 1c). A bulky phosphite ligand cooperating with PtCl_2_ improve the catalytic activity of platinum but also successfully suppress the annoying in situ deoxygenation, giving outstanding yields of *N*-oxides. Intriguingly, taking advantage of a pyridine-based gold(III) catalyst (2,6-di-MeO-PyrAuCl_3_) result in valuable 2-aminobenzimidazole derivatives. Rather than the Pt carbene, being always intercepted by the nitrogen atom, the Au(III) carbene is trapped by the terminal oxygen atom of the nitrosyl group and then undergoes further C–C bond cleavage and rearrangement. DFT calculations examine the different free energy activation barriers for the formation of six- and seven-membered ring transition states and elucidate the divergent annulation routes of assumed Pt(II) and Au(III) carbene intermediates. Selected bioactive molecules containing the frameworks of quinoxaline *N*-oxide and 2-aminobenzimidazole are listed in Fig. 2^45–49^.

## Results and discussion

### Reaction optimization

Initially, the reaction of 2 equiv. benzofurazan **1a** and ynamide **2a** with 5 mol% Me_4_tBuXPhosAuCl/AgNTf_2_ in 1,2-DCE at 70 °C (Table 1, entry 1) provided a mixture of the products **3a** (20%), **5a** (45%) and a trace amount of **4a**. The product **5a** is convinced from the in situ deoxygenation of **4a** with residual ynamides^44^. Other gold(I) catalysts still afforded a mixture, whether we altered ligands, counter anions or temperature (entry 2–4). Simple AuCl_3_ increased the yield of **3a** as well as **4a** (entry 4). Gold(III) with bidentate ligands just led to a trace amount of **3a** (entry 5–6). A pyridine-coordinated gold(III) complex could provide higher selectivity for **3a** (entry 7–8). With the take-up of 4 equiv. **1** and Pyr-2, the gold(III) complex **6** (Pyr-2AuCl_3_) could give rise in 70% yield of **3a** (entry 9). A similar result was obtained with Pyr-3AuCl_3_ (entry 10). In contrast to gold(III) catalyst, platinum catalysis only gave quinoxaline derivatives without the benzimidazole **3**. Simple PtCl_2_ had less catalytic reactivity (entry 11). The addition of ligands led to higher total yields (entry 12–14). PtCl_2_ in combination with bulky phosphite ligands **L2** reacted selectively (entry 15). Ligand **L3** with large cone angle afforded the quinoxaline *N*-oxide **4a** even in 90% yield and with specific selectivity (entry 16). This suggested that the bulky umbrella-shaped phosphite ligand might protect the platinum carbene from the intermolecular oxidation with **4a**. Pt(0) catalyst could not promote this reaction (entry 17).

**Table 1:** Optimization of reaction conditions^a^.


Entry	Catalyst	Solvent	Yield (%)^b^		
			3a	4a	5a
1	5 mol% Me_4_tBuXPhosAuCl/AgNTf_2_	1, 2-DCE	20%	<5%	45%
2	5 mol% Me_4_tBuXPhosAuCl/AgSbF_6_	1, 2-DCE	10%	13%	30%
3	5 mol% **L1**AuCl/AgSbF_6_	1, 2-DCE	8%	<5%	40%
4^c^	5 mol% tBuXPhosAuCl/AgSbF_6_	1, 2-DCE	12%	36%	18%
4	10 mol% AuCl_3_	1, 2-DCE	38%	36%	13%
5	10 mol% PicAuCl_2_	1, 2-DCE	<5%	61%	10%
6	10 mol% AuppyCl_2_	1, 2-DCE	<5%	<5%	0%
7^d^	10 mol% PyrAuCl_3_	1, 2-DCE	43%	32%	<5%
8^d^	10 mol% Pyr-1AuCl_3_	1, 2-DCE	59%	30%	<5%
**9**^**d, f**^	**10** **mol% Pyr-2AuCl**_**3**_	**1, 2-DCE**	**70%**	**19%**	**<5%**
10^d^	10 mol% Pyr-3AuCl_3_	1, 2-DCE	67%	20%	<5%
11	10 mol% PtCl_2_	1, 2-DCE	0%	8%	0%
12	10 mol% PtCl_2_ (1 atm. CO)	Toluene	0%	37%	<5%
13^e^	10 mol% PtCl_2_/P(C_6_F_5_)_3_	Toluene	0%	21%	0%
14^e^	10 mol% PtCl_2_/**L1**	Toluene	0%	62%	<5%
15^e^	10 mol% PtCl_2_/**L2**	Toluene	0%	84%	0%
**16**^**e, f**^	**10** **mol% PtCl**_**2**_**/L3**	**Toluene**	**0%**	**90%**	**0%**
17^e^	10 mol% Pt(PPh_3_)_4_	Toluene	0%	0%	0%

*Ad* 1-adamantyl, *1, 2-DCE* 1, 2-dichloroethane, *NTf*_*2*_ bis(trifluoromethanesulfonyl)imide.

^a^**1a** (0.2 mmol, 2 equiv.) and **2a** (0.1 mmol) in 2 ml solvent at 70 °C.

^b^Isolated yield.

^c^At 25 °C.

^d^**1a** (0.4 mmol, 4 equiv.), 4 ml 1, 2-DCE.

^e^In 1 ml toluene.

^f^The best condition.

### Substrate scope for the synthesis of benzimidazole by Au(III) catalysis

**Fig. 3:**
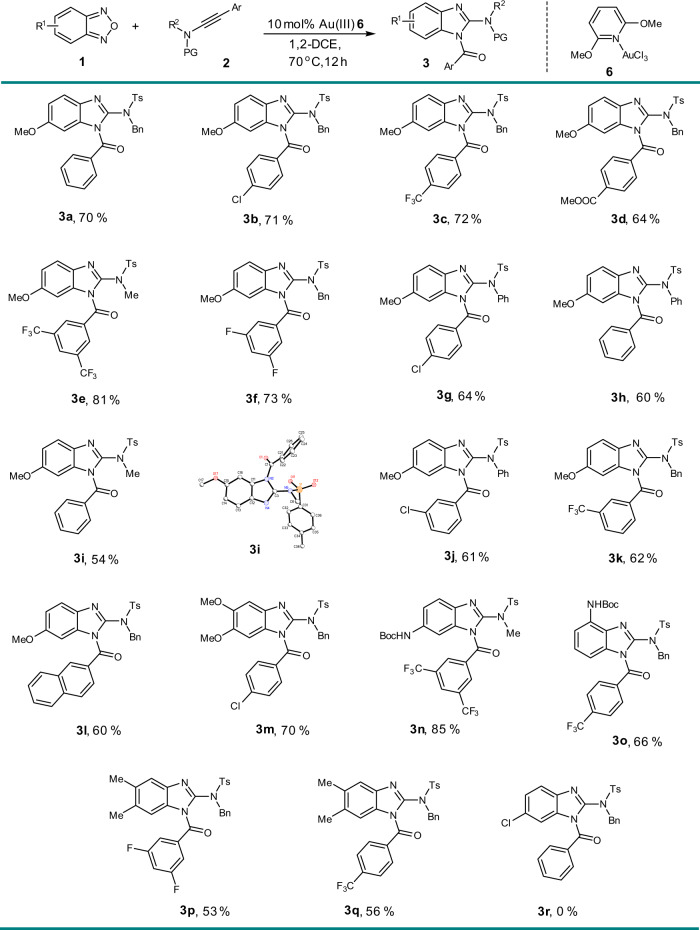
Scope with respect to the Au(III)-catalyzed tandem annulations. Reaction conditions: **1** (0.4 mmol), **2** (0.1 mmol), 10 mol% Au(III) catalyst **6** in 4 ml 1,2- dichloroethane at 70 °C for 12 h. Isolated yields were listed.

Under the optimized reaction conditions, the scope of reaction was evaluated. A range of ynamides was investigated under the gold(III) catalysis (Fig. 3). Benzyl-, methyl- and phenyl-substituted ynamides reacted smoothly. In general, ynamides bearing electron-withdrawing aryl groups gave better yields. Different functional groups, including halogen (**3b**, **3f**), trifluoromethyl (**3e**) and ester (**3d**) groups, were tolerated. The scope of benzofurazans was also checked. Apart from methoxy group, benzofurazans bearing other electron-donating substituents, such as an amide (**3n**) and a methyl group (**3p**) were also suitable, delivering moderate to good yields. Besides of β-substitution, the α-substituted benzofurazan also performed well (**3o**). The solid state molecular structure of **3i** was confirmed by single-crystal X-ray diffraction. Benzofurazan with electron-withdrawing group could not afford the benzimidazole (**3r**), which may owe to the low nucleophilicity of oxygen atom on nitroso induced by negative inductive effect. Alkyl-substituted ynamides easily decomposed to α,β-unsaturated amides due to α-H elimination of carbene intermediate.

### Substrate scope for the synthesis of quinoxaline *N*-oxides by Pt(II) catalysis

**Fig. 4:**
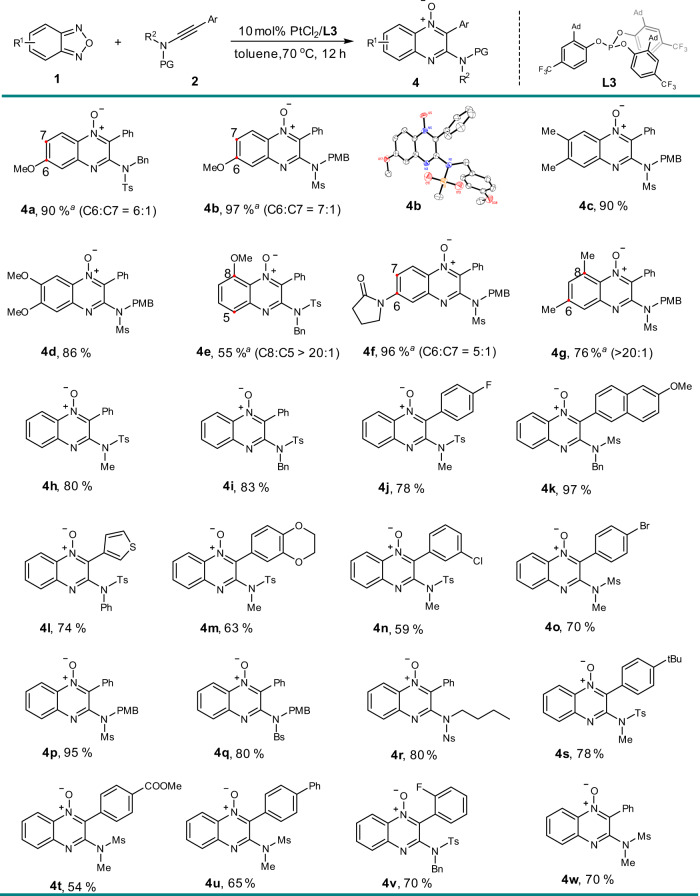
Scope with respect to the Pt(II)-catalyzed annulations. Reaction conditions: **1** (0.2 mmol), **2** (0.1 mmol), 10 mol% PtCl_2_ with 10 mol% **L3** in 1 ml toluene at 70 °C for 12 h, unless otherwise noted. Isolated yields were listed. ^*a*^Isomeric ratios were determined by ^1^H NMR of the crude product mixture.PMB *para*-methoxybenzyl, Ms methanesulfonyl, Ns 4-nitrobenzenesulfonyl, Bs 4-bromobenzenesulfonyl.

Then, the treatment of ynamides with diverse benzofurazans was conducted under platinum catalysis (Fig. 4). An array of substituted derivatives was explored to check the reaction with ynamides. Due to the positive conjugative effect of an electron-rich substituent on the nucleophilicity of adjacent nitrogen atom, the ynamide prefers to react with the proximal nitrogen to the β-methoxy (**4a**, **b**) or amide (**4f**) group, inducing good regio-selectivity. The solid state molecular structure of **4b** could be obtained by an X-ray single-crystal structure analysis and is shown in Fig. 4. Because of the steric effect, the reaction of α-substituted benzofurazans also showed excellent regio-selectivity (**4e**, **4g**). The non-substituted benzofurazan also worked well. Variations on the aryl moiety of the ynamide was investigated. A wide range of functional groups, including halogen (**4j**, **4n**, **4o**), thiophene (**4l**) and methoxy substituents (**4k**), were all tolerated, offering quinoxaline *N*-oxides in good to excellent yields. Ms, Bs and the easily removable nosyl protected ynamides also reacted smoothly (**4p**–**r**).

### Further modifications of quinoxaline *N*-oxide

**Fig. 5:**
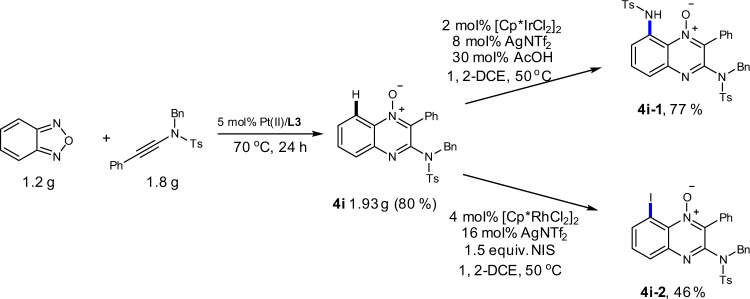
Synthetic transformations by C–H functionalization. Gram-scale synthesis of quinoxaline *N*-oxide **4i**. Iridium-catalyzed directed C–H bond amination. Rhodium-catalyzed directed C–H bond iodination.

A gram-scale synthesis of **4i** was completed in 80% yield by 5 mol% PtCl_2_/**L3**, which prove the synthetic feasibility of the Pt(II) catalytic system. By taking the advantage of *N*-oxides as directing groups, the further decoration could be accomplished via a direct C–H functionalization (Fig. 5). The quinoxaline *N*-oxide **4i** was suitable for iridium-/rhodium-catalyzed selective C8-amination and iodination^50,51^.

### Mechanism studies

**Fig. 6:**
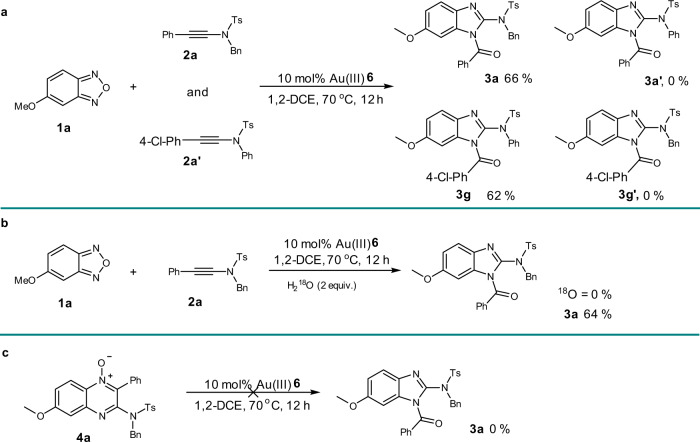
Mechanistic experiments. **a** Intermolecular control experiment. **b** ^18^O-labing experiment. **c** Exclusion of the transformation from **4a** to **3a**.

For gold(III)-catalyzed the formation of benzimidazole, an intermolecular control experiment did not show intermolecular cross-over (Fig. 6a), which suggests an intramolecular acyl migration process. Notably, in contrast with the both isomers of *N*-oxide were observed, only 1-acyl substituted benzimidazole was produced. It may indicate same kind of acyl migration precursor was produced whether the proximal or distal nitrogen to the methoxy group of **1a** attack the C–C triple bond initially. The acyl migration always prefers to the proximal N atom due to the higher nucleophilicity. The ^18^O-labeling experiment showed that the O atom of acyl group owes to the substrate rather than ambient H_2_^18^O (Fig. 6b). Moreover, the quinoxaline *N*-oxide **4a** could not be convert to benzimidazole under the standard condition (Fig. 6c).

**Fig. 7:**
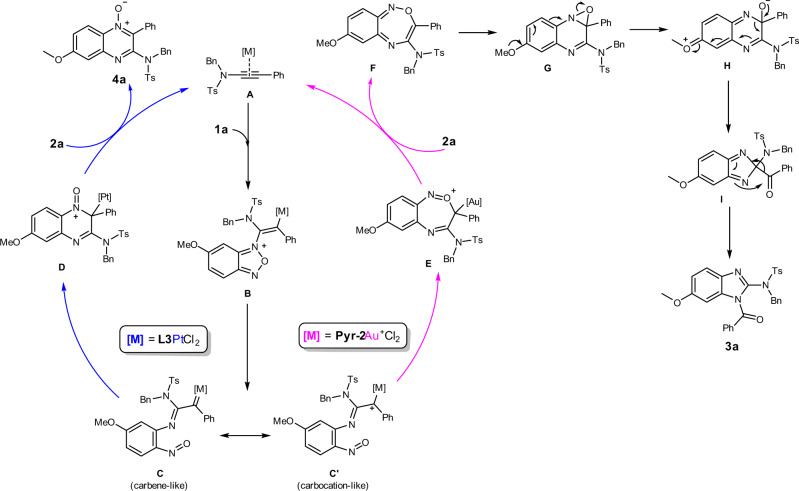
Proposed reaction mechanism for Au(III) and Pt(II) systems. The difference between gold(III) and platinum(II) catalysis.

A plausible reaction mechanism is depicted in Fig. 7. The α-imino gold or platinum carbene species **C**/**C′** is generated via initial nucleophilic attack of benzofurazan **1a** to the metal-ligated ynamide (**A**), followed by N–O bond cleavage. While the Pt(II) carbene prefers *N*-attack, leading to the six-membered ring **D**, the gold(III) carbene favors *O*-attack, furnishing the seven-membered ring intermediate **E**, which then releases intermediate **F** after ligand exchange. Following the electrocyclization and the second N–O bond cleavage, a ring contraction reaction gives the five-membered ring intermediate **I**. After intramolecular 1,2-acyl migration, the product **3a** is afforded eventually.

### Computational calculations

**Fig. 8:**
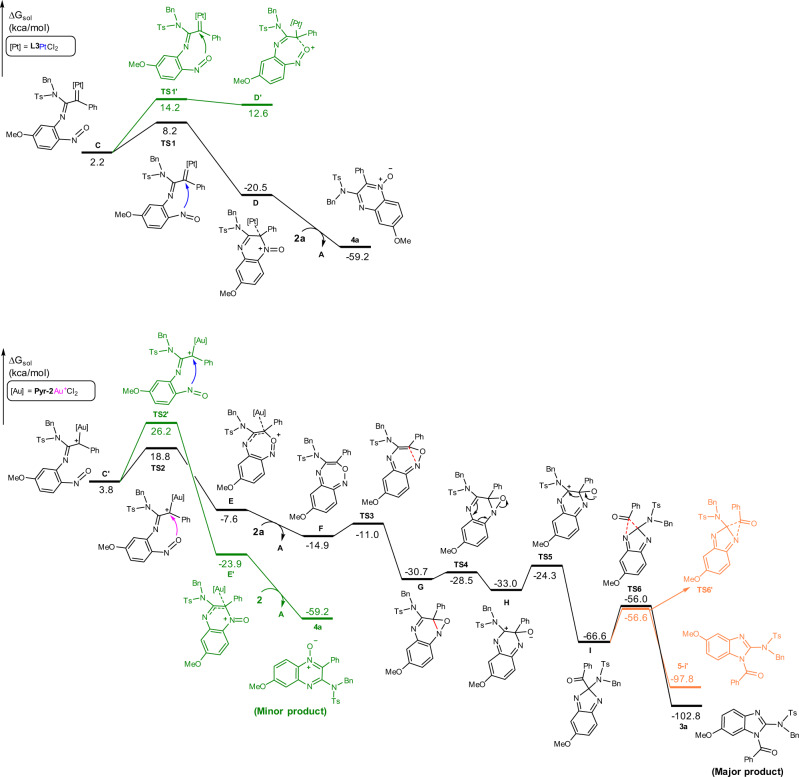
Mechanism investigation by computational calculations. Calculated free energy profile of the Pt(II) and Au(III) system.

In order to gain further insights into the reaction mechanism and to clarify the different annulation pathways of gold(III) and platinum(II) carbene intermediates, density functional theory (DFT) calculations were carried out (DFT calculations were performed by Gaussian program under the level of M06/6-311++G**/SDD-SMD(CH_2_Cl_2_)//B3LYP(D3BJ)/6-31G*/SDD. The CM5 atomic partial charges were calculated by Multiwfn program under the M06/6-311++G**/SDD-SMD(CH_2_Cl_2_) level. See Supplementary Information for computational details). Since the mechanism for the generation of Au/Pt carbene is similar to those of previous analogous reports^35,41^, here we do not discuss it but give the corresponding energetic details in Supplementary Information (Figs. S2, S3). Starting from metal-carbene species (**C**/**C′**), both annulation pathways are compared for Au(III) and Pt(II) system respectively, and the corresponding calculated free energy profiles are shown below. For the Pt(II) system (Fig. 8), the formation of six-membered ring occurs facilely via **TS1**, crossing a free energy activation barrier of 6.0 kcal/mol. The resulting complex **D** is lower than **C** by 22.7 kcal/mol, in which the product **4a** is already formed and can be easily released by ligand exchange. However, producing a seven-membered ring by *O*-attack is unfavorable. Although the activation barrier of 12.0 kcal/mol (**TS1′** relative to **C**) is accessible, the resulting complex **D′** is quite unstable, which is higher than **C** by 10.4 kcal/mol. This is also supported by the weak C–O bond (the resulted C–O bond length is 1.587 Å, which is obviously longer than normal C–O bonds, see Fig. S4 in Supplementary Information). Thus, the annulation en route to the seven-membered ring is reversible and is highly inclining towards **C**, whereas the six-membered ring annulation pathway is both kinetically and thermodynamically feasible. The calculated results well consist with the experimental observation that the Pt(II) catalyst shows specific selectivity for product **4a**.

**Fig. 9:**
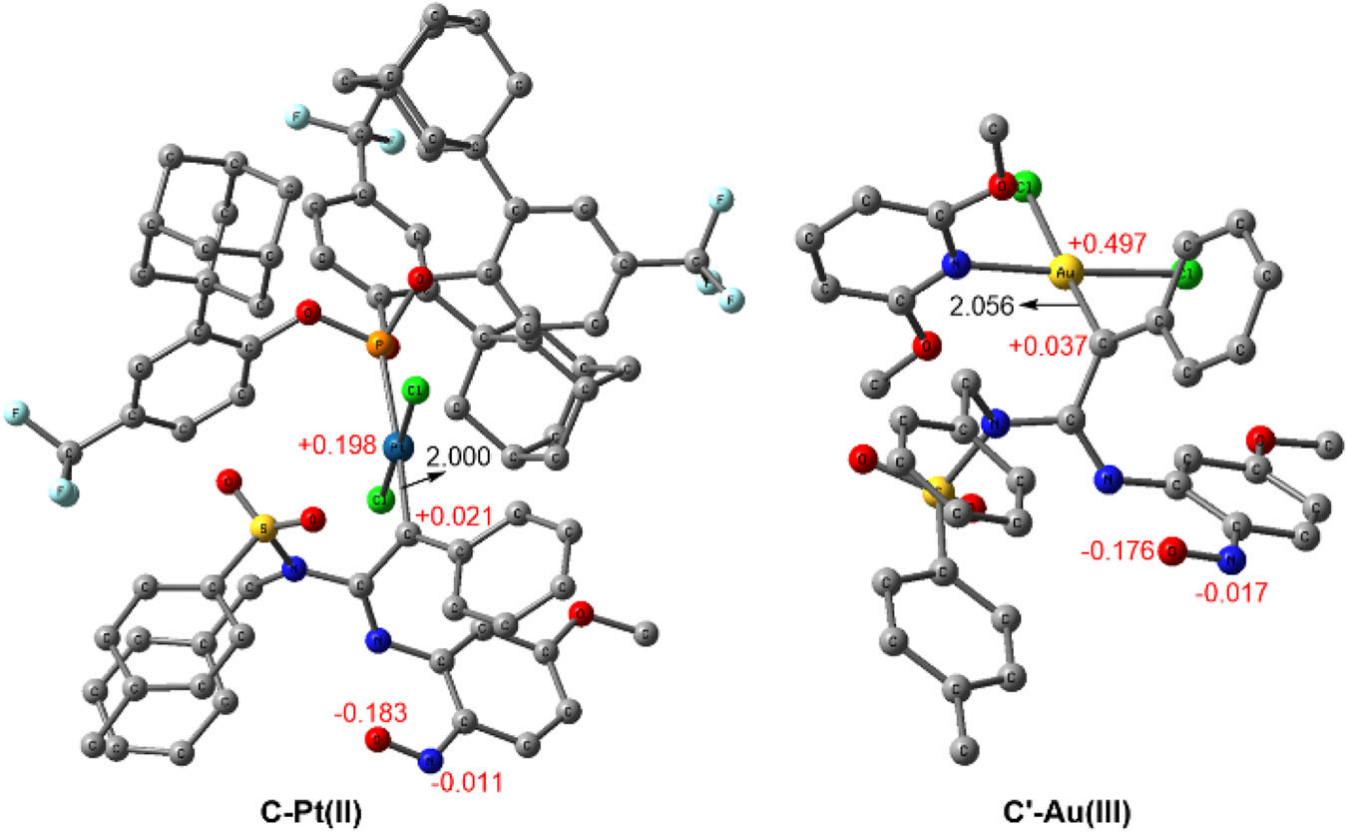
Gold(III) and Platinum(II) carbene intermediates. Selected bond lengths (black, Å) and CM5 atomic partial charges (red, au) for optimized geometries of Pt(II) and Au(III) carbene species. Hydrogen atoms are omitted for clarity.

On the contrary, the seven-membered ring cyclization process is preferable in the case of gold(III) carbene (Fig. 9). The nucleophilic attack of nitrosyl oxygen to the carbene carbon crosses a free energy activation barrier of 15.0 kcal/mol via **TS2**, leading to the seven-membered ring complex **E** with energy decreased by 11.4 kcal/mol. Alternatively, the attack of nitrosyl nitrogen atom to the carbene has to overcome a free energy activation barrier of 22.4 kcal/mol (**TS2′** relative to **C′**). Despite kinetic disfavor, we could not thoroughly exclude this route. Once the activation barrier (22.4 kcal/mol) could be accessible, the resulted six-membered ring complex **E′** is thermodynamically much more stable than **E** (by 16.3 kcal/mol). In fact, **4a**, released from **E′** via ligand exchange, serves as a side product. Nevertheless, the annulation via *O*-attack to form **E** is still kinetically more favorable as the major pathway. After ligand exchange to afford **F**, the electrocyclization undergoes facilely via **TS3** to give **G**, which is prone to N–O bond cleavage with an activation energy of only 2.2 kcal/mol (**TS4** relative to **G**). Next, a ring contraction takes place, resulting in the five-membered ring intermediate I with energy reduced by 33.6 kcal/mol (from **H** to **I**). In agreement with experimental result, the 1,2-acyl migration prefers the nitrogen proximal to methoxy group under thermodynamic control. Overall, the final benzimidazole product **3a** is thermodynamically more stable than the six-membered ring by-product **4a** by 43.6 kcal/mol. The calculation on the other gold(III) catalyst, PicAuCl_2_, was also carried out in contrary to above Au system, which further disclosed the annulation selectivity relied on the electronic character of gold carbene (see Fig. S6).

The optimized geometries and atomic partial charges for Au(III) and Pt(II) carbene intermediates are shown in Fig. 9. The Au(III) carbene is cationic species because of alkyne coordination instead of coordination to a Cl^−^ ion. In addition, the pyridine is a weaker electron-donating ligand than the phosphite **L3**. Therefore, the Au(III) center is much more electron deficient than the Pt(II) core, which is supported by the calculated partial atomic charges (+0.497 vs. +0.198). As a result, the π-back donation from Au(III) center to the carbene carbon is weaker than that from platinum to the carbene carbon, leading to the carbocation-like gold carbene **C′**. This is well supported by comparing the metal-carbene bond lengths and atomic charges of carbene atoms. The Au–C bond length of C′ is 2.056 Å, which is apparently longer than those of most prior Au carbene species,^2*c*^ while the Pt–C bond length of C is 2.000 Å, which is obviously shorter than the Au–C bond, indicating stronger π-back donation. The calculated partial atomic charges of the carbene carbon also show that the Au(III) carbene is obviously more cationic than Pt(II) carbene (+0.037 vs. +0.018). On the basis of the above results, the discrepancy between the current Au(III) and Pt(II) systems should be mainly attributed to the different electronic characteristics of the metal-carbene species. The Au(III) carbene favors the *O*-attack through a seven-membered ring owing to the carbocation-like trait (because the oxygen atom is more electron rich than the nitrogen atom, −0.176 vs. −0.017). By taking advantage of electron-rich aryl group to reduce the cationic character of gold carbene, only product **4a** was observed (see Supplementary Information). It supported that the high electrophilicity is crucial to the formation of seven-membered ring intermediate. In contrast, the Pt(II) carbene features carbene-like character and the less cationic carbene carbon is not effective to accept electrons from the strong electronegative *O*-atom (supported by the unfavorable *O*-attack and the weak resulted C–O bond, 1.587 Å, vide supra), but prefers the *N*-attack to form the more stable six-membered ring. We further explored the size effect of formed ring on the selectivity of annulation in theory. The calculations indicated that with extending the ring size, the electronic effect is not predominant. The formation of smaller ring is favorable (Figs. S7, S8). Although this was out of the scope of the reactions, it suggested the annulation selectivity may not only depend on electronic effect.

In conclusion, the difference between gold and platinum catalysts was studied in detail. The platinum catalyst shows higher chemo-selectivity for the synthesis of quinoxaline *N*-oxides than gold(I) catalysts. In contrast to Pt(II) catalysis, the gold(III) complex **6** enables the selective synthesis of benzimidazoles via tandem annulation/ ring contraction/ acyl migration. DFT calculations support the experimentally observed selectivity for gold(III) and Pt(II) systems well. Analysis of geometric and electronic structures of key metal-carbene intermediates reveals that the electronic effect is the main reason for the annulation selectivity. The Au(III) carbene shows carbocation-like character, favoring *O*-attack, while the Pt(II) carbene prefers forming a six-membered ring via *N*-attack, which rationalizes the divergent annulation routes. These insight in transition metal catalysis can help to on purpose switch to certain reaction channels of metal-carbene intermediates in future methodology development.

## Methods

### Representative procedure for Au(III)-catalyzed tandem annulation

A round bottom flask equipped with a magnetic stirrer bar was added 10 mol% 2,6-dimethoxypyrAuCl_3_ (4.4 mg), **1a** (0.4 mmol), **2a** (0.1 mmol) and 1,2-DCE (4 ml). The reaction was heated at 70 °C for 12 h. After cooling to room temperature, the solvent was reduced in vacuo, and the residue was purified by column chromatography (SiO_2_, hexanes/EtOAc = 10/1) to provide the title compound **3a** as a colorless soild.

### Representative procedure for Pt(II)-catalyzed formal [4 + 2] annulation

A round bottom flask equipped with a magnetic stirrer bar was added 10 mol% PtCl_2_ (2.6 mg), 10 mol% **L3** (9.2 mg), **1a** (0.2 mmol), **2a** (0.1 mmol) and toluene (1 ml). The reaction was heated at 70 °C for 12 h. After cooling to room temperature, the solvent was reduced in vacuo, and the residue was purified by column chromatography (SiO_2_, hexanes/EtOAc = 10/1 − 5/1) to provide the title compound **4a** as a colorless soild.

## Supplementary information

Supplementary Information

Description of Additional Supplementary Files

Dataset 1

## Data Availability

Additional data generated in this study have been available in the Supplementary Information file. For full characterization data of new compounds and experimental details, see Supplementary Methods, Notes and Figures in Supplementary Information file. For the energies and Cartesian coordinates, see Supplementary Data file. The X-ray crystallographic coordinates for structures **3i** and **4b** reported in this study have been deposited at the Cambridge Crystallographic Data Center (CCDC), under deposition number 1954158 (**3i**) and 1570451 (**4b**). These data can be obtained free of charge from The Cambridge Crystallographic Data Center via www.ccdc.cam.ac.uk/data_request/cif.
