# Supplementary material for: Dichotomy of platinum(II) and gold(III) carbene intermediates switching from N- to O-selectivity
Source: Nat Commun. 2022 Mar 30;13:1672. doi: 10.1038/s41467-022-29326-0 (PMC8967914; doi:10.1038/s41467-022-29326-0)
Supplement: Supplementary file 1 — Supplementary Information [file 41467_2022_29326_MOESM1_ESM.pdf]

Supplementary Information

Dichotomy of Platinum(II) and Gold(III) Carbene Intermediates Switching from *N*- to *O*-selectivity

Hongming Jin*[1,2], Wen-Yan Tong[3], Jing Zhang[4], Matthias Rudolph[2], Frank Rominger[2], Xu Shen[4], Shuanglin Qu*[3] & A. Stephen K. Hashmi*[2]

[1]College of Pharmacy, Experiment Center for Science and Technology, Nanjing University of Chinese Medicine, 210023 Nanjing, China.

[2]Organisch-Chemisches Institut, Universität Heidelberg, Im Neuenheimer Feld 270, D-69120 Heidelberg, Germany.

[3]College of Chemistry and Chemical Engineering, Hunan University, 410082 Changsha, China

[4]Jiangsu Key Laboratory for Pharmacology and Safety Evaluation of Chinese Materia Medica, State Key Laboratory Cultivation Base for TCM Quality and Efficacy, Nanjing University of Chinese Medicine, 210023 Nanjing, China

*e-mail: H.J. (hmjin@njucm.edu.cn), S.Q. (squ@hnu.edu.cn) or A.S.K.H (hashmi@hashmi.de).

Contents

1. **Supplementary Notes** S1
2. **Supplementary Methods** S2
3. **Supplementary References** S82

1. Supplementary Notes

Chemicals were purchased from commercial suppliers and used as delivered. The benzofurazan, 5-bromobenzofurazan and 5-methoxybenzofurazan are bought from SigmaAldrich. Other benzofurazan derivatives **1**, ynamides **2** and ligands (**L2 L3**) were prepared according to related literatures.[1-6] Deuterated solvents were bought from Euriso-Top. NMR spectra were, if not mentioned otherwise, recorded at room temperature on the following spectrometers: Bruker Avance-III-300, Bruker Avance DRX-300, Bruker-Avance DRX-500 and Bruker Avance-III-500. Chemical shifts are given in ppm and coupling constants in Hz. The following abbreviations were used for ^{1}H NMR spectra to indicate the signal multiplicity: s (singlet), brs (broad singlet), d (doublet), t (triplet), q (quartet), quint (quintet), sext (sextet), sept (septet) and m (multiplet) as well as combinations of them. When combinations of multiplicities are given the first character noted refers to the biggest coupling constant. All ^{13}C NMR spectra were measured with ^{1}H-decoupling. The multiplicities mentioned in these spectra [s (singlet, quaternary carbon), d (doublet, CH-group), t (triplet, CH_2-group), q (quartet, CH_3-group)] were determined by DEPT135 and HSQC spectra. Mass spectra (MS and HRMS) were determined at the chemistry department of the University of Heidelberg under the direction of Dr. J. Gross. EI^+ -spectra were measured on a JOEL JMS-700 spectrometer. For ESI^+-spectra a Bruker ApexQu FT-ICR-MS spectrometer was applied. Infrared Spectroscopy (IR) was processed on an FT-IR Bruker (IF528), IR Perkin Elmer (283) or FT-IR Bruker Vector 22. The solvent or matrix is denoted in brackets. For the most significant bands the wave number ν (cm^{-1}) is given. X-ray crystal structure analyses were measured at the chemistry department of the University of Heidelberg under the direction of Dr. F. Rominger on a Bruker Smart CCD or Bruker APEX-II CCD instrument using Mo-K_α-radiation. Diffraction intensities were corrected for Lorentz and polarization effects. An empirical absorption correction was applied using SADABS based on the Laue symmetry of reciprocal space. Heavy atom diffractions were solved by direct methods and refined against F2 with full matrix least square algorithm. Hydrogen atoms were either isotropically refined or calculated. The structures were solved and refined by Dr. F. Rominger using the SHELXTL software package. Gas Chromatography / Mass Spectrometry (GC/MS) spectra were measured on two different hardware systems: 1. HP 5972 Mass Selective Detector, coupled with a HP 5890 SERIES II plus gas chromatograph. 2. Agilent 5975C Mass Selective Detector, coupled with an Agilent 7890A gas chromatograph. In both cases, as a capillary column, an OPTIMA 5 cross-linked Methyl Silicone column (30 m x 0.32 mm, 0.25 μm) was employed and helium was used as the carrier gas. Gas Chromatography (GC) was carried out on a HP 5890 SERIES II plus gas chromatograph. As a capillary column, an OPTIMA 5 cross-linked Methyl Silicone column (30 m x 0.32 mm, 0.25 μm) was employed and nitrogen was used as the carrier gas. Melting Points were measured in open glass capillaries in a Büchi melting point apparatus (according to Dr. Tottoli) and were not corrected. Flash Column Chromatography was accomplished using Silica gel 60 (0.04 - 0.063 mm / 230 - 400 mesh ASTM) purchased from Macherey-Nagel or Aluminium oxide (neutral or basic) purchased from Macherey-Nagel. As eluents, mixtures of petroleum ether (PE), ethyl acetate (EA), dichloromethane (DCM) and diethylether (Et_2O) were used. Analytical Thin Layer Chromatography (TLC) was carried out on precoated Macherey-Nagel POLYGRAM® SIL G/UV254 or POLYGRAM® ALOX N/UV254 plastic sheets. Detection was accomplished using UV-light (254 nm), $KMnO_4$ (in 1.5M Na_2CO_3 (aq.)), molybdatophosphoric acid (5 % in ethanol), vanillin/H_2SO_4 (in ethanol) or anisaldehyde/HOAc (in ethanol). IUPAC names of the compounds described in the experimental section were determined with the program ACDLabs 12.0®.

2. Supplementary Methods

2.1 General procedures

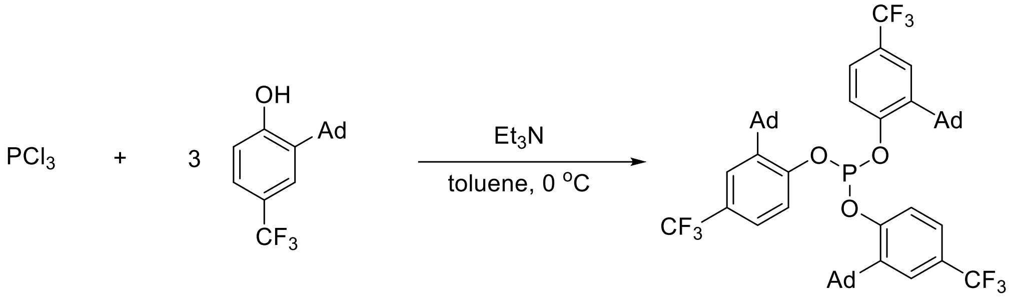

Supplementary Figure 1. The procedures for the synthesis of **L3**:

To a mixture of phosphorous trichloride (82 μL, 0.9 mmol) and 2-(adamantan-1-yl)-4-(trifluoromethyl)phenol (888 mg, 3 mmol) was added triethylamine (0.42 mL, 3 mmol) in toluene (20 mL) at 0 ºC. The mixture was refluxed for 6 hours, then cooled to room temperature and removed precipitated triethylamine hydrochloride. Removal of the volatiles in vacuo afforded a white solid that slowly crystallized at room temperature upon standing to give pure **L3** as a white solid (640 mg, 78%).

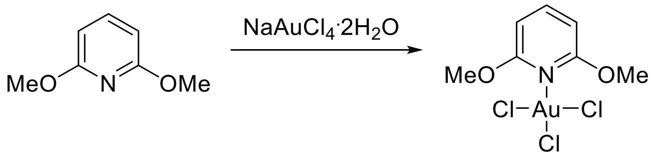

Supplementary Figure 2. The procedures for the synthesis of the gold complex **6**[7]:

To an aqueous solution of $NaAuCl_4 \cdot 2H_2O$ (199 mg, 0.5 mmol) was added 2,6-dimethoxypyridine (83.4 μL, 0.6 mmol). The mixture was stirred at room temperature for overnight. The yellow solid was filtered and washed by water (2×1 mL) and cool methanol (2×1 mL) (115 mg, 52 %).

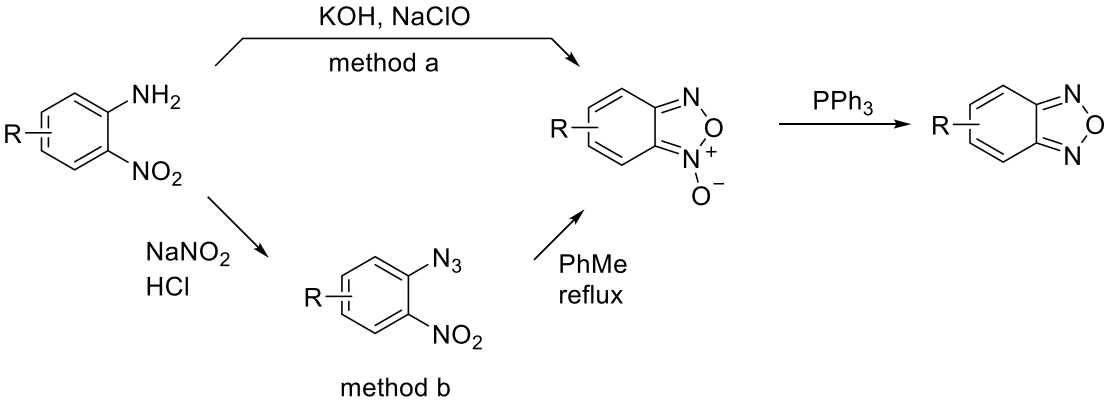

Supplementary Figure 3. The procedures for the preparation of benzofurazans[5]

Method a[5a]: To a mixture of *o*-nitroaniline (3 mmol) in 2 ml EtOH was added 1.5 ml aq. KOH (2 M) and 6 % NaClO solution at 0 ºC. Removing the ice bath and then rapid stirring for 30 mins, a brilliant yellow solid formed. The mixture was filtered and washed by a large amount of water and dried under reduced pressure.

The mixture of above crude product with PPh_3 (2.5 equiv.) was refluxed in PhMe for 3 h under N_2 atmosphere. After cooling to room temperature, the solvent was evaporated under reduced pressure. The residue was purified by column

chromatography to afford the benzofurazan.

Method b[5b]**:** To a solution of *o*-nitroaniline (5 mmol) in EtOAc at 0 °C was added conc. HCl (2 ml). Subsequently, a solution of $NaNO_2$ (6 mmol) in 5 ml H_2O was dropwise added over 15 mins with continued stirring. After keeping the reaction at 0 °C for 1 h, NaN_3 (6 mmol) was carefully added to the mixture at 0 °C. Then, the reaction was conducted at room temperature for 2-3 h. The mixture was poured into 50 ml H_2O, extracted with EtOAc (3×10 ml), dried over anhydrous Na_2SO_4 and concentrated under reduced pressure. The residue was purified by column chromatography to afford the 1-azido-2-nitrobenzene in nearly quantitative yield.

The 1-azido-2-nitrobenzene (5 mmol) was heated at 110 °C in PhMe (5 ml) for 1 h under N_2 atmosphere. Then, PPh_3 (12 mmol) was added directly. The resulting mixture was refluxed for an additional 3 h. After cooling to room temperature, the solvent was evaporated under reduced pressure. The crude product was purified by column chromatography to furnish the benzofurazan.

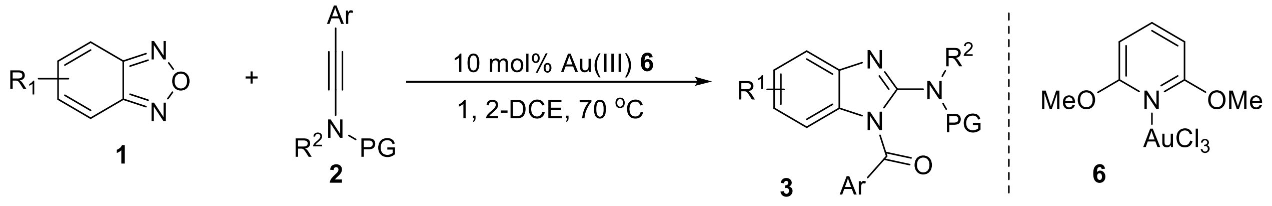

Supplementary Figure 4. General procedure for Au(III)-catalyzed tandem annulation.

A round bottom flask equipped with a magnetic stirrer bar was added 10 mol% 2,6-dimethoxypyr$AuCl_3$ (4.4 mg), **1** (0.4 mmol), **2** (0.1 mmol) and 1,2-DCE (4 ml). The reaction was heated at 70 °C for 12 h. After cooling to room temperature, the solvent was reduced in vacuo, and the residue was purified by column chromatography (SiO_2, hexanes/EtOAc = 10/1) to provide the title compound **5**. The characterization data of the products are listed in part 3.

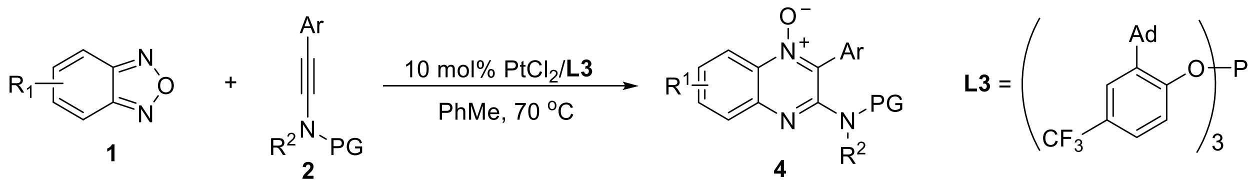

Supplementary Figure 5. General procedure for Pt(II)-catalyzed formal [4+2] annulation.

A round bottom flask equipped with a magnetic stirrer bar was added 10 mol% $PtCl_2$ (2.6 mg), 10 mol% **L3** (9.2 mg), **1** (0.2 mmol), **2** (0.1 mmol) and toluene (1 ml). The reaction was heated at 70 °C for 12 h. After cooling to room temperature, the solvent was reduced in vacuo, and the residue was purified by column chromatography (SiO_2, hexanes/EtOAc = 10/1 - 5/1) to provide the title compound. The characterization data of the products are listed in part 3.

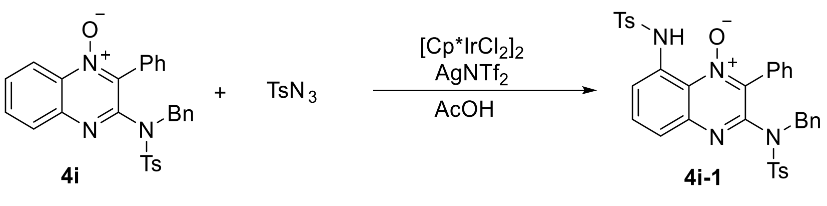

Supplementary Figure 6. Representative procedure for directed C-H amination.

A round bottom flask equipped with a magnetic stirrer bar was charged with [Cp*IrCl$_2$]$_2$ (2 mol%, 3.2 mg), AgNTf$_2$ (8 mol%, 3.2 mg), quinoxaline *N*-oxide **4i** (0.1 mmol), TsN$_3$ (0.15 mmol) and 1,2-dichloroethane (0.5 ml). The mixture heated at 50 °C for 12 h. After cooling to room temperature, the solvent was reduced in vacuo, and the residue was purified by column chromatography (SiO$_2$, hexanes/EtOAc = 5/1) to provide the title compound.

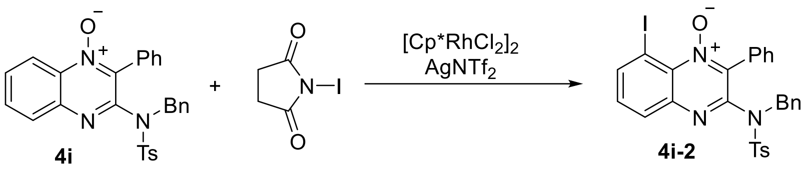

Supplementary Figure 7. Representative procedure for directed C-H iodination.

A round bottom flask equipped with a magnetic stirrer bar was charged with [Cp*RhCl$_2$]$_2$ (4 mol%, 2.5 mg), AgNTf$_2$ (16 mol%, 6.4 mg), quinoxaline *N*-oxide **3b** (0.1 mmol), NIS (0.15 mmol) and 1,2-dichloroethane (0.5 ml) under nitrogen atmosphere. The mixture was heated at 50 °C for 12 h. After cooling to room temperature, the solvent was reduced in vacuo, and the residue was purified by column chromatography (SiO$_2$, hexanes/EtOAc = 5/1) to provide the title compound.

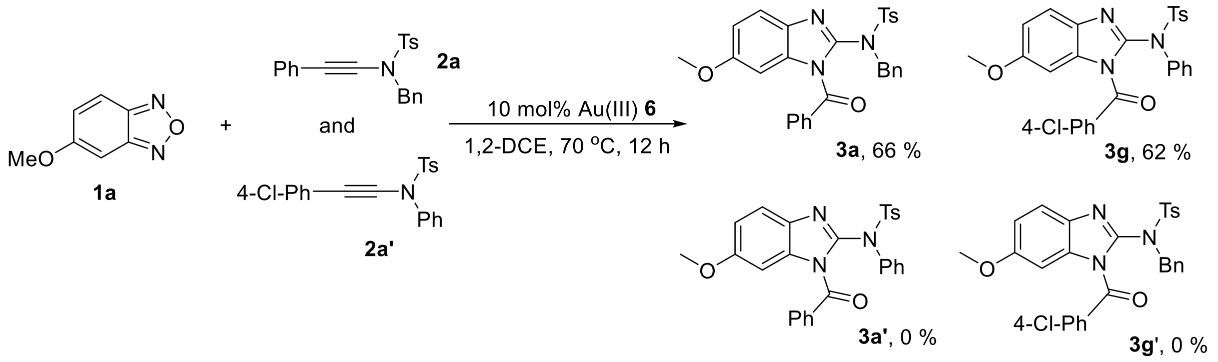

Supplementary Figure 8. Representative procedure for intermolecular control experiment.

A round bottom flask equipped with a magnetic stirrer bar was charged with 2,6-dimethoxypyrAuCl$_3$ (10 mol%, 4.4 mg), 5-methoxybenzofurazan **1a** (0.8 mmol), ynamides **2a** (0.1 mmol) and **2a'** (0.1 mmol) and 1, 2-DCE (8 ml). The mixture was heated at 70 °C for 12 h. After cooling to room temperature, the solvent was reduced in vacuo, and the residue was purified by column chromatography (SiO$_2$, hexanes/EtOAc = 10/1) to provide the title compound.

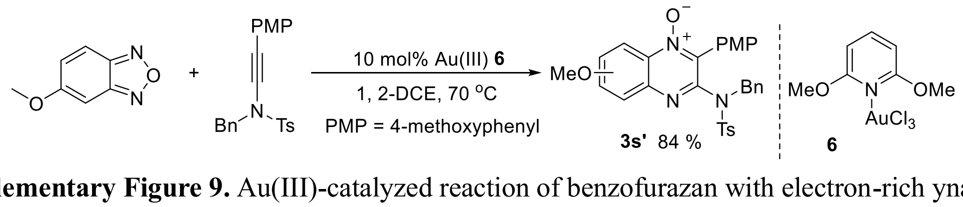

Supplementary Figure 9. Au(III)-catalyzed reaction of benzofurazan with electron-rich ynamide.

A round bottom flask equipped with a magnetic stirrer bar was added 10 mol% 2,6-dimethoxypyrAuCl$_3$ (4.4 mg), **1a** (0.4 mmol), *N*-benzyl-*N*-((4-methoxyphenyl)ethynyl)-4-methylbenzenesulfonamide (0.1 mmol) and 1,2-DCE (4 ml). The reaction was heated at 70 °C for 12 h. After cooling to room temperature, the solvent was reduced in vacuo, and the residue was purified by column chromatography (SiO$_2$, hexanes/EtOAc = 5/1) to provide the title compound **3s'**. The characterization data of the products are listed in part 3.

2.2 Characterization

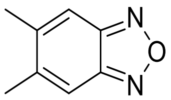

S1a, 5,6-dimethylbenzofurazan

Prepared by the method a. Total yield 80 %, colourless soild, mp: 87-88 °C; ^{1}H NMR (500 MHz, $CDCl_3$) δ = 7.46 (s, 2 H), 2.31 (s, 6 H) ppm; ^{13}C NMR (125 MHz, $CDCl_3$) δ = 148.9 (s), 143.1 (s), 113.7 (d), 21.1 (q) ppm; IR (ATR): $\tilde{\nu}$ = 2958, 2933, 1738, 1679, 1638, 1537, 1522, 1494, 1463, 1448, 1405, 1390, 1339, 1249, 1197, 1137, 1026, 995, 878, 836, 707 cm^{-1}; HRMS (EI) m/z calcd for $[C_8H_8N_2O]^+$: 148.0637; found: 148.0626.

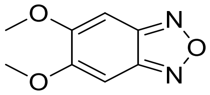

S1b, 5,6-dimethoxybenzofurazan

Prepared by the method b. Total yield 78 %, yellow soild, mp: 179-180 °C; ^{1}H NMR (500 MHz, $CDCl_3$) δ = 6.81 (s, 2 H), 3.93 (s, 6 H) ppm; ^{13}C NMR (125 MHz, $CDCl_3$) δ = 155.4 (s), 146.7 (s), 90.7 (d), 56.6 (q) ppm; IR (ATR): $\tilde{\nu}$ = 3070, 2944, 1737, 1635, 1593, 1542, 1518, 1492, 1445, 1407, 1364, 1223, 1177, 1153, 1140, 998, 870, 852, 828, 811, 750, 727 cm^{-1}; HRMS (EI) m/z calcd for $[C_8H_8N_2O_3]^+$: 180.0535; found: 180.0532.

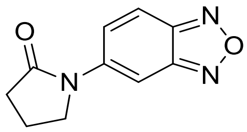

S1c, 1-(benzofurazan-5-yl)pyrrolidin-2-one

Prepared by the method b. Total yield 77 %, colourless soild, mp: 138-140 °C; ^{1}H NMR (500 MHz, $CDCl_3$) δ = 8.35-8.29 (m, 1 H), 7.77-7.71 (m, 1 H), 7.39 (s, 1 H), 3.88 (t, *J* = 7.0 Hz, 2 H), 2.62 (t, *J* = 8.0 Hz, 2 H), 2.23-2.14 (m, 2 H) ppm; ^{13}C NMR (125 MHz, $CDCl_3$) δ = 175.0 (s), 149.3 (s), 147.0 (s), 142.0 (s), 127.6 (d), 116.6 (d), 100.7 (d), 48.9 (t), 32.8 (t), 17.8 (t) ppm; IR (ATR): $\tilde{\nu}$ = 3113, 3058, 2962, 1698, 1625, 1538, 1476, 1461, 1419, 1389, 1352, 1332, 1294, 1246, 1202, 1168, 1155, 1096, 1028, 1008, 876, 849, 821, 786, 715, 672, 624 cm^{-1}; HRMS (EI) m/z calcd for $[C_{10}H_9N_3O_2]^+$: 203.0689; found: 203.0688.

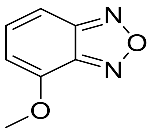

S1d, 4-methoxybenzofurazan

Prepared by the method b. Total yield 85 %, colourless soild, mp: 91-92 °C; ^{1}H NMR (500 MHz, $CDCl_3$) δ = 7.32-7.23 (m, 2 H), 6.47-6.44 (m, 1 H), 3.98 (s, 3 H) ppm; ^{13}C NMR (125 MHz, $CDCl_3$) δ = 150.58 (s), 148.8 (s), 145.1 (s), 132.5 (d), 107.9 (d), 105.5 (d), 56.3 (q) ppm; IR (ATR): $\tilde{\nu}$ = 3062, 1633, 1548, 1495, 1463, 1446, 1414, 1376, 1350, 1326, 1261, 1233, 1184, 1144, 1009, 974, 879, 830, 816, 741, 631 cm^{-1}; HRMS (EI) m/z calcd for $[C_7H_6N_2O_2]^+$: 150.0424; found: 150.0422.

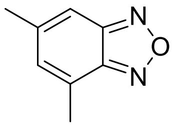

S1e, 4,6-dimethylbenzofurazan

Prepared by the method a. Total yield 81 %, colourless soild, mp: 57-58 °C; ^{1}H NMR (500 MHz, $CDCl_3$) δ = 7.27 (s, 1 H), 6.87 (s, 1 H), 2.54 (s, 3 H), 2.34 (s, 3 H) ppm; ^{13}C NMR (125 MHz, $CDCl_3$) δ = 149.8 (s), 149.6 (s), 142.5 (s), 132.9 (d), 126.8 (s), 110.7 (d), 22.4 (q), 17.4 (q) ppm; IR (ATR): $\tilde{\nu}$ = 2951, 2922, 1740, 1676, 1634, 1561, 1479, 1443, 1402, 1376, 1334, 1204, 1039, 1005, 946, 904, 878, 848, 836, 806, 769, 656 cm^{-1}; HRMS (EI) m/z calcd for $[C_8H_8N_2O]^+$: 148.0637; found: 148.0643.

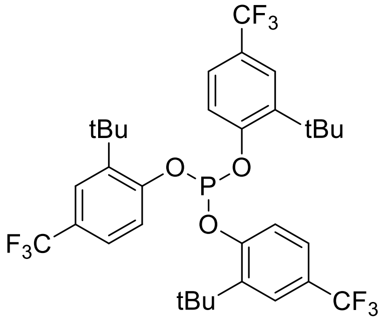

L2[6]: Yield 84 %, colourless soild, mp: 123-124 °C; ^{1}H NMR (500 MHz, $CDCl_3$) δ = 7.54 (s, 3 H), 7.46-7.37 (m, 6 H), 1.30 (s, 27 H) ppm; ^{31}P NMR (200 MHz, $CDCl_3$): δ = 130.3 ppm; ^{19}F NMR (471 MHz, $CDCl_3$): δ = -61.98 ppm.

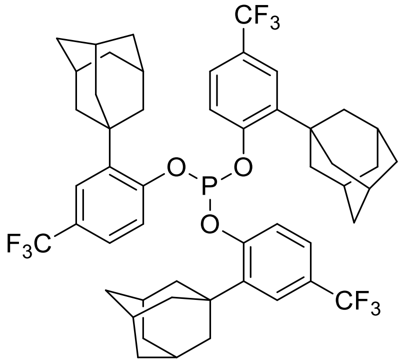

L3: Yield 78 %, colourless soild, mp: 226-227 °C; ^{1}H NMR (300 MHz, $CDCl_3$) δ = 7.50 (s, 3 H), 7.37-7.26 (m, 6 H), 2.04-1.82 (m, 27 H), 1.67-1.43 (m, 18 H) ppm; ^{13}C NMR (100 MHz, C_6D_6) δ = 154 (s), 140.9 (*J* = 1.9 Hz, s), 126.2 (*J* = *32* Hz, s), 125.4 (*J* = 3.4 Hz, d), 124.9 (*J* = 270 Hz, s), 124.2 (*J* = 2.4 Hz, d), 119.4 (*J* = 20.8 Hz, d), 40.1 (t), 37.2 (s), 36.3 (t), 28.8 (d) ppm; ^{31}P NMR (120 MHz, $CDCl_3$): δ = 130.5 ppm; ^{19}F NMR (283 MHz, $CDCl_3$): δ = -61.93 ppm; IR (ATR): $\tilde{\nu}$ = 2904, 2854, 1738, 1612, 1493, 1454, 1415, 1332, 1315, 1286, 1256, 1219, 1185, 1161, 1118, 1101, 1089, 1034, 977, 925, 905, 853, 821, 795, 767, 720, 690, 654, 638, 611 cm^{-1}; HRMS (DART) m/z calcd for $[M+H]^+$: 917.3740; found: 917.3721.

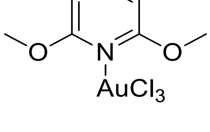

Catalyst 6: Yield 52 %, yellow soild, mp: 138-140 °C; ^{1}H NMR (400 MHz, CD_2Cl_2) δ = 7.97 (t, *J* = 8.0 Hz, 1 H), 6.62 (d, *J* = 8.4 Hz, 2 H), 4.08 (s, 6 H) ppm; ^{13}C NMR (125 MHz, $CDCl_3$) δ = 160.8 (s), 147.6 (d), 100.9 (d), 58.2 (q) ppm; IR (ATR): $\tilde{\nu}$ = 1611, 1583, 1484, 1455, 1413, 1269, 1104, 890, 775, 734, 726 cm^{-1}; HRMS (ESI) m/z calcd

for [M+Na]+: 463.9257; found: 463.9253.

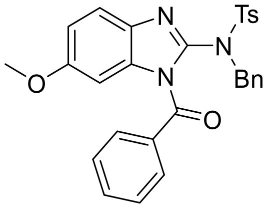

3a, N-(1-benzoyl-6-methoxy-1H-benzo[d]imidazol-2-yl)-N-benzyl-4-methylbenzenesulfonamide

Yield 70 %, colourless soild, mp: 173-175 °C; ^{1}H NMR (400 MHz, CD_2Cl_2) δ = 7.58-7.50 (m, 1 H), 7.50-7.36 (m, 5 H), 7.32-7.09 (m, 9 H), 6.85-6.80 (m, 1 H), 6.54-6.51 (m, 1 H), 4.59 (s, 2 H), 3.57 (s, 3 H), 2.35 (s, 3 H) ppm; ^{13}C NMR (100 MHz, CD_2Cl_2) δ = 167.8 (s), 157.6 (s), 145.0 (s), 144.5 (s), 134.5 (s), 134.32 (s), 134.30 (s), 133.4 (d), 133.3 (s), 130.4 (d), 130.0 (d), 129.7 (d), 128.5 (d), 128.3 (d), 128.29 (d), 128.1 (d), 120.2 (d), 113.0 (d), 97.2 (d), 55.7 (q), 54.0 (t), 21.4 (q) ppm; IR (ATR): $\tilde{\nu}$ = 2941, 1713, 1616, 1597, 1485, 1361, 1309, 1206, 1164, 1026, 801, 659 cm^{-1}; HRMS (ESI) m/z calcd for [M+Na]+: 534.1458; found: 534.1462.

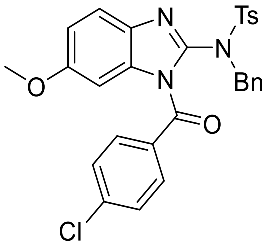

3b, N-benzyl-N-(1-(4-chlorobenzoyl)-6-methoxy-1H-benzo[d]imidazol-2-yl)-4-methylbenzenesulfonamide

Yield 71 %, colourless soild, mp: 188-189 °C; ^{1}H NMR (400 MHz, CD_2Cl_2) δ = 7.51-7.44 (m, 1 H), 7.38 (d, *J*= 8.4 Hz, 2 H), 7.27 (d, *J* = 8.4 Hz, 2 H), 7.24-7.12 (m, 9 H), 6.88-6.82 (m, 1 H), 6.74-6.70 (m, 1 H), 4.57 (s, 2 H), 3.63 (s, 3 H), 2.34 (s, 3 H) ppm; ^{13}C NMR (100 MHz, CD_2Cl_2) δ = 167.0 (s), 157.9 (s), 145.2 (s), 144.4 (s), 139.5 (s), 134.4 (s), 134.3 (s), 134.2 (s), 133.0 (s), 131.9 (s), 131.7 (d), 130.2 (d), 129.8 (d), 128.8 (d), 128.4 (d), 128.3 (d), 120.3 (d), 113.2 (d), 97.2 (d), 55.6 (q), 54.1 (t), 21.4 (q) ppm; IR (ATR): $\tilde{\nu}$ = 2951, 1715, 1597, 1437, 1277, 1161, 1087, 906, 754, 697, 589 cm^{-1}; HRMS (ESI) m/z calcd for [M+Na]+: 568.1068; found: 568.1072.

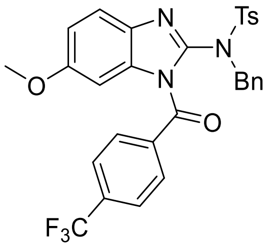

3c, N-benzyl-N-(6-methoxy-1-(4-(trifluoromethyl)benzoyl)-1H-benzo[d]imidazol-2-yl)-4-methylbenzenesulfonamide

Yield 72 %, colourless soild, mp: 161-162 °C; ^{1}H NMR (400 MHz, CD_2Cl_2) δ = 7.53-7.47 (m, 1 H), 7.42-7.32 (m, 6 H), 7.26-7.09 (m, 7 H), 6.92-6.86 (m, 2 H), 4.54 (s, 2 H), 3.67 (s, 3 H), 2.34 (s, 3 H) ppm; ^{13}C NMR (100 MHz,

CD_2Cl_2) δ = 167.3 (s), 158.2 (s), 145.5 (s), 144.4 (s), 137.2 (*J* = 1.1 Hz, s), 134.5 (s), 134.4 (s), 133.9 (*J* = 32.3 Hz, s), 132.8 (s), 130.4 (d), 129.9 (d), 128.64 (d), 128.60 (d), 128.5 (d), 125.6 (*J* = 3.7 Hz, d), 123.8 (*J* = 271 Hz, s), 120.5 (d), 113.6 (d), 97.4 (d), 55.9 (q), 54.1 (t), 21.6 (q) ppm; IR (ATR): $\tilde{\nu}$ = 2928, 1719, 1624, 1587, 1337, 1164, 1089, 1067, 1019, 895, 831, 814, 776, 743, 705, 692, 615 cm^{-1}; HRMS (ESI) m/z calcd for $[M+Na]^+$: 602.1332; found: 602.1330.

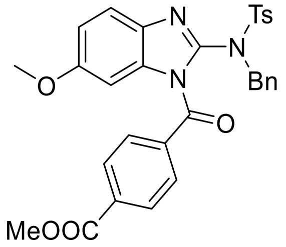

3d, methyl 4-(2-(N-benzyl-4-methylphenylsulfonamido)-6-methoxy-1H-benzo[d]imidazole-1-carbonyl)benzoate

Yield 64 %, colourless soild, mp: 205-206 °C; ^1H NMR (400 MHz, CD_2Cl_2) δ = 7.82 (d, *J* = 8.8 Hz, 2 H), 7.51-7.46 (m, 1 H), 7.40-7.31 (m, 4 H), 7.25-7.12 (m, 7 H), 6.90-6.84 (m, 1 H), 6.81-6.76 (m, 1 H), 4.53 (s, 2 H), 3.88 (s, 3 H), 3.64 (s, 3 H), 2.34 (s, 3 H) ppm; ^{13}C NMR (100 MHz, CD_2Cl_2) δ = 167.5 (s), 165.9 (s), 157.9 (s), 145.2 (s), 144.4 (s), 137.3 (s), 134.4 (s), 134.0 (s), 133.9 (s), 132.9 (s), 130.3 (d), 130.0 (d), 129.8 (d), 129.5 (d), 128.43 (d), 128.41 (d), 128.3 (d), 120.4 (d), 113.4 (d), 97.4 (d), 55.7 (q), 54.0 (t), 52.4 (q), 21.4 (q) ppm; IR (ATR): $\tilde{\nu}$ = 2954, 1720, 1700, 1635, 1590, 1436, 1363, 1274, 1165, 1105, 810, 712, 670 cm^{-1}; HRMS (ESI) m/z calcd for $[M+Na]^+$: 592.1513; found: 592.1519.

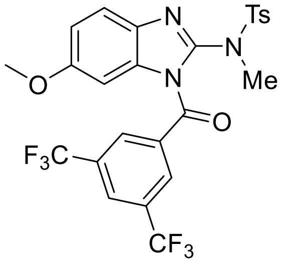

3e, N-(1-(3,5-bis(trifluoromethyl)benzoyl)-6-methoxy-1H-benzo[d]imidazol-2-yl)-N,4-dimethylbenzenesulfonamide

Yield 81 %, colourless soild, mp: 167-168 °C; ^1H NMR (400 MHz, CD_2Cl_2) δ = 8.09 (s, 3 H), 7.50-7.43 (m, 2 H), 7.26 (d, *J* = 8.4 Hz, 2 H), 7.19 (d, *J* = 8.4 Hz, 2 H), 6.97-6.92 (m, 1 H), 3.81 (s, 3 H), 2.81 (s, 3 H), 2.33 (s, 3 H) ppm; ^{13}C NMR (100 MHz, CD_2Cl_2) δ = 165.9 (s), 158.7 (s), 145.6 (s), 144.8 (s), 136.7 (s), 134.6 (s), 133.8 (s), 131.7 (*J* = 33 Hz, s), 131.0 (s), 130.0 (*J* = 3.2 Hz, d), 129.8 (d), 128.4 (d), 126.0 (*J* = 3.7 Hz, d), 122.9 (*J* = 271 Hz, s), 120.5 (d), 114.2 (d), 97.8 (d), 56.0 (q), 36.7 (q), 21.4 (q) ppm; IR (ATR): $\tilde{\nu}$ = 2970, 2929, 1720, 1684, 1597, 1343, 1288, 1179, 1064, 1014, 899, 848, 787, 716, 682, 615 cm^{-1}; HRMS (ESI) m/z calcd for $[M+Na]^+$: 594.0893; found: 594.0895.

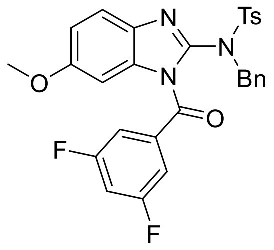

3f, N-benzyl-N-(1-(3,5-difluorobenzoyl)-6-methoxy-1H-benzo[d]imidazol-2-yl)-4-methylbenzenesulfonamide

Yield 73 %, colourless soild, mp: 189-191 °C; ^{1}H NMR (400 MHz, CD_2Cl_2) δ = 7.50-7.44 (m, 1 H), 7.41 (d, J = 8.4 Hz, 2 H), 7.22 (d, J = 8.4 Hz, 2 H), 7.20-7.07 (m, 5 H), 7.05-6.96 (m, 1 H), 6.96-6.88 (m, 2 H), 6.88-6.82 (m, 1 H), 6.71-6.66 (m, 1 H), 4.52 (s, 2 H), 3.63 (s, 3 H), 2.35 (s, 3 H) ppm; ^{13}C NMR (100 MHz, CD_2Cl_2) δ = 165.8 (s), 162.5 (J = 249.4 Hz, s), 162.4 (J = 249.4 Hz, s), 158.0 (s), 145.3 (s), 144.2 (s), 136.8 (J = 8.5 Hz, s), 134.4 (s), 134.1 (s), 133.8 (s), 132.8 (s), 129.9 (d), 129.8 (d), 128.5 (d), 128.4 (d), 128.3 (d), 120.5 (d), 113.3 (J = 19.4 Hz, d), 113.2 (J = 16.1 Hz, d), 108.6 (J = 25.2 Hz, d), 97.4 (d), 55.8 (q), 54.2 (t), 21.4 (q) ppm; IR (ATR): $\tilde{\nu}$ = 2957, 1716, 1616, 1598, 1482, 1362, 1164, 1123, 833, 758, 658 cm^{-1}; HRMS (ESI) m/z calcd for $[M+Na]^+$: 570.1270; found: 570.1275.

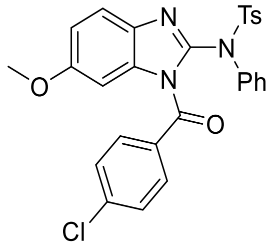

3g, N-(1-(4-chlorobenzoyl)-6-methoxy-1H-benzo[d]imidazol-2-yl)-4-methyl-N-phenylbenzenesulfonamide

Yield 64 %, colourless soild, mp: 191-192°C; ^{1}H NMR (400 MHz, CD_2Cl_2) δ = 7.63 (d, J = 8.8 Hz, 2 H), 7.54 (d, J= 8.8 Hz, 1 H), 7.44 (d, J = 8.8 Hz, 2 H), 7.36 (d, J = 8.0 Hz, 2 H), 7.21-7.10 (m, 5 H), 7.00-6.94 (m, 2 H), 6.92-6.87 (m, 1 H), 6.83-6.80 (m, 1 H), 3.67 (s, 3 H), 2.33 (s, 3 H) ppm; ^{13}C NMR (100 MHz, CD_2Cl_2) δ = 167.0 (s), 158.4 (s), 145.1 (s), 143.8 (s), 140.4 (s), 137.8 (s), 134.8 (s), 134.1 (s), 133.9 (s), 132.1 (s), 132.0 (d), 129.37 (d), 129.34 (d), 129.0 (d), 128.8 (d), 128.5 (d), 128.2 (d), 121.0 (d), 113.7 (d), 97.4 (d), 55.9 (q), 21.5 (q) ppm; IR (ATR): $\tilde{\nu}$ = 2938, 1718, 1615, 1604, 1433, 1309, 1161, 1087, 821, 748, 649 cm^{-1}; HRMS (ESI) m/z calcd for $[M+Na]^+$: 554.0912; found: 554.0912.

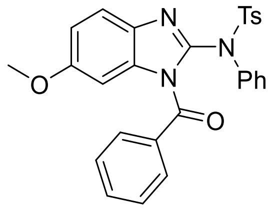

3h, N-(1-benzoyl-6-methoxy-1H-benzo[d]imidazol-2-yl)-4-methyl-N-phenylbenzenesulfonamide

Yield 60 %, colourless soild, mp: 188-189 °C; ^{1}H NMR (400 MHz, CD_2Cl_2) δ = 7.76-7.59 (m, 3 H), 7.57-7.29 (m, 5 H), 7.25-7.04 (m, 5 H), 7.02-6.85 (m, 3 H), 6.80-6.70 (s, 1 H), 3.64 (s, 3 H), 2.33 (s, 3 H) ppm; ^{13}C NMR (100 MHz,

CD_2Cl_2) δ = 167.8 (s), 158.2 (s), 144.8 (s), 143.8 (s), 137.8 (s), 134.6 (s), 134.1 (s), 133.9 (s), 133.8 (d), 133.5 (s), 130.5 (d), 129.2 (d), 128.9 (d), 128.8 (d), 128.6 (d), 128.4 (d), 128.3 (d), 120.7 (d), 113.5 (d), 97.3 (d), 55.7 (q), 21.5 (q) ppm; IR (ATR): $\tilde{\nu}$ = 2943, 1712, 1606, 1597, 1433, 1377, 1308, 1164, 1076, 801, 659, 549 cm^{-1}; HRMS (ESI) m/z calcd for $[M+Na]^+$: 520.1301; found: 520.1303.

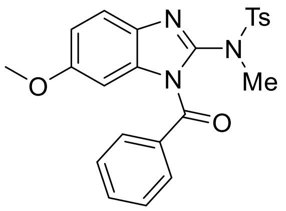

3i, N-(1-benzoyl-6-methoxy-1H-benzo[d]imidazol-2-yl)-N,4-dimethylbenzenesulfonamide

Yield 54 %, colourless soild, mp: 175-177 °C; ^{1}H NMR (500 MHz, $CDCl_3$) δ = 7.68 (d, *J* = 8.0 Hz, 2 H), 7.63-7.57 (m, 1 H), 7.50-7.44 (m, 3 H), 7.35 (d, *J* = 8.5 Hz, 2 H), 7.19-7.14 (m, 3 H), 6.95-6.87 (m, 1 H), 3.75 (s, 3 H), 2.95 (s, 3 H), 2.33 (s, 3 H) ppm; ^{13}C NMR (100 MHz, CD_2Cl_2) δ = 168.4 (s), 158.1 (s), 145.5 (s), 144.8 (s), 134.4 (s), 134.1 (s), 134.0 (s), 133.5 (d), 131.9 (s), 130.0 (d), 129.7 (d), 128.4 (d), 128.3 (d), 120.3 (d), 113.9 (d), 97.3 (d), 55.8 (q), 37.1 (q), 21.7 (q) ppm; IR (ATR): $\tilde{\nu}$ = 2913, 1713, 1608, 1597, 1420, 1305, 1108, 1049, 821, 666 cm^{-1}; HRMS (ESI) m/z calcd for $[M+Na]^+$: 458.1145; found: 458.1147.

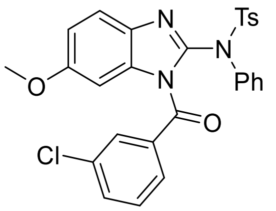

3j, N-(1-(3-chlorobenzoyl)-6-methoxy-1H-benzo[d]imidazol-2-yl)-4-methyl-N-phenylbenzenesulfonamide

Yield 61 %, colourless soild, mp: 178-180 °C; ^{1}H NMR (400 MHz, CD_2Cl_2) δ = 7.65-7.59 (m, 2 H), 7.59-7.51 (m, 2 H), 7.47-7.41 (m, 1 H), 7.35 (d, *J* = 8.4 Hz, 2 H), 7.21-7.10 (m, 5 H), 6.97-6.88 (m, 4 H), 3.69 (s, 3 H), 2.32 (s, 3 H) ppm; ^{13}C NMR (100 MHz, CD_2Cl_2) δ = 166.7 (s), 158.4 (s), 145.0 (s), 143.5 (s), 137.6 (s), 135.5 (s), 134.8 (s), 134.6 (s), 133.9 (s), 133.6 (d), 130.3 (d), 130.1 (d), 129.2 (d), 128.9 (d), 128.7 (d), 128.6 (d), 128.4 (d), 128.0 (d), 120.9 (d), 113.8 (d), 97.4 (d), 55.8 (q), 21.4 (q) ppm; IR (ATR): $\tilde{\nu}$ = 2952, 1718, 1607, 1423, 1267, 1166, 885, 734, 667 cm^{-1}; HRMS (ESI) m/z calcd for $[M+Na]^+$: 554.0912; found: 554.0910.

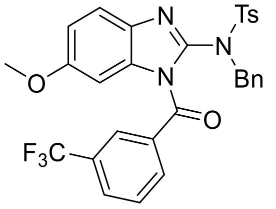

3k, N-benzyl-N-(6-methoxy-1-(3-(trifluoromethyl)benzoyl)-1H-benzo[d]imidazol-2-yl)-4-methylbenzenesulfonamide

Yield 62 %, colourless soild, mp: 156-157 °C; ^{1}H NMR (400 MHz, CD_2Cl_2) δ = 8.00 (s, 1 H), 7.81-7.75 (m, 1 H), 7.51-7.45 (m, 1 H), 7.44-7.39 (m, 2 H), 7.30-7.10 (m, 9 H), 6.88-6.83 (m, 1 H), 6.61-6.57 (m, 1 H), 4.55 (s, 2 H),

3.59 (s, 3 H), 2.35 (s, 3 H) ppm; ^{13}C NMR (100 MHz, CD_2Cl_2) δ = 166.8 (s), 158.1 (s), 145.3 (s), 144.7 (s), 134.5 (s), 134.4 (s), 134.3 (s), 134.2 (s), 133.4 (s), 133.3 (d), 131.2 (J = 32.9 Hz, s), 130.1 (d), 129.9 (d), 129.7 (J = 3.6 Hz, d), 129.4 (d), 128.5 (d), 128.49 (d), 128.42 (d), 127.6 (J = 3.6 Hz, d), 123.8 (J = 271 Hz, s), 120.6 (d), 113.4 (d), 97.4 (d), 55.8 (q), 54.4 (t), 21.4 (q) ppm; IR (ATR): $\tilde{\nu}$ = 2955, 2934, 1720, 1689, 1594, 1476, 1361, 1174, 805, 682 cm^{-1}; HRMS (ESI) m/z calcd for $[M+Na]^+$: 602.1332; found: 602.1341.

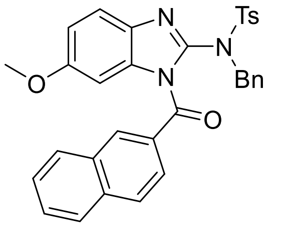

3l, N-(1-(2-naphthoyl)-6-methoxy-1H-benzo[d]imidazol-2-yl)-N-benzyl-4-methylbenzenesulfonamide

Yield 60 %, colourless soild, mp: 199-200 °C; ^{1}H NMR (400 MHz, CD_2Cl_2) δ = 8.02 (s, 1 H), 7.85 (d, J = 8.0 Hz, 1 H), 7.77 (d, J = 8.4 Hz, 1 H), 7.70 (d, J = 8.0 Hz, 1 H), 7.61-7.42 (m, 6 H), 7.25-7.02 (m, 7 H), 6.85-6.80 (m, 1 H), 6.49 (d, J = 1.6 Hz, 1 H), 4.58 (s, 2 H), 3.48 (s, 3 H), 2.34 (s, 3 H) ppm; ^{13}C NMR (100 MHz, CD_2Cl_2) δ = 167.8 (s), 157.6 (s), 145.0 (s), 144.8 (s), 135.7 (s), 134.5 (s), 134.4 (s), 133.5 (s), 132.2 (d), 130.6 (s), 129.72 (d), 129.71 (d), 128.9 (d), 128.5 (d), 128.4 (d), 128.3 (d), 128.1 (d), 127.8 (d), 127.0 (d), 125.7 (d), 120.3 (d), 112.8 (d), 97.4 (d), 55.6 (q), 54.3 (t), 21.4 (q) ppm; IR (ATR): $\tilde{\nu}$ = 2923, 2852, 1713, 1687, 1583, 1485, 1361, 1243, 1165, 1068, 948, 803, 676, 547 cm^{-1}; HRMS (ESI) m/z calcd for $[M+Na]^+$: 584.1614; found: 584.1615.

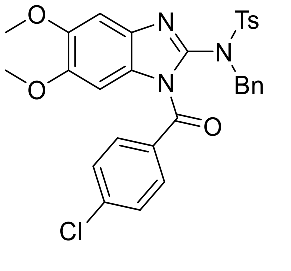

3m, N-benzyl-N-(1-(4-chlorobenzoyl)-5,6-dimethoxy-1H-benzo[d]imidazol-2-yl)-4-methylbenzenesulfonamide

Yield 70 %, colourless oil; ^{1}H NMR (400 MHz, $CDCl_3$) δ = 7.38 (d, J = 8.4 Hz, 2 H), 7.25-7.15 (m, 9 H), 7.13 (s, 1 H), 7.09 (d, J = 8.8 Hz, 2 H), 6.95 (s, 1 H), 4.56 (s, 2 H), 3.88 (s, 3 H), 3.75 (s, 3 H), 2.34 (s, 3 H) ppm; ^{13}C NMR (100 MHz, $CDCl_3$) δ = 167.3 (s), 148.4 (s), 147.9 (s), 144.9 (s), 143.7 (s), 139.3 (s), 134.0 (s), 133.6 (s), 132.9 (s), 132.1 (s), 131.6 (d), 130.5 (d), 129.8 (d), 128.7 (d), 128.52 (d), 128.49 (d), 128.4 (d), 127.4 (s), 101.6 (d), 96.6 (d), 56.4 (q), 56.3 (q), 53.9 (t), 21.7 (q) ppm; IR (ATR): $\tilde{\nu}$ = 3064, 2921, 1718, 1623, 1537, 1481, 1334, 1299, 1164, 1084, 902, 867, 814, 767, 699, 623 cm^{-1}; HRMS (ESI) m/z calcd for $[M+Na]^+$: 598.1174; found: 598.1178.

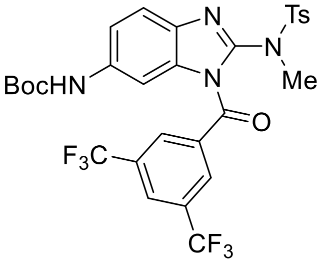

3n, tert-butyl (1-(3,5-bis(trifluoromethyl)benzoyl)-2-(N,4-dimethylphenylsulfonamido)-1H-benzo[d]imidazol-6-yl)carbamate

Yield 85 %, colourless soild, mp: 188-189 °C; ^{1}H NMR (400 MHz, CD_2Cl_2) δ = 8.10-8.08 (m, 4 H), 7.46 (d, *J* = 8.4 Hz, 1 H), 7.29-7.16 (m, 5 H), 6.75 (s, 1 H), 2.83 (s, 3 H), 2.32 (s, 3 H), 1.44 (s, 9 H) ppm; ^{13}C NMR (100 MHz, CD_2Cl_2) δ = 165.7 (s), 152.7 (s), 145.6 (s), 145.5 (s), 137.1 (s), 136.6 (s), 136.2 (s), 133.5 (s), 131.6 (*J* = 33 Hz, s), 131.0 (s), 130.1 (d), 129.8 (d), 128.4 (d), 126.1 (*J* = 4.1 Hz, d), 122.9 (*J* = 271 Hz, s), 120.5 (d), 116.8 (d), 103.9 (d), 80.8 (s), 36.7 (q), 28.1 (q), 21.4 (q) ppm; IR (ATR): $\tilde{\nu}$ = 3020, 2925, 1736, 1698, 1633, 1504, 1289, 1365, 1169, 1032, 1014, 848, 779, 740, 673 cm^{-1}; HRMS (ESI) m/z calcd for $[M+Na]^+$: 679.1420; found: 679.1431.

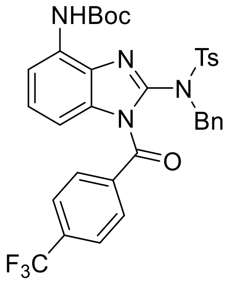

3o, tert-butyl (2-(N-benzyl-4-methylphenylsulfonamido)-1-(4-(trifluoromethyl)benzoyl)-1H-benzo[d]imidazol-4-yl)carbamate

Yield 66 %, colourless soild, mp: 174-175 °C; ^{1}H NMR (400 MHz, CD_2Cl_2) δ = 7.92-7.84 (m, 1 H), 7.45-7.34 (m, 7 H), 7.28-7.10 (m, 8 H), 6.86-6.80 (m, 1 H), 4.63 (s, 2 H), 2.36 (s, 3 H), 1.47 (s, 9 H) ppm; ^{13}C NMR (100 MHz, CD_2Cl_2) δ = 166.8 (s), 152.4 (s), 145.4 (s), 144.3 (s), 136.6 (s), 134.5 (s), 134.0 (*J* = 33 Hz, s), 133.4 (s), 132.8 (s), 130.5 (s), 130.4 (d), 130.0 (d), 129.9 (d), 129.8 (s), 128.5 (d), 128.4 (d), 128.3 (d), 125.7 (d), 125.4 (*J* = 3.5 Hz, d), 123.5 (*J* = 271 Hz, s), 111.4 (d), 106.9 (d), 80.8 (s), 53.7 (t), 28.1 (q), 21.4 (q) ppm; IR (ATR): $\tilde{\nu}$ = , 2363, 1718, 1617, 1597, 1484, 1397, 1352, 1187, 1164, 1124, 1091, 990, 901, 827, 770, 732, 698, 622 cm^{-1}; HRMS (ESI) m/z calcd for $[M+Na]^+$: 687.1859; found: 687.1860.

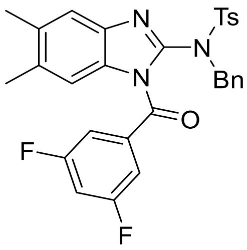

3p, N-benzyl-N-(1-(3,5-difluorobenzoyl)-5,6-dimethyl-1H-benzo[d]imidazol-2-yl)-4-methylbenzenesulfonamide

Yield 53 %, colourless soild, mp: 194-196 °C; ^{1}H NMR (400 MHz, CD_2Cl_2) δ = 7.42 (d, *J* = 8.4 Hz, 2 H), 7.35 (s, 1 H), 7.23 (d, *J* = 8.0 Hz, 2 H), 7.21-7.16 (m, 2 H), 7.15-7.10 (m, 3 H), 7.05-6.97 (m, 1 H), 6.96-6.88 (m, 3 H), 4.54 (s, 2 H), 2.36 (s, 3 H), 2.26 (s, 3 H), 2.19 (s, 3 H) ppm; ^{13}C NMR (100 MHz, CD_2Cl_2) δ = 165.7 (s), 162.5 (*J* = 249.2 Hz, s), 162.4 (*J* = 249.3 Hz, s), 145.3 (s), 144.7 (s), 138.6 (s), 136.9 (*J* = 8.8 Hz, s), 134.5 (s), 133.9 (s), 133.6 (s), 132.9 (s), 131.8 (s), 129.8 (d), 129.7 (d), 128.42 (d), 128.41 (d), 128.2 (d), 120.0 (d), 113.3 (*J* = 21.5 Hz, d), 113.2 (*J* = 19.7 Hz, d), 108.5 (*J* = 25.2 Hz, d), 54.1 (t), 21.4 (q), 20.3 (q), 19.9 (q) ppm; IR (ATR): $\tilde{\nu}$ = 2957, 2931, 1714, 1596, 1439, 1321, 1164, 991, 804, 671 cm^{-1}; HRMS (ESI) m/z calcd for $[M+Na]^+$: 568.1477; found: 568.1475.

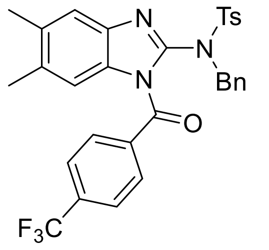

3q, N-benzyl-N-(5,6-dimethyl-1-(4-(trifluoromethyl)benzoyl)-1H-benzo[d]imidazol-2-yl)-4-methylbenzenesulfonamide

Yield 56 %, colourless soild, mp: 178-179 ºC; ^{1}H NMR (400 MHz, $CDCl_3$) δ = 7.43 (s, 1 H), 7.38-7.31 (m, 6 H), 7.26 (s, 1 H), 7.23-7.11 (m, 7 H), 4.57 (s, 2 H), 2.34 (s, 3 H), 2.31 (s, 3 H), 2.26 (s, 3 H) ppm; ^{13}C NMR (100 MHz, CD_2Cl_2) δ = 165.2 (s), 143.1 (s), 142.9 (s), 136.7 (s), 135.2 (s), 132.6 (s), 132.0 (s), 131.9 (*J* = 33 Hz, s), 131.7 (s), 130.7 (s), 130.2 (s), 128.6 (d), 128.3 (d), 128.0 (d), 126.7 (d), 126.6 (d), 126.5 (d), 123.5 (*J* = 3.9 Hz, d), 121.8 (*J* = 271 Hz, s), 118.1 (d), 111.9 (d), 51.8 (t), 19.8 (q), 18.6 (q), 18.4 (q) ppm; IR (ATR): $\tilde{\nu}$ = 2947, 1717, 1597, 1475, 1367, 1287, 1181, 1053, 827, 728, 699 cm^{-1}; HRMS (ESI) m/z calcd for $[M+Na]^+$: 600.1539; found: 600.1539.

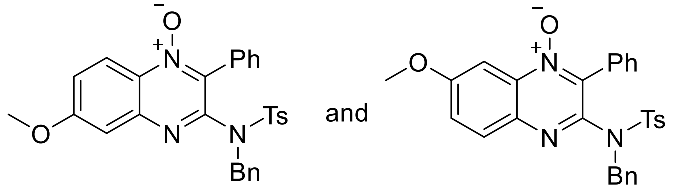

4a, total yield 90 % (C7: C6 = 6:1), colourless soild; ^{1}H NMR (500 MHz, CD_2Cl_2) δ = 8.30-8.23 (m, 0.84 H), 7.85-7.71 (m, 0.26 H), 7.42-7.33 (m, 3 H), 7.32-7.25 (m, 3 H), 7.25-7.17 (m, 3 H), 7.15-7.07 (m, 1 H), 7.07-6.98 (m, 2 H), 6.94-6.80 (m, 2 H), 6.78-6.68 (m, 2 H), 4.45 (s, 1.67 H), 4.43 (s, 0.3 H), 3.92 (s, 2.56 H), 3.89 (s, 0.42 H), 2.36 (m, 3 H) ppm; ^{13}C NMR (125 MHz, $CDCl_3$) δ = 162.4 (s), 149.6 (s), 144.5 (s), 143.9 (s), 140.9 (s), 135.2 (s), 134.2 (s), 132.3 (s), 130.8 (d), 129.9 (d), 129.5 (s), 129.4 (d), 129 (d), 128.9 (d), 128.4 (d), 128.2 (d), 127.8 (d), 123.0 (d), 120.5 (d), 107.4 (d), 56.2 (q), 53.6 (t), 21.4 (q) ppm; IR (ATR): $\tilde{\nu}$ = 2956, 2830, 1621, 1584, 1492, 1463, 1426, 1366, 1338, 1309, 1263, 1210, 1154, 1101, 998, 949, 871, 852, 763, 751, 688, 646, 630 cm^{-1}; HRMS (ESI) m/z calcd for $[M+H]^+$: 512.1639; found: 512.1640.

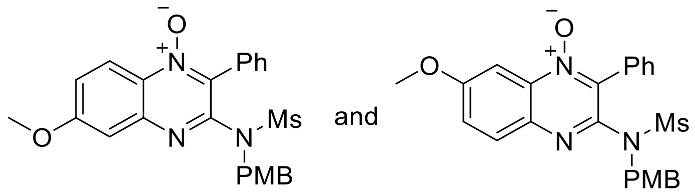

4b, total yield 97 % (C7: C6 = 7:1), colourless soild; ^{1}H NMR (500 MHz, $CDCl_3$) δ = 8.38-8.33 (m, 0.89 H), 7.89-7.85 (m, 0.12 H), 7.80-7.78 (m, 0.10 H), 7.40-7.21 (m, 7 H), 6.91-6.82 (m, 2 H), 6.77-6.59 (m, 2 H), 4.40 (s, 2 H), 3.95-3.87 (m, 3 H), 3.68 (s, 3 H), 2.93-2.83 (m, 3 H) ppm; ^{13}C NMR (125 MHz, $CDCl_3$) δ = 162.4 (s), 159.6 (s), 150.6 (s), 143.9 (s), 139.2 (s), 132.0 (s), 130.8 (d), 130.6 (d), 129.6 (d), 128.6 (s), 128.3 (d), 126.3 (s), 123.1 (d), 120.8 (d), 114.0 (d), 107.4 (d), 56.1 (q), 55.3 (q), 53.7 (t), 41.4 (q) ppm; IR (ATR): $\tilde{\nu}$ = 2966, 2930, 1611, 1581, 1514, 1470, 1414, 1380, 1350, 1336, 1316, 1257, 1216, 1178, 1147, 1105, 1047, 1024, 956, 866, 850, 828, 771, 754, 738, 720, 699, 659, 634, 620 cm^{-1}; HRMS (ESI) m/z calcd for $[M+H]^+$: 466.1431; found: 466.1430.

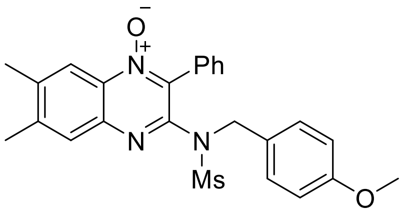

4c, 3-(N-(4-methoxybenzyl)methylsulfonamido)-6,7-dimethyl-2-phenylquinoxaline 1-oxide

Yield 90 %, colourless soild, mp: 191-192 °C; ^{1}H NMR (500 MHz, $CDCl_3$) δ = 8.21 (s, 1 H), 7.73 (s, 1 H), 7.40-7.33 (m, 3 H), 7.32-7.25 (m, 2 H), 6.85 (d, *J* = 8.5 Hz, 2 H), 6.62 (d, *J* = 8.5 Hz, 2 H), 4.38 (s, 2 H), 3.67 (s, 3 H), 2.91 (s, 3 H), 2.43 (s, 6 H) ppm; ^{13}C NMR (125 MHz, $CDCl_3$) δ = 159.5 (s), 149.3 (s), 142.7 (s), 141.8 (s), 140.7 (s), 140.3 (s), 135.1 (s), 130.8 (d), 130.5 (d), 129.5 (d), 128.8 (d), 128.7 (s), 128.2 (d), 126.3 (s), 118.6 (d), 114.0 (d), 55.3 (q), 53.8 (t), 41.1 (q), 20.6 (q), 20.2 (q) ppm; IR (ATR): $\tilde{\nu}$ = 2994, 2919, 2835, 1698, 1613, 1570, 1513, 1484, 1462, 1423, 1399, 1353, 1337, 1320, 1267, 1248, 1231, 1150, 1097, 1075, 1026, 1011, 976, 952, 937, 888, 865, 854, 827, 776, 767, 755, 744, 691, 678, 666, 651, 622 cm^{-1}; HRMS (ESI) m/z calcd for $[M+H]^+$: 464.1639; found: 464.1640.

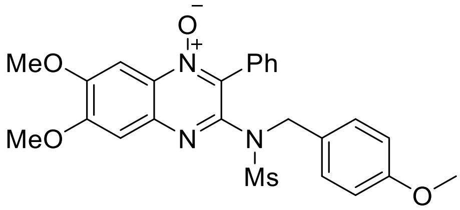

4d, 6,7-dimethoxy-3-(N-(4-methoxybenzyl)methylsulfonamido)-2-phenylquinoxaline 1-oxide

Yield 86 %, colourless soild, mp: 198-200 °C; ^{1}H NMR (500 MHz, $CDCl_3$) δ = 7.79 (s, 1 H), 7.39-7.33 (m, 3 H), 7.30-7.22 (m, 3 H), 6.85 (d, *J* = 9.0 Hz, 2 H), 6.63 (d, *J* = 8.5 Hz, 2 H), 4.40 (s, 2 H), 4.02 (s, 3 H), 3.98 (s, 3 H), 3.68 (s, 3 H), 2.90 (s, 3 H) ppm; ^{13}C NMR (125 MHz, $CDCl_3$) δ = 159.5 (s), 154.2 (s), 153.7 (s), 148.0 (s), 139.7 (s), 139.0 (s), 132.6 (s), 130.8 (d), 130.5 (d), 129.4 (d), 128.8 (s), 128.2 (d), 126.3 (s), 114.0 (d), 107.3 (d), 98.0 (d), 56.8 (q), 56.7 (q), 55.3 (q), 53.7 (t), 41.0 (q) ppm; IR (ATR): $\tilde{\nu}$ = 2942, 2836, 1716, 1613, 1581, 1499, 1456, 1434, 1387, 1322, 1285, 1237, 1212, 1174, 1150, 1112, 1069, 1028, 1001, 955, 903, 831, 771, 748, 701, 669, 654, 612 cm^{-1}; HRMS (ESI) m/z calcd for $[M+H]^+$: 496.1537; found: 496.1536.

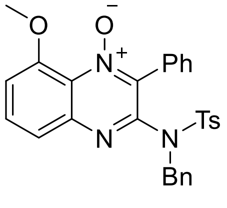

4e, 3-(N-benzyl-4-methylphenylsulfonamido)-8-methoxy-2-phenylquinoxaline 1-oxide

Yield 55 %, colourless soild, mp: 198-199 °C;; ^{1}H NMR (500 MHz, $CDCl_3$) δ = 7.64-7.56 (m, 1 H), 7.54-7.48 (m, 1 H), 7.45-7.39 (m, 2 H), 7.39-7.33 (m, 1 H), 7.32-7.25 (m, 2 H), 7.21-7.16 (m, 2 H), 7.13-7.08 (m, 1 H), 7.04-6.98 (m, 3 H), 6.98-6.90 (m, 2 H), 6.74-6.67 (m, 2 H), 4.44 (s, 2 H), 3.89 (s, 3 H), 2.39 (s, 3 H) ppm; ^{13}C NMR (125 MHz, $CDCl_3$) δ = 153.5 (s), 149.3 (s), 144.7 (s), 144.2 (s), 144.1 (s), 135.5 (s), 134.1 (s), 131.3 (d), 130.7 (d), 129.8 (d), 129.6 (s), 129.3 (d), 129.04 (d), 129.02 (d), 128.9 (s), 128.4 (d), 128.1 (d), 127.9 (d), 121.7 (d), 110.8 (d), 57.1 (q), 53.4 (t), 21.7 (q) ppm; IR (ATR): $\tilde{\nu}$ = 3062, 2930, 1716, 1595, 1500, 1455, 1364, 1334, 1266, 1236, 1168, 1087, 1035, 1023, 972, 944, 869, 843, 813, 770, 737, 697, 665 cm^{-1}; HRMS (ESI) m/z calcd for $[M+H]^+$: 512.1639; found: 512.1636.

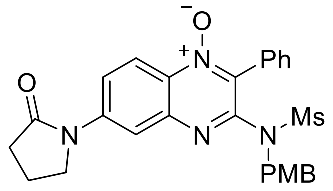

and

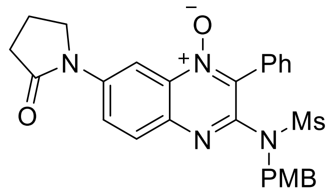

4f, total yield 96 % (C7: C6 = 5:1), colourless soild; ^{1}H NMR (500 MHz, $CDCl_3$) δ = 8.85-8.80 (m, 0.17 H), 8.45-8.40 (m, 0.84 H), 8.26-8.21 (m, 0.85 H), 8.09-8.03 (m, 1 H), 7.99-7.94 (m, 0.18 H), 7.42-7.24 (m, 5 H), 6.91-6.82 (m, 2 H), 6.67-6.60 (m, 2 H),4.43-4.35 (m, 2 H), 4.00-3.88 (m, 2 H), 3.67 (s, 3 H), 2.95-2.85 (m, 3 H), 2.68-2.59 (m, 2 H), 2.24-2.12 (m, 2 H) ppm; ^{13}C NMR (125 MHz, $CDCl_3$) δ = 175.0 (s), 159.7 (s), 150.9 (s), 149.0 (s), 142.6 (s), 142.4 (s), 142.2 (s), 139.9 (s), 138.7 (s), 136.8 (s), 133.2 (s), 130.85 (d), 130.81 (d), 130.5 (d), 130.4 (d), 130.0 (d), 129.7 (d), 128.5 (s), 128.4 (s), 128.3 (d), 128.2 (d), 126.2 (s), 126.1 (s), 125.3 (d), 123.1 (d), 120.1 (d), 116.7 (d), 114.1 (d), 114.0 (d), 105.9 (d), 55.3 (q), 53.8 (t), 53.7 (t), 48.8 (t), 48.7 (t), 41.5 (q), 41.1 (q), 33.0 (t), 32.9 (t), 17.9 (t), 17.7 (t) ppm; IR (ATR): $\tilde{\nu}$ = 2960, 1703, 1612, 1578, 1513, 1471, 1379, 1332, 1276, 1248, 1214, 1178, 1150, 1123, 1028, 954, 868, 825, 779, 756, 731, 699, 669, 625 cm^{-1}; HRMS (ESI) m/z calcd for $[M+H]^+$: 519.1697; found: 519.1699.

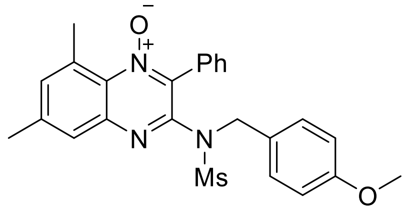

4g, 3-(N-(4-methoxybenzyl)methylsulfonamido)-6,8-dimethyl-2-phenylquinoxaline 1-oxide

Yield 76 %, colourless soild, mp: 194-195 °C; ^{1}H NMR (500 MHz, $CDCl_3$) δ = 7.58 (s, 1 H), 7.38-7.32 (m, 3 H), 7.26-7.16 (m, 3 H), 6.89 (d, J = 8.5 Hz, 2 H), 6.66 (d, J = 8.5 Hz, 2 H), 4.40 (s, 2 H), 3.68 (s, 3 H), 2.91 (s, 3 H), 2.85 (s, 3 H), 2.43 (s, 3 H) ppm; ^{13}C NMR (125 MHz, $CDCl_3$) δ = 159.5 (s), 149.6 (s), 144.0 (s), 141.7 (s), 141.1 (s), 135.4 (d), 134.8 (s), 133.1 (s), 130.8 (d), 130.4 (d), 129.4 (d), 129.0 (s), 128.4 (d), 127.1 (d), 126.4 (s), 114.0 (d), 55.3 (q), 53.6 (t), 41.1 (q), 24.2 (q), 21.3 (q) ppm; IR (ATR): $\tilde{\nu}$ = 2932, 2838, 1714, 1612, 1581, 1513, 1461, 1342, 1284, 1248, 1152, 1113, 1075, 1031, 947, 926, 839, 765, 699, 656, 640, 618 cm^{-1}; HRMS (ESI) m/z calcd for $[M+H]^+$: 464.1639; found: 464.1639.

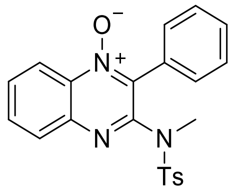

4h, 3-(N,4-dimethylphenylsulfonamido)-2-phenylquinoxaline 1-oxide

Yield 80 %, colourless soild, mp: 195-196 °C; ^{1}H NMR (500 MHz, $CDCl_3$) δ = 8.64-8.58 (m, 1 H), 8.01-7.95 (m, 1 H), 7.87-7.82 (m, 1 H), 7.82-7.56 (m, 1 H), 7.74-7.67 (m, 4 H), 7.63-7.53 (m, 3 H), 7.35-7.30 (m, 2 H), 2.94 (s, 3 H), 2.49 (s, 3 H) ppm; ^{13}C NMR (125 MHz, $CDCl_3$) δ = 151.1 (s), 144.1 (s), 142.0 (s), 140.8 (s), 136.8 (s), 135.1 (s), 131.8 (d), 130.6 (d), 130.4 (d), 129.8 (d), 129.5 (d), 129.4 (d), 129.0 (s), 128.9 (d), 128.6 (d), 119.5 (d), 36.9 (q), 21.7 (q) ppm; IR (ATR): $\tilde{\nu}$ = 3064, 2957, 1717, 1597, 1575, 1537, 1497, 1481, 1453, 1397, 1344, 1297, 1237, 1162, 1117, 1084, 1032, 1018, 995, 902, 867, 814, 767, 728, 699, 666, 618 cm^{-1}; HRMS (ESI) m/z calcd for $[M+H]^+$: 406.1220; found: 406.1223.

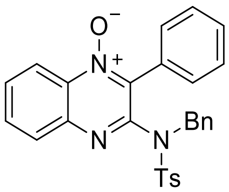

4i, 3-(N-benzyl-4-methylphenylsulfonamido)-2-phenylquinoxaline 1-oxide

Yield 83 %, colourless soild, mp: 194-195 °C; ^{1}H NMR (500 MHz, $CDCl_3$) δ = 8.47-8.42 (m, 1 H), 7.98-7.94 (m, 1 H), 7.79-7.72 (m, 1 H), 7.70-7.64 (m, 1 H), 7.45-7.35 (m, 3 H), 7.35-7.29 (m, 2 H), 7.22-7.15 (m, 2 H), 7.14-7.07 (m, 1 H), 7.05-6.93 (m, 4 H), 6.78-6.67 (m, 2 H), 4.48 (s, 2 H), 2.39 (s, 3 H) ppm; ^{13}C NMR (125 MHz, $CDCl_3$) δ = 149.6 (s), 144.3 (s), 142.7 (s), 141.9 (s), 136.8 (s), 135.3 (s), 134.0 (s), 131.8 (d), 130.7 (d), 130.6 (d), 129.8 (d), 129.5 (d), 129.4 (d), 129.0 (d), 128.9 (s), 128.5 (d), 128.2 (d), 128.1 (d), 119.6 (d), 53.7 (t), 21.7 (q) ppm; IR (ATR): $\tilde{\nu}$ = 3067, 2931, 1911, 1599, 1578, 1530, 1495, 1479, 1454, 1402, 1375, 1344, 1300, 1225, 1184, 1152, 1117, 1088, 1049, 1019, 993, 963, 915, 892, 866, 810, 769, 722, 700, 677, 663, 653, 630, 617 cm^{-1}; HRMS (ESI) m/z calcd for $[M+H]^+$: 482.1533; found: 482.1532.

A gram-scale synthesis procedures: a round bottom flask equipped with a magnetic stirrer bar was added 5 mol% $PtCl_2$ (65 mg), 5 mol% **L3** (230 mg), **1** (10 mmol, 1.2 g), **2** (5 mmol, 1.8 g) and toluene (10 ml). The reaction was heated at 70 °C for 24 h. After cooling to room temperature, the solvent was reduced in vacuo, and the residue was purified by column chromatography (SiO_2, hexanes/EtOAc = 5/1) to offer 1.93 g **4i**.

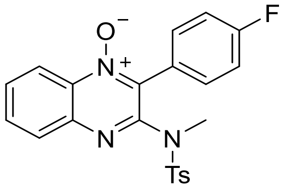

4j, 3-(N,4-dimethylphenylsulfonamido)-2-(4-fluorophenyl)quinoxaline 1-oxide

Yield 78 %, colourless soild, mp: 185-186 °C; ^{1}H NMR (500 MHz, $CDCl_3$) δ = 8.52-8.47 (m, 1 H), 7.87-7.84 (m, 1 H), 7.77-7.72 (m, 1 H), 7.71-7.66 (m, 1 H), 7.66-7.59 (m, 4 H), 7.27-7.22 (m, 2 H), 7.22-7.15 (m, 2 H), 2.86 (s, 3 H), 2.40 (s, 3 H) ppm; ^{13}C NMR (125 MHz, $CDCl_3$) δ = 163.4 (J = 249 Hz, s), 151.1 (s), 144.2 (s), 142.0 (s), 140.0 (s), 136.9 (s), 134.9 (s), 132.6 (J = 8.7 Hz, d), 131.9 (d), 130.7 (d), 129.5 (d), 129.4 (d), 129.0 (d), 124.8 (J = 3.8 Hz, s), 119.5 (d), 115.8 (J = 21.8 Hz, d), 36.9 (q), 21.7 (q) ppm; IR (ATR): $\tilde{\nu}$ = 2926, 1600, 1577, 1535, 1510, 1483, 1451, 1428, 1395, 1345, 1295, 1231, 1186, 1161, 1117, 1084, 1019, 1003, 903, 874, 834, 812, 771, 732, 706, 666, 643, 616 cm^{-1}; HRMS (ESI) m/z calcd for $[M+H]^+$: 424.1126; found: 424.1125.

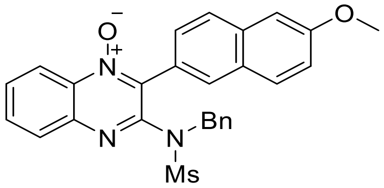

4k, 3-(N-benzylmethylsulfonamido)-2-(6-methoxynaphthalen-2-yl)quinoxaline 1-oxide

Yield 97 %, colourless soild, mp: 191-193 °C; ^{1}H NMR (500 MHz, $CDCl_3$) δ = 8.50-8.45 (m, 1 H), 8.01-7.98 (m, 1 H), 7.79-7.71 (m, 2 H), 7.71-7.64 (m, 1 H), 7.64-7.58 (m, 2 H), 7.48-7.41 (m, 1 H), 7.19-7.13 (m, 1 H), 7.12-7.04 (m, 4 H), 6.96-6.90 (m, 2 H), 4.47 (s, 2 H), 3.87 (s, 3 H), 2.88 (s, 3 H) ppm; ^{13}C NMR (125 MHz, $CDCl_3$) δ = 158.9 (s), 150.5 (s), 141.8 (s), 141.0 (s), 136.9 (s), 135.1 (s), 134.4 (s), 131.9 (d), 130.6 (d), 130.4 (d), 130.3 (d), 129.6 (d),

129.5 (d), 128.7 (d), 128.4 (d), 128.3 (s), 127.8 (d), 126.8 (d), 123.4 (s), 119.5 (d), 119.1 (d), 105.8 (d), 55.4 (q), 54.1 (t), 41.3 (q) ppm; IR (ATR): $\tilde{\nu}$ = 3034, 2929, 1738, 1632, 1650, 1573, 1492, 1479, 1451, 1411, 1342, 1287, 1272, 1209, 1155, 1140, 1110, 1060, 1022, 950, 919, 895, 856, 835, 810, 772, 758, 740, 721, 697, 680, 668, 644, 628 cm^{-1}; HRMS (ESI) m/z calcd for $[M+H]^+$: 486.1482; found: 486.1480.

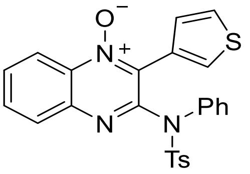

4l, 3-(4-methyl-N-phenylphenylsulfonamido)-2-(thiophen-3-yl)quinoxaline 1-oxide

Yield 74 %, yellow soild, mp: 190-192 °C; ^1H NMR (500 MHz, $CDCl_3$) δ = 8.47-8.42 (m, 1 H), 7.90-7.85 (m, 1 H), 7.82-7.78 (m, 1 H), 7.76-7.70 (m, 1 H), 7.68-7.61 (m, 3 H), 7.33-7.29 (m, 1 H), 7.28-7.24 (m, 1 H), 7.23-7.18 (m, 2 H), 7.10-7.05 (m, 1 H), 7.02-6.96 (m, 2 H), 6.79-6.72 (m, 2 H), 2.38 (s, 3 H) ppm; ^{13}C NMR (125 MHz, $CDCl_3$) δ = 150.9 (s), 144.0 (s), 141.3 (s), 138.2 (s), 136.7 (s), 136.6 (s), 136.2 (s), 131.7 (d), 130.4 (d), 129.8 (d), 129.4 (d), 128.9 (d), 128.8 (d), 128.7 (d), 128.5 (d), 128.1 (d), 127.4 (s), 125.0 (d), 119.4 (d), 21.7 (q) ppm; IR (ATR): $\tilde{\nu}$ = 3104, 2959, 2925, 1734, 1596, 1578, 1522, 1484, 1451, 1428, 1346, 1308, 1280, 1237, 1211, 1186, 1165, 1138, 1111, 1089, 1056, 1017, 949, 908, 846, 811, 767, 717, 694, 680, 661, 634, 619, 610 cm^{-1}; HRMS (ESI) m/z calcd for $[M+H]^+$: 474.0941; found: 474.0939.

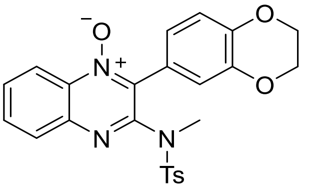

4m, 2-(2,3-dihydrobenzo[b][1,4]dioxin-6-yl)-3-(N,4-dimethylphenylsulfonamido)quinoxaline 1-oxide

Yield 63 %, colourless soild, mp: 208-209 °C; ^1H NMR (500 MHz, $CDCl_3$) δ = 8.51-8.47 (m, 1 H), 7.86-7.82 (m, 1 H), 7.74-7.63 (m, 4 H), 7.27-7.23 (m, 2 H), 7.17-7.10 (m, 2 H), 6.99-6.96 (m, 1 H), 4.31-4.21 (m, 4 H), 2.87 (s, 3 H), 2.39 (s, 3 H) ppm; ^{13}C NMR (125 MHz, $CDCl_3$) δ = 151.2 (s), 145.0 (s), 144.0 (s), 143.6 (s), 141.8 (s), 140.2 (s), 136.9 (s), 135.4 (s), 131.6 (d), 130.4 (d), 129.4 (d), 129.3 (d), 128.9 (d), 123.8 (d), 121.5 (s), 119.6 (d), 119.5 (d), 117.6 (d), 37.0 (q), 21.7 (q) ppm; IR (ATR): $\tilde{\nu}$ = 2975, 2872, 1717, 1599, 1577, 1540, 1508, 1483, 1459, 1448, 1430, 1398, 1367, 1343, 1304, 1281, 1249, 1186, 1158, 1139, 1126, 1116, 1087, 1062, 1003, 937, 915, 885, 836, 810, 767, 748, 720, 705, 687, 663, 618 cm^{-1}; HRMS (ESI) m/z calcd for $[M+H]^+$: 464.1275; found: 464.1271.

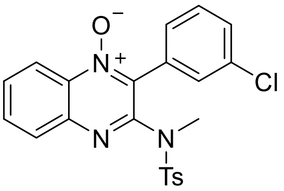

4n, 2-(3-chlorophenyl)-3-(N,4-dimethylphenylsulfonamido)quinoxaline 1-oxide

Yield 59 %, colourless soild, mp: 179-181 °C; ^1H NMR (500 MHz, $CDCl_3$) δ = 8.52-8.48 (m, 1 H), 7.89-7.85 (m, 1 H), 7.78-7.73 (m, 1 H), 7.73-7.67 (m, 1 H), 7.62-7.56 (m, 3 H), 7.55-7.50 (m, 1 H), 7.45-7.40 (m, 2 H), 7.26-7.21 (m, 2 H), 2.89 (s, 3 H), 2.39 (s, 3 H) ppm; ^{13}C NMR (125 MHz, $CDCl_3$) δ = 150.9 (s), 144.2 (s), 142.1 (s), 139.7 (s), 136.9 (s), 134.7 (s), 134.3 (s), 132.1 (d), 130.8 (d), 130.7 (s), 130.4 (d), 130.0 (d), 129.9 (d), 129.6 (d), 129.4 (d),

128.9 (d), 128.8 (d), 119.5 (d), 37.0 (q), 21.7 (q) ppm; IR (ATR): $\tilde{\nu}$ = 3068, 2929, 1724, 1597, 1576, 1534, 1490, 1450, 1397, 1346, 1292, 1263, 1236, 1187, 1161, 1117, 1085, 999, 873, 806, 774, 752, 728, 691, 667, 617 cm^{-1}; HRMS (ESI) m/z calcd for $[M+H]^+$: 440.0830; found: 440.0827.

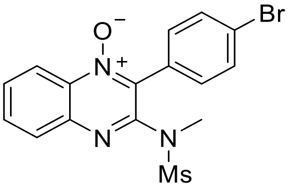

4o, 2-(4-bromophenyl)-3-(N-methylmethylsulfonamido)quinoxaline 1-oxide

Yield 70 %, colourless soild, mp: 210-211 °C; ^{1}H NMR (500 MHz, $CDCl_3$) δ = 8.51-8.46 (m, 1 H), 7.98-7.94 (m, 1 H), 7.80-7.75 (m, 1 H), 7.73-7.68 (m, 1 H), 7.62 (d, *J* = 8.5 Hz, 2 H), 7.51 (d, *J* = 8.5 Hz, 2 H), 3.19 (s, 3 H), 2.91 (s, 3 H) ppm; ^{13}C NMR (125 MHz, $CDCl_3$) δ = 151.0 (s), 142.0 (s), 139.0 (s), 136.9 (s), 132.2 (d), 132.0 (d), 131.9 (d), 130.8 (d), 129.5 (d), 127.3 (s), 124.6 (s), 119.5 (d), 39.3 (q), 37.1 (q) ppm; IR (ATR): $\tilde{\nu}$ = 2930, 1717, 1578, 1537, 1495, 1481, 1454, 1405, 1390, 1338, 1294, 1242, 1200, 1156, 1115, 1085, 1070, 1014, 1002, 963, 904, 871, 822, 798, 766, 748, 728, 707, 665, 651, 620 cm^{-1}; HRMS (ESI) m/z calcd for $[M+H]^+$: 408.0012; found: 408.0010.

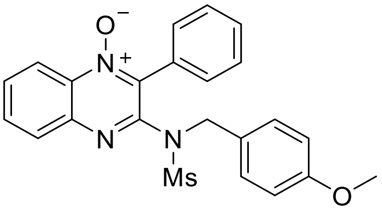

4p, 3-(N-(4-methoxybenzyl)methylsulfonamido)-2-phenylquinoxaline 1-oxide

Yield 95 %, colourless soild, mp: 189-191 °C; ^{1}H NMR (500 MHz, $CDCl_3$) δ = 8.49-8.44 (m, 1 H), 8.01-7.95 (m, 1 H), 7.79-7.73 (m, 1 H), 7.71-7.65 (m, 1 H), 7.42-7.36 (m, 3 H), 7.35-7.28 (m, 2 H), 6.88 (d, *J* = 8.5 Hz, 2 H), 6.64 (d, *J* = 8.5 Hz, 2 H), 4.41 (s, 2 H), 3.67 (s, 3 H), 2.89 (s, 3 H) ppm; ^{13}C NMR (125 MHz, $CDCl_3$) δ = 159.6 (s), 150.4 (s), 141.9 (s), 140.9 (s), 136.8 (s), 131.9 (d), 130.8 (d), 130.6 (d), 130.5 (d), 129.8 (d), 129.6 (d), 128.5 (s), 128.4 (d), 126.2 (s), 119.6 (d), 114.1 (d), 55.3 (q), 53.8 (t), 41.4 (q) ppm; IR (ATR): $\tilde{\nu}$ = 2933, 2837, 1723, 1612, 1576, 1513, 1483, 1454, 1402, 1337, 1247, 1177, 1150, 1112, 1028, 953, 893, 865, 808, 784, 765, 722, 698, 665, 651, 620 cm^{-1}; HRMS (ESI) m/z calcd for $[M+H]^+$: 436.1326; found: 436.1323.

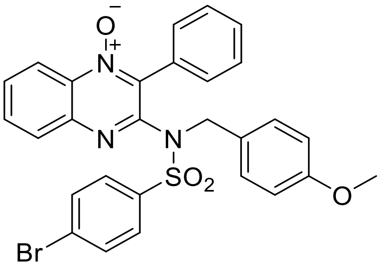

4q, 3-(4-bromo-N-(4-methoxybenzyl)phenylsulfonamido)-2-phenylquinoxaline 1-oxide

Yield 80 %, colourless soild, mp: 194-195 °C; ^{1}H NMR (500 MHz, $CDCl_3$) δ = 8.49-8.45 (m, 1 H), 7.96-7.92 (m, 1 H), 7.81-7.75 (m, 1 H), 7.73-7.67 (m, 1 H), 7.56-7.51 (m, 2 H), 7.45-7.39 (m, 3 H), 7.39-7.33 (m, 2 H), 7.13-7.02 (m, 2 H), 6.66 (d, *J* = 8.5 Hz, 2 H), 6.64 (d, *J* = 9.0 Hz, 2 H), 4.40 (s, 2 H), 3.65 (s, 3 H) ppm; ^{13}C NMR (125 MHz,

CDCl3) δ = 159.6 (s), 149.5 (s), 142.5 (s), 141.9 (s), 137.6 (s), 136.9 (s), 131.95 (d), 131.92 (d), 131.0 (d), 130.8 (d), 130.5 (d), 130.4 (d), 129.5 (d), 129.4 (d), 128.7 (s), 128.4 (s), 128.2 (d), 125.6 (s), 119.7 (d), 113.9 (d), 55.2 (q), 53.6 (t) ppm; IR (ATR): $\tilde{\nu}$ = 3064, 2934, 2836, 1738, 1612, 1573, 1513, 1500, 1482, 1452, 1401, 1390, 1349, 1293, 1279, 1247, 1162, 1087, 1068, 1009, 963, 894, 865, 822, 803, 782, 754, 737, 698, 670, 633 cm^{-1}; HRMS (ESI) m/z calcd for $[M+H]^+$: 576.0587; found: 576.0597.

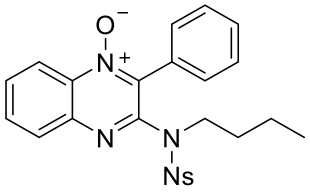

4r, 3-(N-butyl-4-nitrophenylsulfonamido)-2-phenylquinoxaline 1-oxide

Yield 80 %, colourless soild, mp: 192-193 °C; ^{1}H NMR (500 MHz, CDCl3) δ = 8.54-8.50 (m, 1 H), 8.31 (d, *J* = 8.5 Hz, 2 H), 8.04 (d, *J* = 9.0 Hz, 2 H), 7.86-7.82 (m, 1 H), 7.81-7.76 (m, 1 H), 7.75-7.69 (m, 1 H), 7.65-7.60 (m, 2 H), 7.54-7.46 (m, 3 H), 3.20 (t, *J* = 7. 5 Hz, 2 H), 1.11-1.02 (m, 2 H), 0.96-0.86 (m, 2 H), 0.60 (t, *J* = 7.5 Hz, 3 H) ppm; ^{13}C NMR (125 MHz, CDCl3) δ = 150.3 (s), 149.2 (s), 145.1 (s), 141.9 (s), 141.6 (s), 137.1 (s), 132.2 (d), 131.0 (d), 130.4 (d), 130.2 (d), 130.1 (d), 129.4 (d), 128.7 (d), 128.5 (s), 123.8 (d), 119.7 (d), 50.4 (t), 29.7 (t), 19.8 (t), 13.5 (q) ppm; IR (ATR): $\tilde{\nu}$ = 3109, 2959, 2930, 2911, 2872, 2850, 1736, 1606, 1575, 1524, 1499, 1480, 1463, 1446, 1404, 1347, 1312, 1293, 1240, 1227, 1160, 1130, 1110, 1077, 1034, 1024, 978, 949, 884, 859, 778, 769, 746, 734, 719, 700, 683, 666, 636 cm^{-1}; HRMS (ESI) m/z calcd for $[M+H]^+$: 479.1384; found: 479.1381.

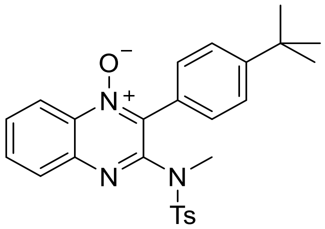

4s, 2-(4-(tert-butyl)phenyl)-3-(N,4-dimethylphenylsulfonamido)quinoxaline 1-oxide

Yield 78 %, colourless soild, mp: 192-193 °C; ^{1}H NMR (500 MHz, CDCl3) δ = 8.52-8.47 (m, 1 H), 7.88-7.84 (m, 1 H), 7.75-7.69 (m, 1 H), 7.69-7.60 (m, 3 H), 7.59-7.54 (m, 2 H), 7.53-7.48 (m, 2 H), 7.25-7.20 (m, 2 H), 2.83 (s, 3 H), 2.38 (s, 3 H), 1.32 (s, 9 H) ppm; ^{13}C NMR (125 MHz, CDCl3) δ = 152.9 (s), 151.2 (s), 144.0 (s), 141.8 (s), 140.7 (s), 136.9 (s), 135.3 (s), 131.6 (d), 130.4 (d), 130.0 (d), 129.4 (d), 129.3 (d), 129.0 (d), 125.7 (s), 125.5 (d), 119.5 (d), 36.9 (q), 34.9 (s), 31.3 (q), 21.7 (q) ppm; IR (ATR): $\tilde{\nu}$ = 2958, 1734, 1599, 1577, 1540, 1509, 1484, 1453, 1393, 1362, 1346, 1300, 1240, 1202, 1187, 1160, 1131, 1108, 1084, 1016, 1003, 896, 875, 831, 810, 788, 773, 758, 730, 717, 704, 675, 660, 635, 624, 615 cm^{-1}; HRMS (ESI) m/z calcd for $[M+H]^+$: 462.1846; found: 462.1846.

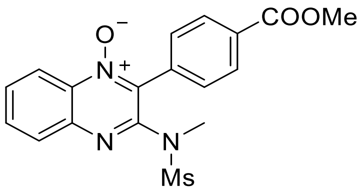

4t, 2-(4-(methoxycarbonyl)phenyl)-3-(N-methylmethylsulfonamido)quinoxaline 1-oxide

Yield 54 %, colourless soild, mp: 208-209 °C; ^{1}H NMR (500 MHz, CDCl3) δ = 8.52-8.47 (m, 1 H), 8.17-8.13 (m, 2 H), 8.01-7.96 (m, 1 H), 7.81-7.77 (m, 1 H), 7.75-7.66 (m, 3 H), 3.89 (s, 3 H), 3.15 (s, 3 H), 2.91 (s, 3 H) ppm; ^{13}C

NMR (125 MHz, $CDCl_3$) δ = 166.5 (s), 150.9 (s), 142.1 (s), 139.2 (s), 136.9 (s), 133.1 (s), 132.3 (d), 131.3 (s), 130.8 (d), 130.5 (d), 129.8 (d), 129.6 (d), 119.5 (d), 52.4 (q), 39.2 (q), 37.1 (q) ppm; IR (ATR): $\tilde{\nu}$ = 2931, 1720, 1611, 1578, 1537, 1511, 1484, 1452, 1435, 1395, 1340, 1276, 1193, 1157, 1110, 1085, 1021, 1005, 963, 907, 878, 853, 825, 799, 767, 747, 723, 701, 666, 651, 620 cm^{-1}; HRMS (ESI) m/z calcd for $[M+H]^+$: 338.0962; found: 338.0959.

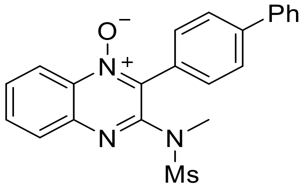

4u, 2-([1,1'-biphenyl]-4-yl)-3-(N-methylmethylsulfonamido)quinoxaline 1-oxide

Yield 65 %, colourless soild, mp: 200-201 °C; ^{1}H NMR (500 MHz, $CDCl_3$) δ = 8.54-8.49 (m, 1 H), 7.99-7.93 (m, 1 H), 7.79-7.74 (m, 1 H), 7.73-7.66 (m, 5 H), 7.62-7.57 (m, 2 H), 7.43-7.37 (m, 2 H), 7.34-7.28 (m, 1 H), 3.18 (s, 3 H), 2.93 (s, 3 H) ppm; ^{13}C NMR (125 MHz, $CDCl_3$) δ = 151.3 (s), 142.7 (s), 141.9 (s), 140.3 (s), 139.6 (s), 137.0 (s), 132.0 (d), 130.7 (d), 130.6 (d), 129.5 (d), 128.9 (d), 127.9 (d), 127.3 (d), 127.27 (d), 127.22 (s), 119.6 (d), 39.6 (q), 37.1 (q) ppm; IR (ATR): $\tilde{\nu}$ = 2926, 1725, 1602, 1579, 1519, 1483, 1450, 1397, 1335, 1299, 1236, 1200, 1157, 1144, 1114, 1084, 1027, 1005, 965, 909, 875, 837, 765, 747, 731, 695, 663, 649, 637, 620 cm^{-1}; HRMS (ESI) m/z calcd for $[M+H]^+$: 406.1220; found: 406.1221.

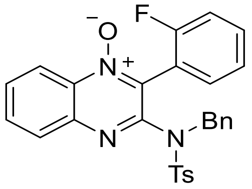

4v, 3-(N-benzyl-4-methylphenylsulfonamido)-2-(2-fluorophenyl)quinoxaline 1-oxide

Yield 70 %, colourless soild, mp: 186-187 °C; ^{1}H NMR (500 MHz, $CDCl_3$) δ = 8.50-8.45 (m, 1 H), 7.94-7.89 (m, 1 H), 7.79-7.73 (m, 1 H), 7.71-7.65 (m, 1 H), 7.52 (d, J = 8.5 Hz, 2 H), 7.45-7.38 (m, 1 H), 7.21 (d, J = 8.0 Hz, 2 H), 7.14-7.07 (m, 2 H), 7.06-6.91 (m, 4 H), 6.87-6.81 (m, 2 H), 4.64 (d, J = 8.5 Hz, 1 H), 4.41 (d, J = 9.0 Hz, 1 H), 2.40 (s, 3 H) ppm; ^{13}C NMR (125 MHz, $CDCl_3$) δ = 161.3 (J = 251 Hz, s), 150.1 (s), 144.3 (s), 142.2 (s), 139.2 (s), 136.7 (s), 135.1 (s), 134.0 (s), 132.1 (d), 131.8 (J = 2.7 Hz, d), 131.7 (J = 8.7 Hz, d), 130.7 (d), 129.6 (d), 129.5 (d), 129.4 (d), 129.0 (d), 128.5 (d), 128.2 (d), 123.8 (J = 3.7 Hz, d), 119.7 (d), 117.5 (J = 15 Hz, s), 115.5 (J = 21.6 Hz, d), 54.0 (q), 21.7 (q) ppm; IR (ATR): $\tilde{\nu}$ = 3065, 2927, 1734, 1618, 1598, 1577, 1533, 1498, 1481, 1455, 1404, 1354, 1304, 1264, 1230, 1186, 1162, 1118, 1089, 1028, 965, 916, 872, 814, 759, 731, 701, 664, 651, 626 cm^{-1}; HRMS (ESI) m/z calcd for $[M+H]^+$: 500.1439; found: 500.1436.

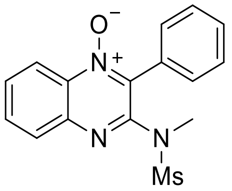

4w, 3-(N-methylmethylsulfonamido)-2-phenylquinoxaline 1-oxide

Yield 70 %, colourless soild, mp: 198-199 °C; ^{1}H NMR (500 MHz, $CDCl_3$) δ = 8.53-8.47 (m, 1 H), 7.99-7.94 (m, 1 H), 7.79-7.73 (m, 1 H), 7.72-7.66 (m, 1 H), 7.63-7.57 (m, 2 H), 7.52-7.40 (m, 3 H), 3.12 (s, 3 H), 2.90 (s, 3 H) ppm; ^{13}C NMR (125 MHz, $CDCl_3$) δ = 151.3 (s), 141.9 (s), 139.8 (s), 136.9 (s), 131.9 (d), 130.6 (d), 130.2 (d), 130.0 (d),

129.5 (d), 128.7 (d), 128.5 (s), 119.6 (d), 39.5 (q), 37.0 (q) ppm; IR (ATR): $\tilde{\nu}$ = 2959, 2903, 2867, 1716, 1600, 1578, 1509, 1484, 1454, 1394, 1362, 1346, 1300, 1240, 1202, 1187, 1161, 1131, 1108, 1084, 1017, 1004, 896, 875, 831, 810, 788, 773, 758, 731, 717, 704, 675, 660, 635, 624, 615 cm^{-1}; HRMS (ESI) m/z calcd for $[M+H]^+$: 330.0907; found: 330.0907.

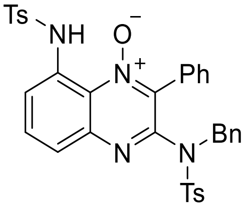

4i-1, 3-(N-benzyl-4-methylphenylsulfonamido)-8-(4-methylphenylsulfonamido)-2-phenylquinoxaline 1-oxide

Yield 77 %, yellow soild, mp: 215-216 °C;; ^{1}H NMR (500 MHz, $CDCl_3$) δ = 12.4 (s, 1 H), 7.80-7.76 (m, 1 H), 7.70-7.65 (m, 2 H), 7.62-7.55 (m, 1 H), 7.55-7.50 (m, 1 H), 7.45-7.40 (m, 1 H), 7.37-7.30 (m, 4 H), 7.20-7.11 (m, 5 H), 7.06-7.00 (m, 2 H), 6.94-6.86 (m, 2 H), 6.73-6.66 (m, 2 H), 4.41 (s, 2 H), 2.38 (s, 3 H), 2.28 (s, 3 H) ppm; ^{13}C NMR (125 MHz, $CDCl_3$) δ = 149.4 (s), 144.6 (s), 144.2 (s), 143.5 (s), 142.9 (s), 136.2 (s), 134.9 (s), 133.7 (s), 133.1 (s), 131.9 (d), 130.4 (d), 129.8 (d), 129.7 (d), 129.4 (d), 128.9 (d), 128.5 (d), 128.3 (d), 128.2 (d), 128.0 (s), 127.4 (d), 126.4 (s), 123.6 (d), 118.2 (d), 53.5 (t), 21.7 (q), 21.6 (q) ppm; IR (ATR): $\tilde{\nu}$ = 1580, 1532, 1496, 1446, 1410, 1345, 1292, 1185, 1162, 1122, 1088, 1023, 957, 889, 855, 811, 756, 726, 698, 659 cm^{-1}; HRMS (ESI) m/z calcd for $[M+Na]^+$: 673.1550; found:673.1563.

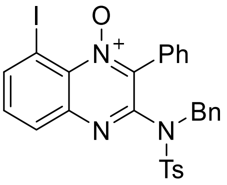

4i-2, 3-(N-benzyl-4-methylphenylsulfonamido)-8-iodo-2-phenylquinoxaline 1-oxide

Yield 46 %, yellow soild, mp: 203-204 °C;; ^{1}H NMR (500 MHz, $CDCl_3$) δ = 8.33-8.27 (m, 1 H), 7.94-7.88 (m, 1 H), 7.42-7.36 (m, 3 H), 7.35-7.28 (m, 3 H), 7.21-7.16 (m, 2 H), 7.15-7.10 (m, 1 H), 7.06-6.92 (m, 4 H), 6.75-6.69 (m, 2 H), 4.45 (s, 2 H), 2.38 (s, 3 H) ppm; ^{13}C NMR (125 MHz, $CDCl_3$) δ = 149.2 (s), 145.4 (d), 144.3 (s), 143.4 (s), 142.5 (s), 135.6 (s), 135.2 (s), 133.9 (s), 132.1 (d), 130.7 (d), 130.6 (d), 129.8 (d), 129.4 (d), 129.3 (d), 128.9 (d), 128.8 (s), 128.5 (d), 128.3 (d), 128.2 (d), 80.6 (s), 53.5 (t), 21.7 (q) ppm; IR (ATR): $\tilde{\nu}$ = 1596, 1548, 1522, 1497, 1475, 1446, 1417, 1387, 1337, 1266, 1185, 1160, 1088, 1025, 968, 907, 862, 804, 772, 756, 722, 696, 664, 629 cm^{-1}; HRMS (ESI) m/z calcd for $[M+Na]^+$: 630.0319; found: 630.0328.

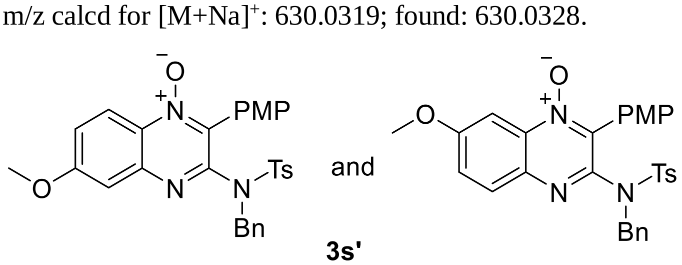

3s'

3s', Total yield 84 % (C7: C6 = 4:1), yellow soild; ^{1}H NMR (400 MHz, $CDCl_3$) δ = 8.51-8.37 (m, 0.84 H), 7.92-7.85 (m, 0.28 H), 7.66-7.53 (m, 2 H), 7.42-7.35 (m, 1 H), 7.35-7.25 (m, 3 H), 7.23-7.16 (m, 1 H), 7.16-7.00 (m, 4 H), 6.99-6.90 (m, 2 H), 6.89-6.80 (m, 2 H), 4.57 (s, 1.6 H), 4.54 (s, 0.4 H), 4.02 (s, 2.4 H), 3.99 (s, 0.6 H), 3.91 (s, 3 H), 2.49 (s, 3 H) ppm; ^{13}C NMR (100 MHz, $CDCl_3$) δ = 162.1 (s), 160.2 (s), 149.9 (s), 144.1 (s), 143.7 (s), 140.9 (s), 135.3 (s), 134.1 (s), 132.2 (d), 129.7 (d), 129.3 (d), 129.0 (d), 128.4 (d), 128.1 (d), 123.0 (d), 121.0 (s), 120.8 (d),

113.5 (d), 107.3 (d), 56.1 (q), 55.3 (q), 53.6 (t), 21.7 (q) ppm; IR (ATR): $\tilde{\nu}$ = 2934, 1613, 1583, 1513, 1495, 1469, 1455, 1407, 1379, 1344, 1315, 1250, 1217, 1163, 1107, 1089, 1026, 829, 742, 701, 670, 666, 548 cm^{-1}; HRMS (ESI) m/z calcd for $[M+H]^+$: 542.1744; found: 542.1735.

2.3 Computational Studies

All the structures were optimized in the gas phase at B3LYP[8]-D3(BJ)[9]/BSI level using GAUSSIAN 16 program,[10] where BSI represents a basis set combination of SDD[11] for metals and 6-31G(d)[12] for all other atoms. Analytic harmonic vibrational frequencies were computed to verify the nature of all optimized stationary points as either minima or transition-state (TS) structures, having zero and one imaginary frequencies, respectively, and to compute thermal contributions to free energies at 298.15 K, 1 atm. All frequencies below 50 cm^{-1} were replaced by 50 cm^{-1} when computing vibrational entropies.[13] IRC calculations were carried out for key transition states to make sure that the TS correctly connects its reactant and product. Improved energies were computed at M06[14]/BSII-SMD single-point calculations. Basis set BSII is a combination of SDD for metals and 6-311++G(d,p)[15] for all other atoms. The experimental used solvents 1,2-DCE (for Pt(II)-system) and toluene (for Au(III)-system) were utilized in the SMD[16] model for solvation calculations. The CM5 atomic partial charges were calculated by Multiwfn program[17] under the level of M06/BSII-SMD.

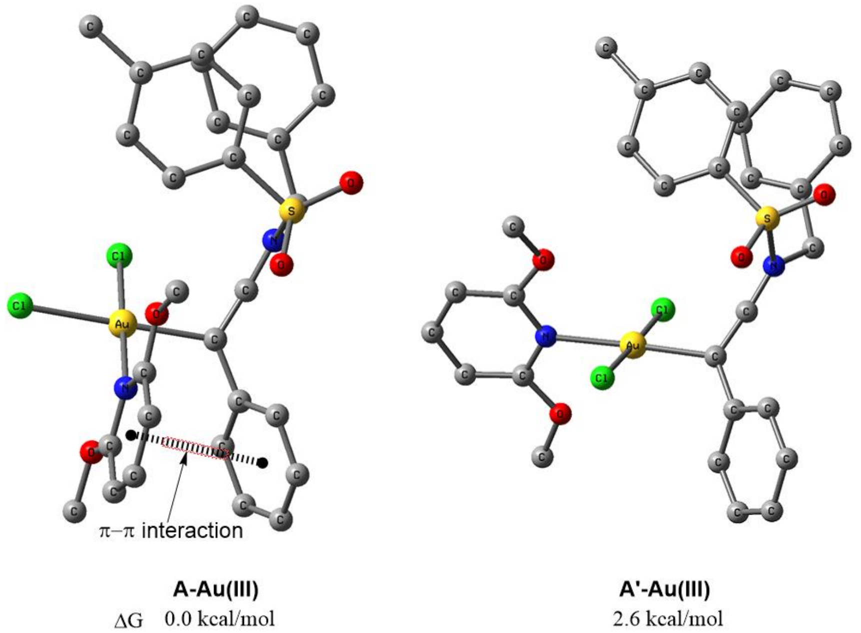

Supplementary Figure 10. Optimized possible geometries of substrate coordination for the Au(III) system. The coordination of substrate **1a** to the position *cis* to the pyridine ligand is more favorable due to the π-π interaction between the ligand and the substrate. Note that the Au(III)-center favors four-coordination, thus one Cl ion has left from the Au(III)-center during substrate coordination, making the complex **A-Au(III)** a cationic species.

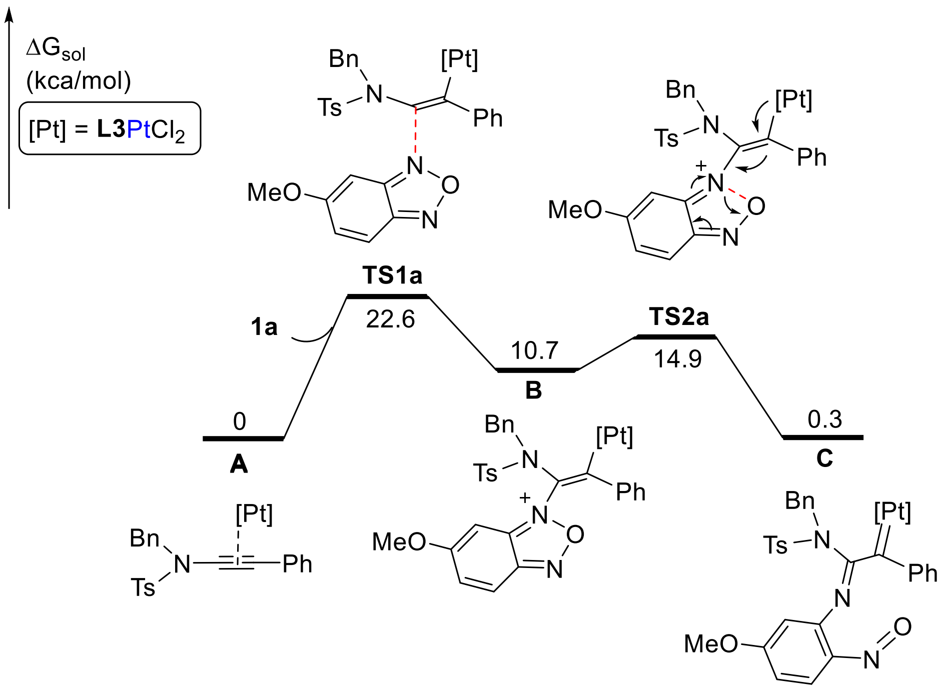

Supplementary Figure 11. Calculated free energy profile of Pt(II)-carbene formation.

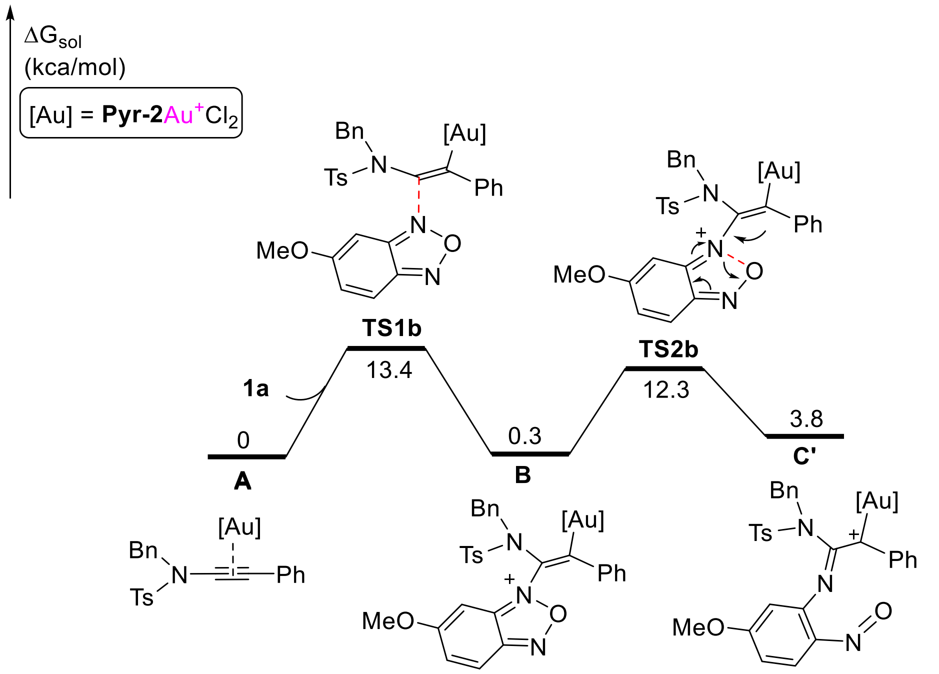

Supplementary Figure 12. Calculated free energy profile of Au(III)-carbene formation.

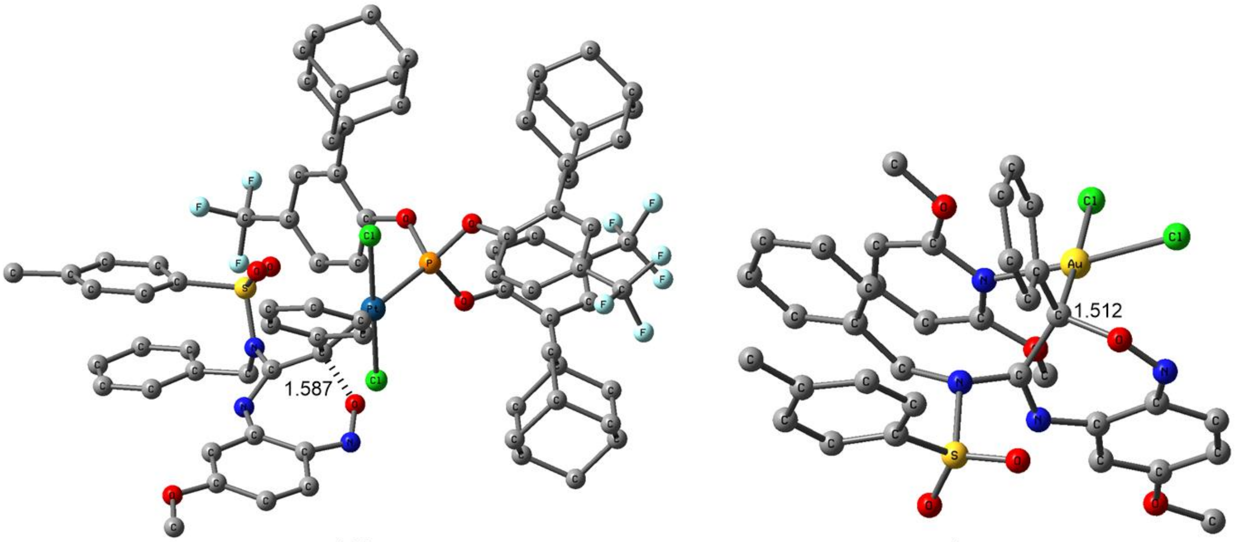

D' **E**

Supplementary Figure 13. Optimized geometries of seven-membered ring complexes resulted from O-attack. Selected bond lengths are given in Å.

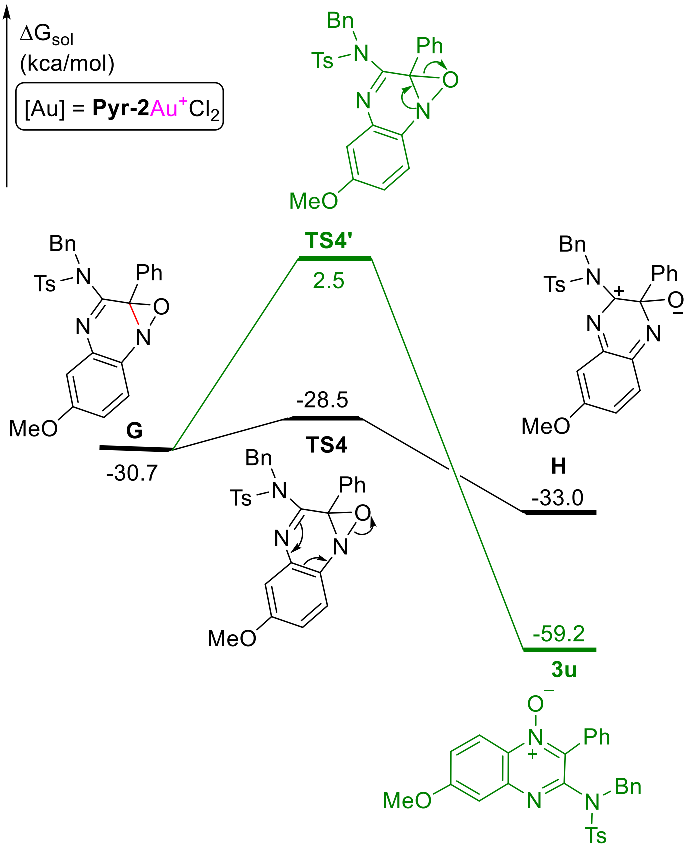

Supplementary Figure 14. Comparison of N-O bond cleavage with C-O bond cleavage.

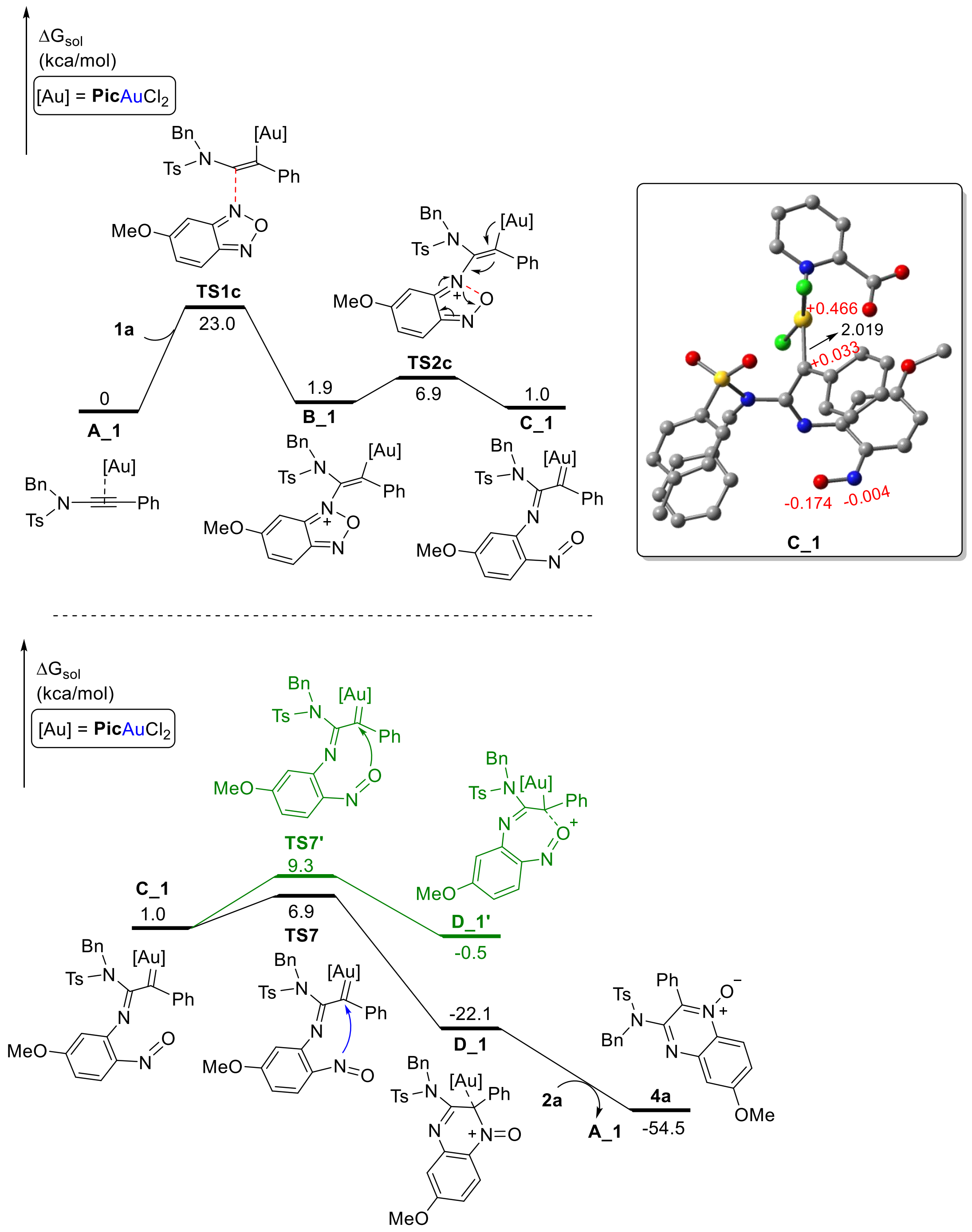

Supplementary Figure 15. The calculated free energy profiles of the $PicAuCl_2$ system. Selected bond lengths (black, Å) and CM5 atomic partial charges (red, au) for **C_1** are provided.

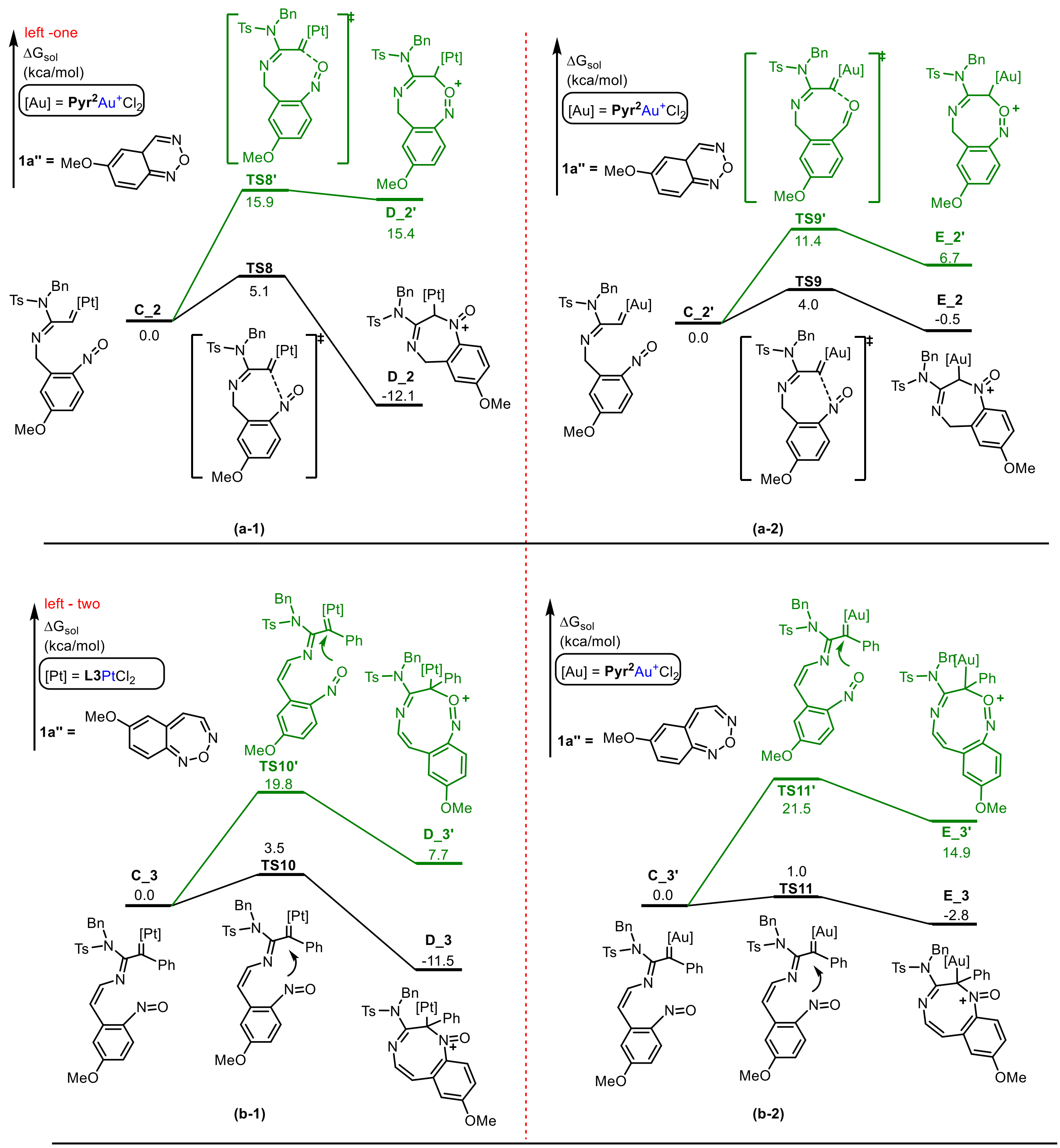

Supplementary Figure 16. The calculated free energy profiles of the ring size effect on the selectivity of annulation.

The phenyl group and imine moiety were tethered by the $-CH_2$ or -CH=CH- linker. (In order to save calculation cost, the adamantane group in the ligand of the Pt systems was simplified to ^{t}Bu except for (**a-1**))

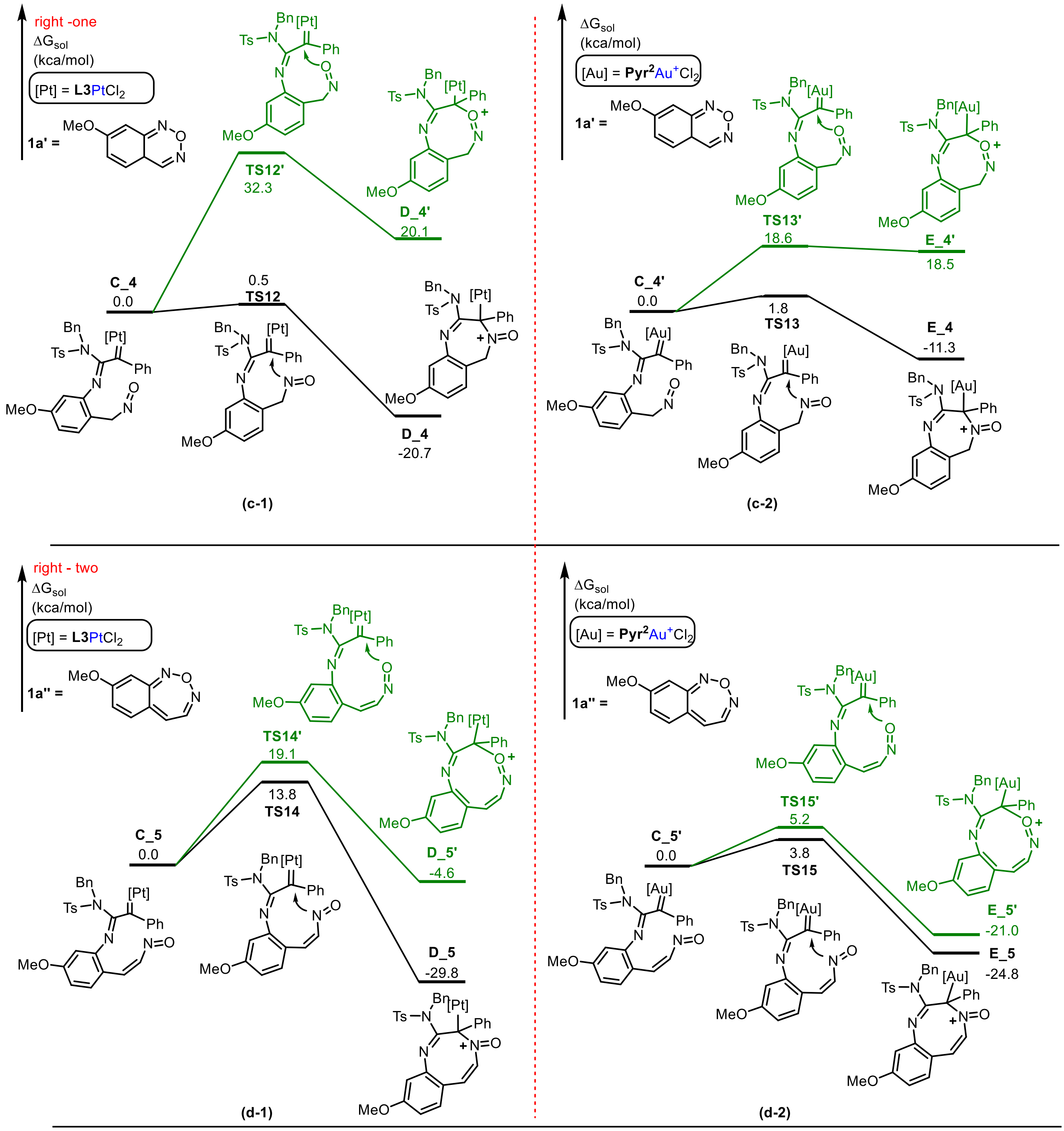

Supplementary Figure 17. The calculated free energy profiles of the ring size effect on the selectivity of annulation.

The phenyl group and nitroso were tethered by the -CH_2 or -CH=CH- linker. (In order to save calculation cost, the adamantane group in the ligand of the Pt systems was simplified to ^{t}Bu)

Supplementary Table 1. Comparison of ring size effect between the Au(III) and the Pt(II) systems.

	[M]	Au			Pt		
	n	N attack TS-N	O-attack TS-O	ΔΔG‡ = (TS-O)-(TS-N)	N attack TS-N	O-attack TS-O	ΔΔG‡ = (TS-O)-(TS-N)
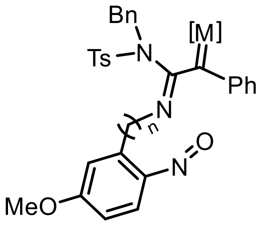	*n* = 1	4	11.4	7.4	5.1	15.9	10.8
	n =2	1	21.5	20.5	3.5	19.8	16.3
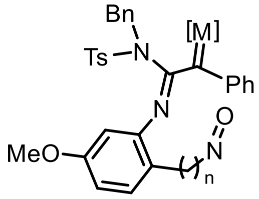	*n* = 1	1.8	18.6	16.8	0.5	32.3	31.8
	n =2	3.8	5.2	1.4	13.8	19.1	5.3

2.4 NMR Spectra

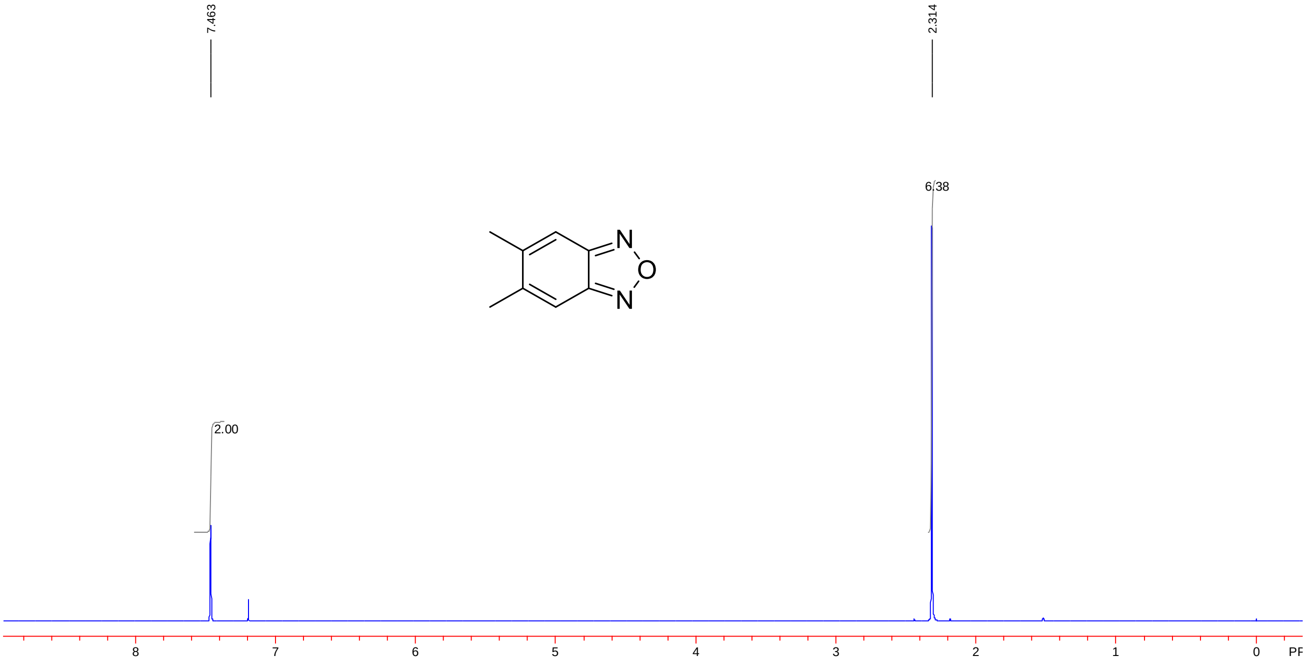

Supplementary Figure 18. ^{1}H NMR of compound **S1a**

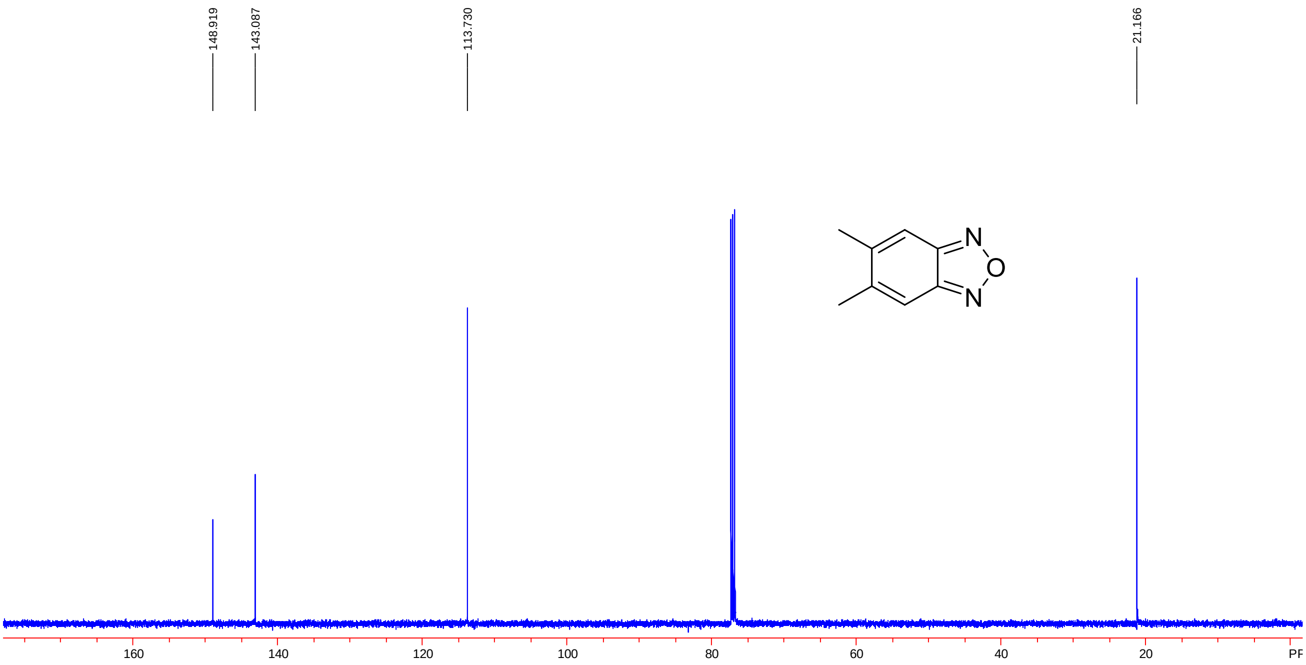

Supplementary Figure 19. ^{13}C NMR of compound **S1a**

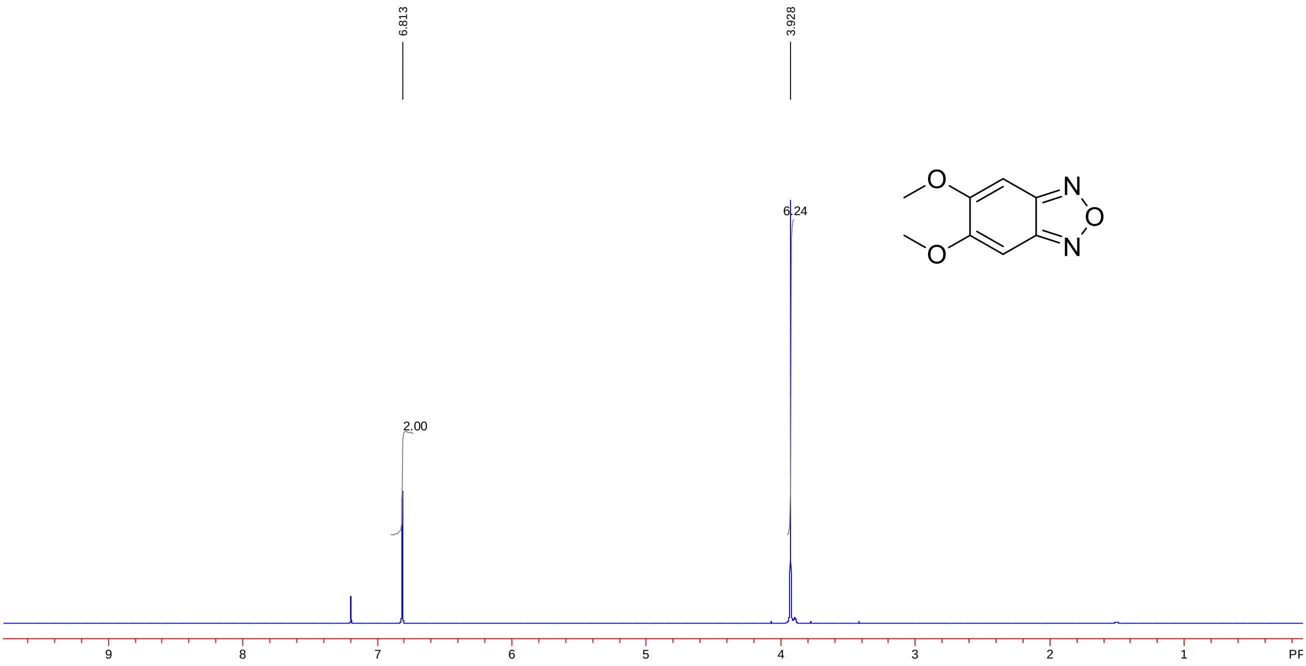

Supplementary Figure 20. ^{1}H NMR of compound **S1b**

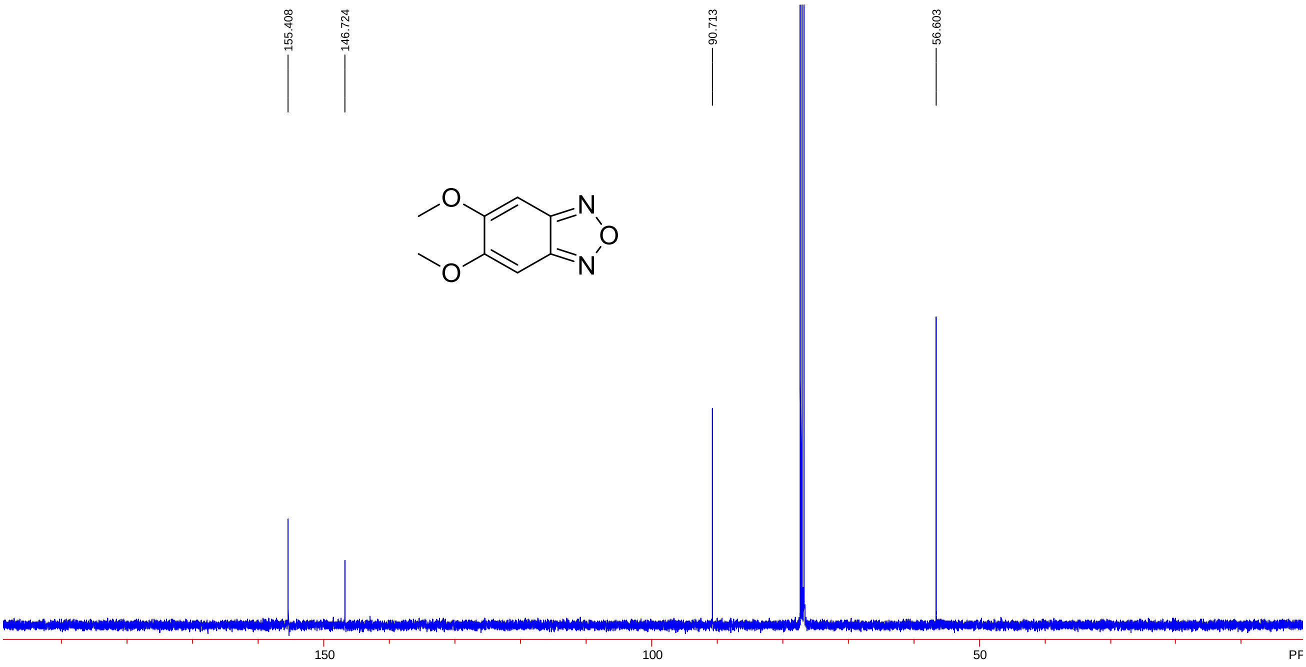

Supplementary Figure 21. ^{13}C NMR of compound **S1b**

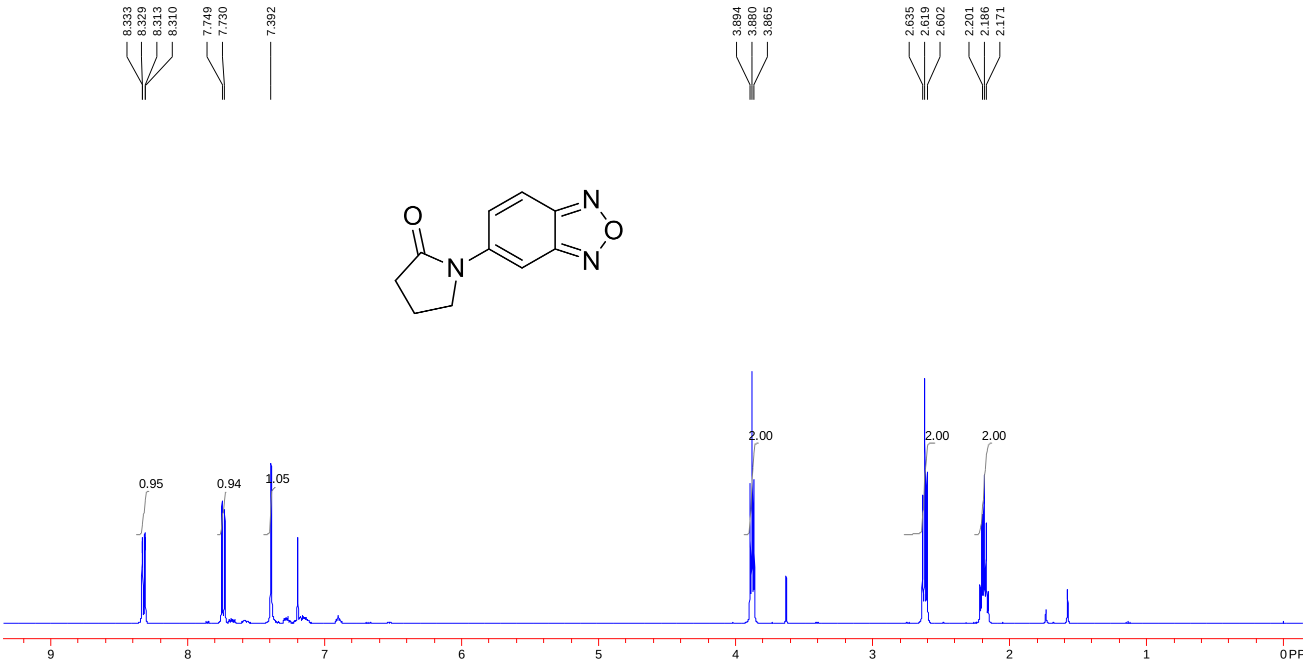

Supplementary Figure 22. ^{1}H NMR of compound **S1c**

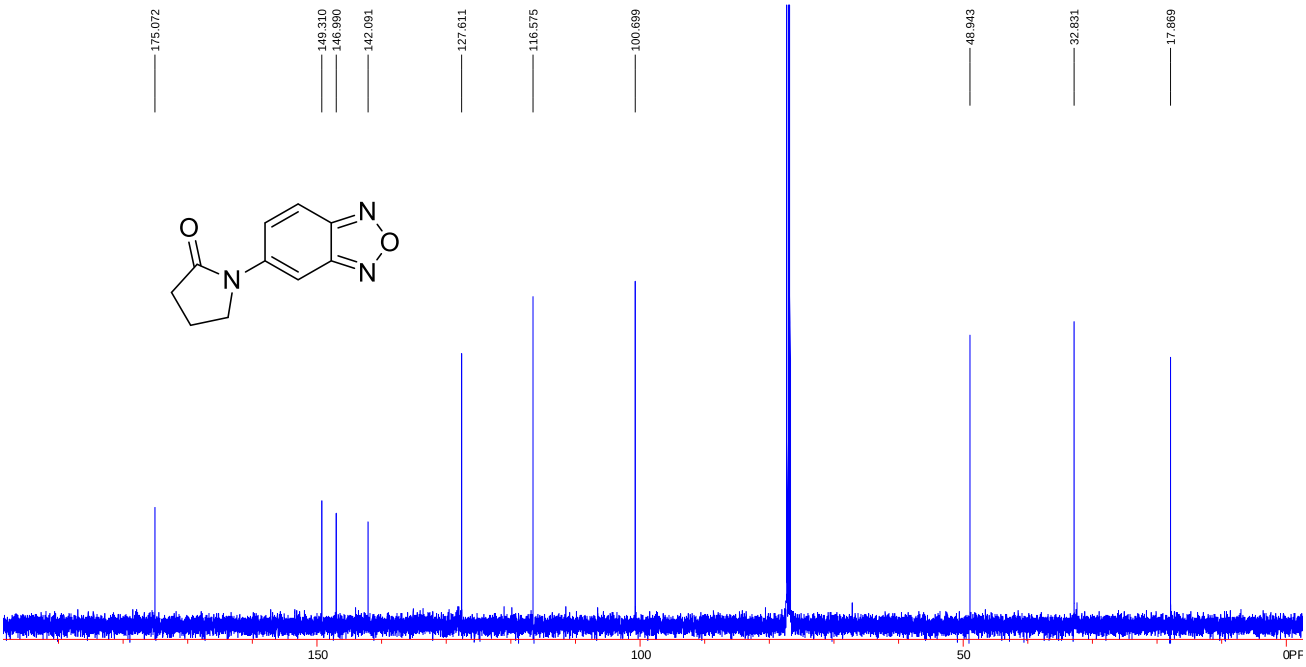

Supplementary Figure 23. ^{13}C NMR of compound **S1c**

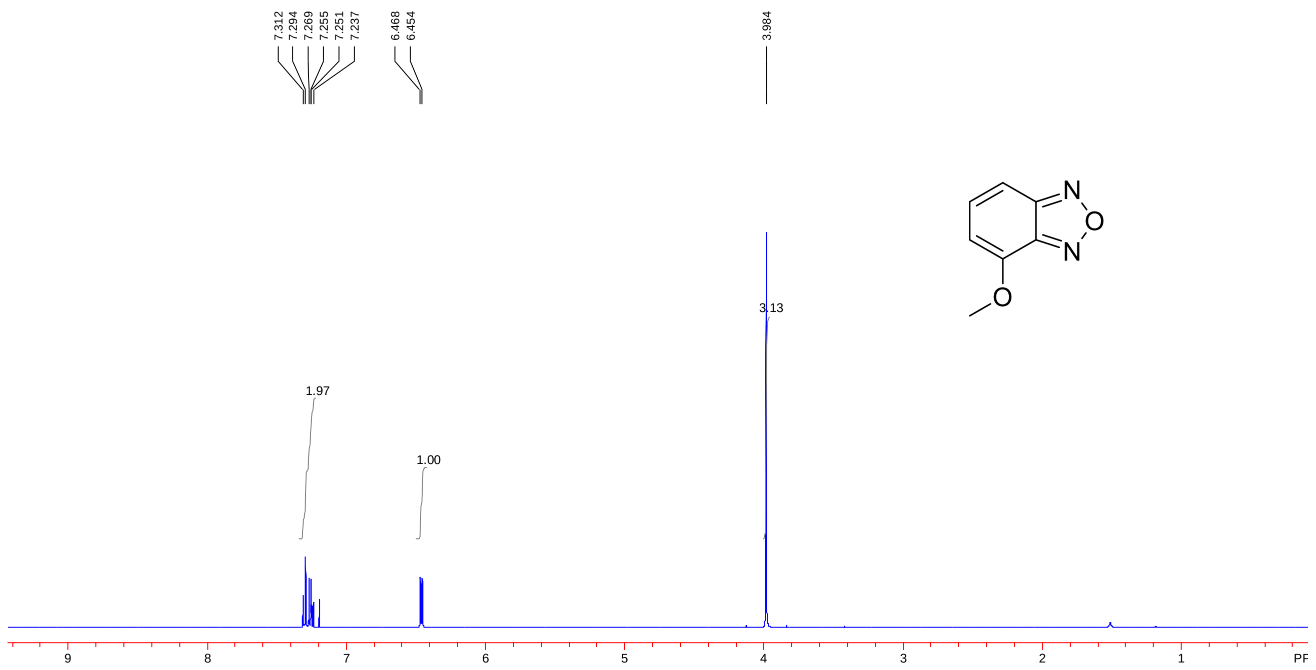

Supplementary Figure 24. ^{1}H NMR of compound **S1d**

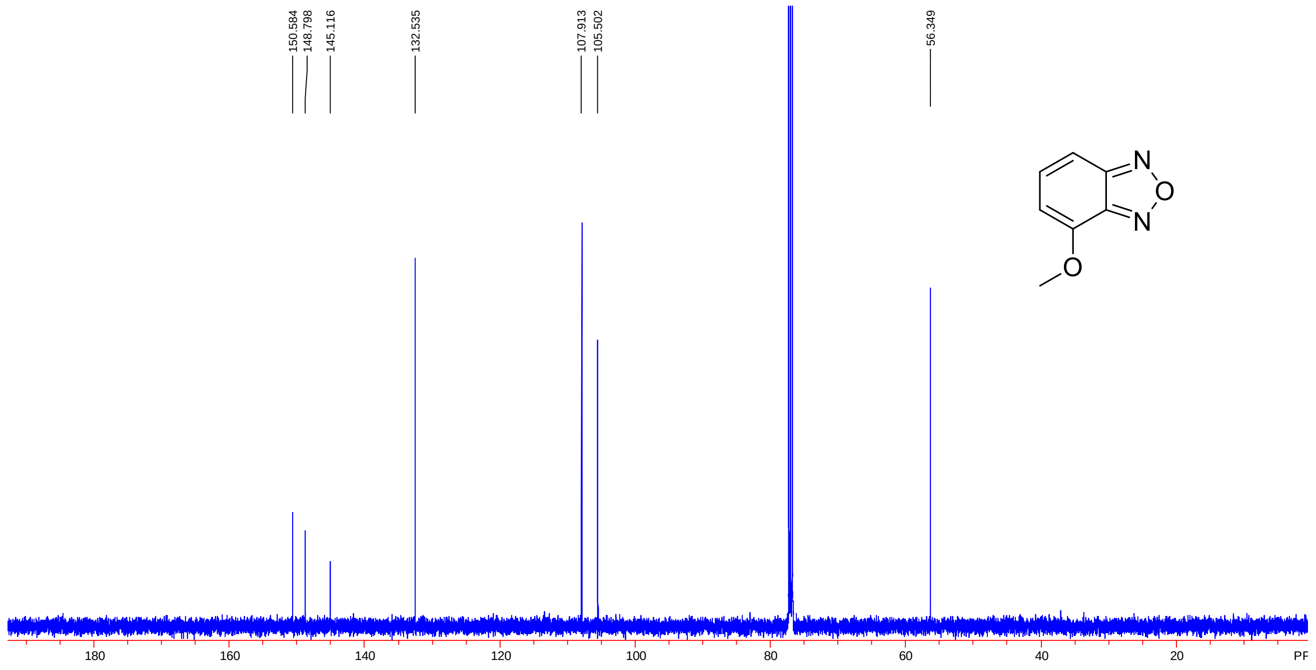

Supplementary Figure 25. ^{13}C NMR of compound **S1d**

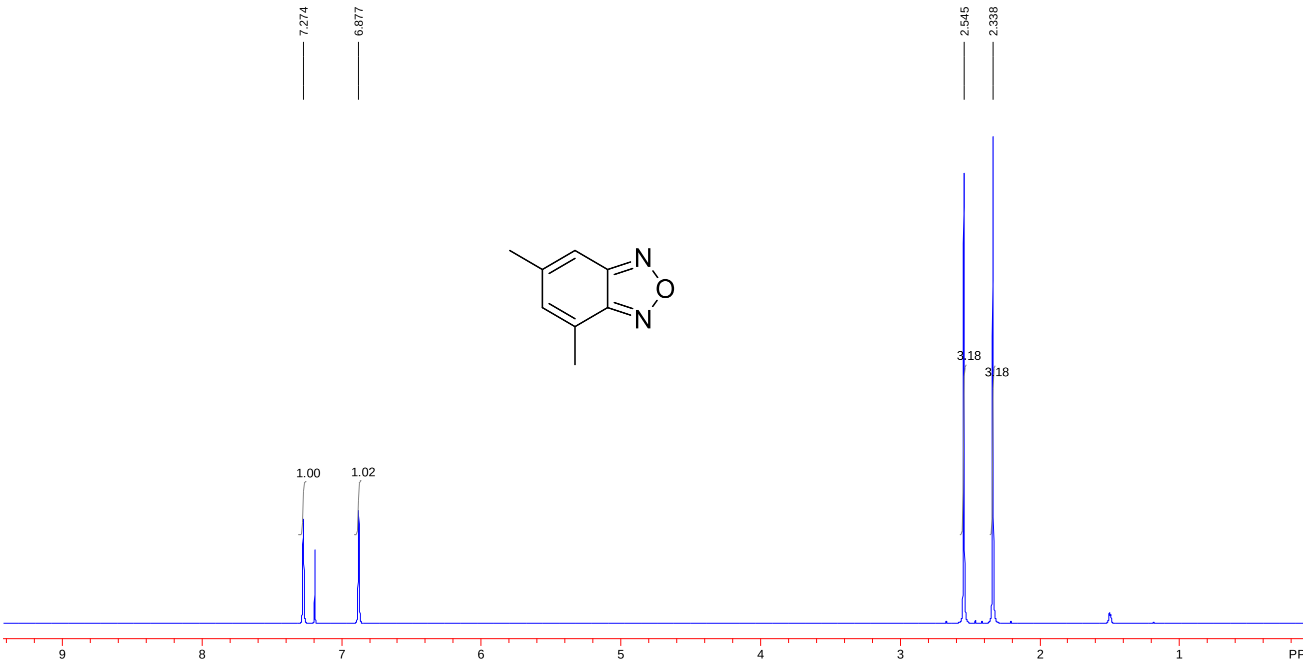

Supplementary Figure 26. ^{1}H NMR of compound **S1e**

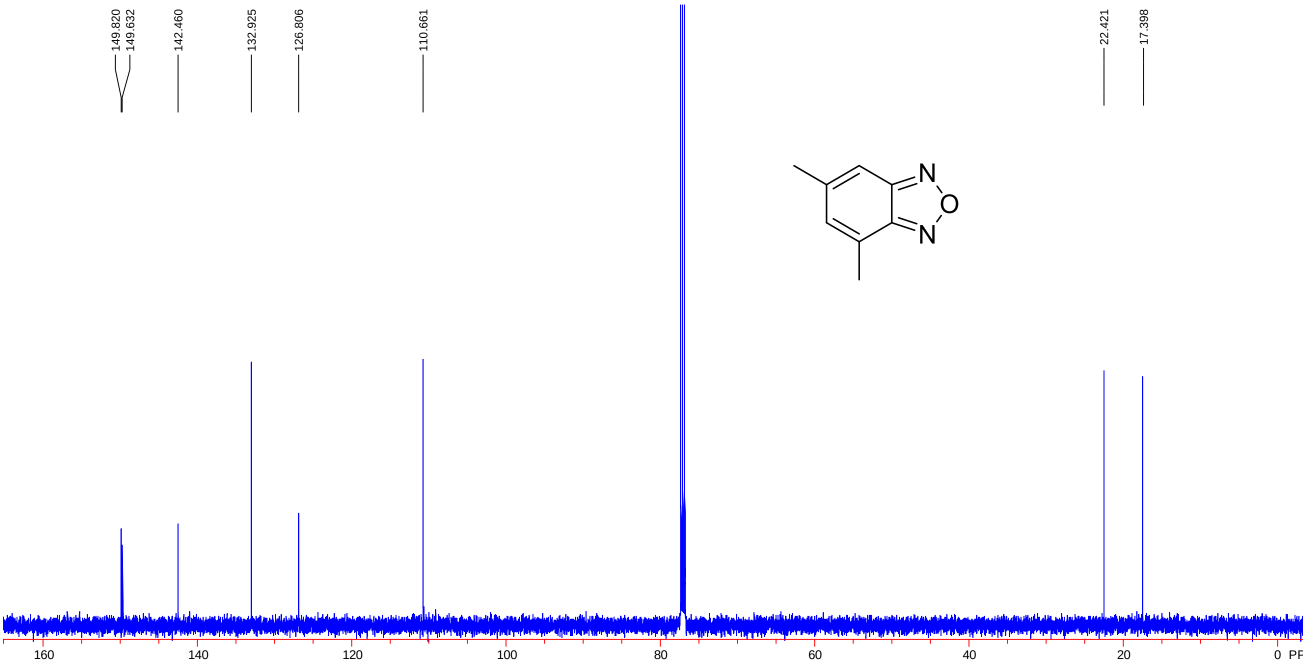

Supplementary Figure 27. ^{13}C NMR of compound **S1e**

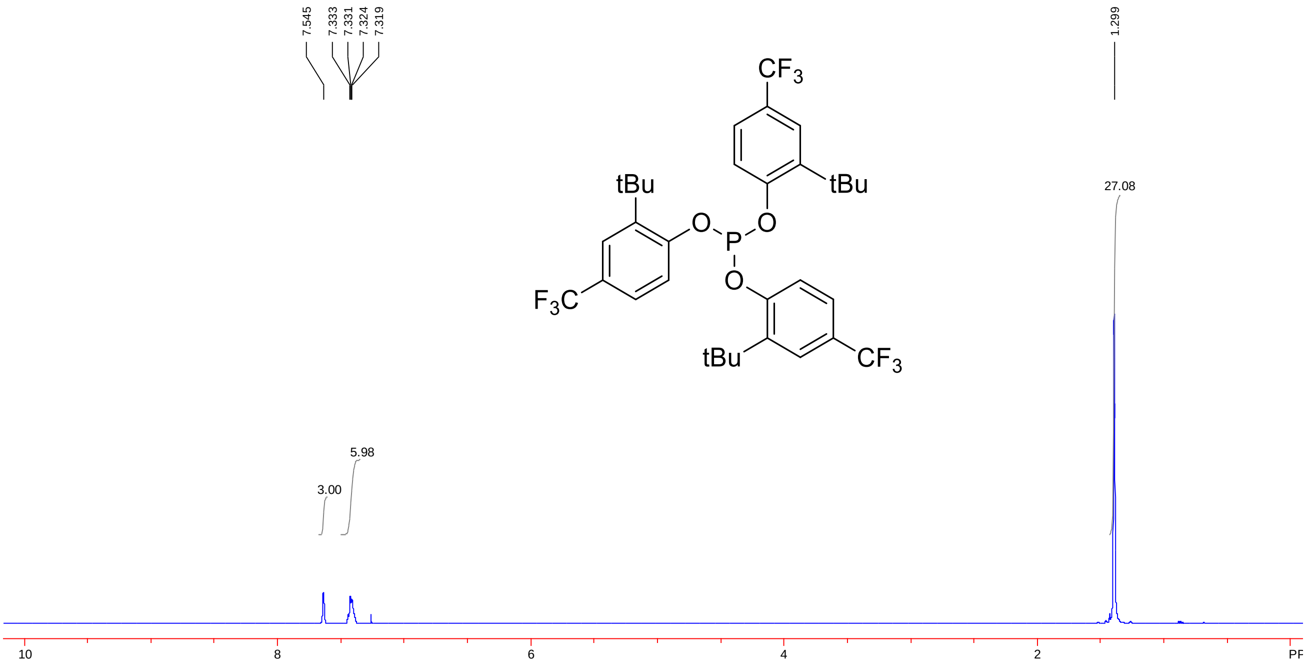

Supplementary Figure 28. ^{1}H NMR of compound **L2**

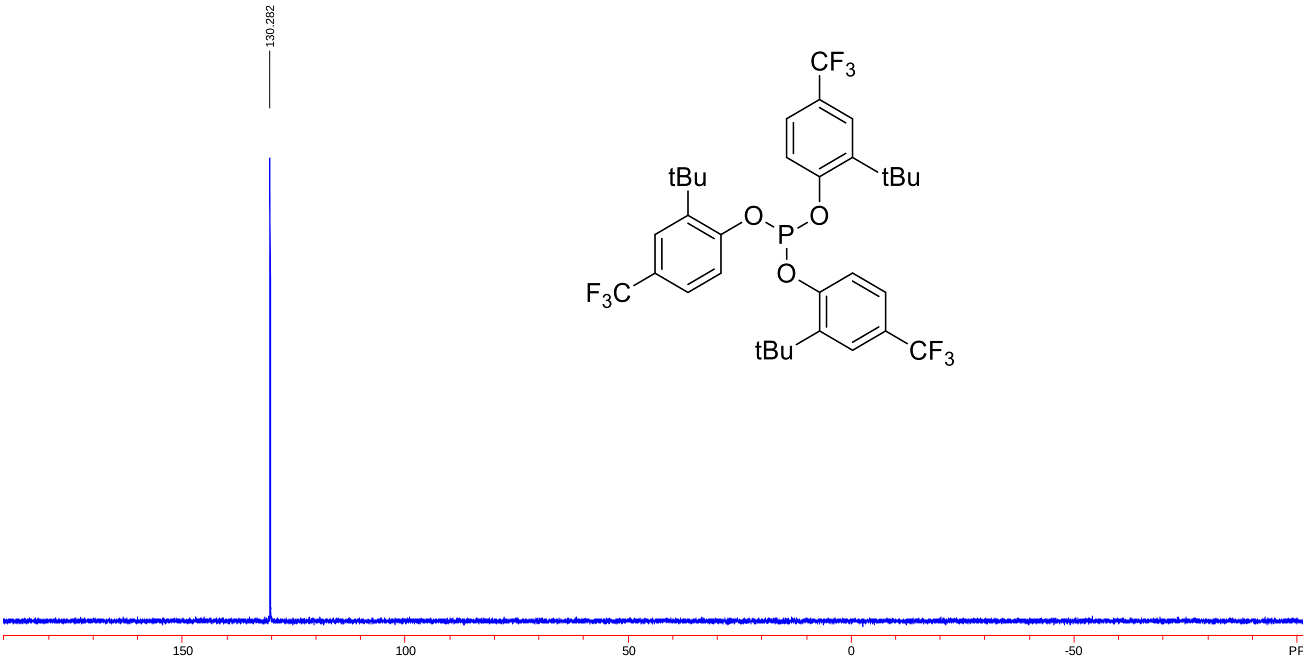

Supplementary Figure 29. ^{13}C NMR of compound **L2**

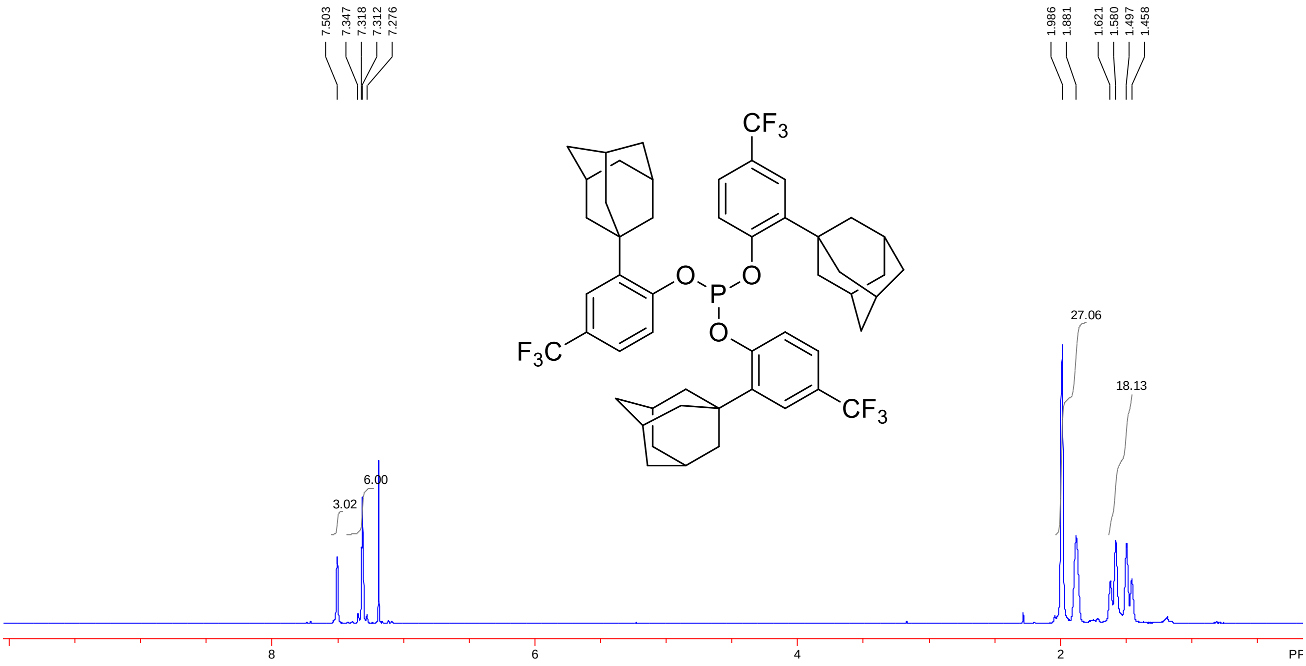

Supplementary Figure 30. ^{1}H NMR of compound **L3**

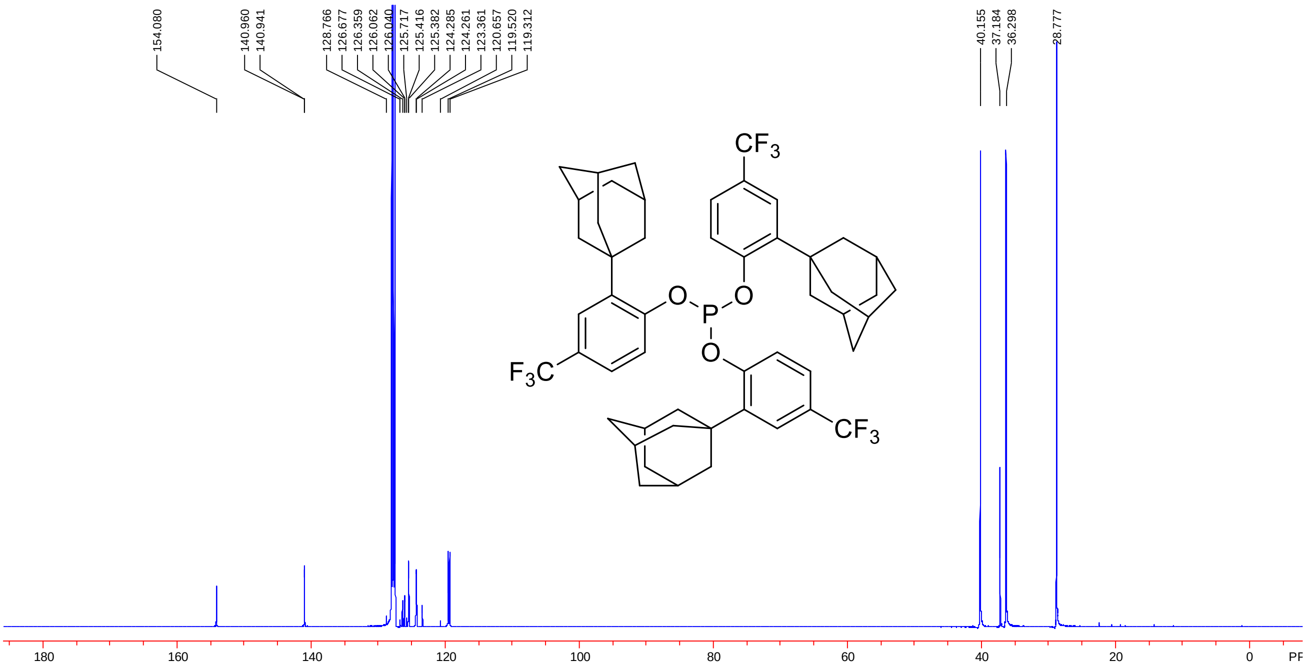

Supplementary Figure 31. ^{13}C NMR of compound **L3**

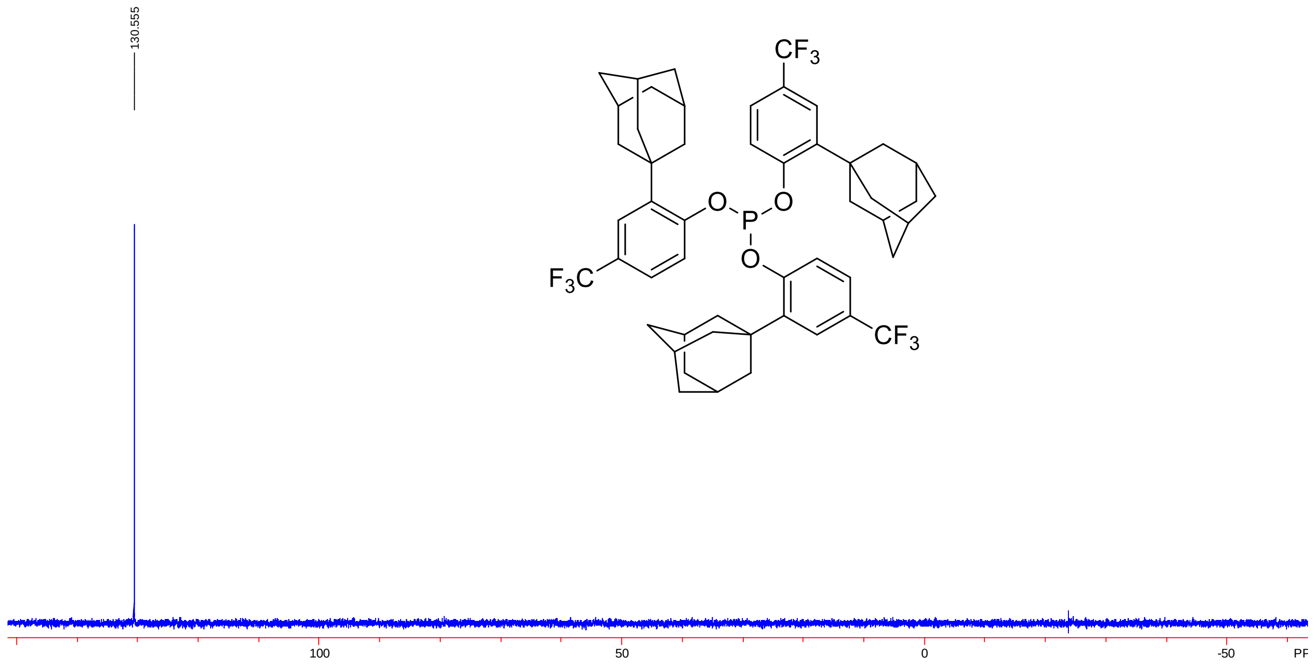

Supplementary Figure 32. ^{31}P NMR of compound **L3**

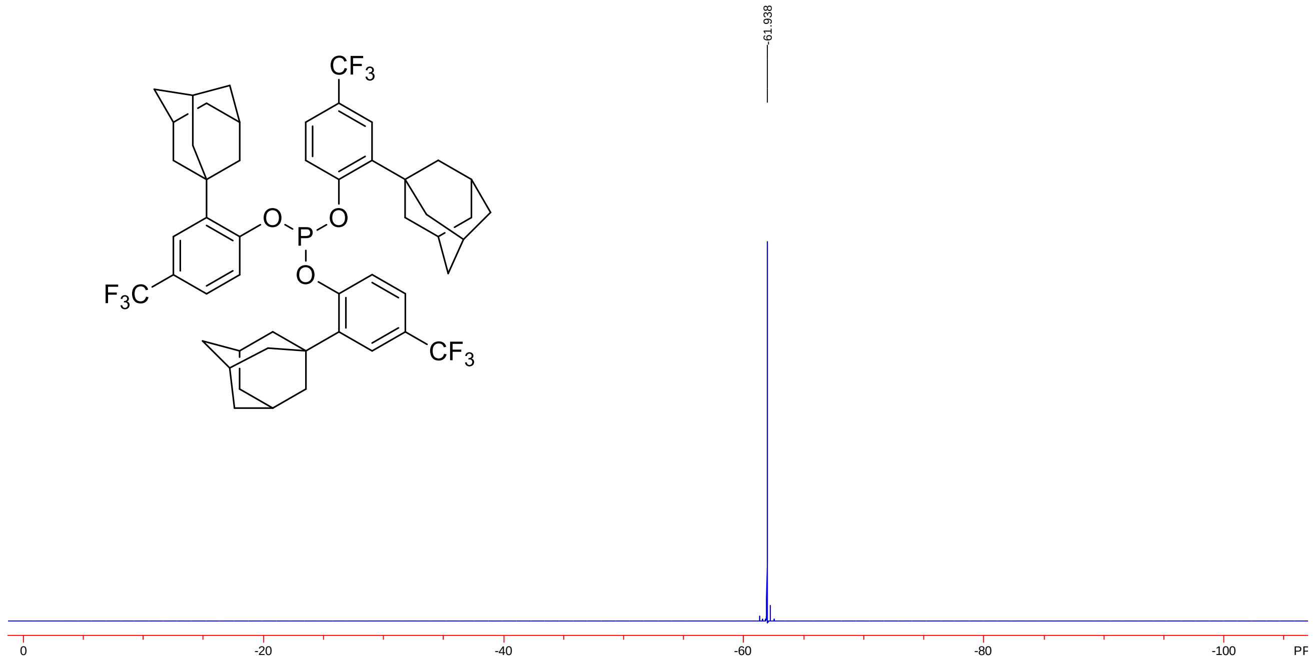

Supplementary Figure 33. ^{19}F NMR of compound **L3**

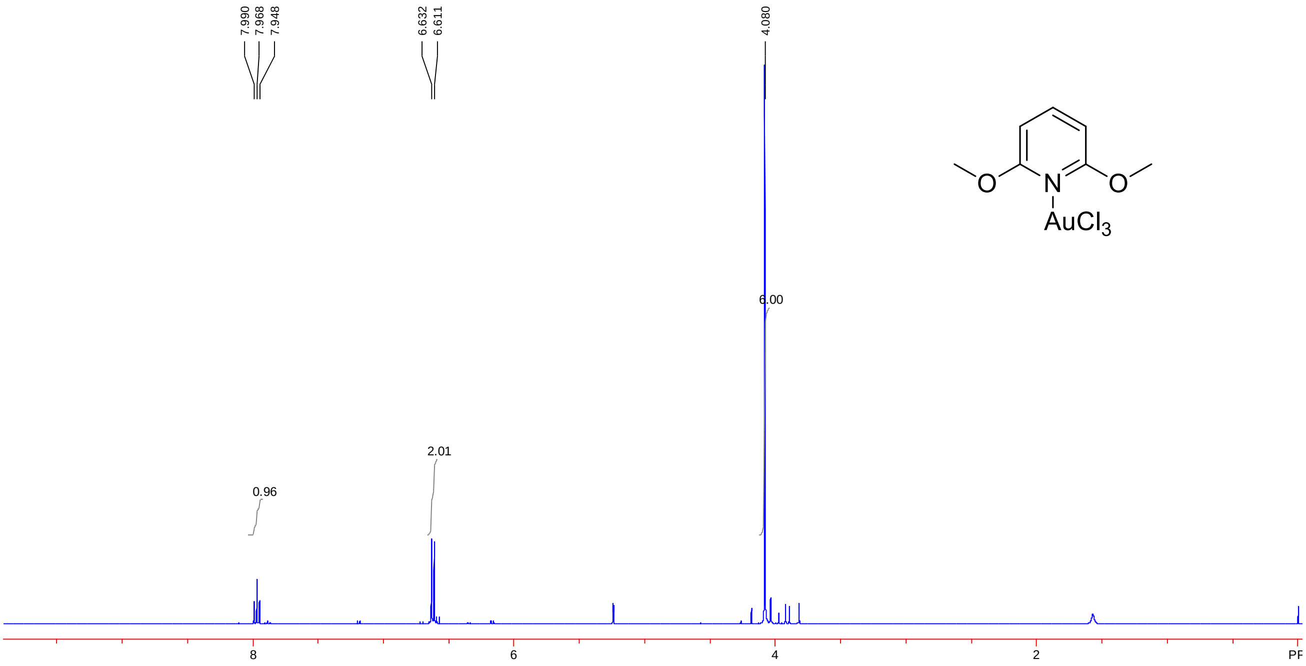

Supplementary Figure 34. ^{1}H NMR of catalyst **6**

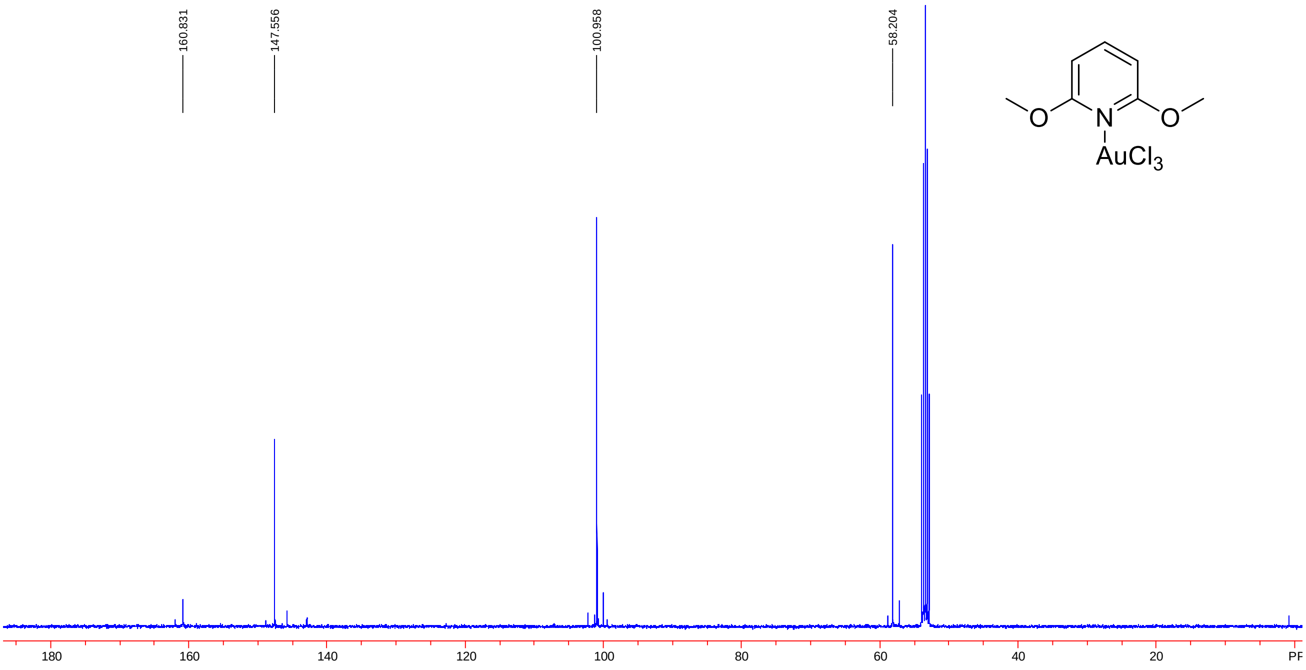

Supplementary Figure 35. ^{13}C NMR of catalyst **6**

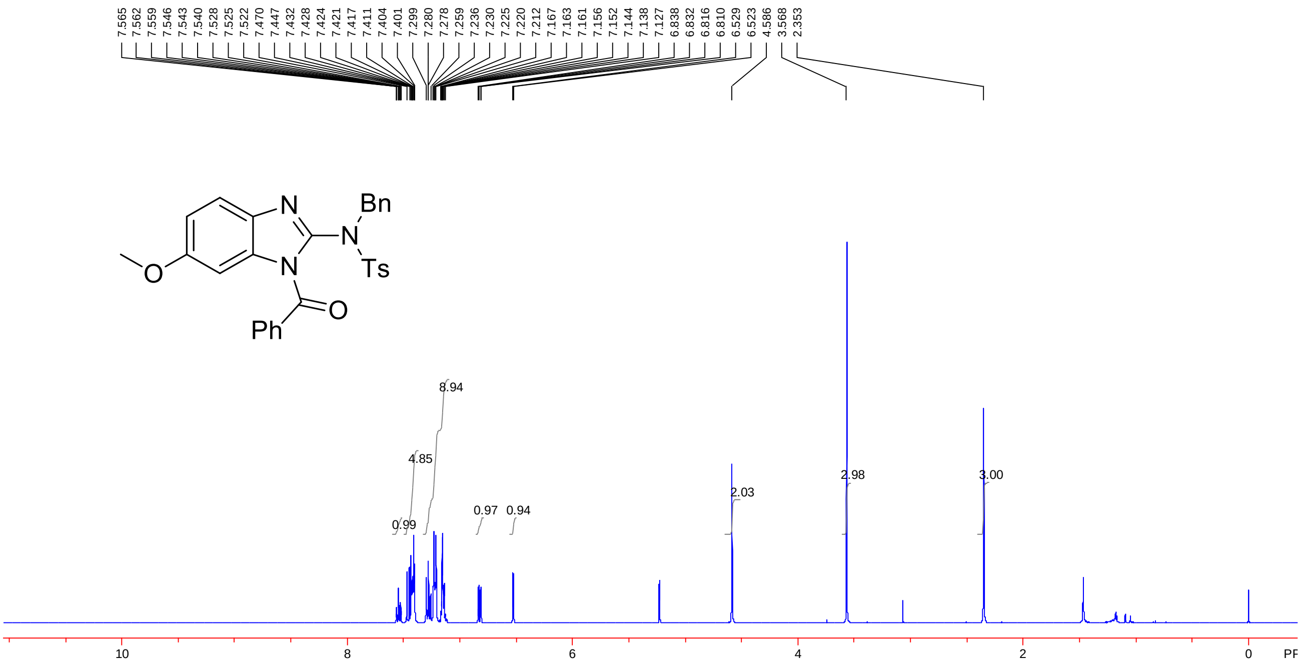

Supplementary Figure 36. ^{1}H NMR of compound **3a**

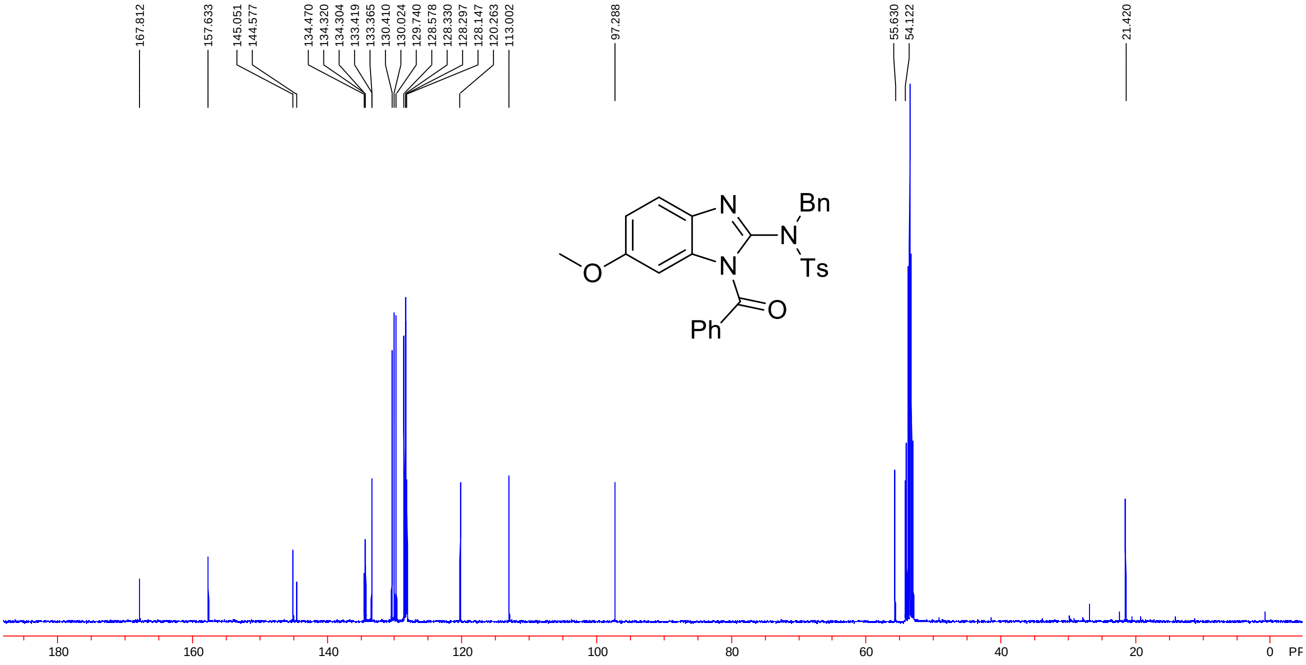

Supplementary Figure 37. ^{13}C NMR of compound **3a**

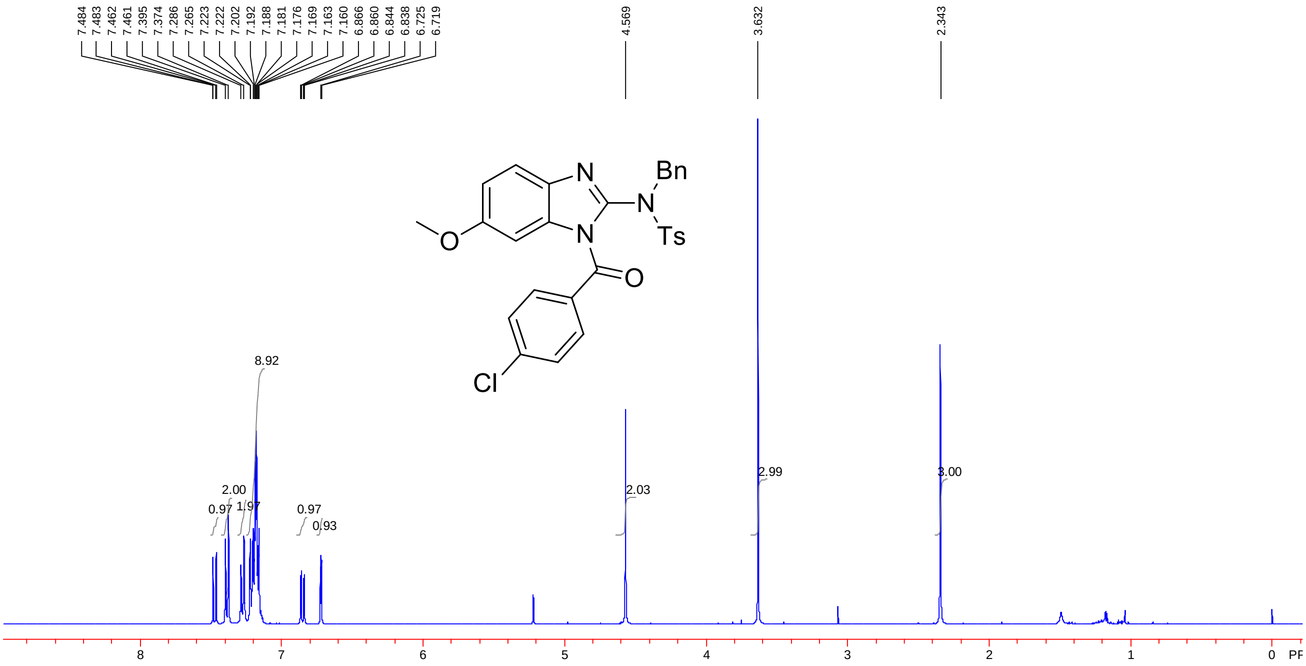

Supplementary Figure 38. ^{1}H NMR of compound **3b**

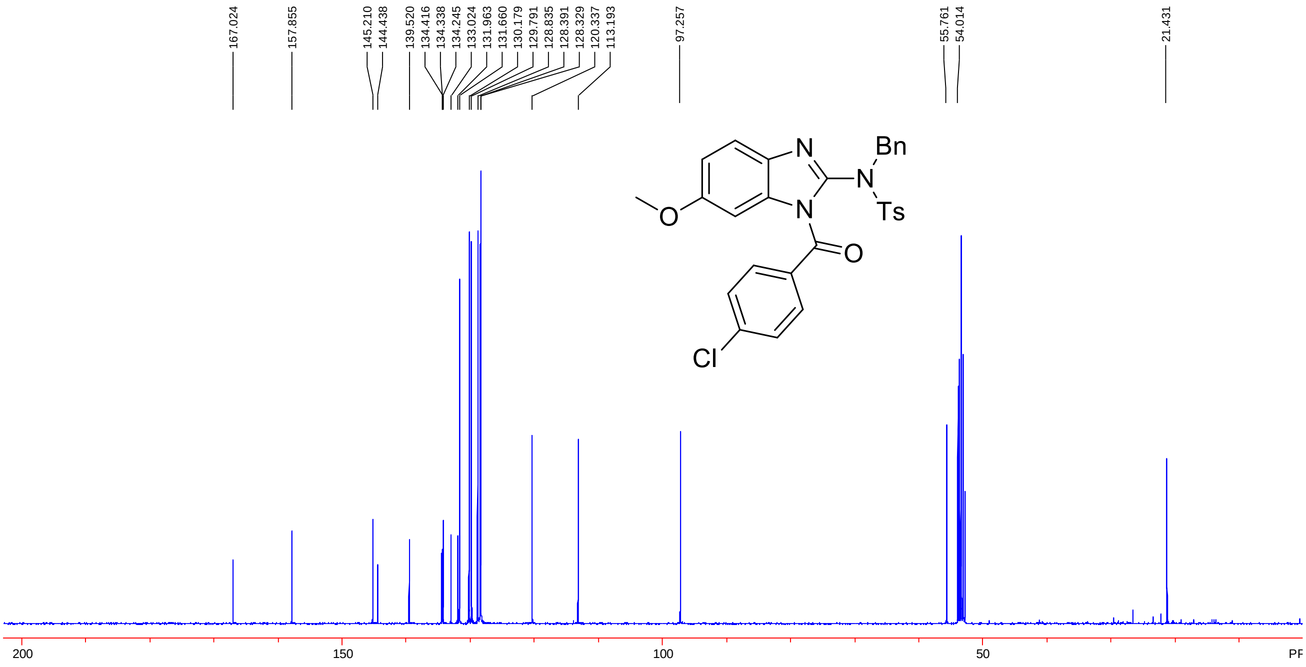

Supplementary Figure 39. ^{13}C NMR of compound **3b**

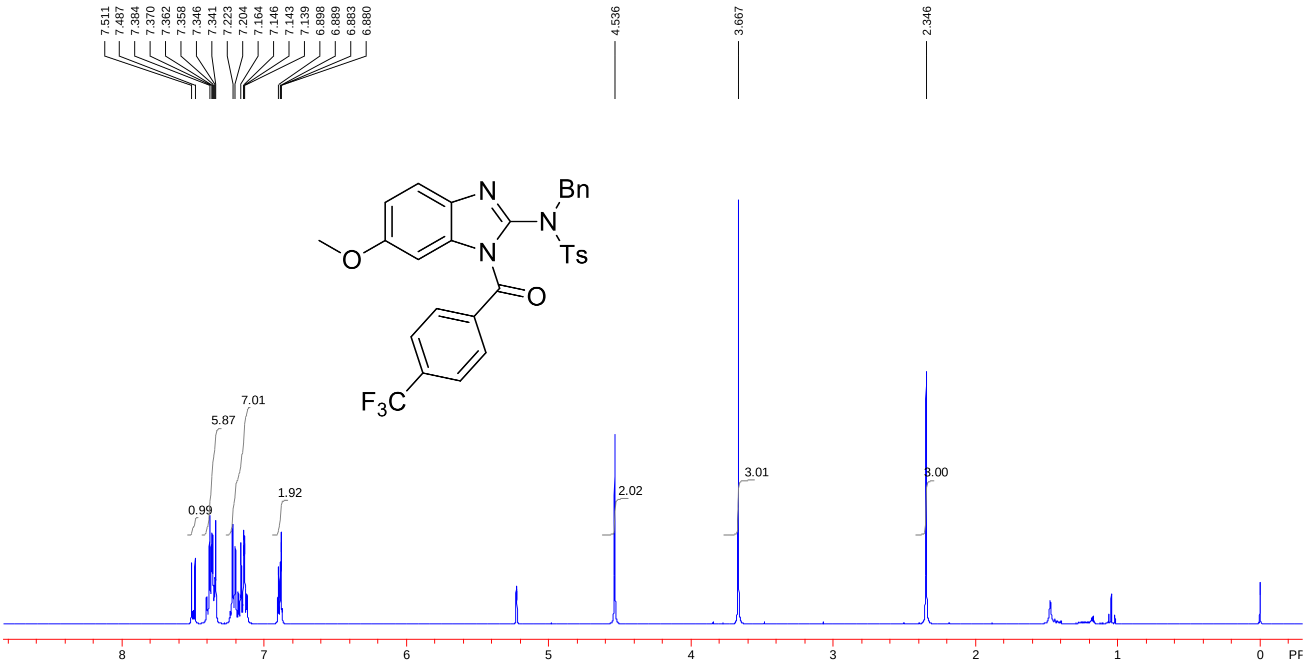

Supplementary Figure 40. ^{1}H NMR of compound **3c**

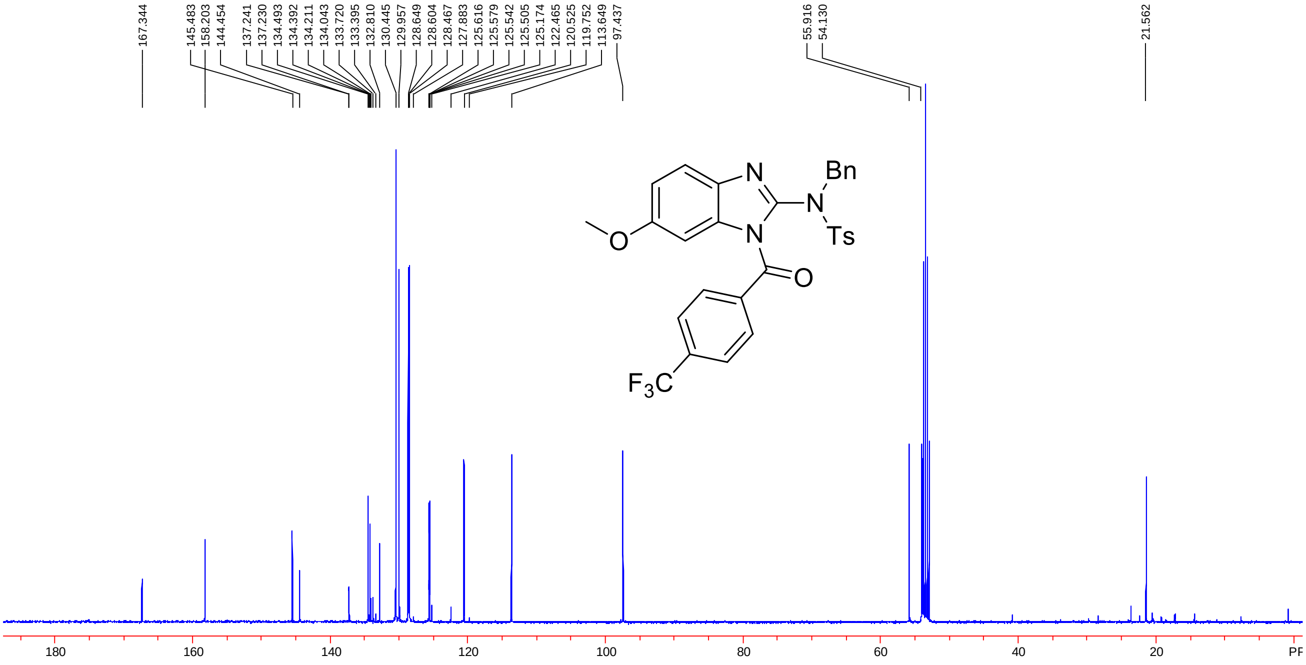

Supplementary Figure 41. ^{13}C NMR of compound **3c**

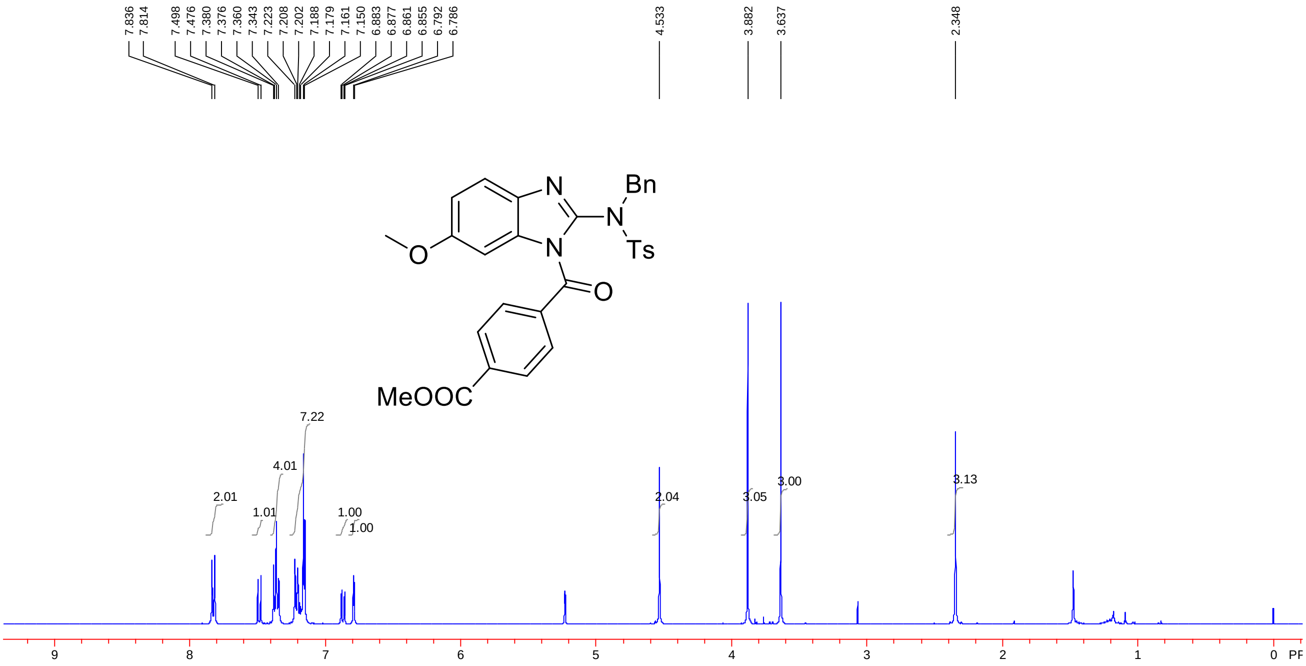

Supplementary Figure 42. ^{1}H NMR of compound **3d**

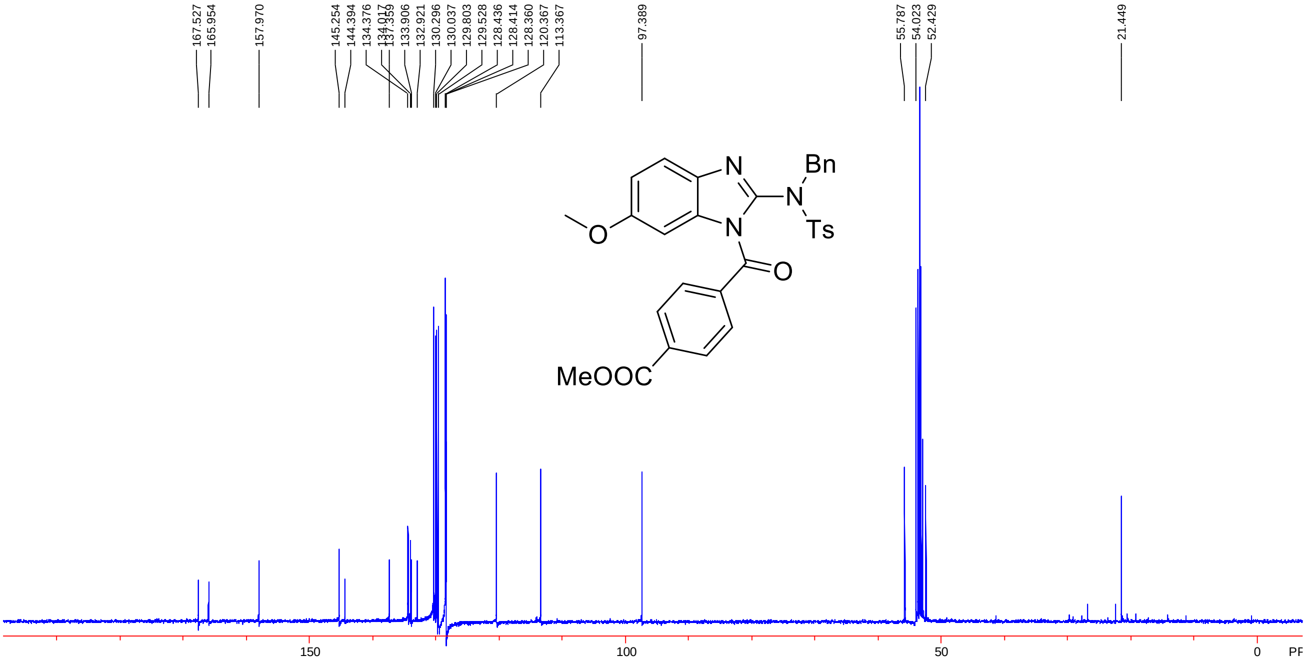

Supplementary Figure 43. ^{13}C NMR of compound **3d**

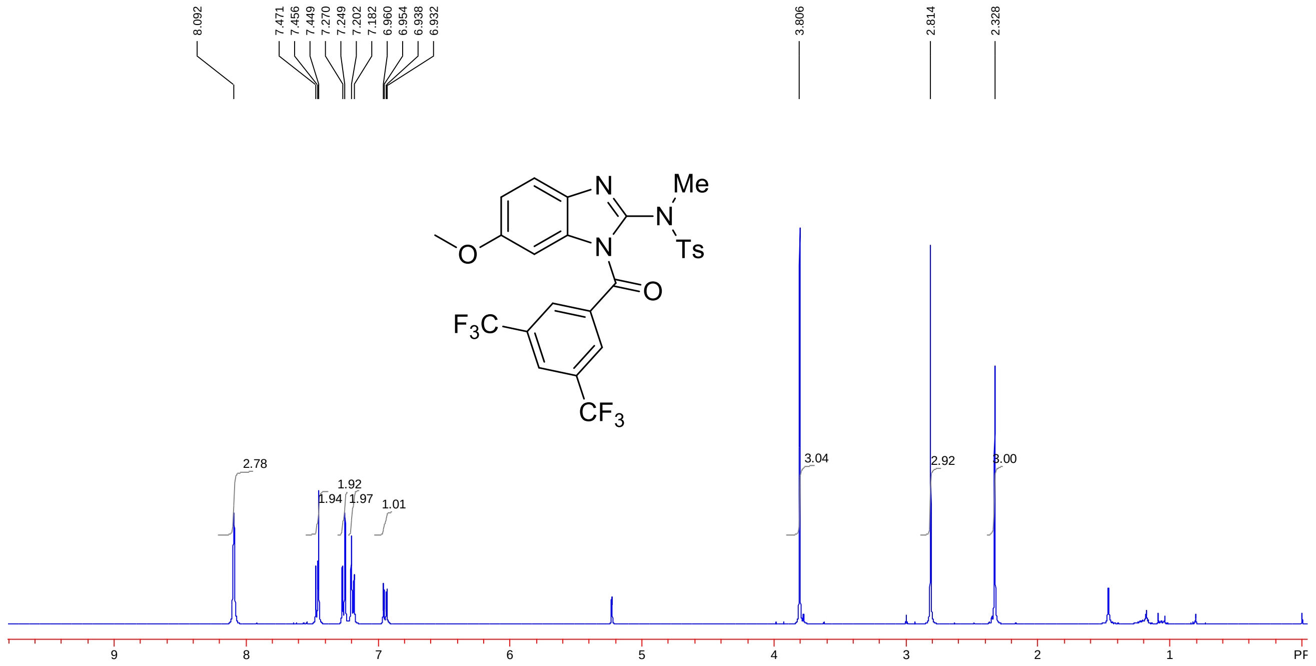

Supplementary Figure 44. ^{1}H NMR of compound **3e**

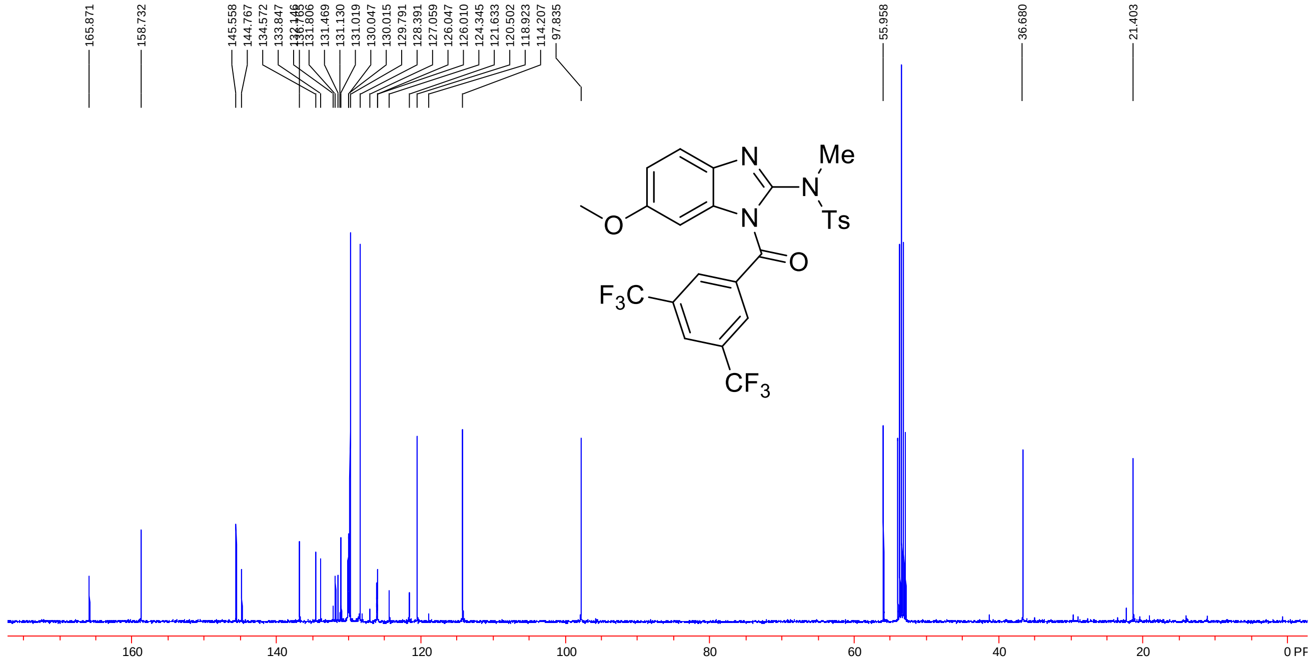

Supplementary Figure 45. ^{13}C NMR of compound **3e**

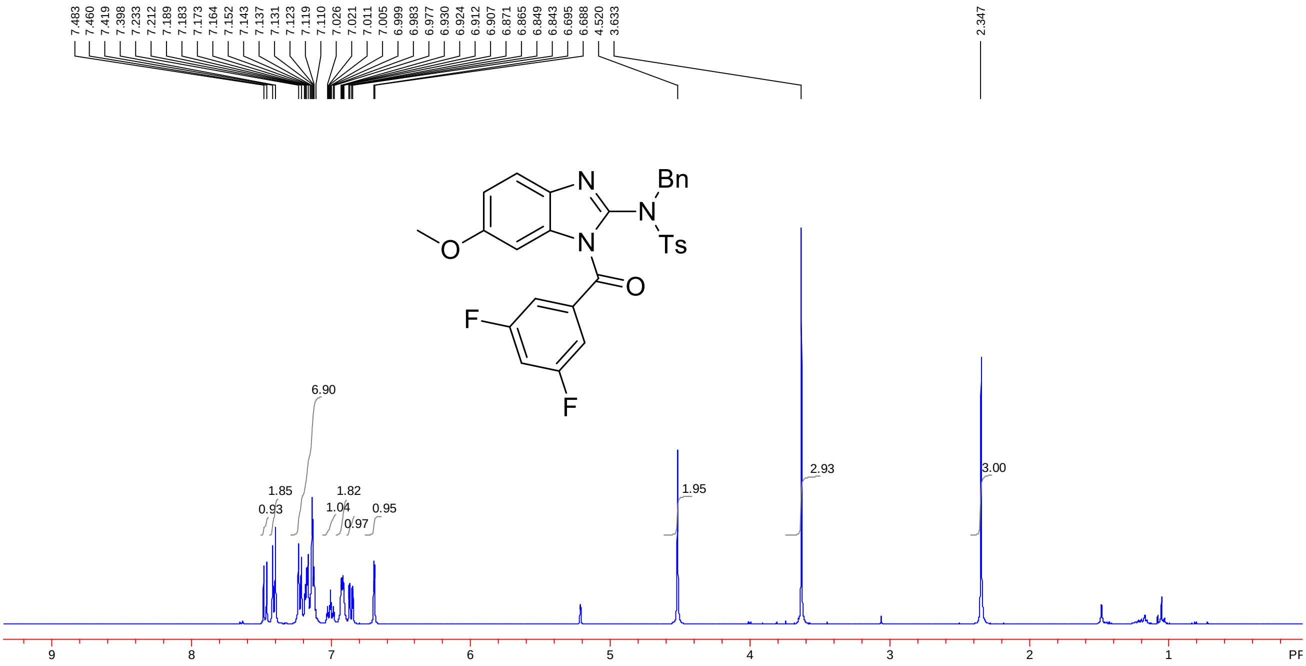

Supplementary Figure 46. ^{1}H NMR of compound **3f**

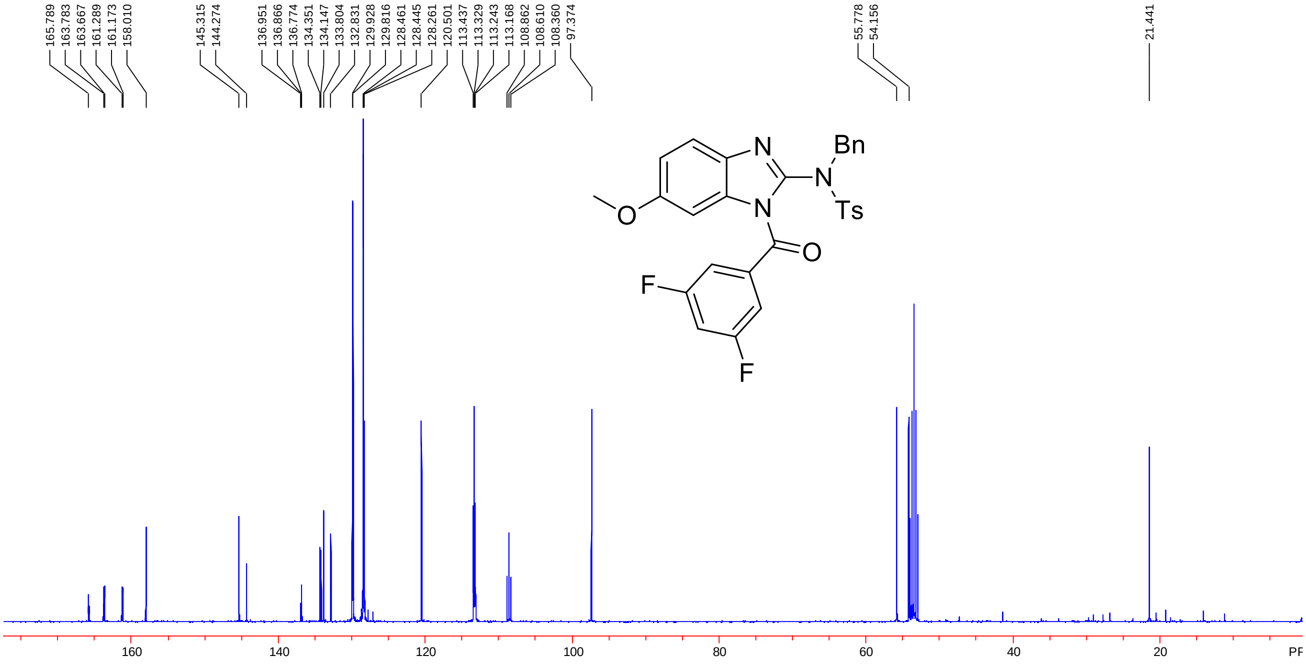

Supplementary Figure 47. ^{13}C NMR of compound **3f**

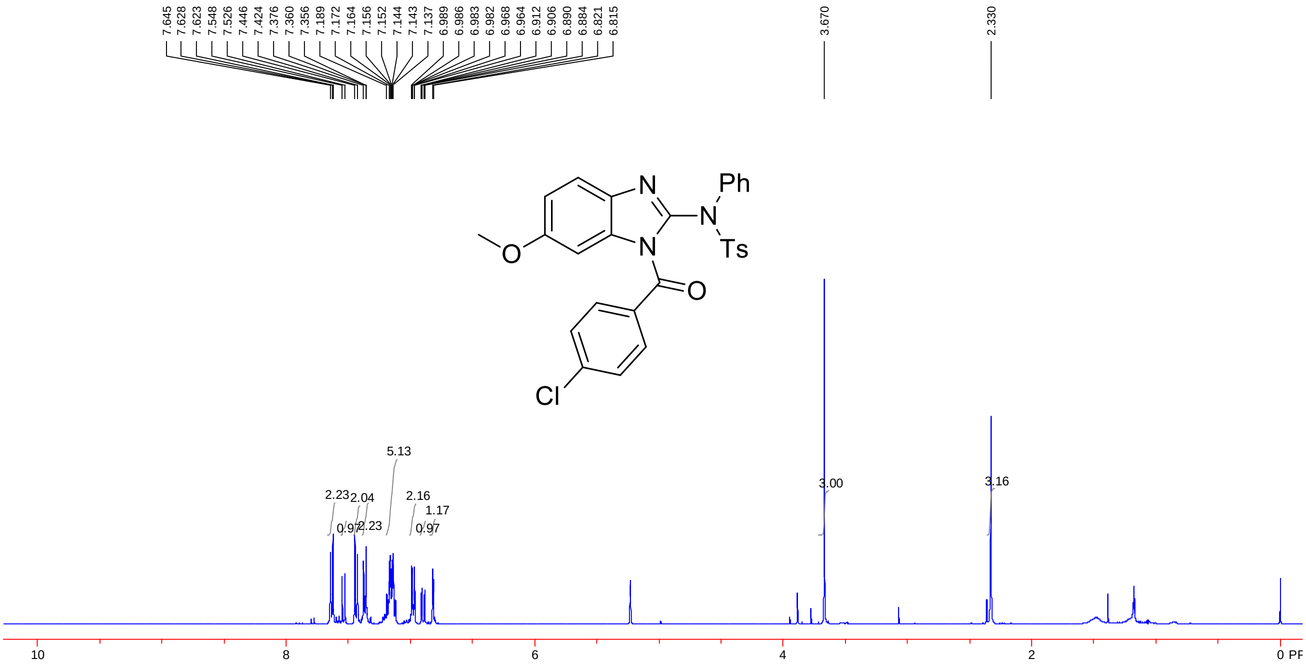

Supplementary Figure 48. ^{1}H NMR of compound **3g**

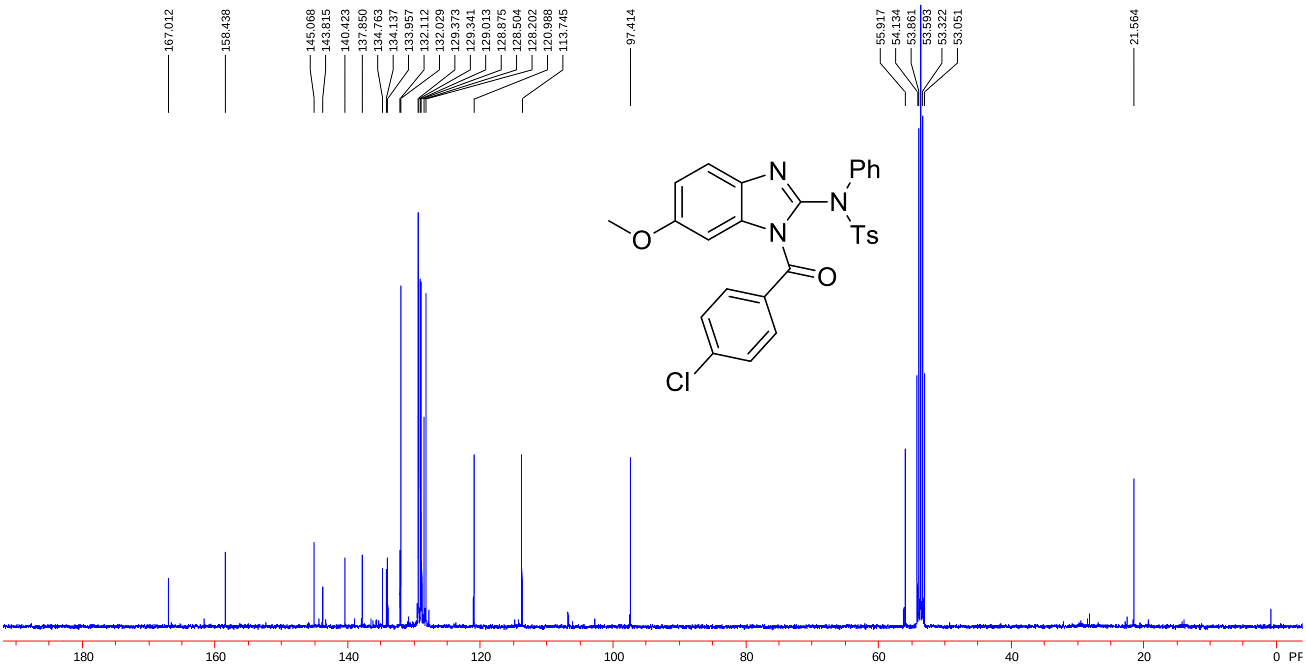

Supplementary Figure 49. ^{13}C NMR of compound **3g**

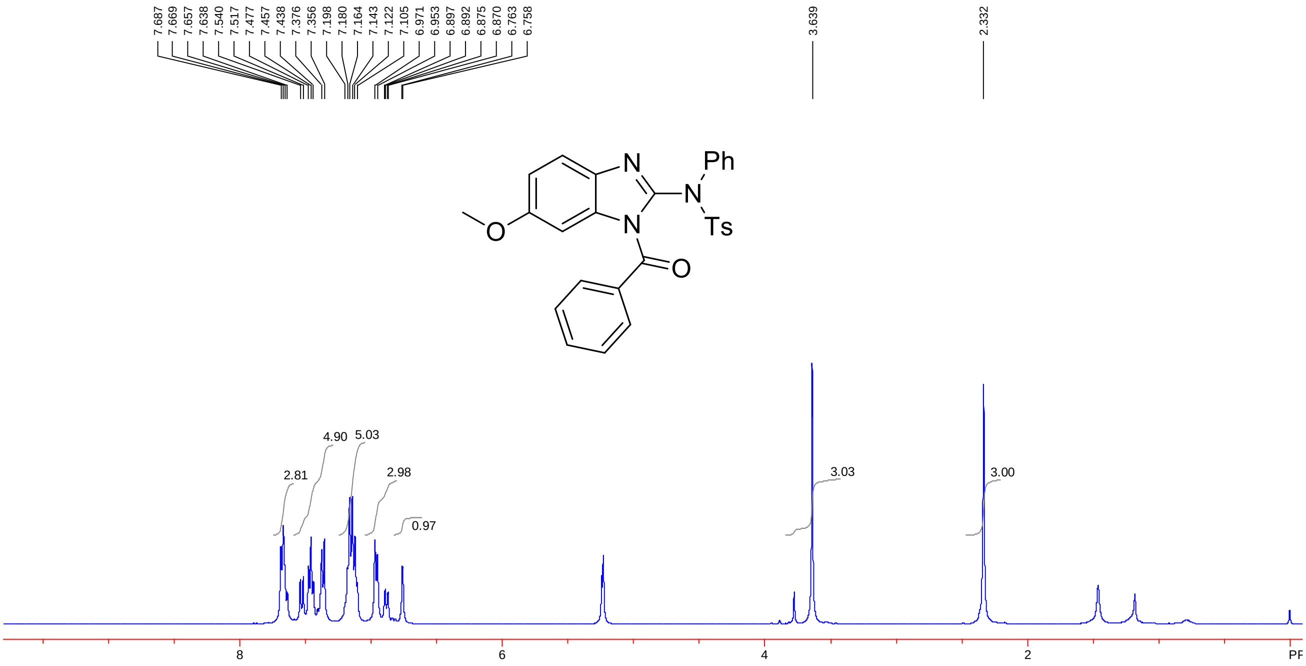

Supplementary Figure 50. ^{1}H NMR of compound **3h**

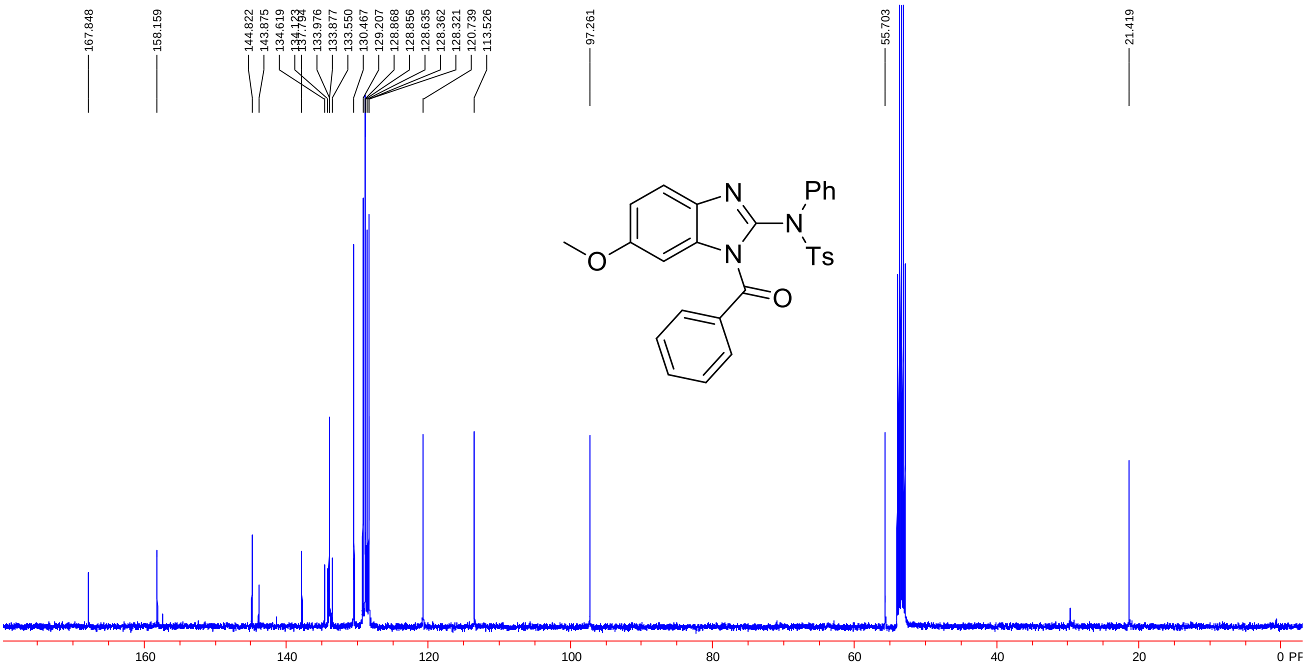

Supplementary Figure 51. ^{13}C NMR of compound **3h**

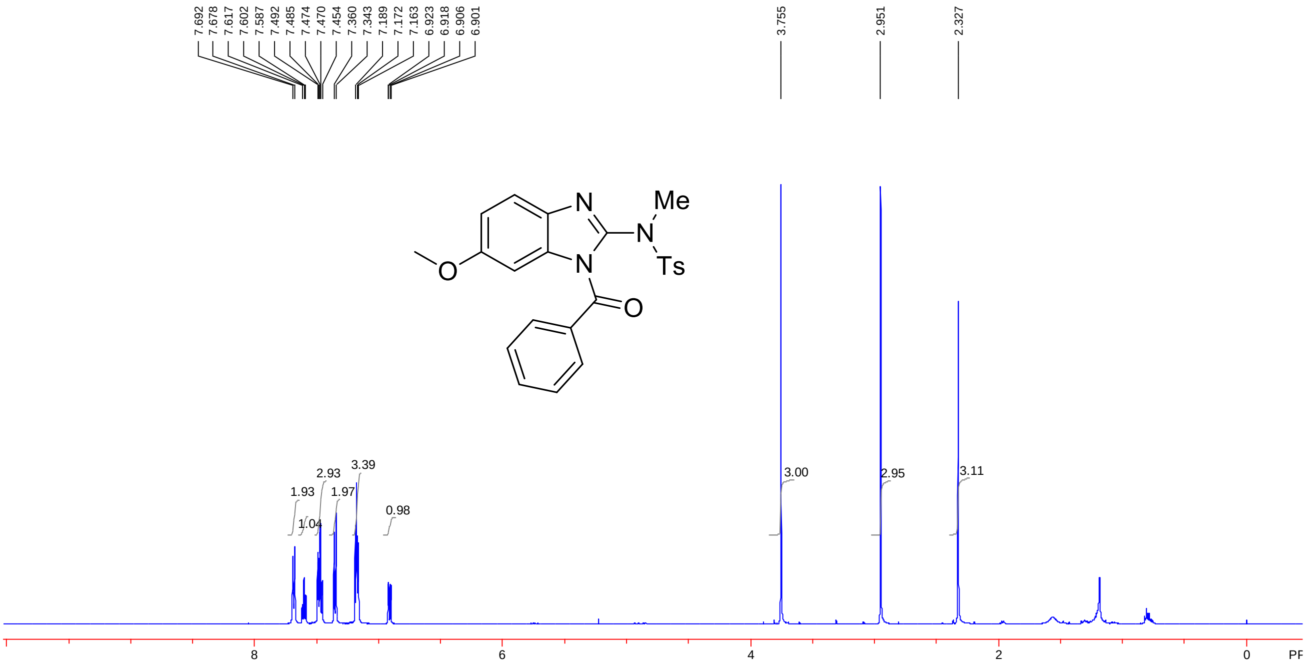

Supplementary Figure 52. ^{1}H NMR of compound **3i**

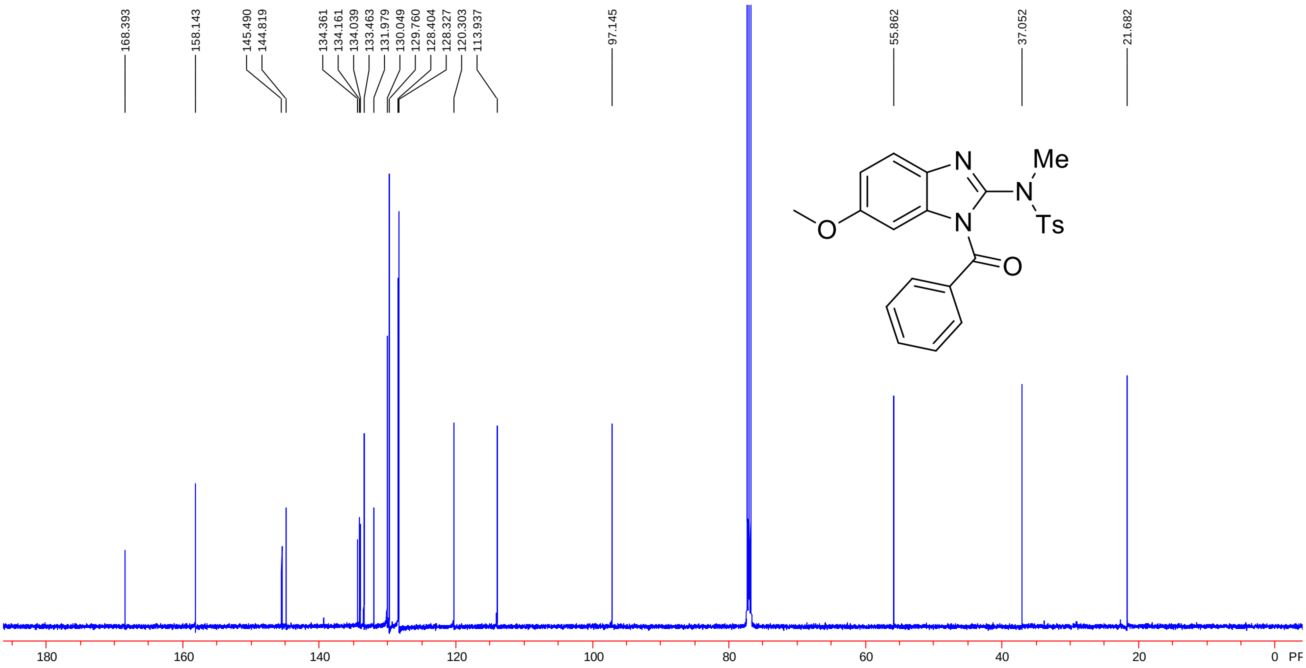

Supplementary Figure 53. ^{13}C NMR of compound **3i**

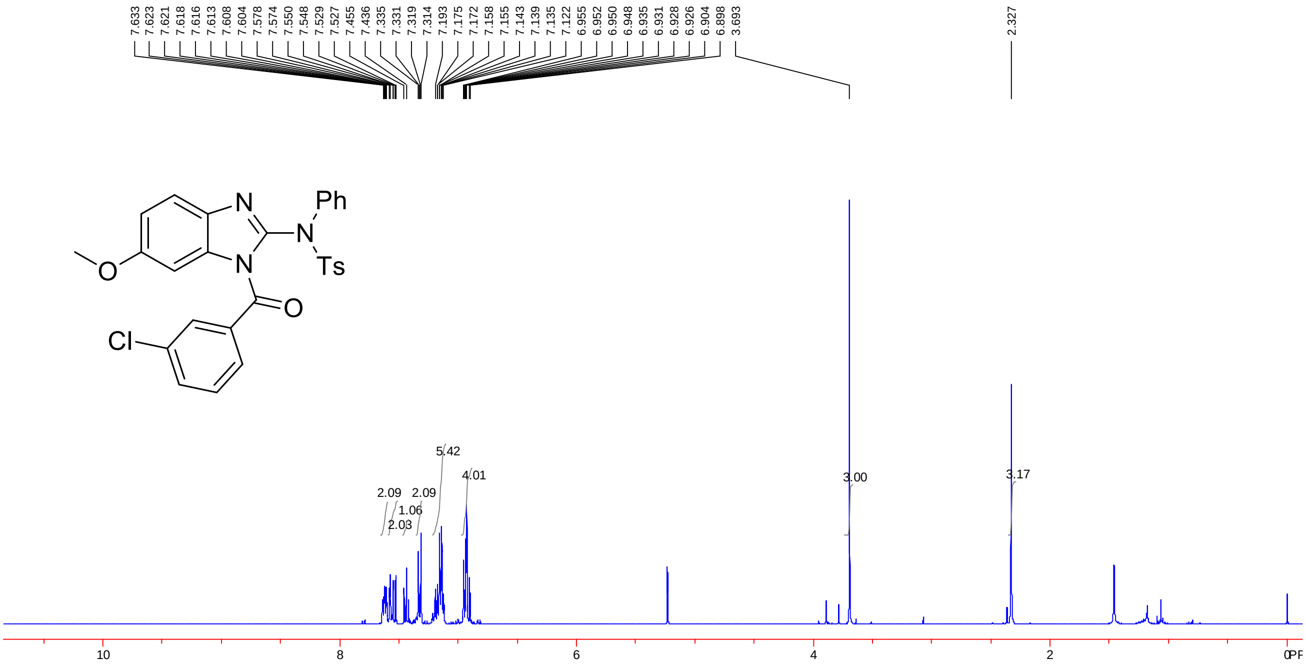

Supplementary Figure 54. ^{1}H NMR of compound **3j**

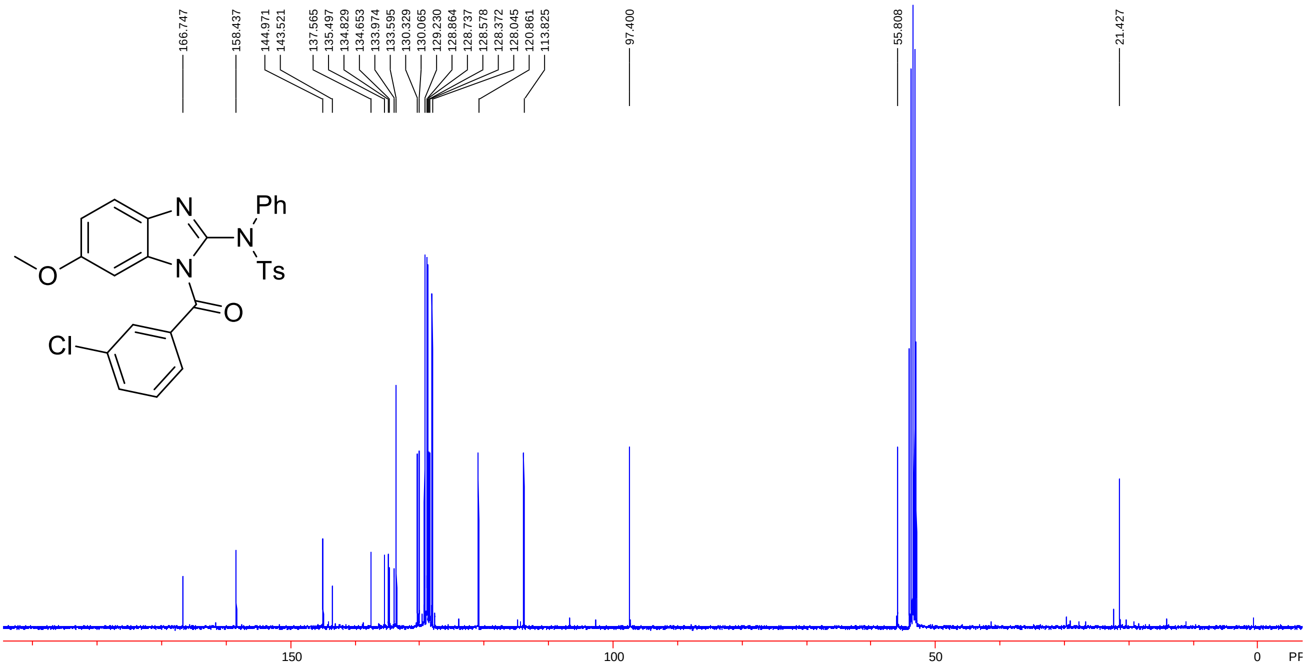

Supplementary Figure 55. ^{13}C NMR of compound **3j**

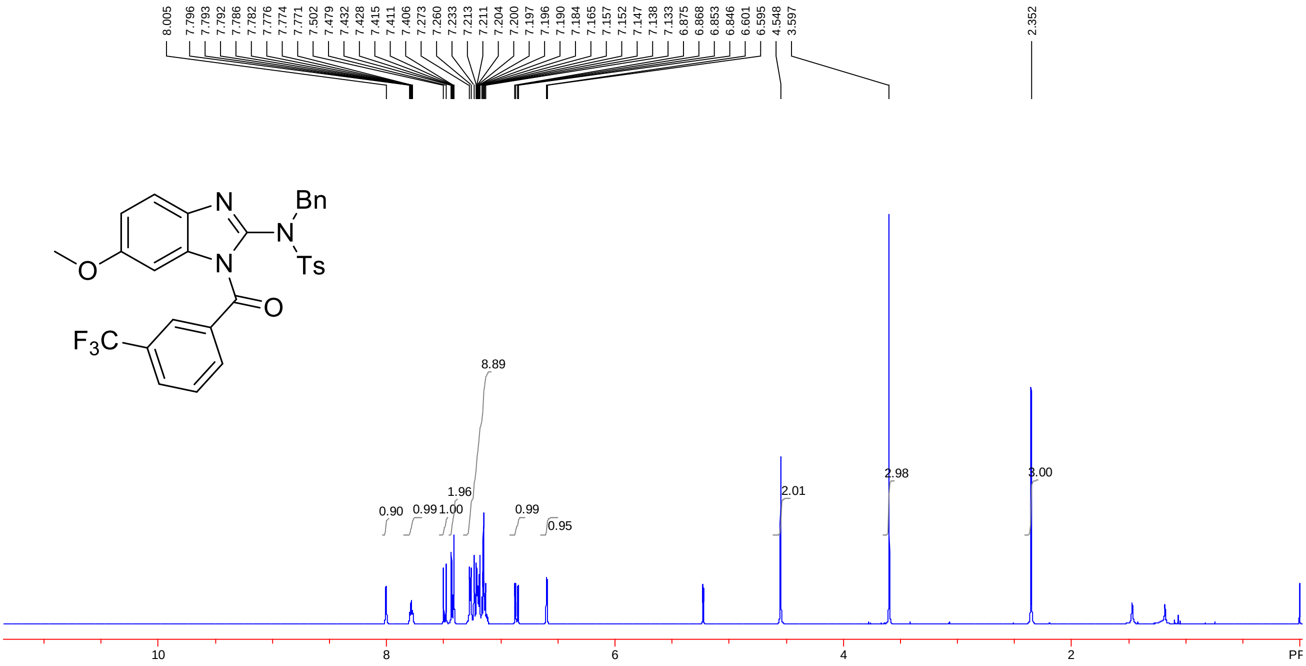

Supplementary Figure 56. ^{1}H NMR of compound **3k**

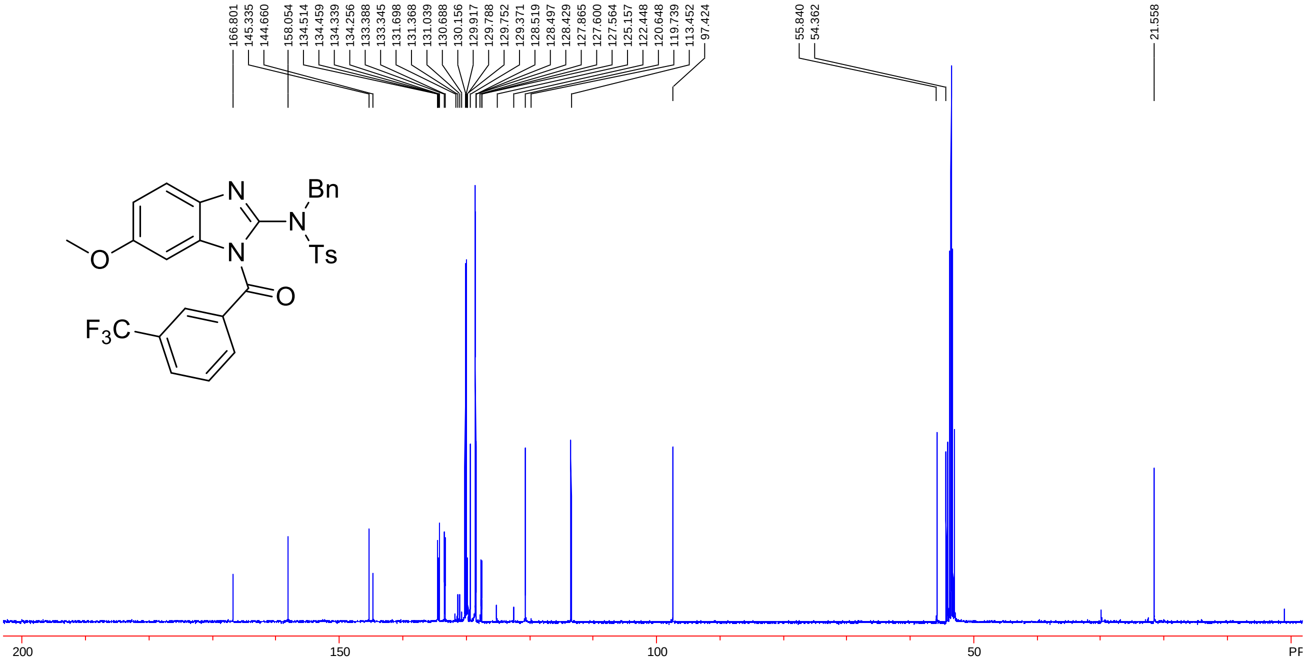

Supplementary Figure 57. ^{13}C NMR of compound **3k**

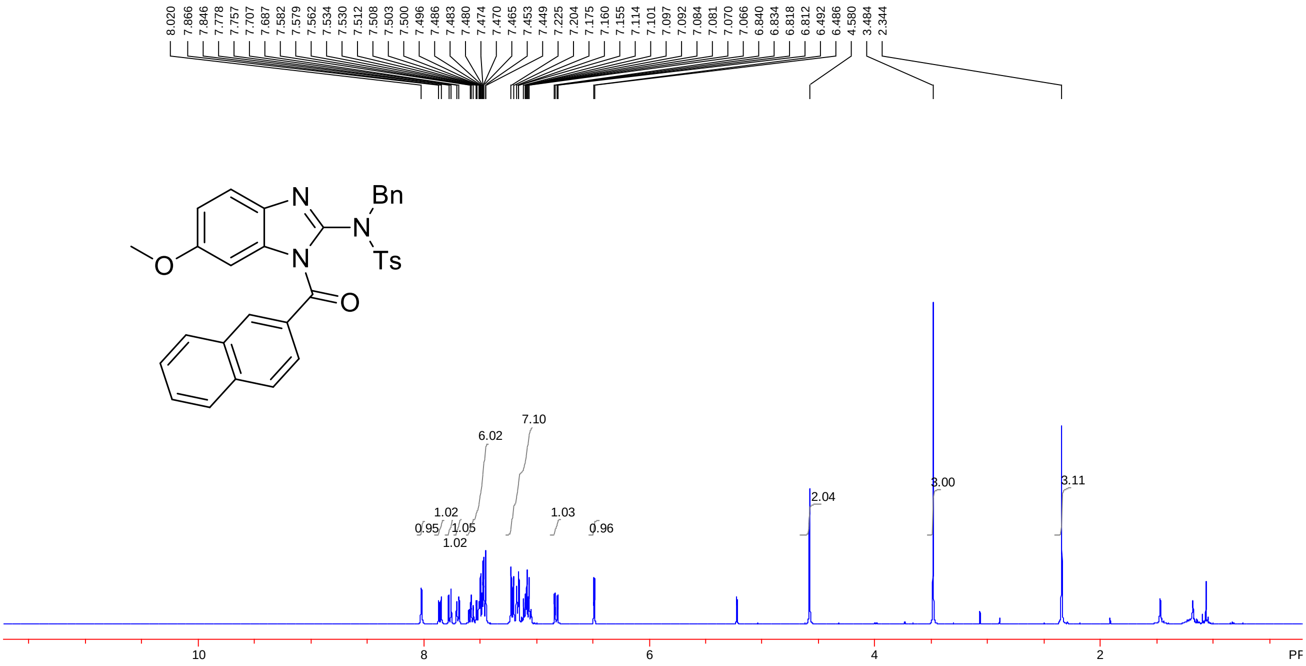

Supplementary Figure 58. ^{1}H NMR of compound **3l**

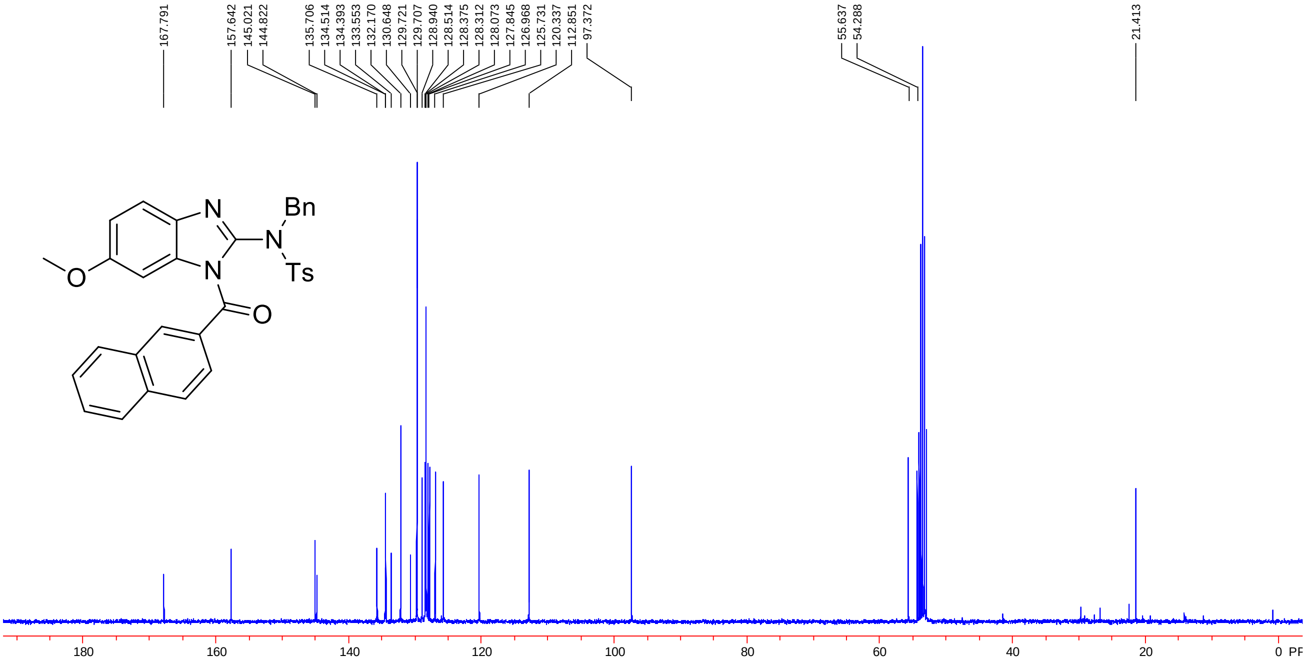

Supplementary Figure 59. ^{13}C NMR of compound **3l**

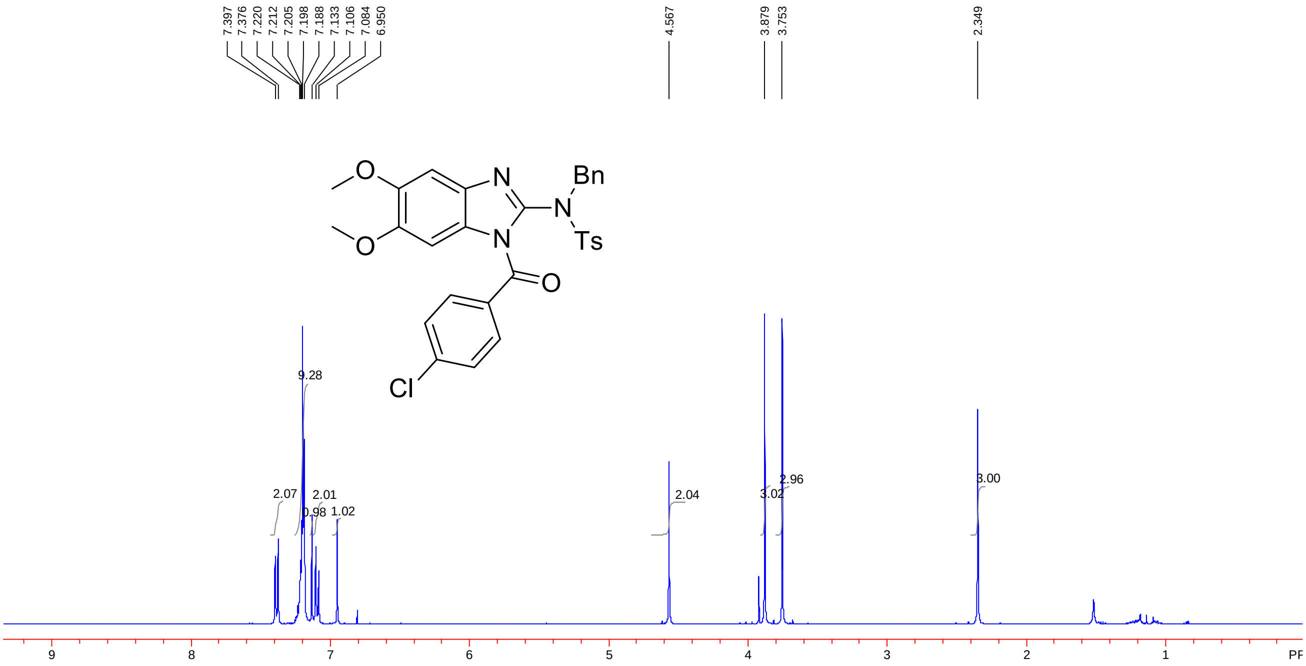

Supplementary Figure 60. ^{1}H NMR of compound **3m**

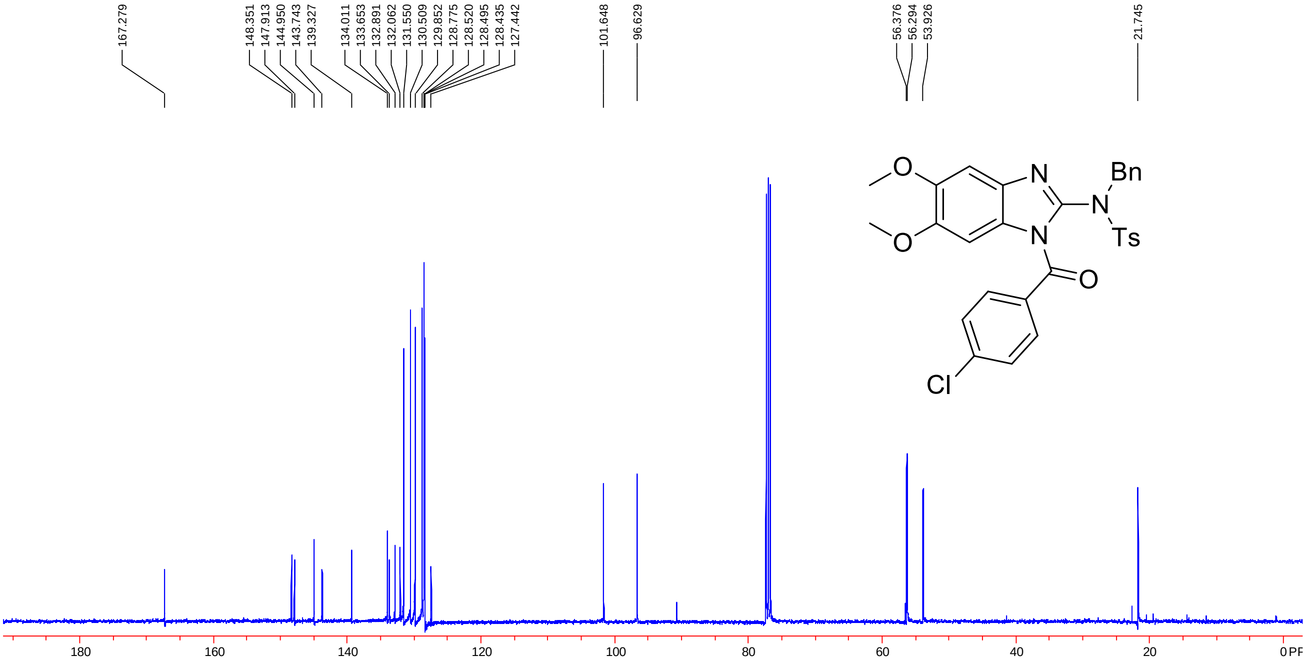

Supplementary Figure 61. ^{13}C NMR of compound **3m**

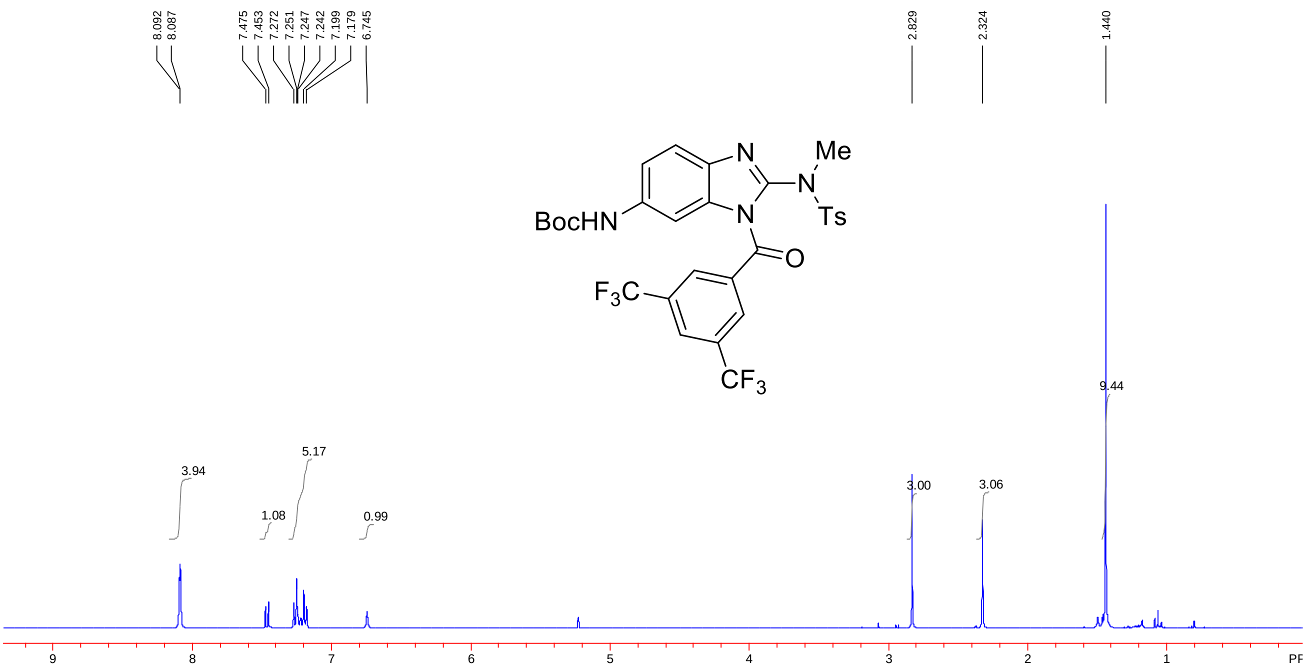

Supplementary Figure 62. ^{1}H NMR of compound **3n**

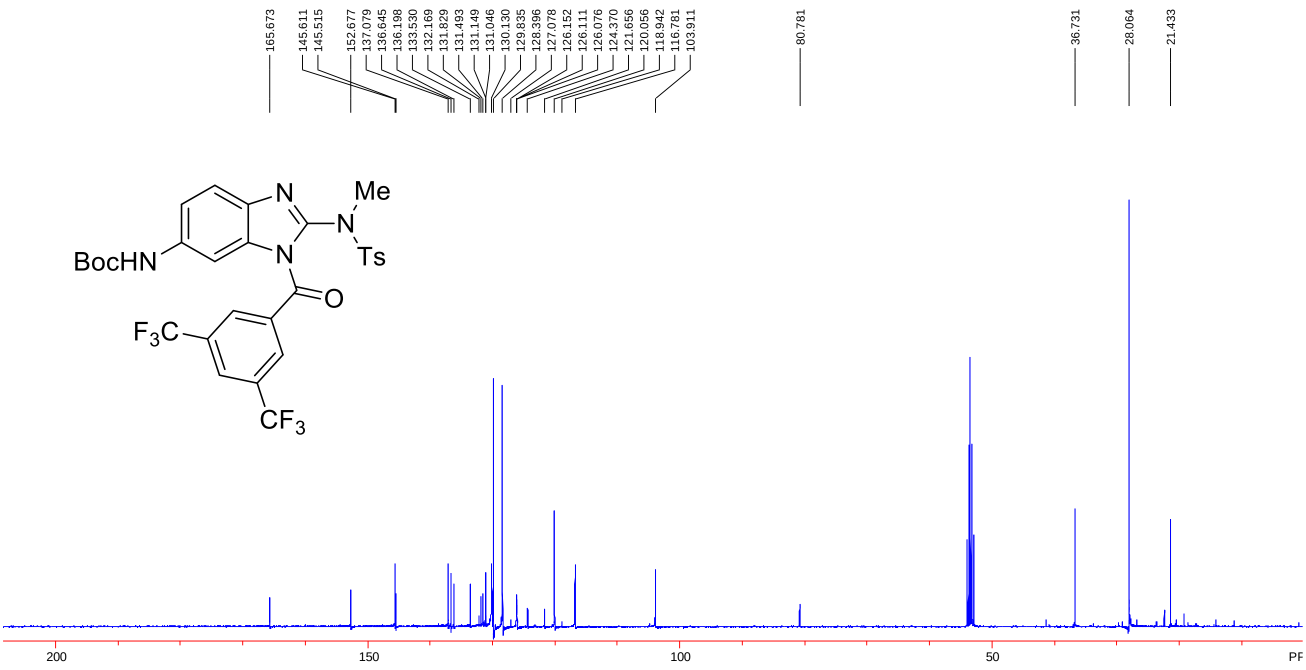

Supplementary Figure 63. ^{13}C NMR of compound **3n**

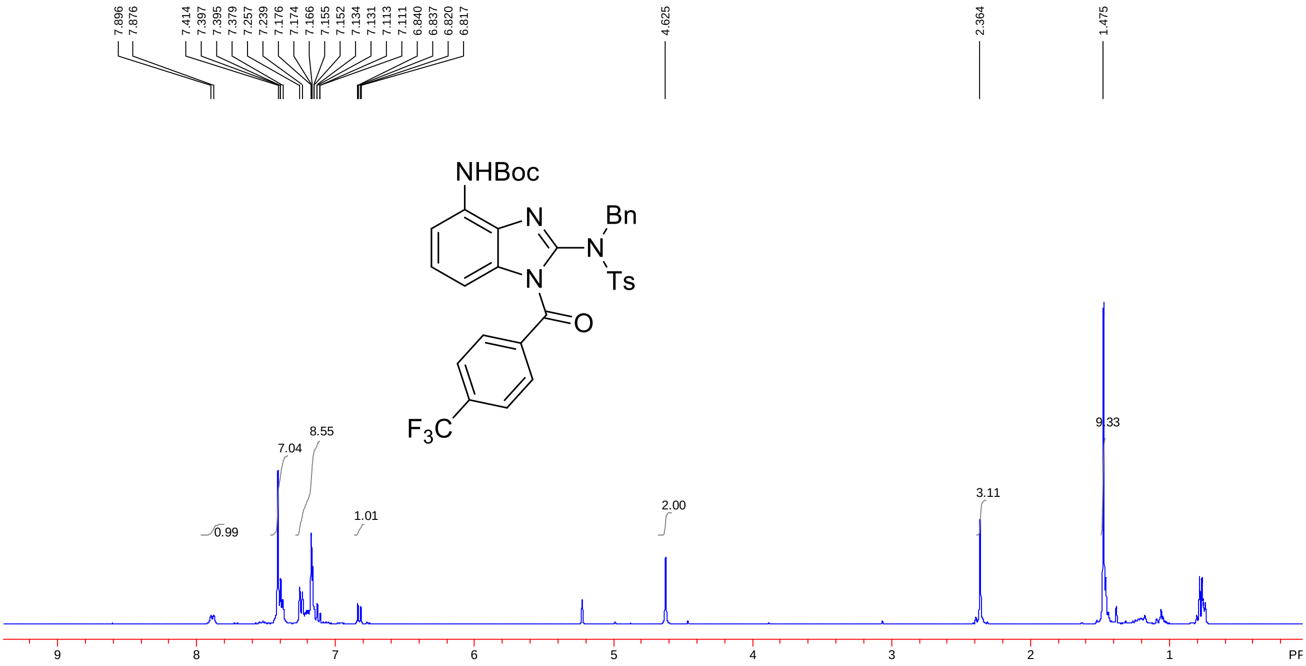

Supplementary Figure 64. ^{1}H NMR of compound **3o**

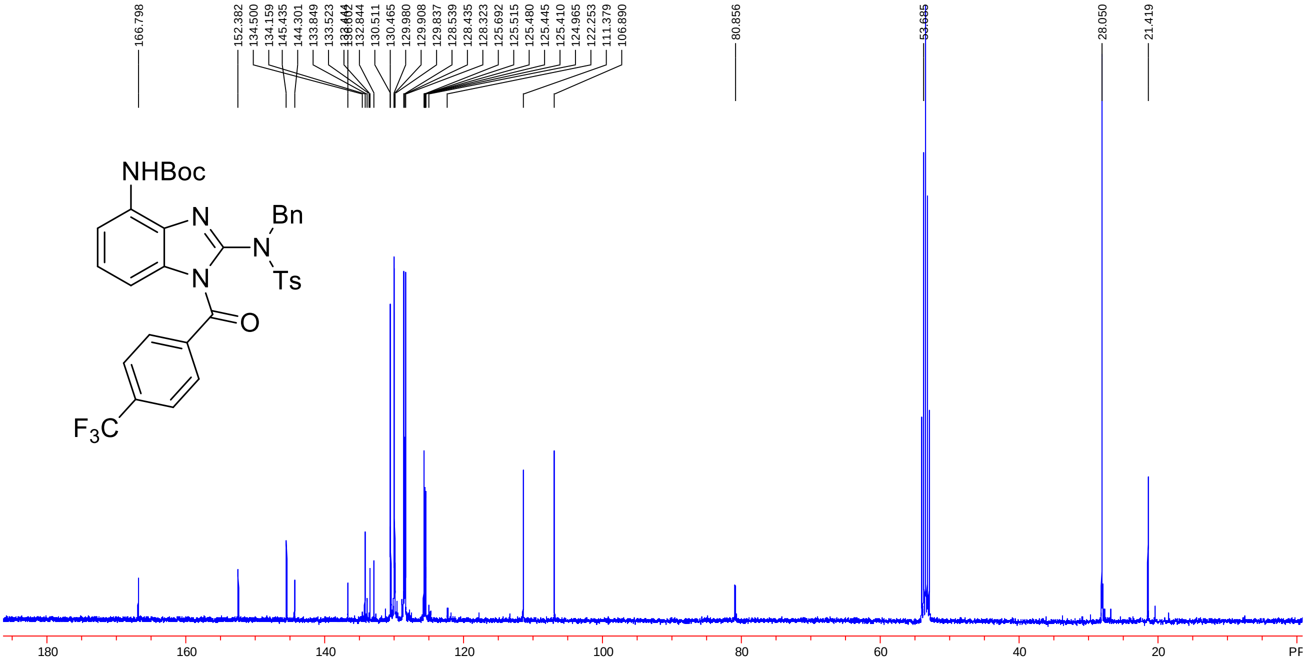

Supplementary Figure 65. ^{13}C NMR of compound **3o**

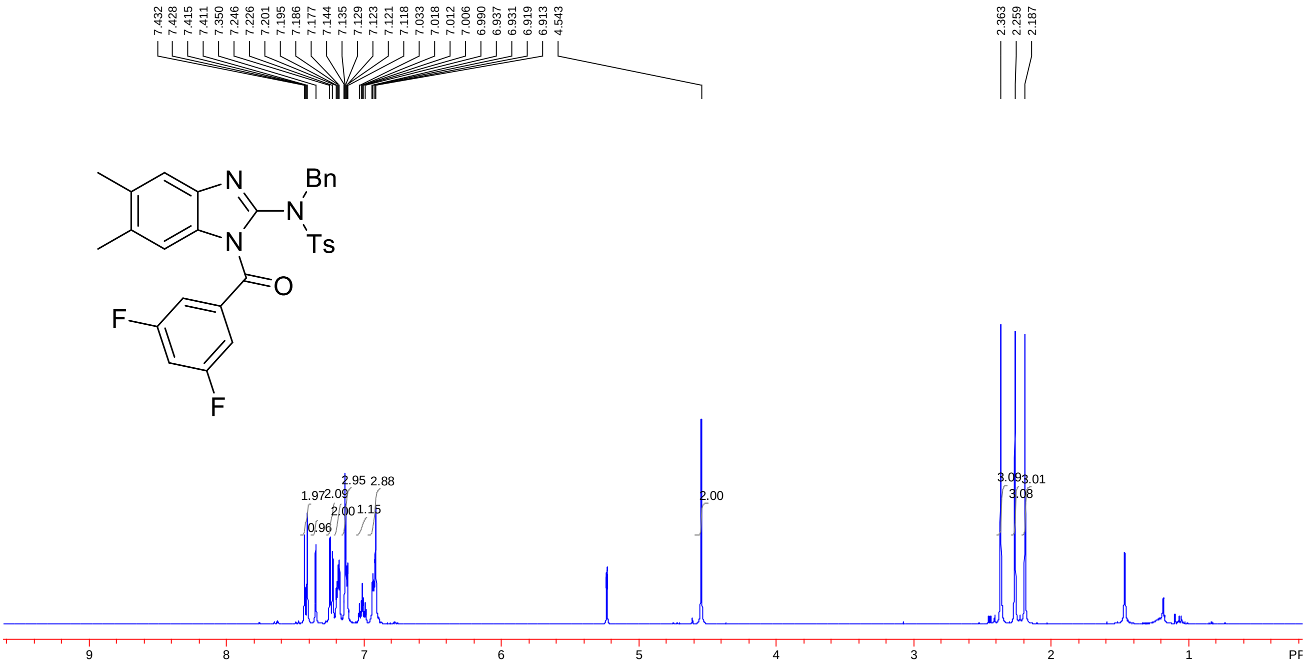

Supplementary Figure 66. ^{1}H NMR of compound **3p**

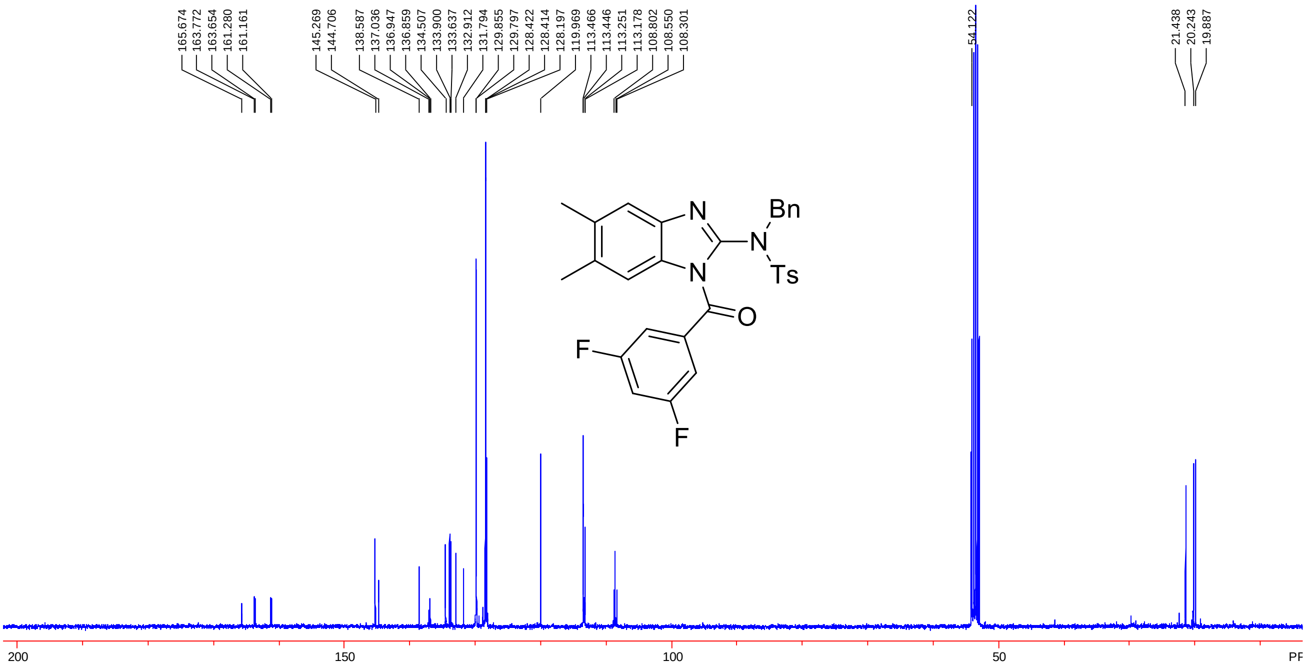

Supplementary Figure 67. ^{13}C NMR of compound **3p**

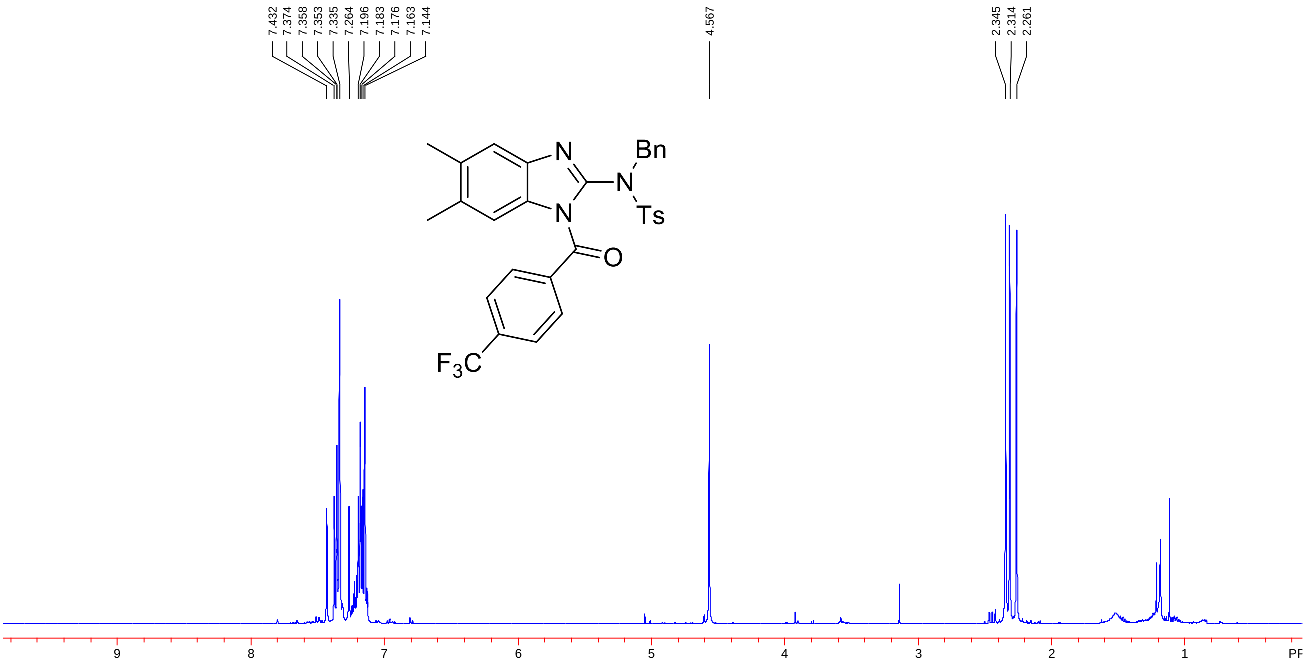

Supplementary Figure 68. ^{1}H NMR of compound **3q**

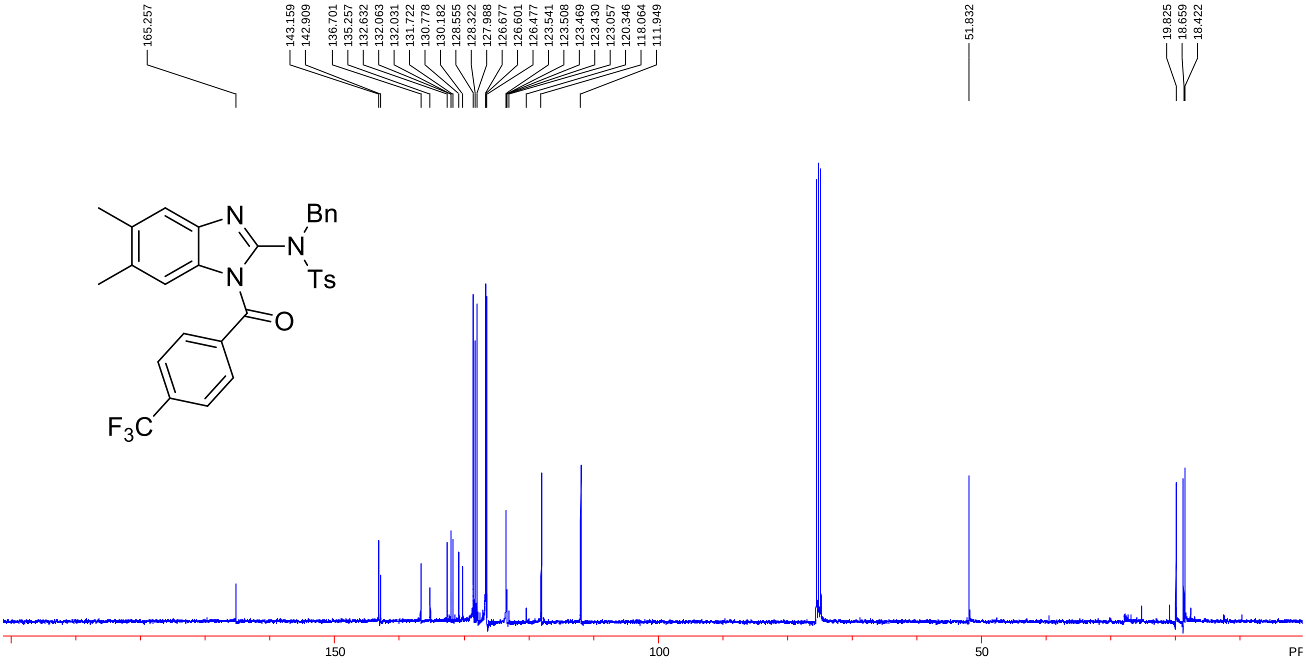

Supplementary Figure 69. ^{13}C NMR of compound **3q**

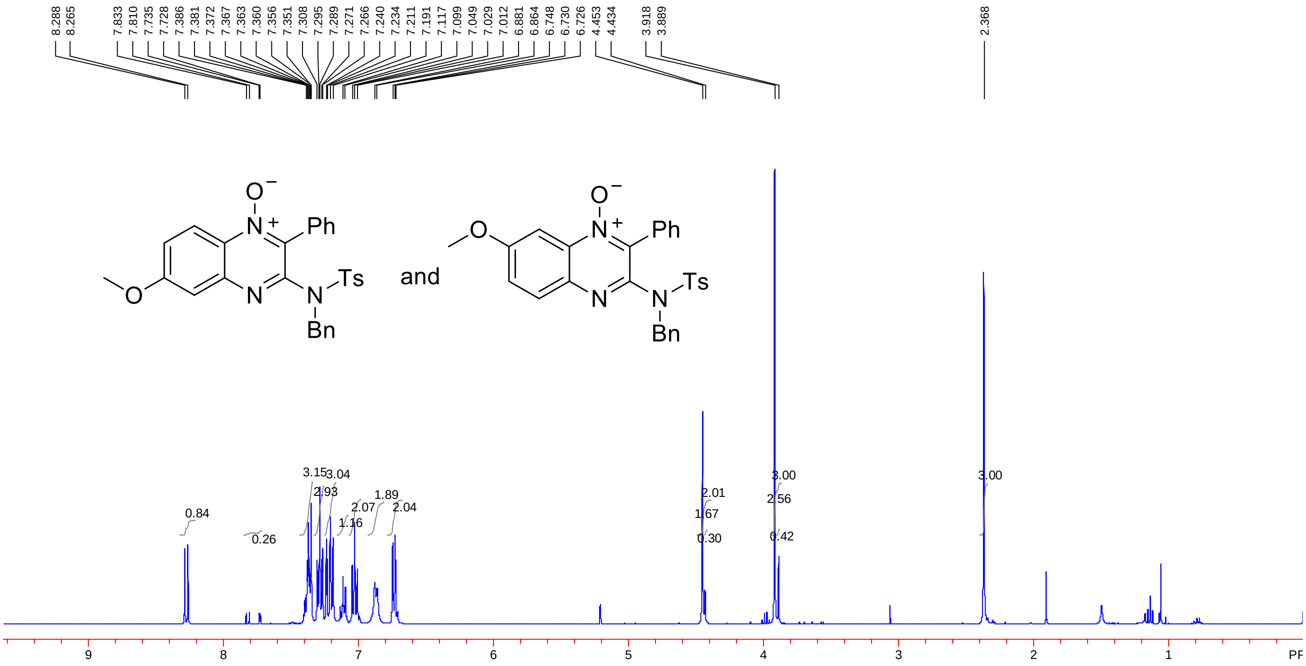

Supplementary Figure 70. ^{1}H NMR of compound **4a**

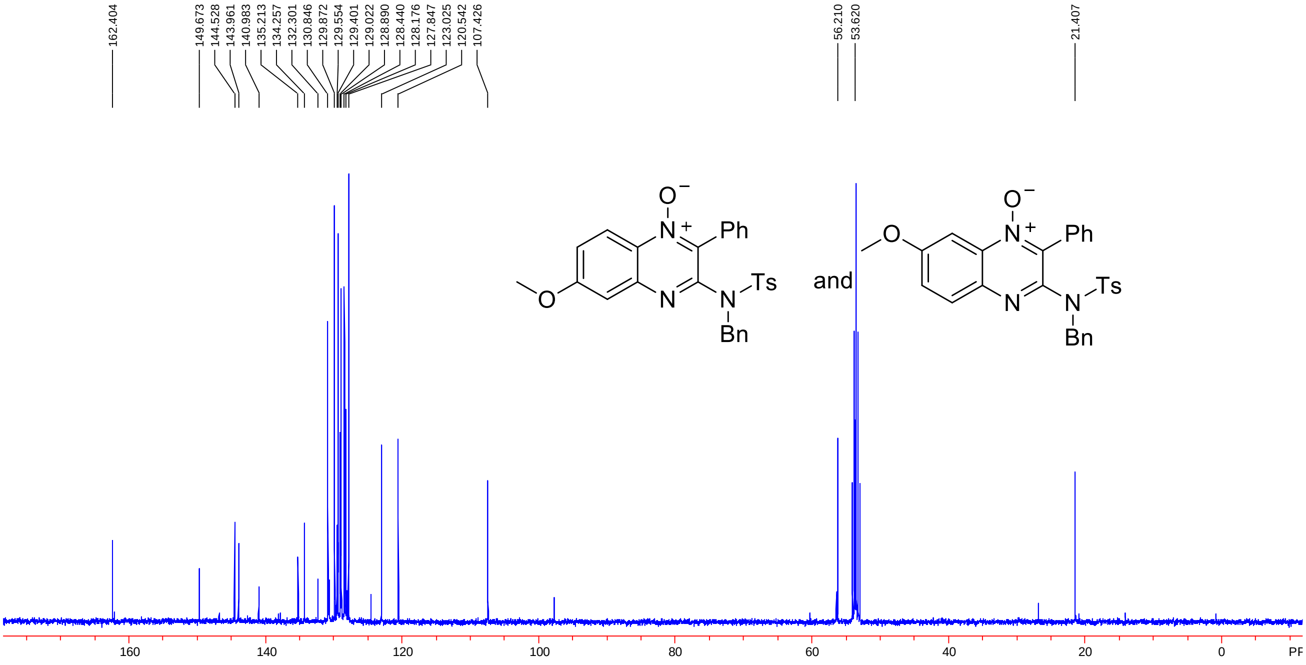

Supplementary Figure 71. ^{13}C NMR of compound **4a**

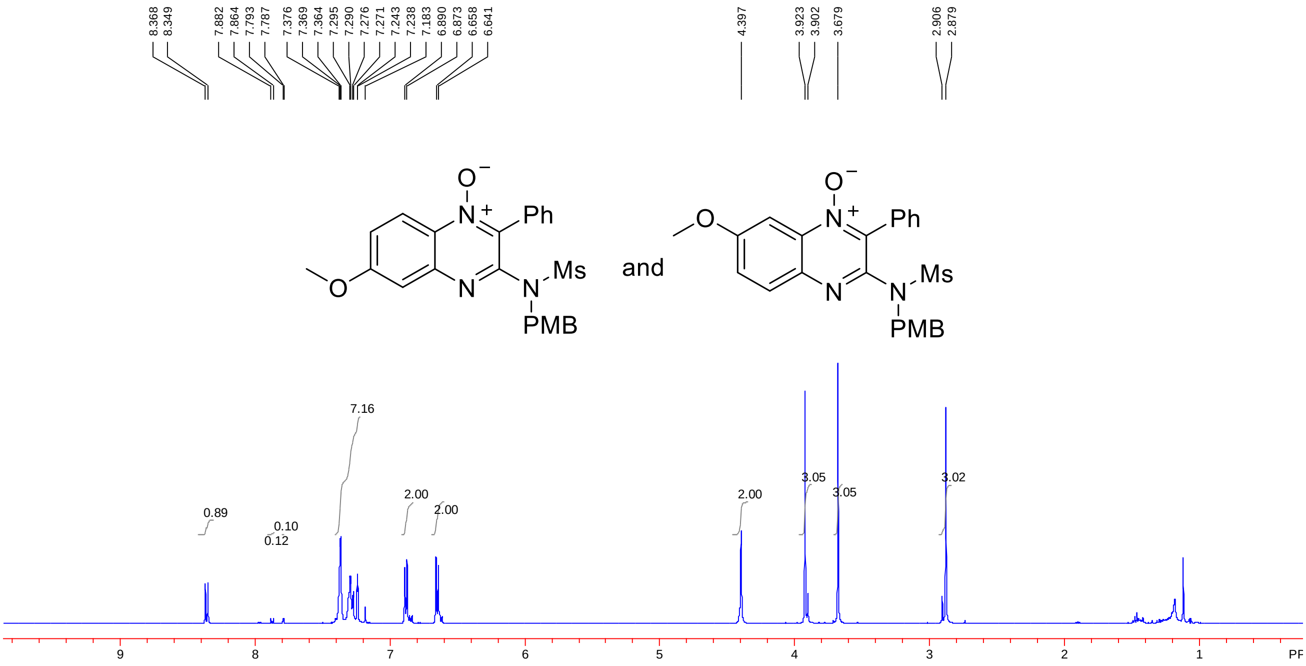

Supplementary Figure 72. ^{1}H NMR of compound **4b**

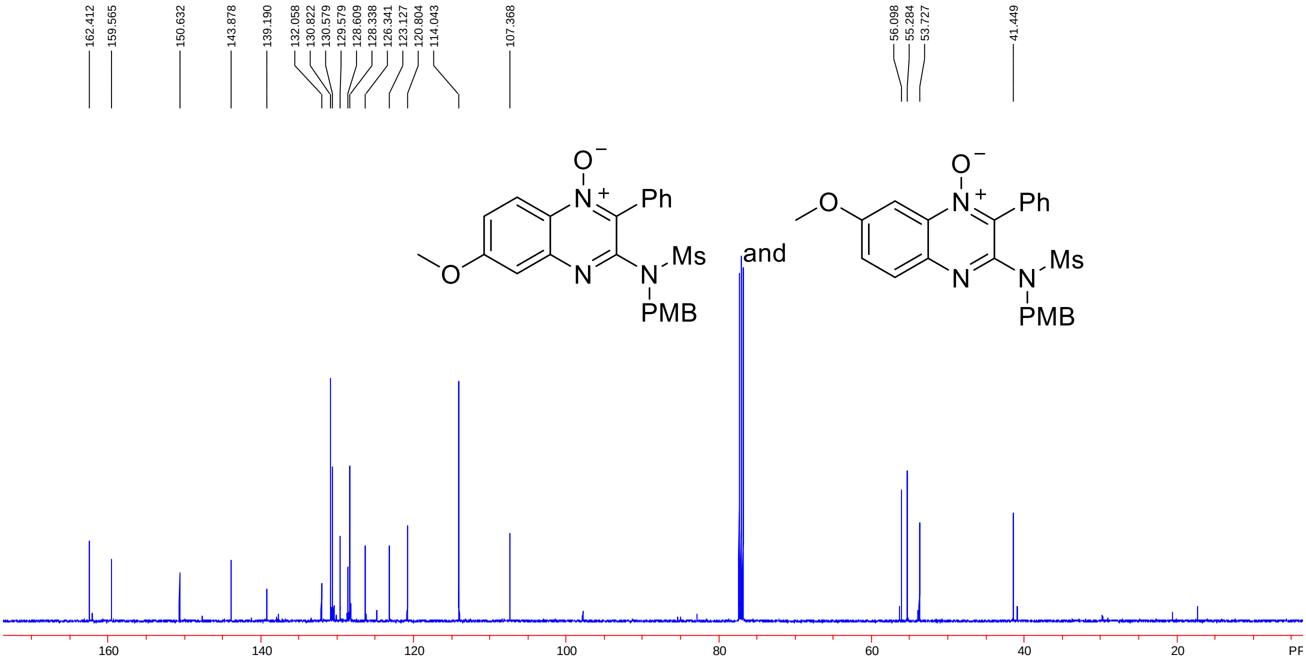

Supplementary Figure 73. ^{13}C NMR of compound **4b**

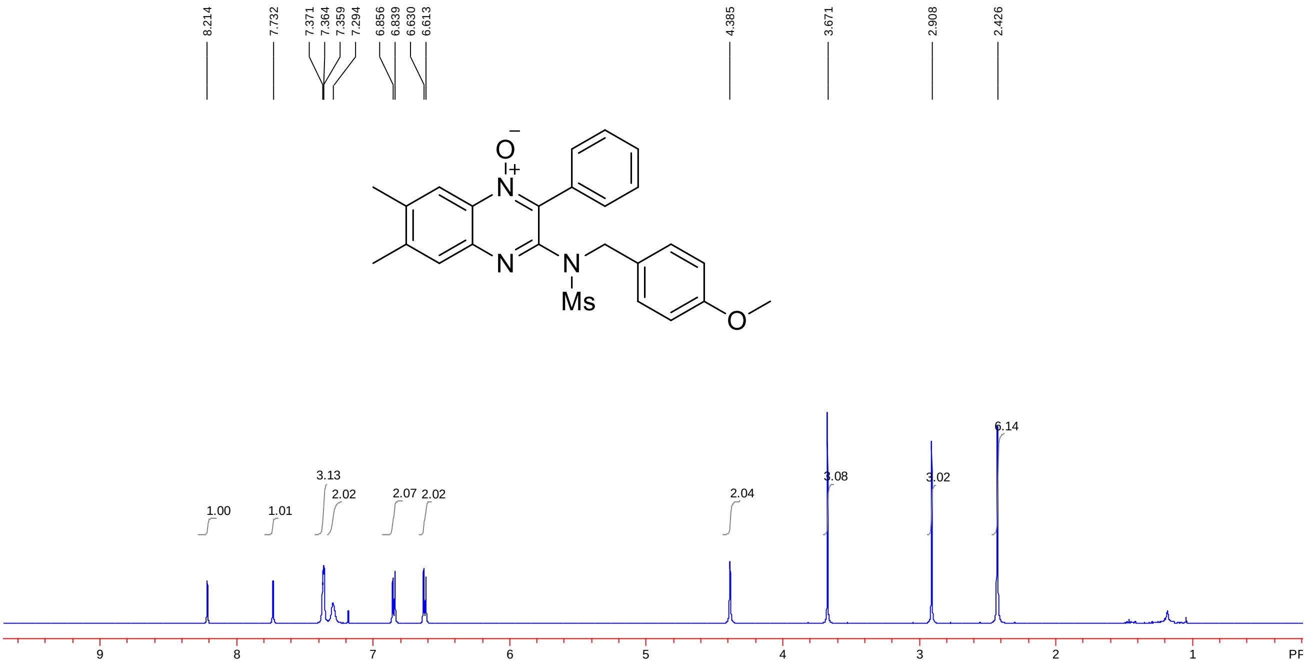

Supplementary Figure 74. ^{1}H NMR of compound **4c**

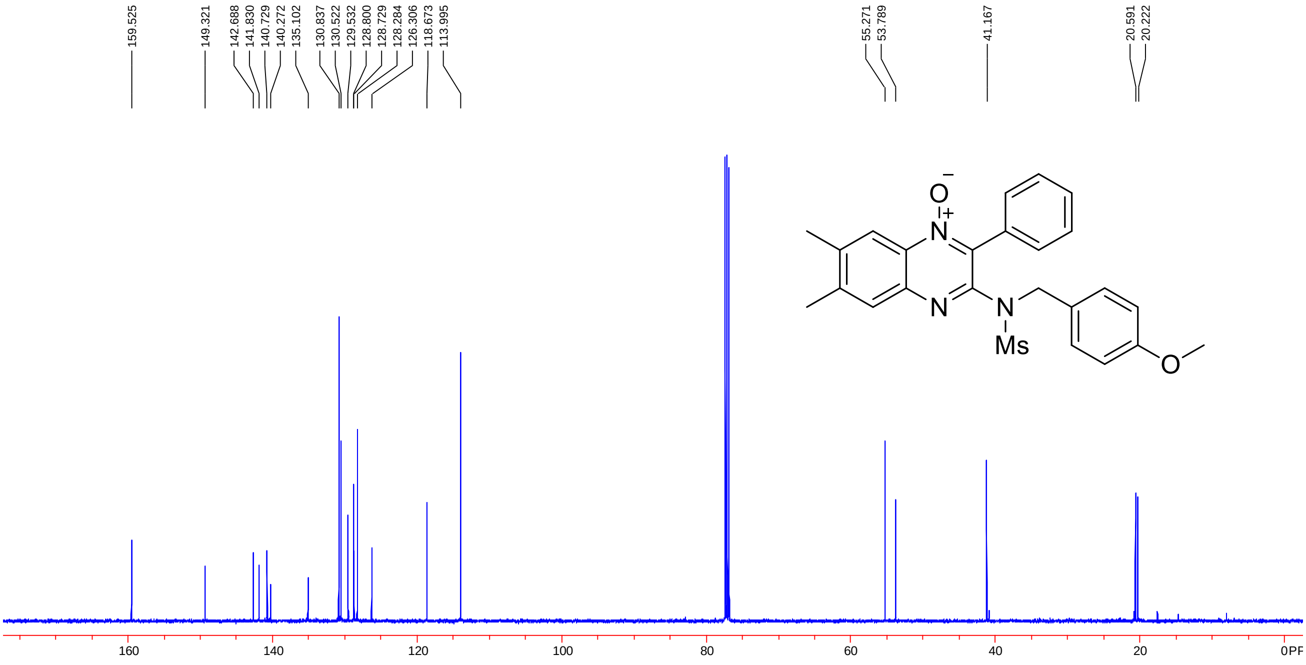

Supplementary Figure 75. ^{13}C NMR of compound **4c**

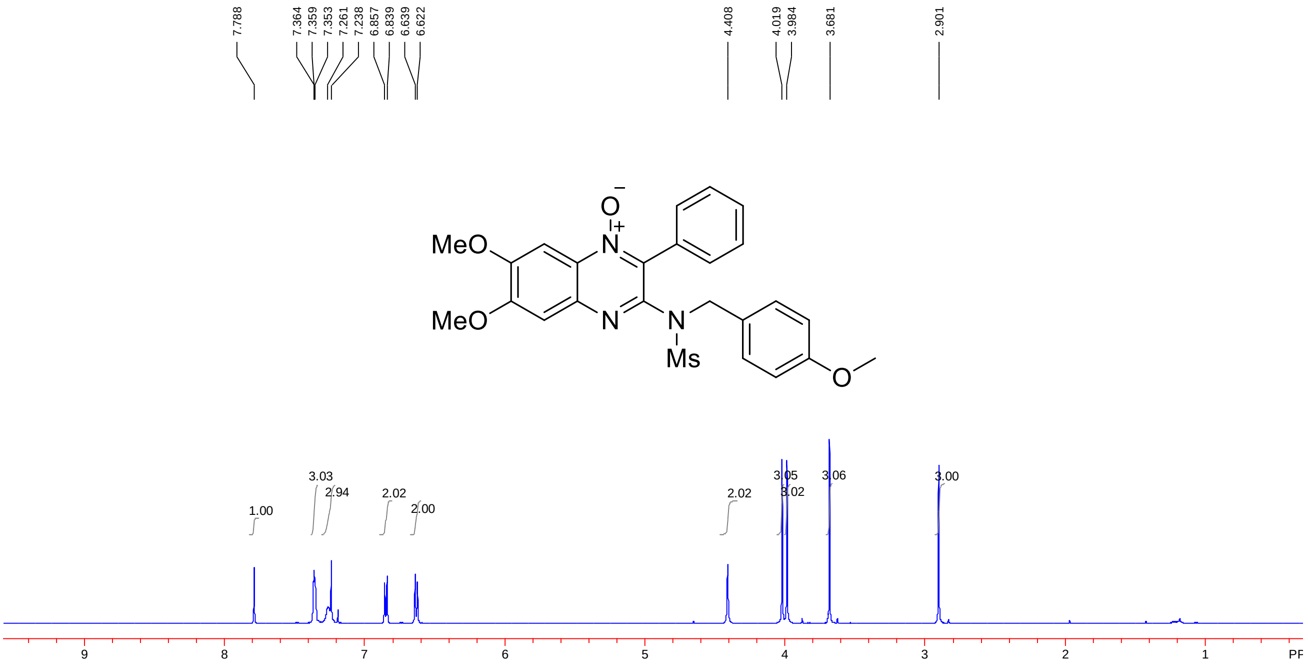

Supplementary Figure 76. ^{1}H NMR of compound **4d**

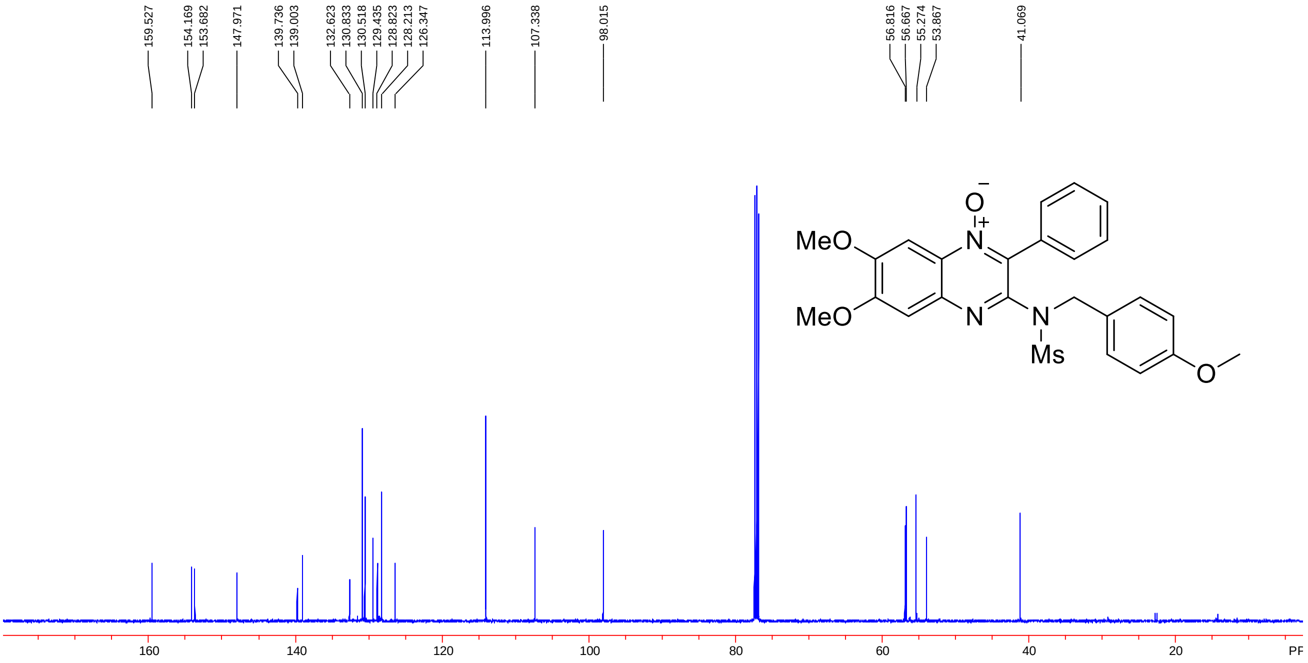

Supplementary Figure 77. ^{13}C NMR of compound **4d**

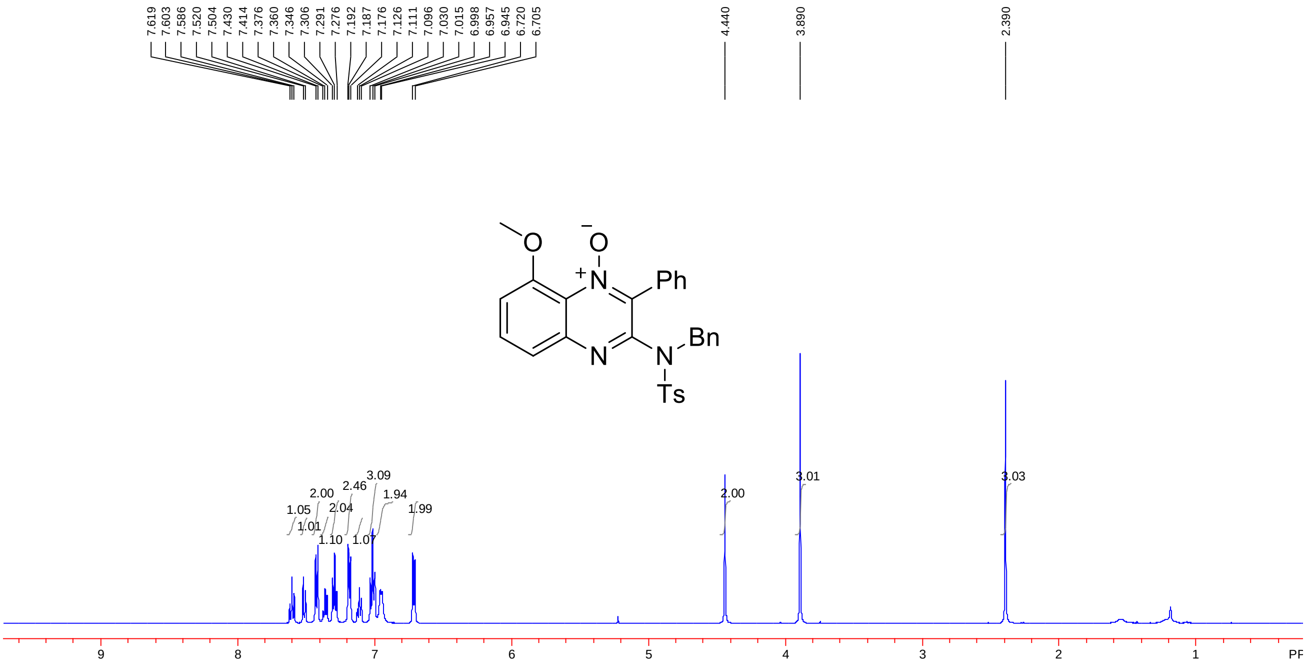

Supplementary Figure 78. ^{1}H NMR of compound **4e**

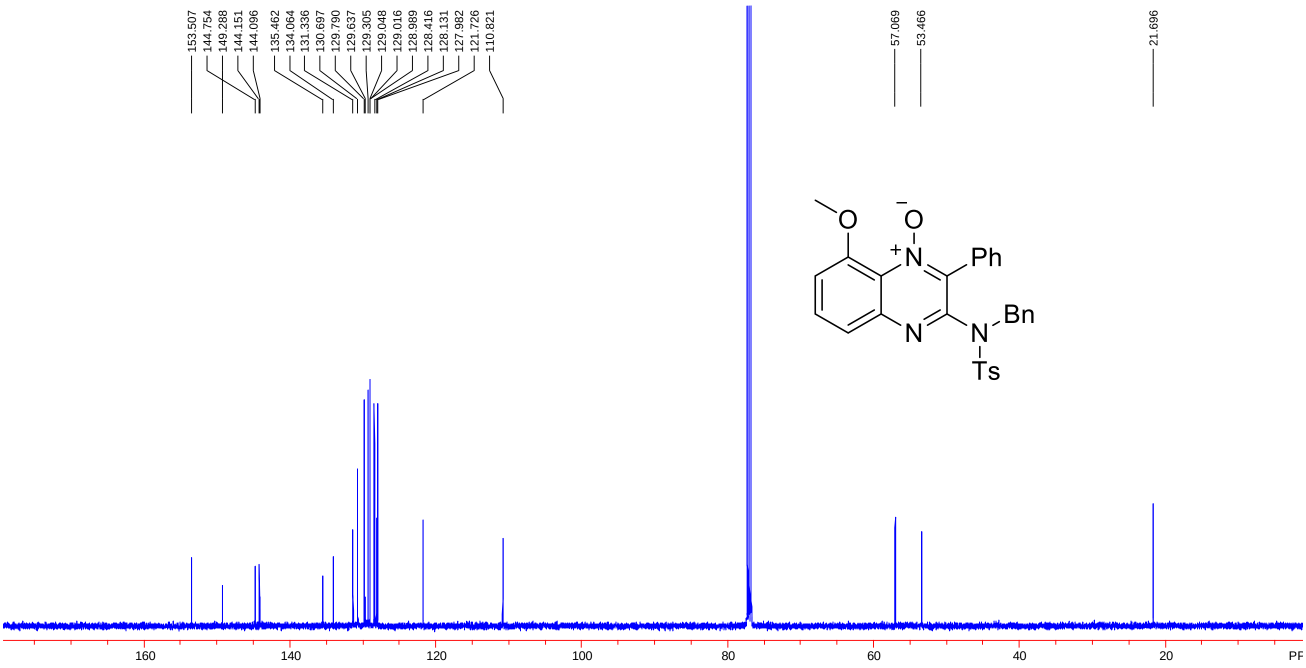

Supplementary Figure 79. ^{13}C NMR of compound **4e**

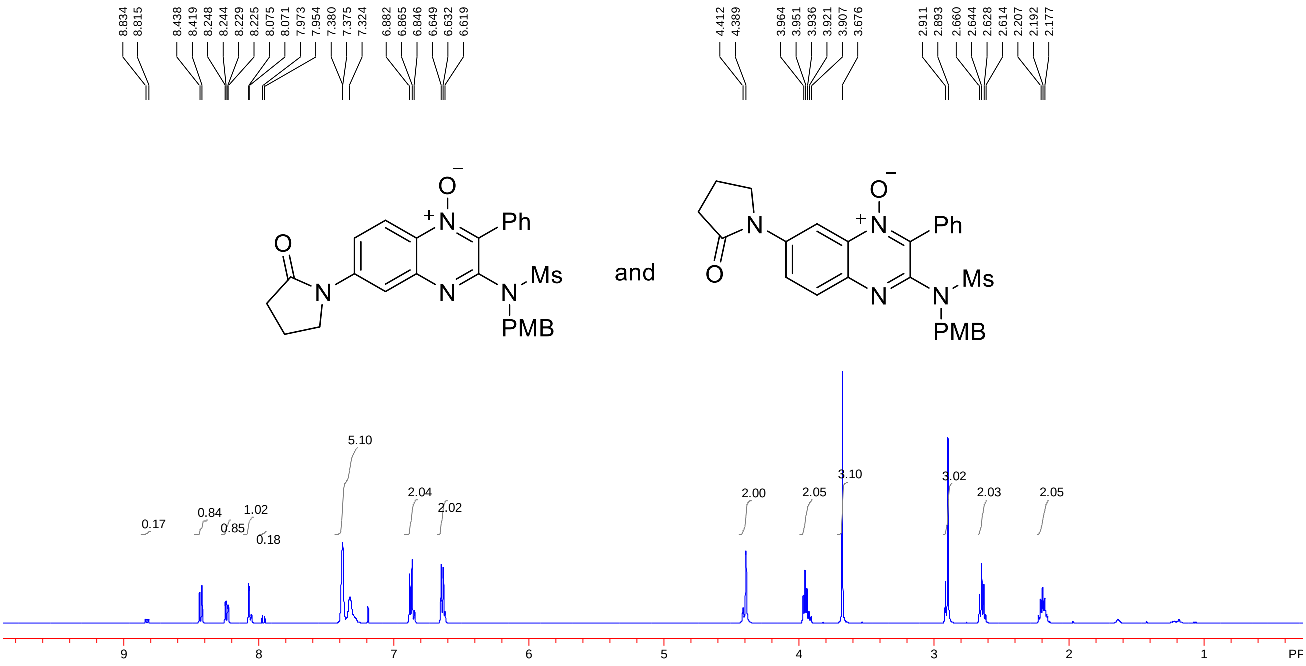

Supplementary Figure 80. ^{1}H NMR of compound **4f**

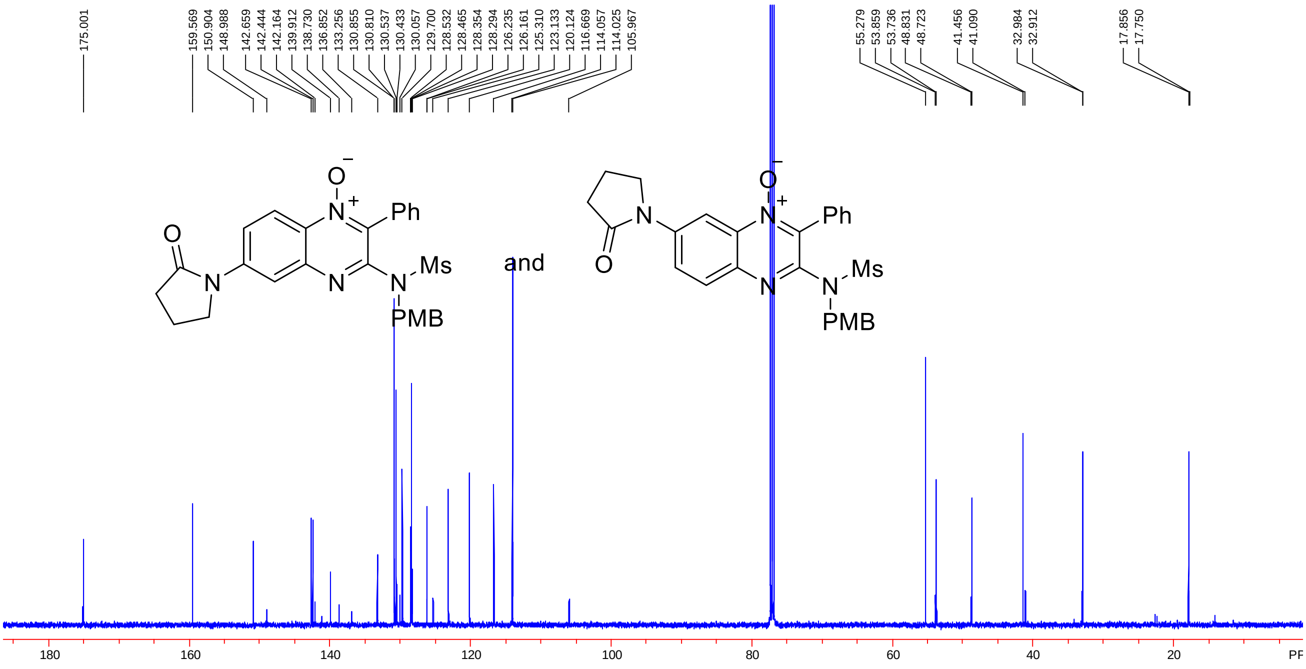

Supplementary Figure 81. ^{13}C NMR of compound **4f**

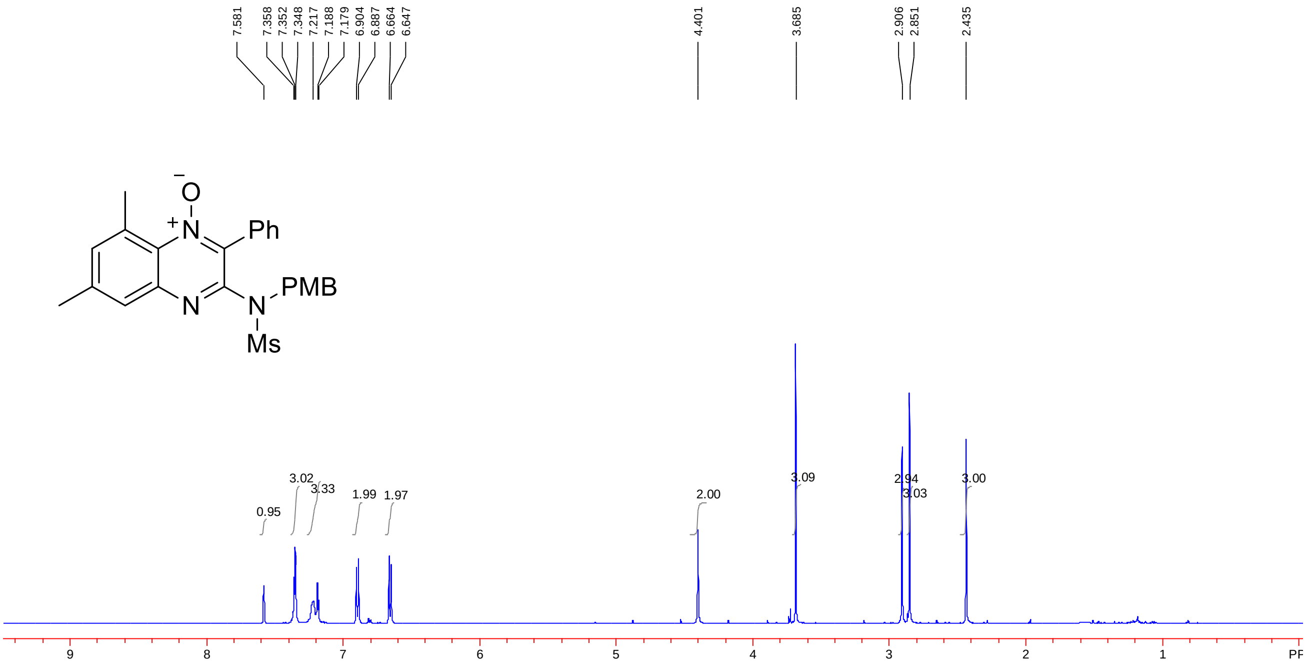

Supplementary Figure 82. ^{1}H NMR of compound **4g**

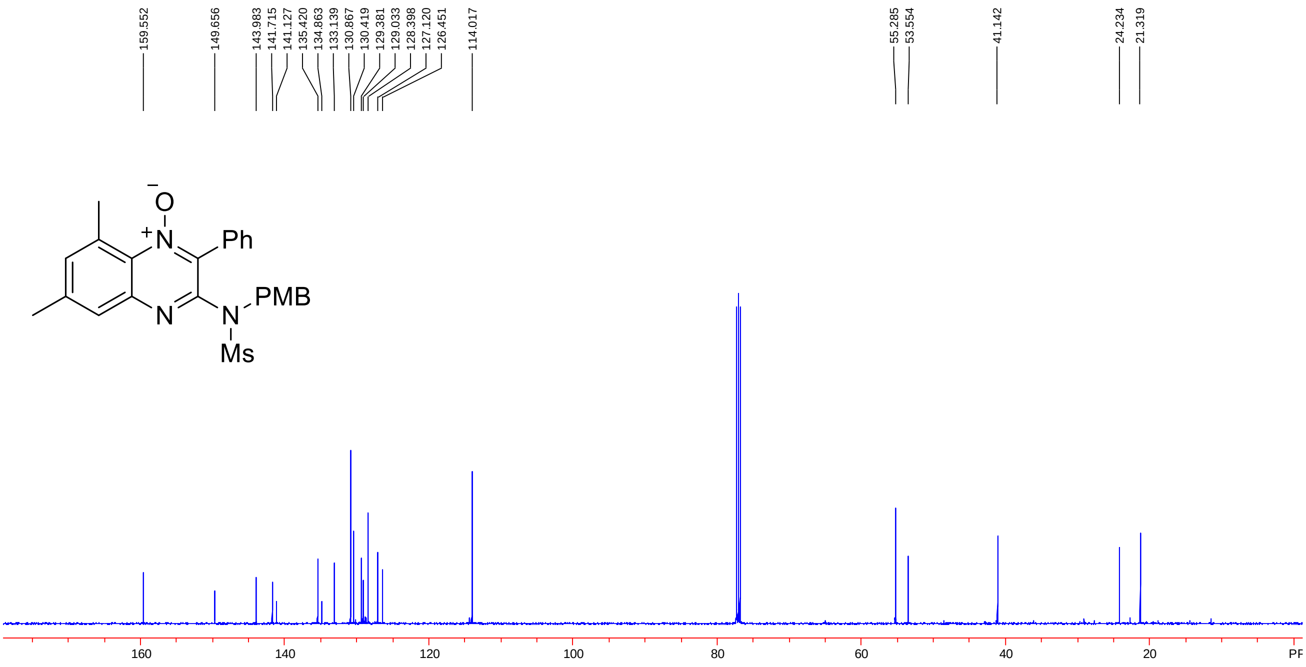

Supplementary Figure 83. ^{13}C NMR of compound **4g**

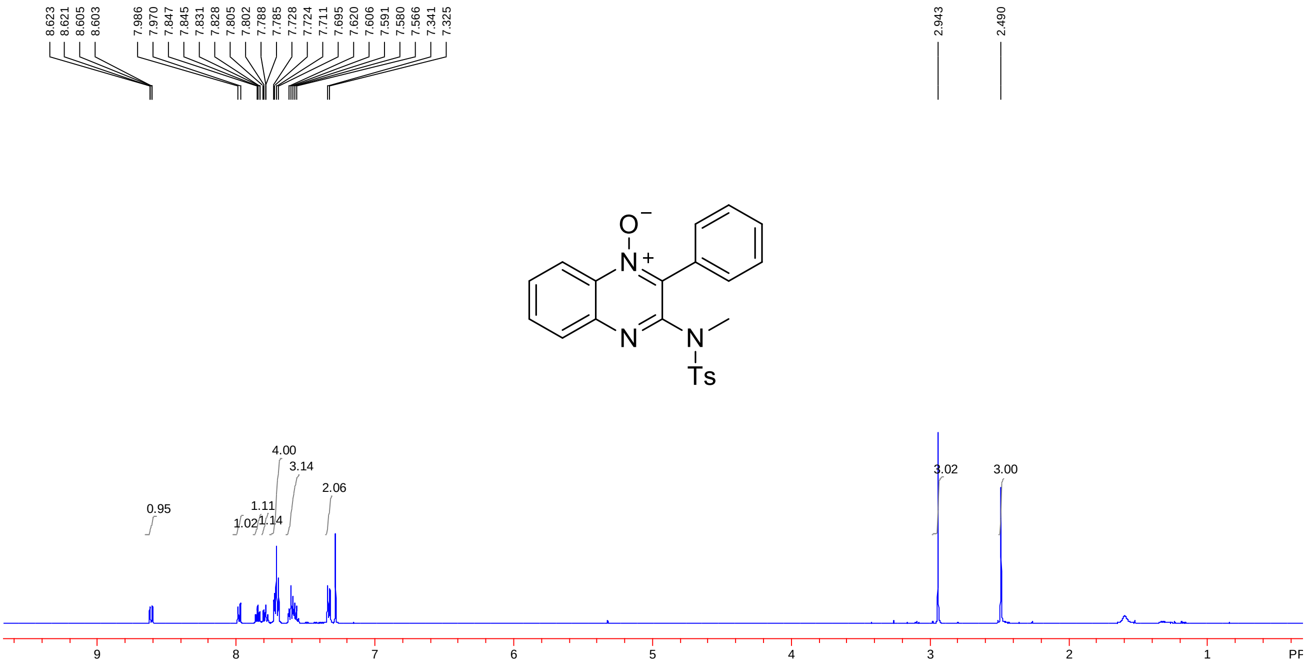

Supplementary Figure 84. ^{1}H NMR of compound **4h**

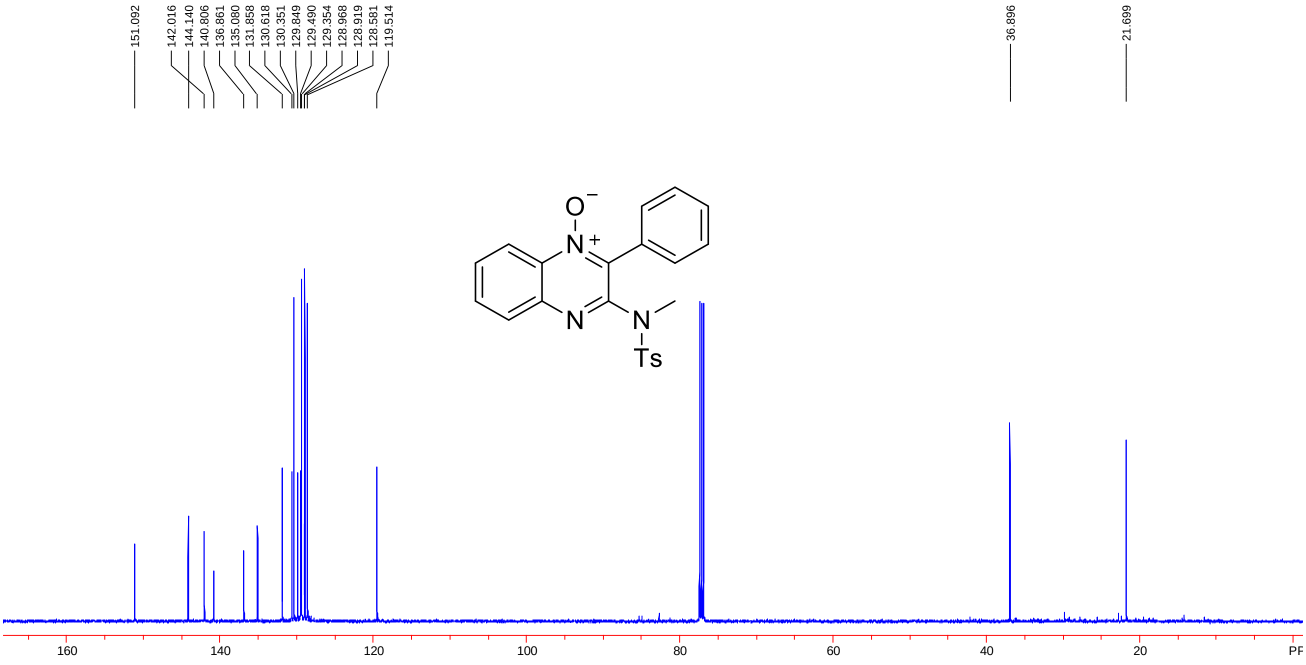

Supplementary Figure 85. ^{13}C NMR of compound **4h**

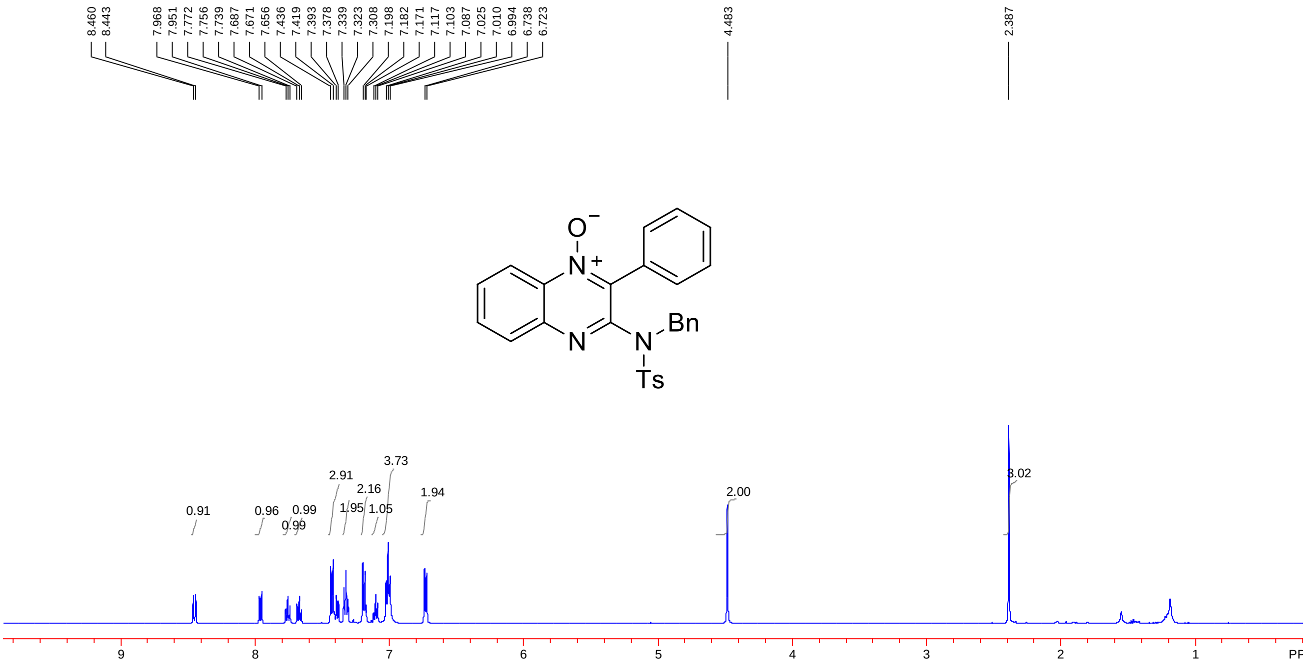

Supplementary Figure 86. ^{1}H NMR of compound **4i**

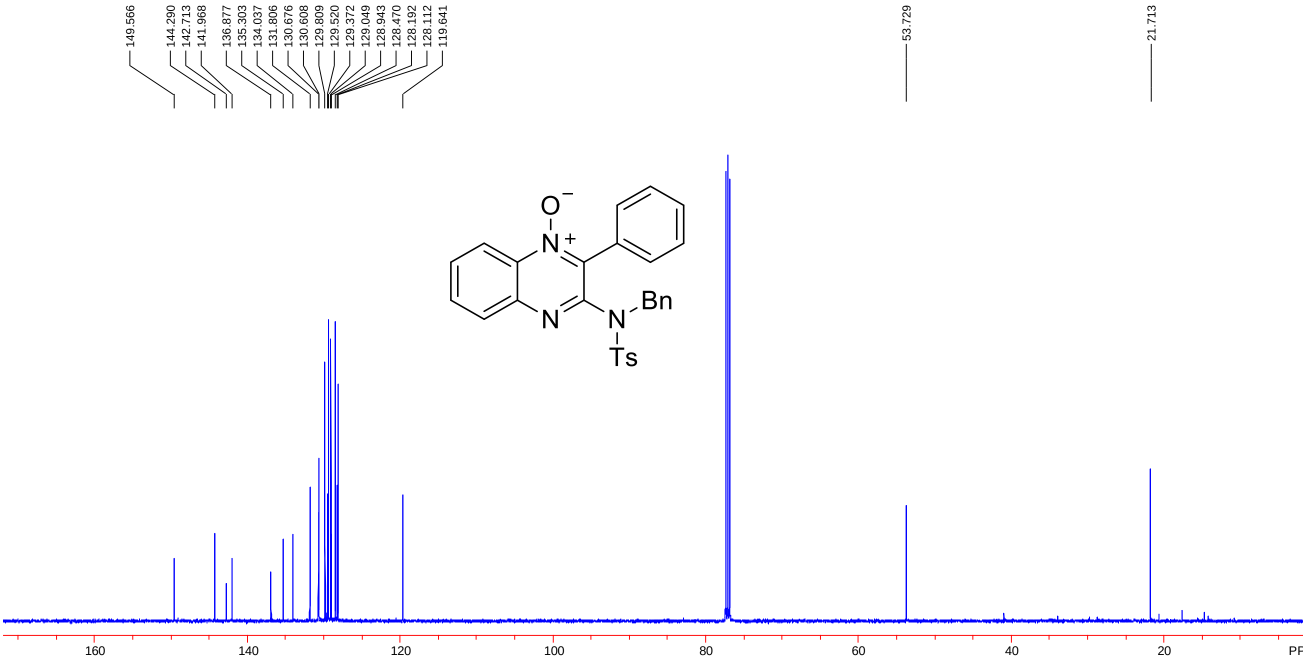

Supplementary Figure 87. ^{13}C NMR of compound **4i**

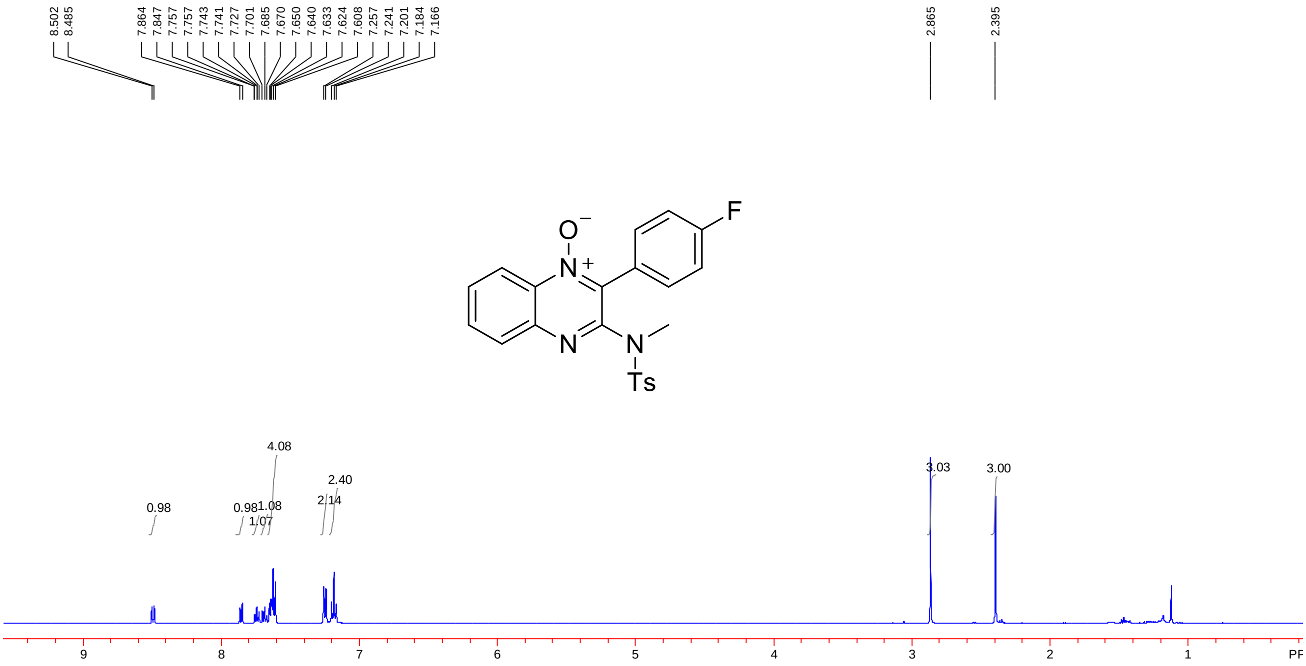

Supplementary Figure 88. ^{1}H NMR of compound **4j**

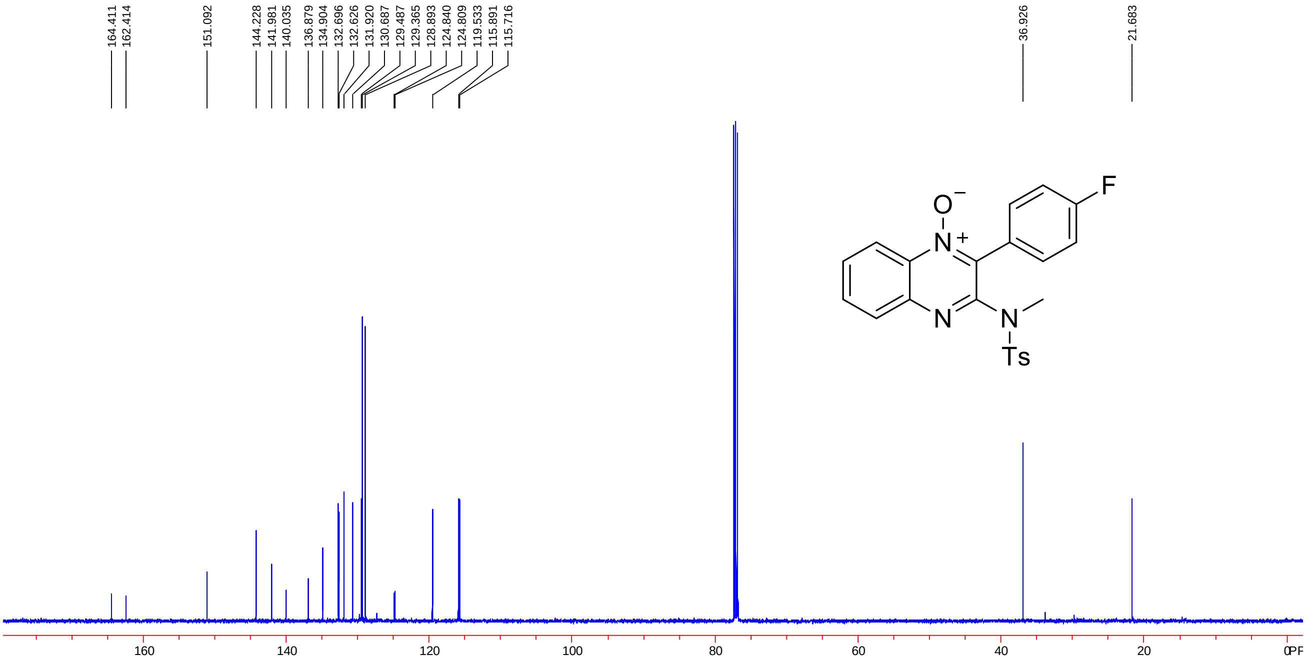

Supplementary Figure 89. ^{13}C NMR of compound **4j**

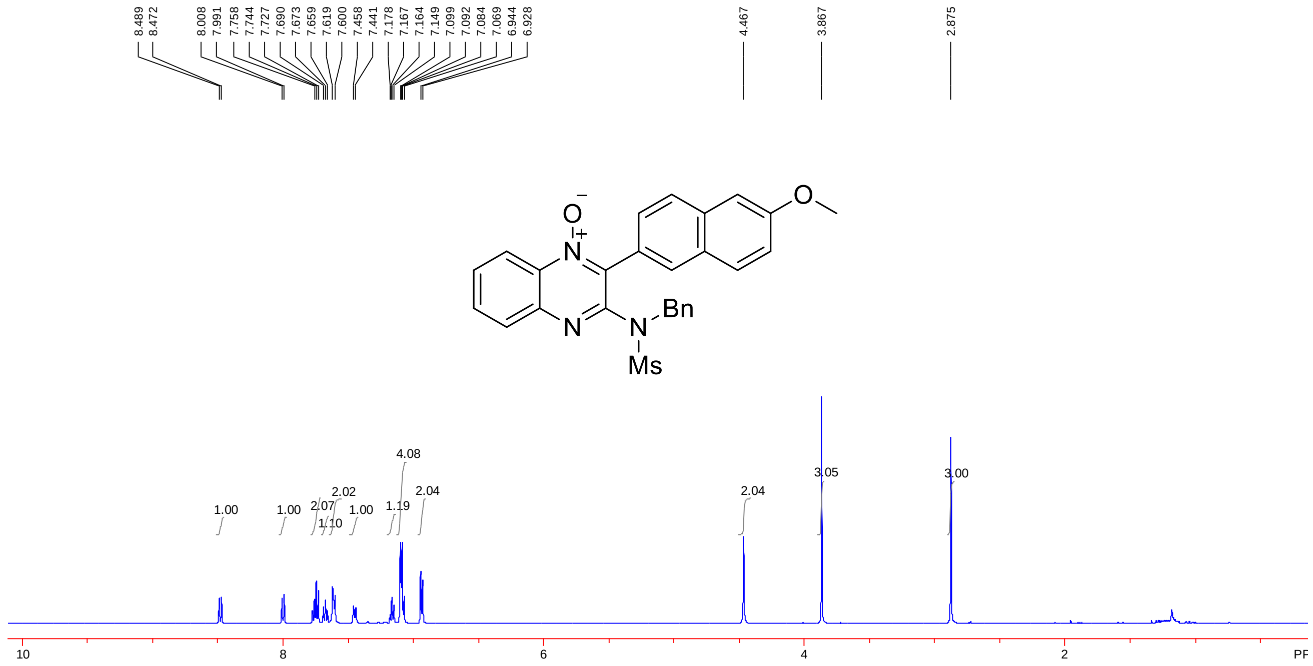

Supplementary Figure 90. ^{1}H NMR of compound **4k**

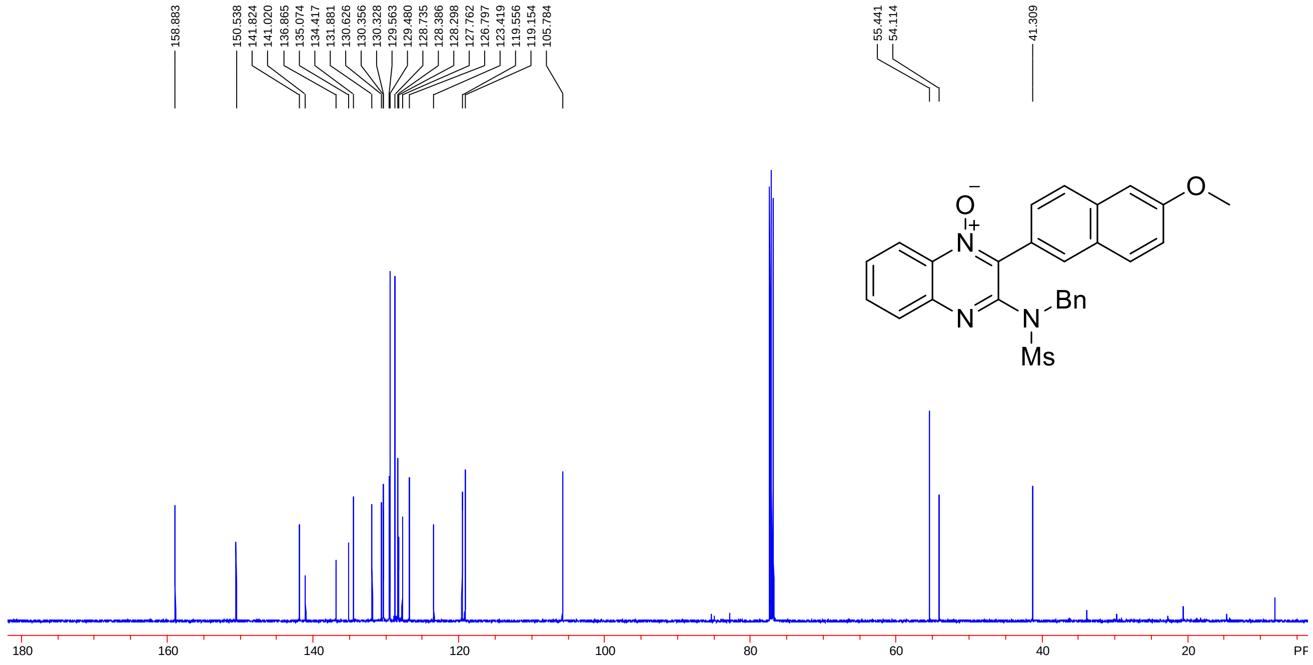

Supplementary Figure 91. ^{13}C NMR of compound **4k**

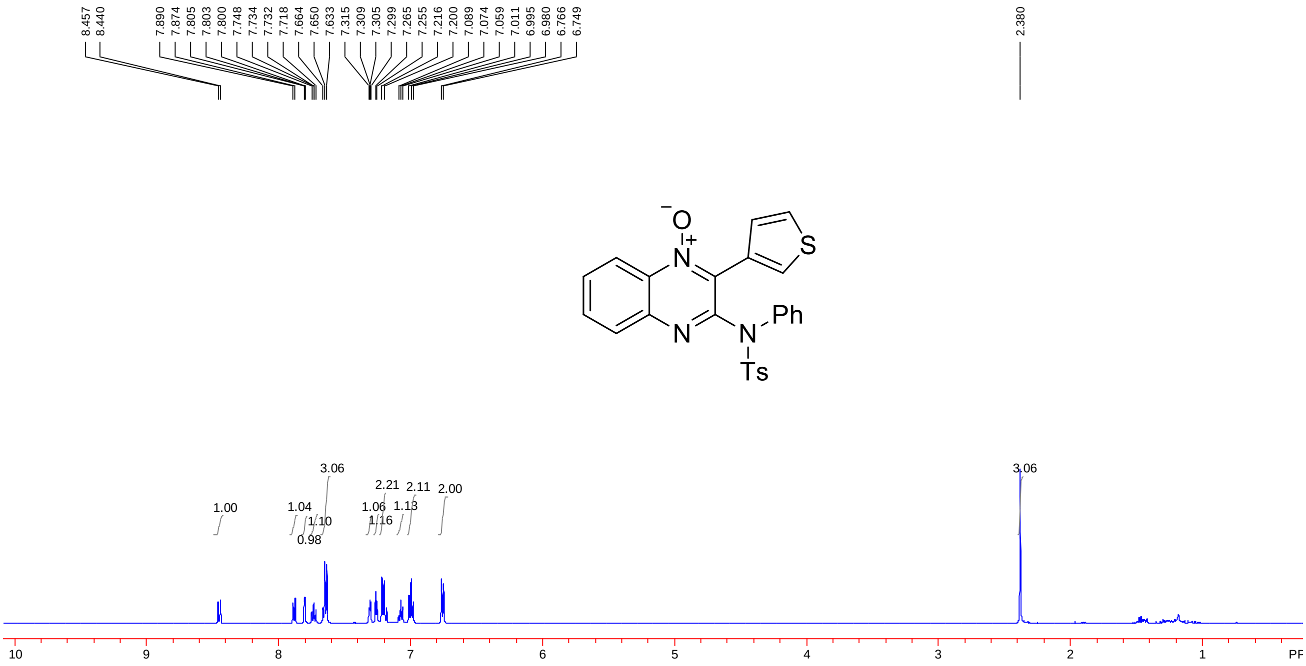

Supplementary Figure 92. ^{1}H NMR of compound **4l**

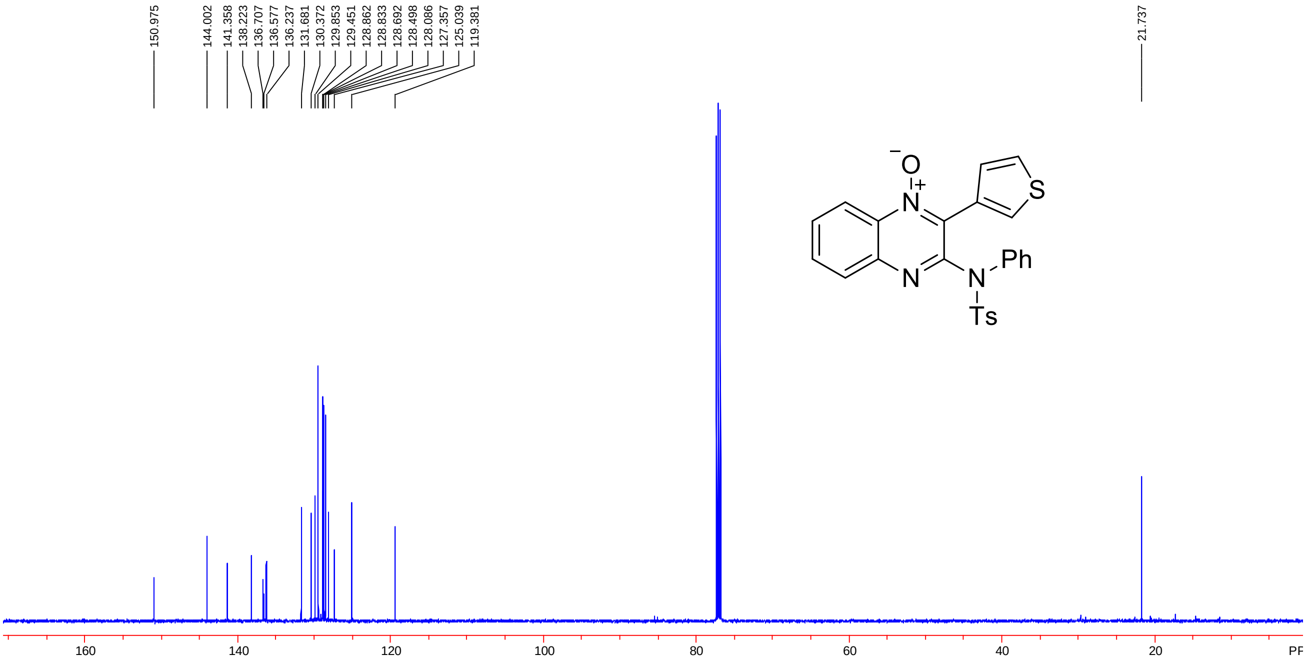

Supplementary Figure 93. ^{13}C NMR of compound **4l**

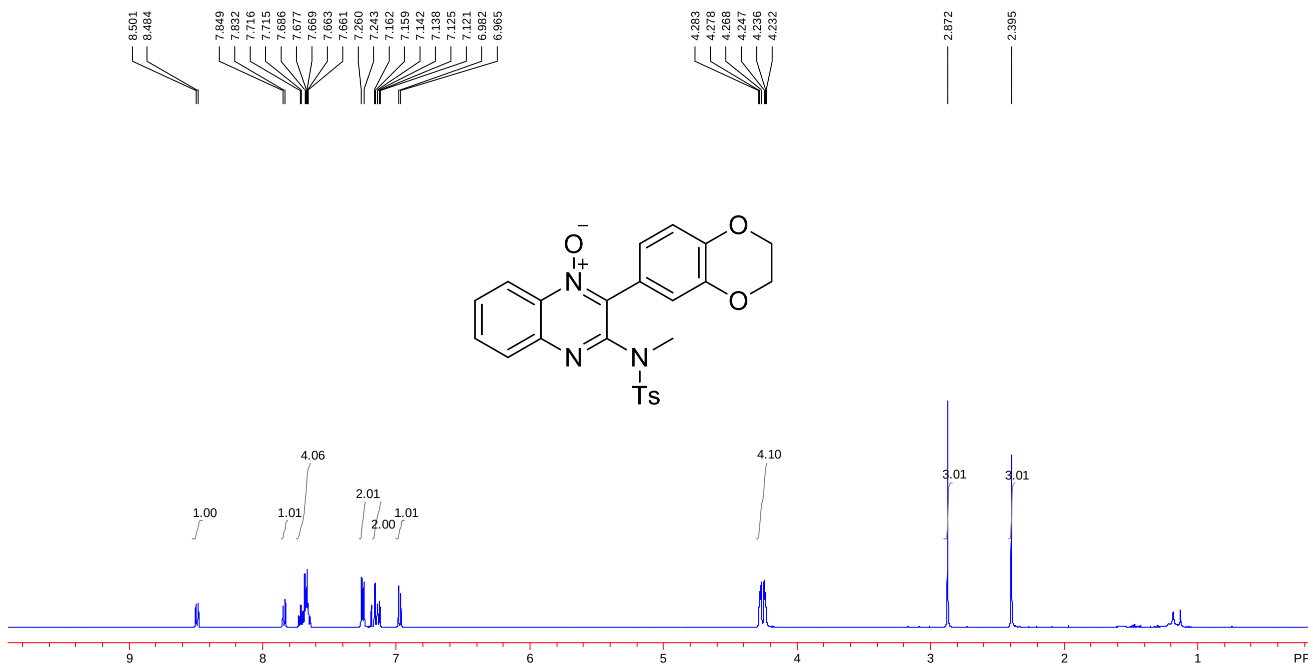

Supplementary Figure 94. ^{1}H NMR of compound **4m**

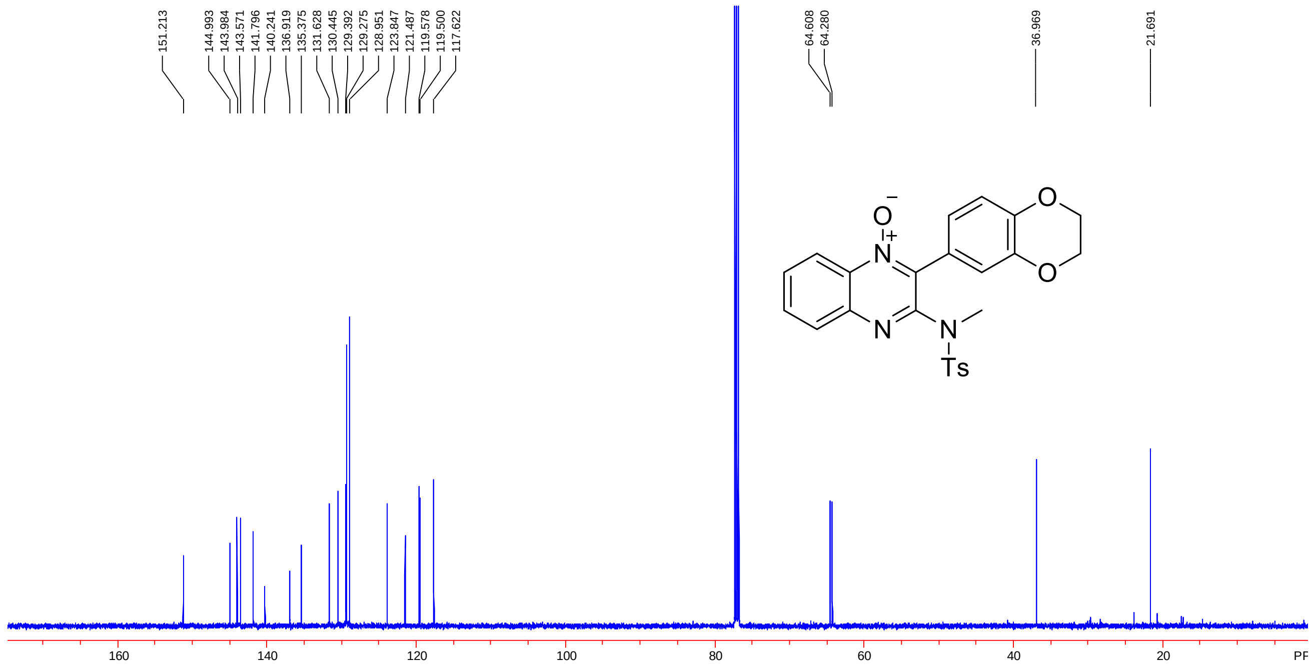

Supplementary Figure 95. ^{13}C NMR of compound **4m**

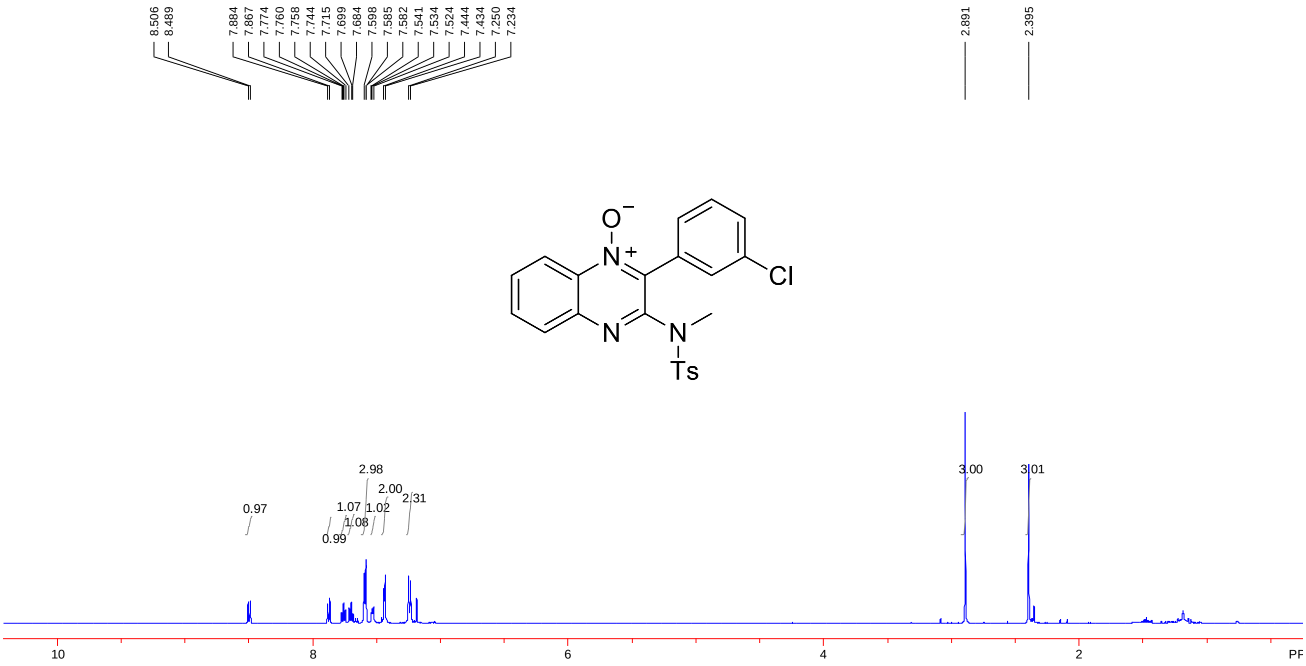

Supplementary Figure 96. ^{1}H NMR of compound **4n**

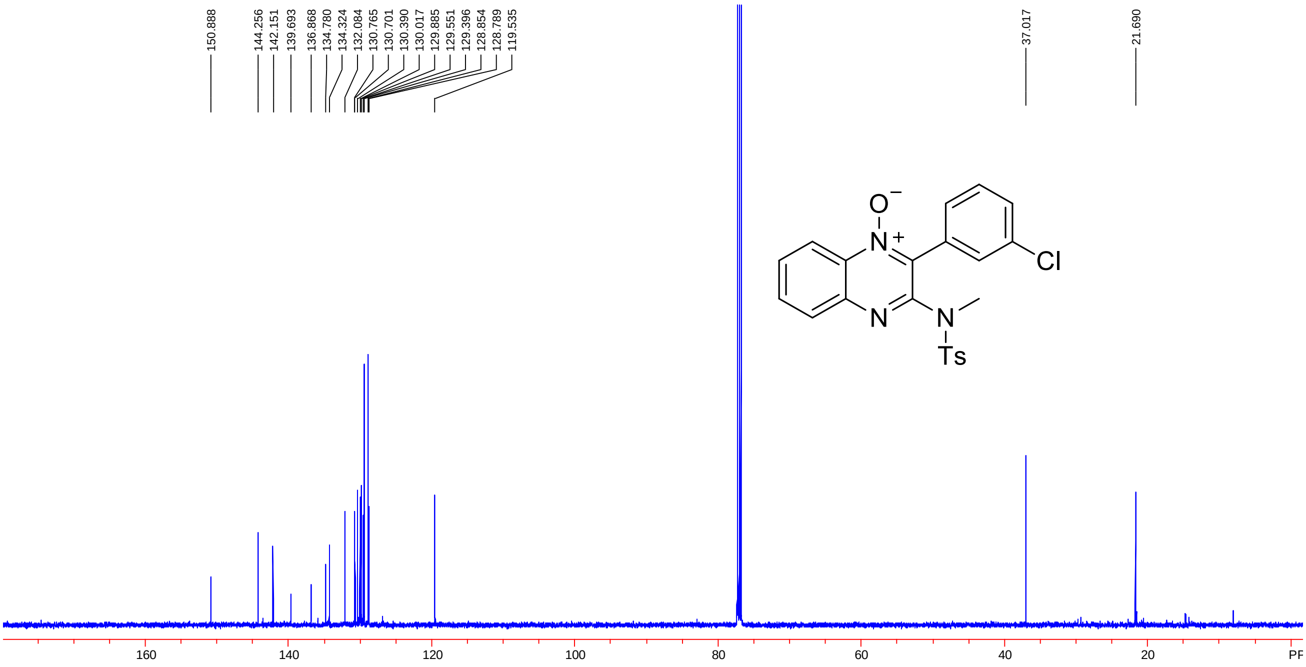

Supplementary Figure 97. ^{13}C NMR of compound **4n**

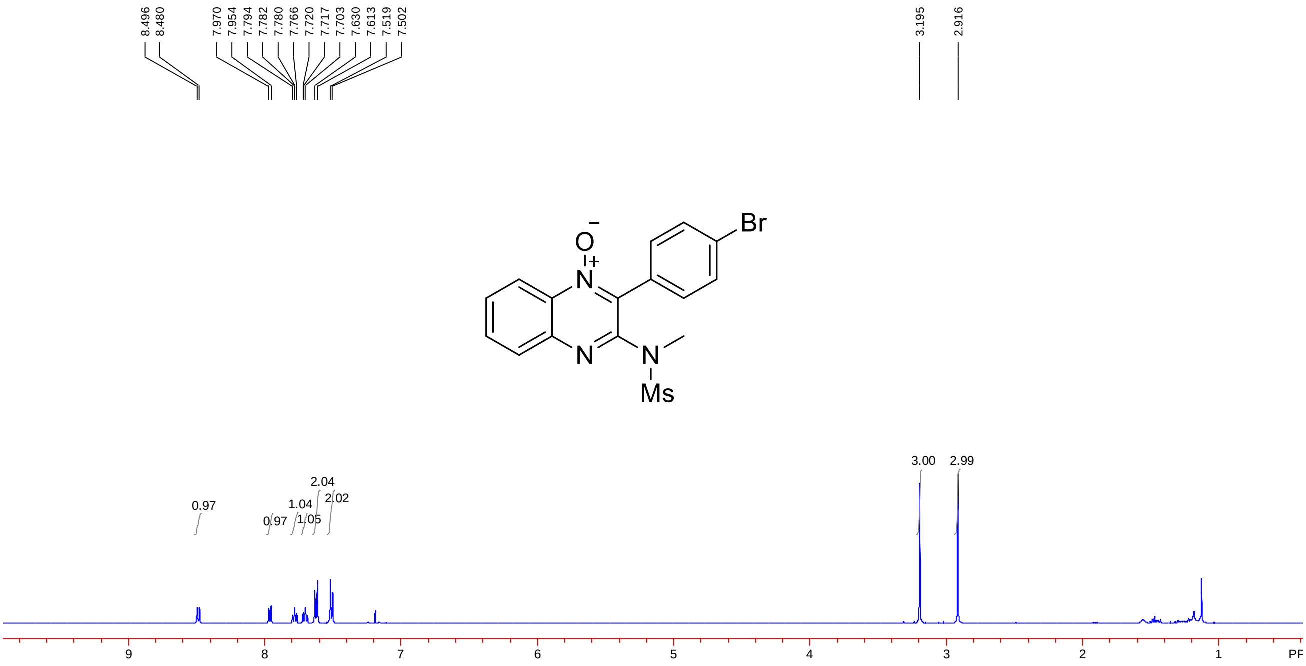

Supplementary Figure 98. ^{1}H NMR of compound **4o**

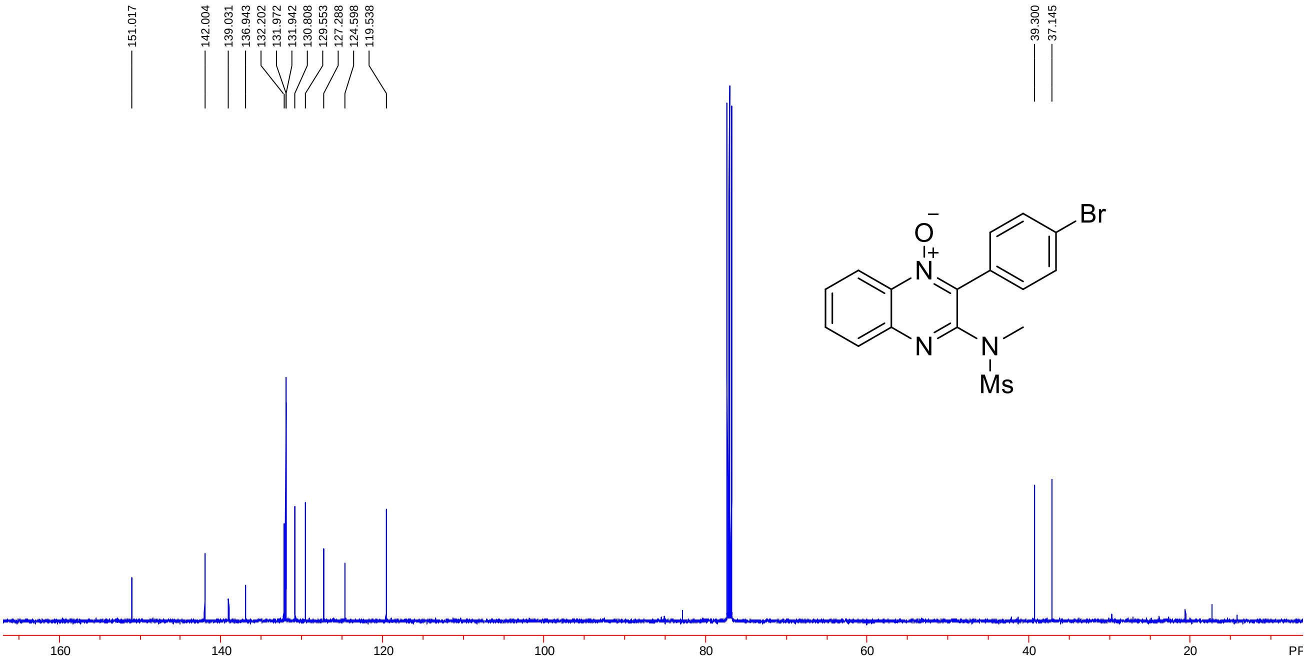

Supplementary Figure 99. ^{13}C NMR of compound **4o**

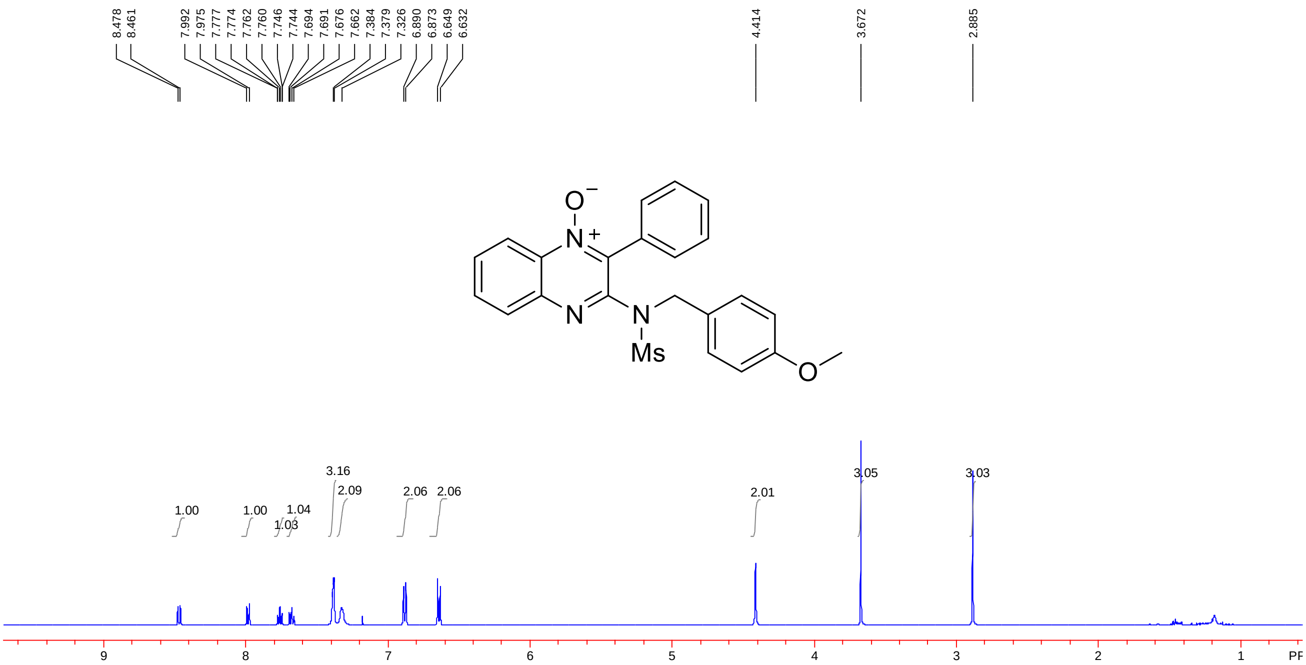

Supplementary Figure 100. ^{1}H NMR of compound **4p**

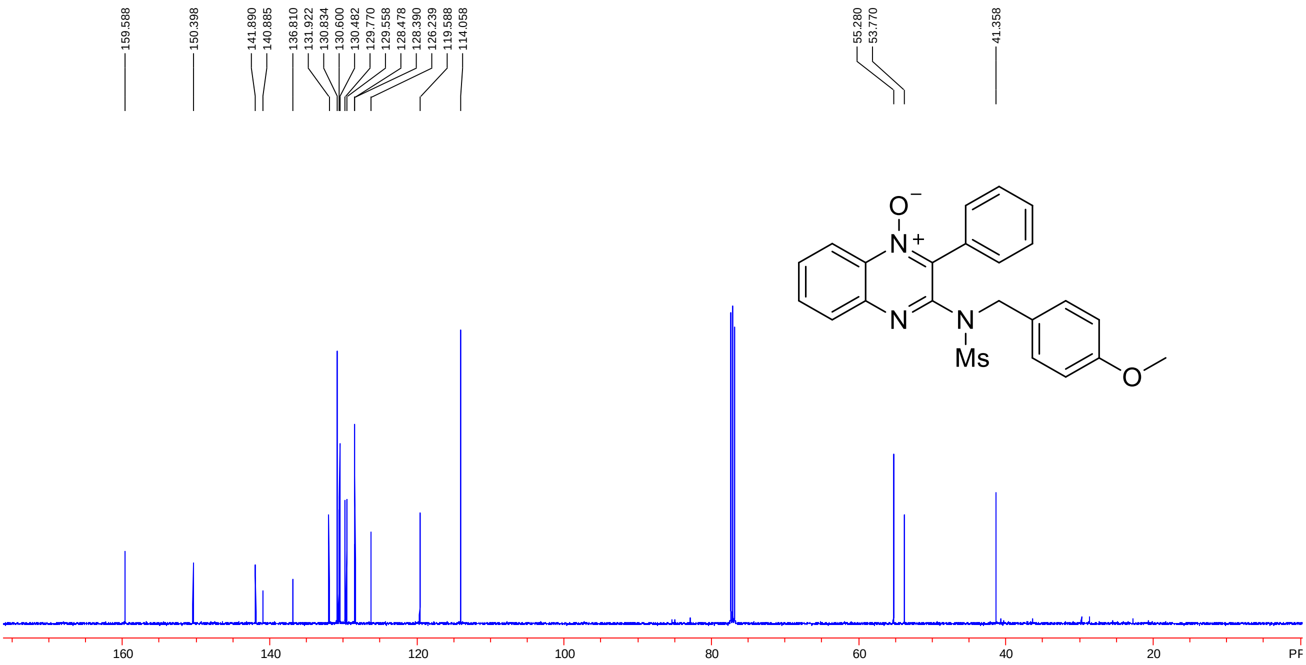

Supplementary Figure 101. ^{13}C NMR of compound **4p**

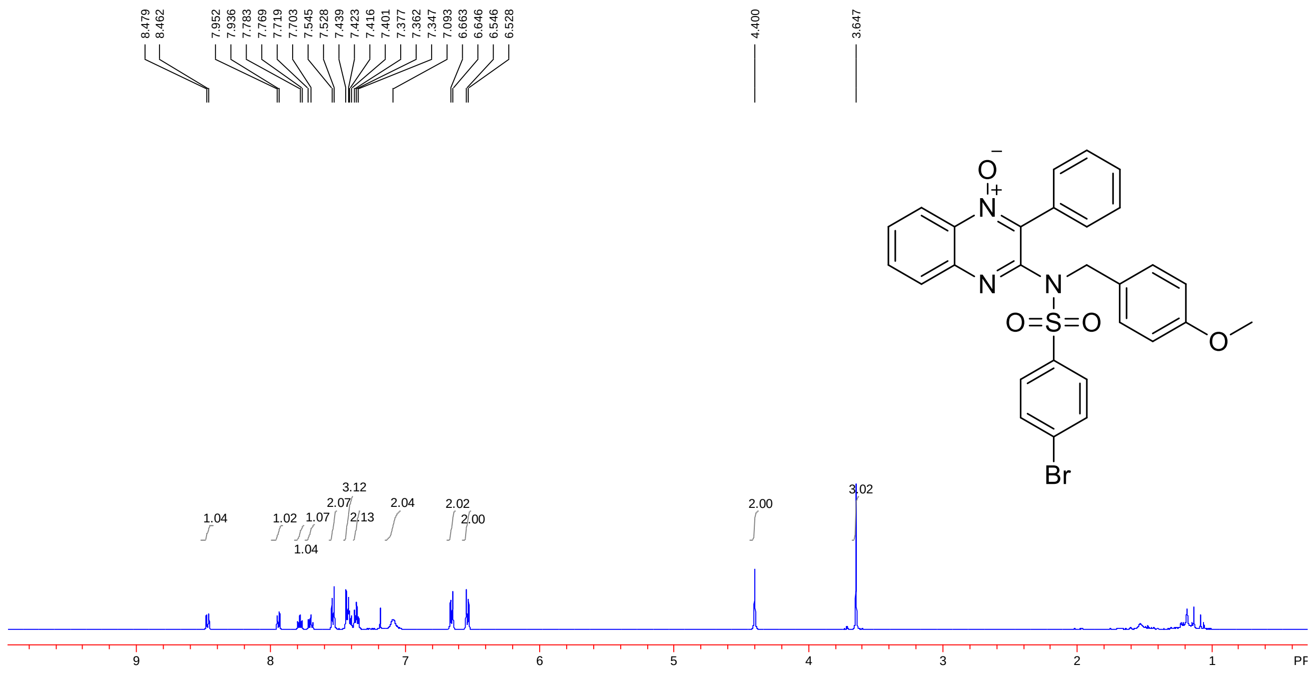

Supplementary Figure 102. ^{1}H NMR of compound **4q**

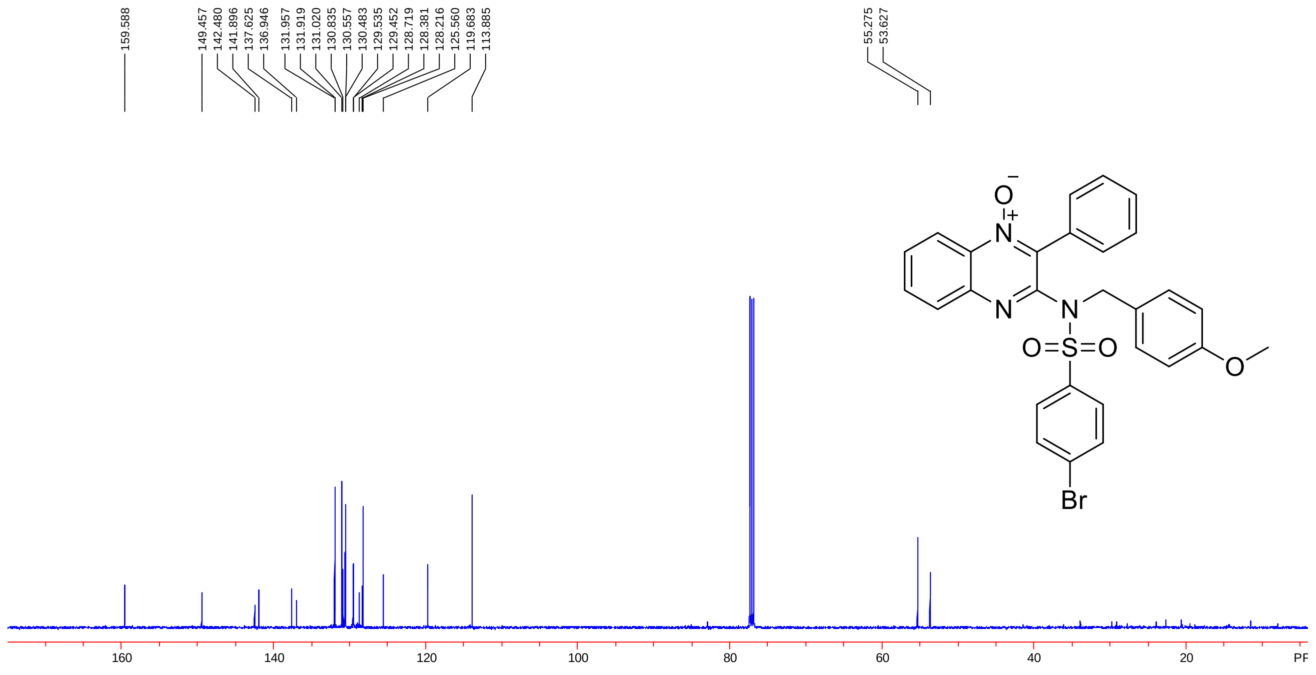

Supplementary Figure 103. ^{13}C NMR of compound **4q**

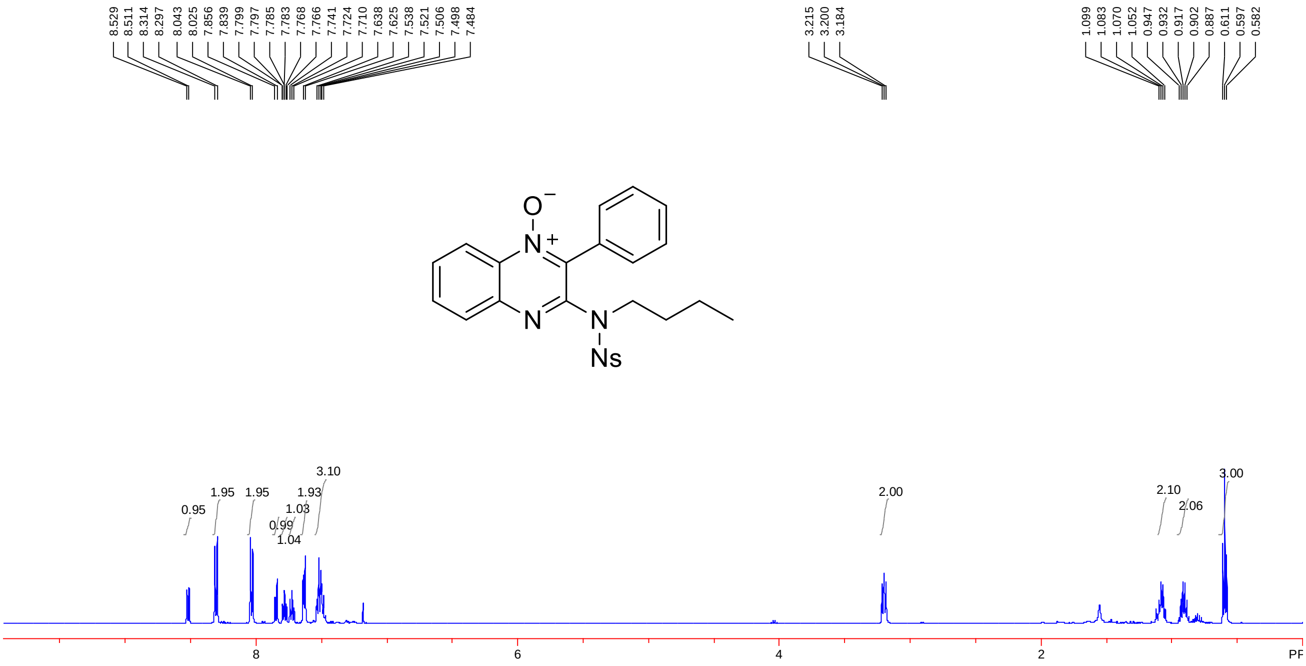

Supplementary Figure 104. ^{1}H NMR of compound **4r**

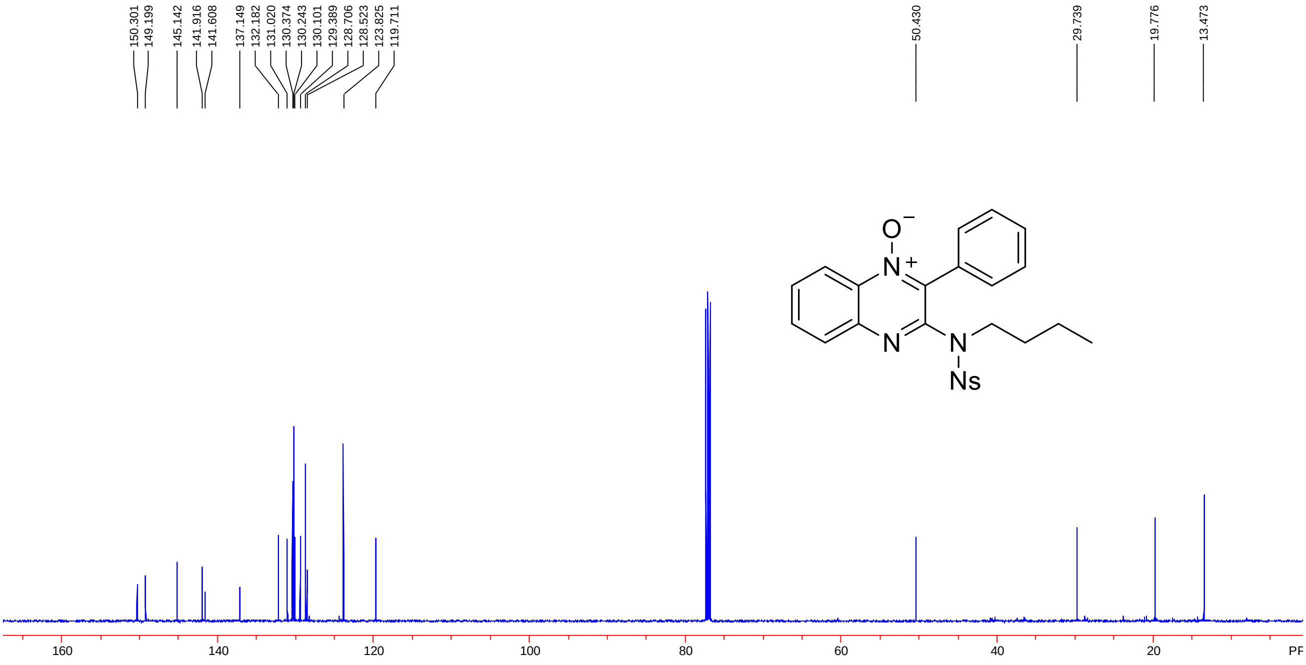

Supplementary Figure 105. ^{13}C NMR of compound **4r**

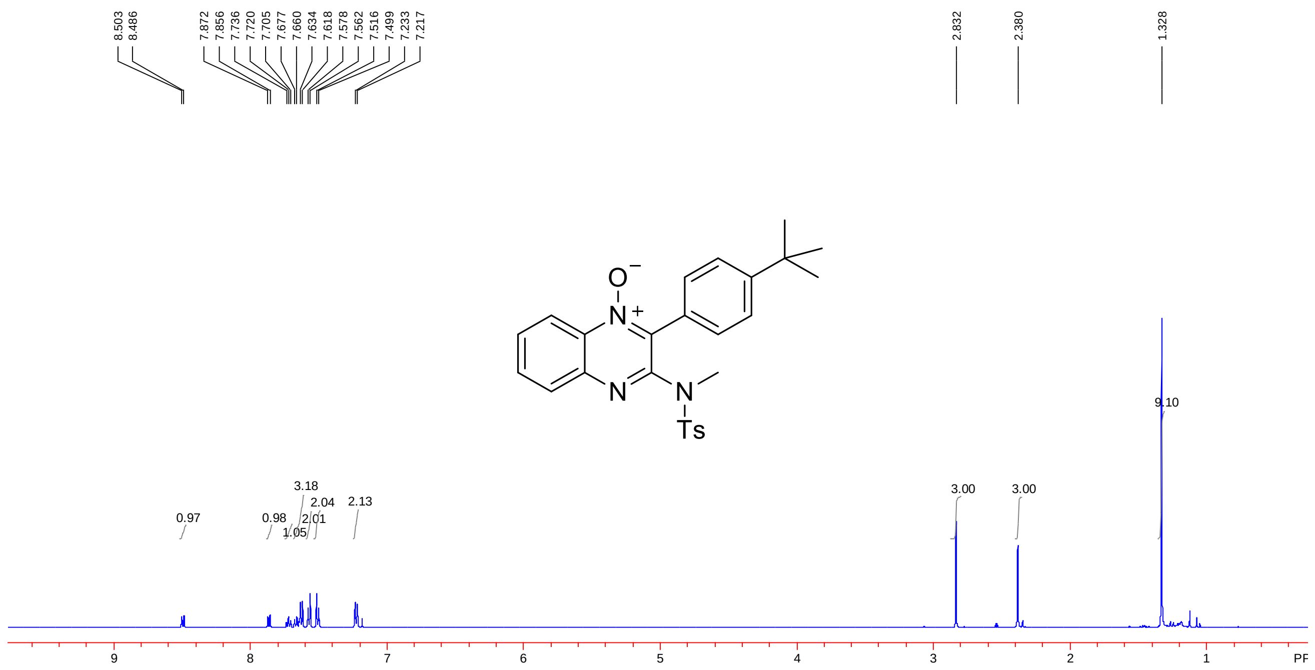

Supplementary Figure 106. ^{1}H NMR of compound **4s**

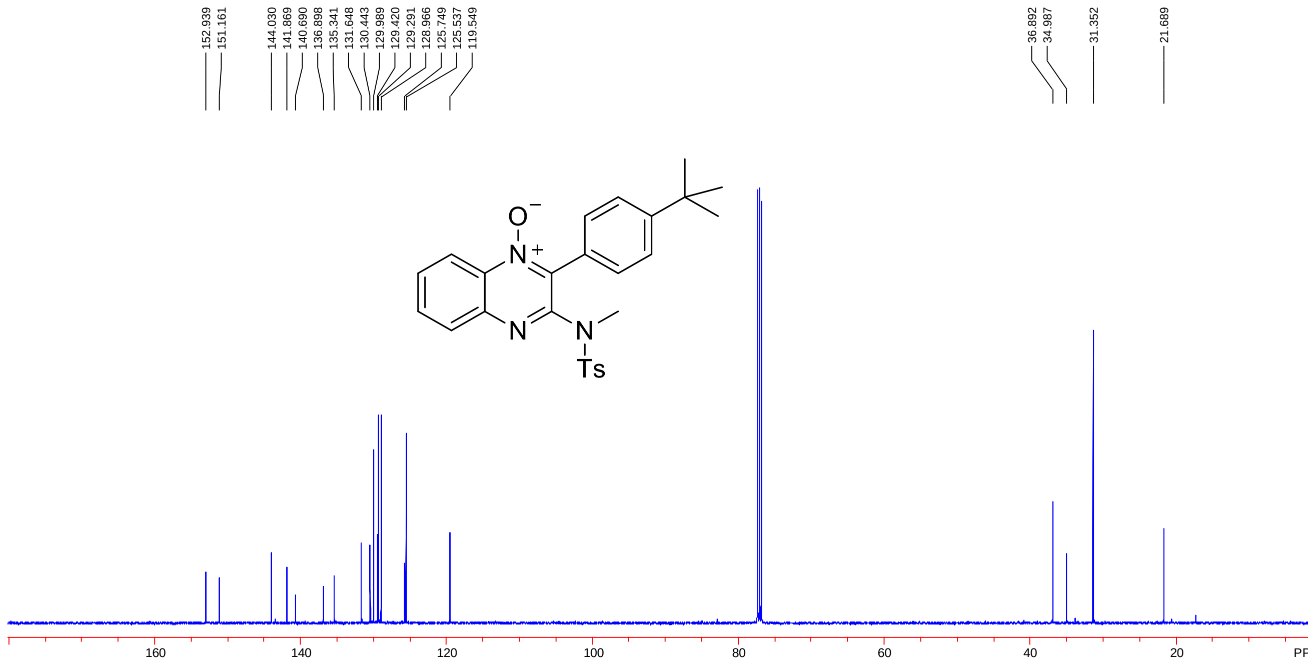

Supplementary Figure 107. ^{13}C NMR of compound **4s**

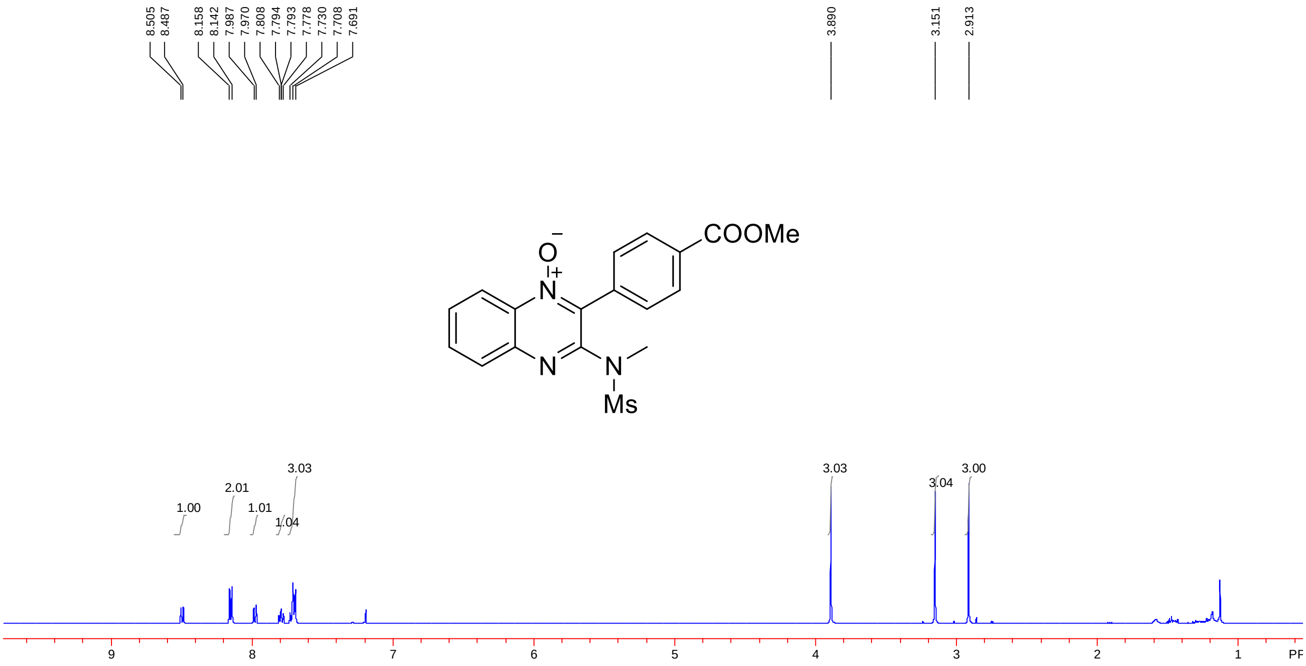

Supplementary Figure 108. ^{1}H NMR of compound **4t**

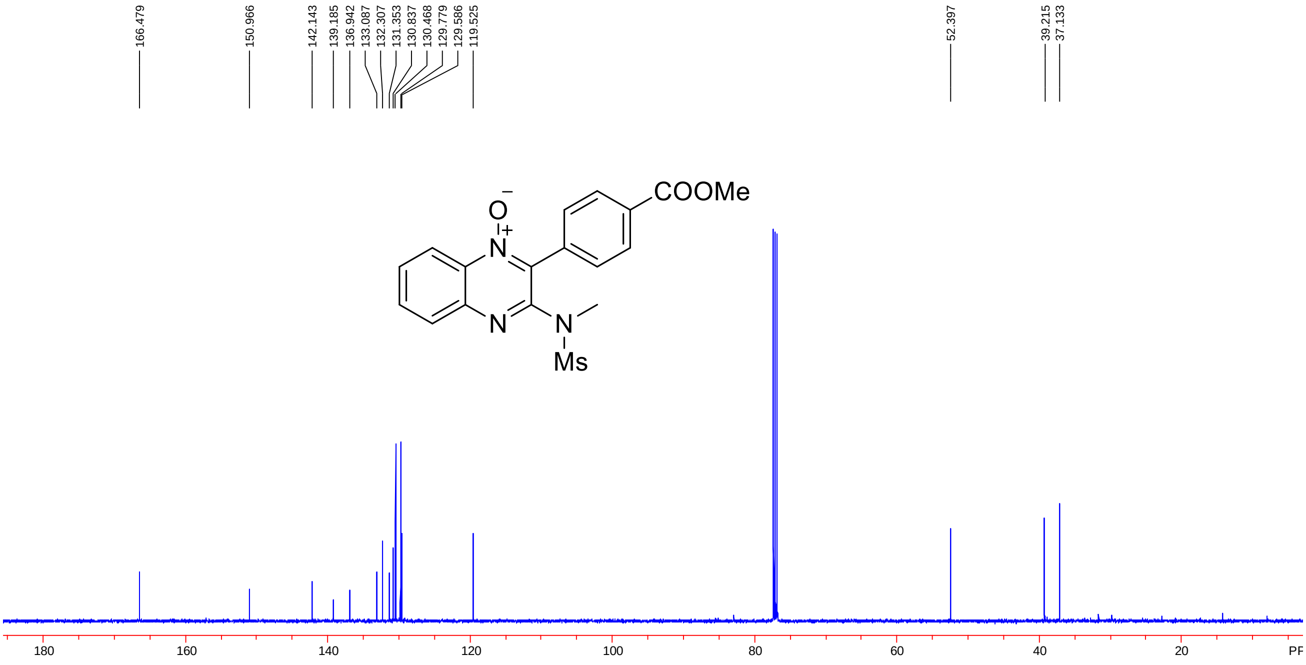

Supplementary Figure 109. ^{13}C NMR of compound **4t**

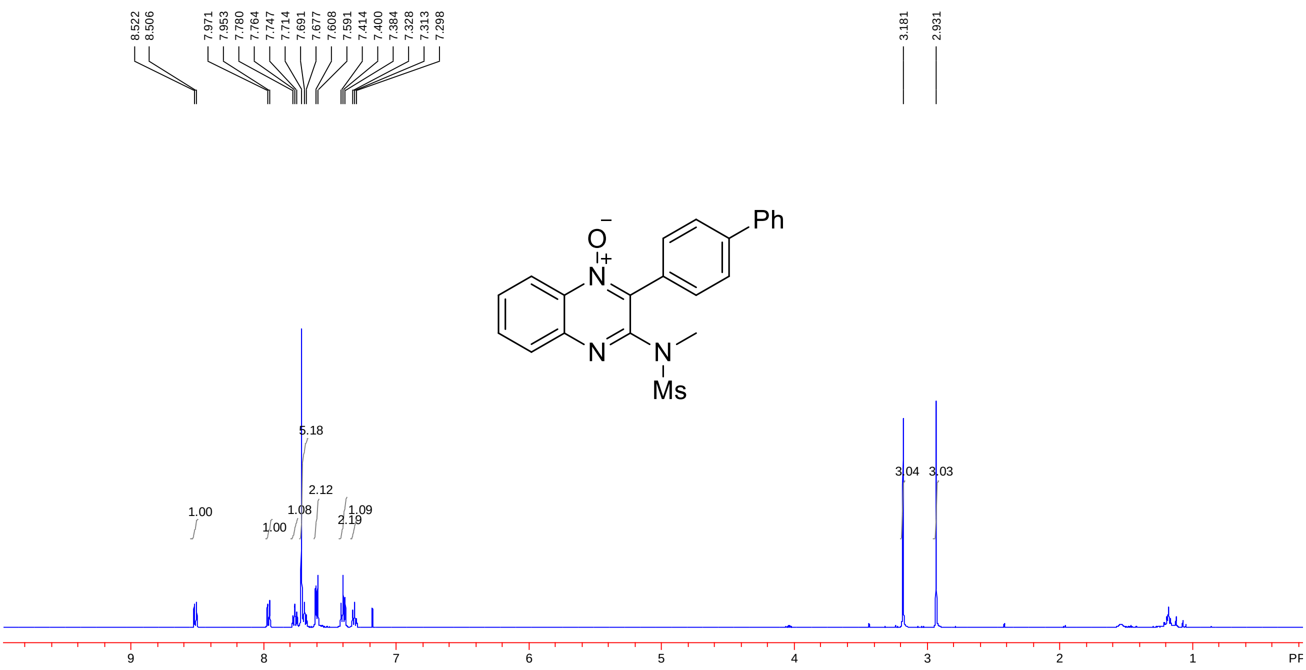

Supplementary Figure 110. ^{1}H NMR of compound **4u**

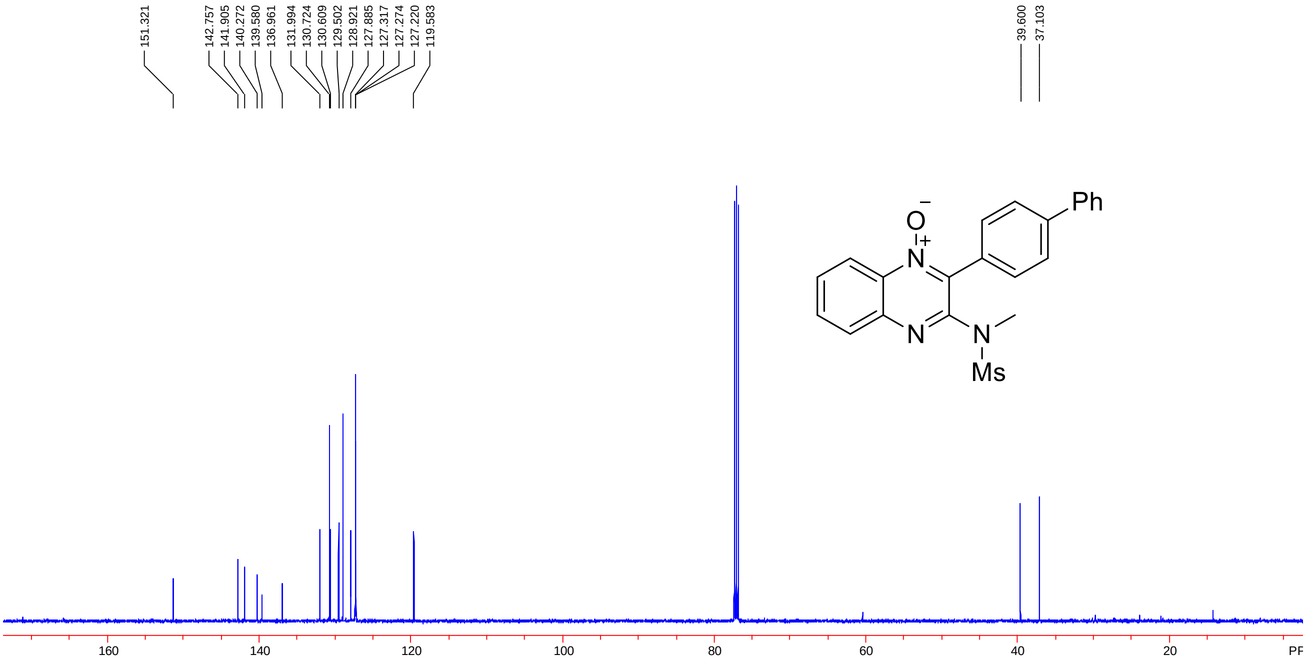

Supplementary Figure 111. ^{13}C NMR of compound **4u**

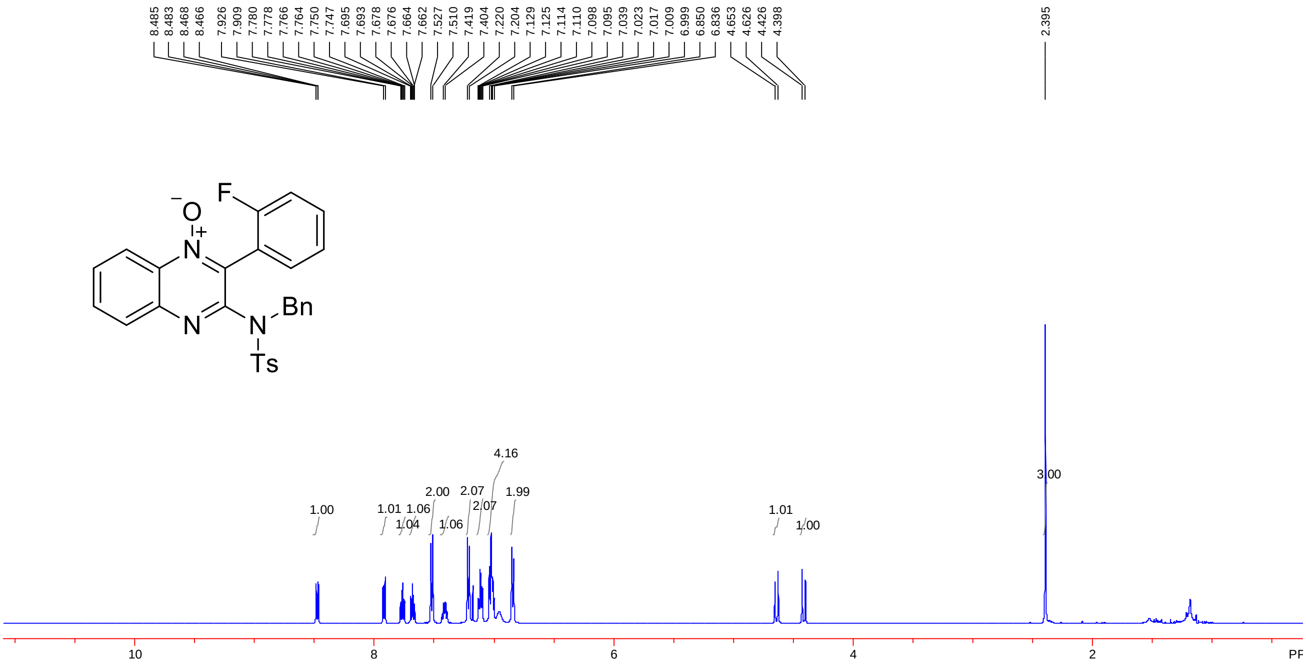

Supplementary Figure 112. ^{1}H NMR of compound **4v**

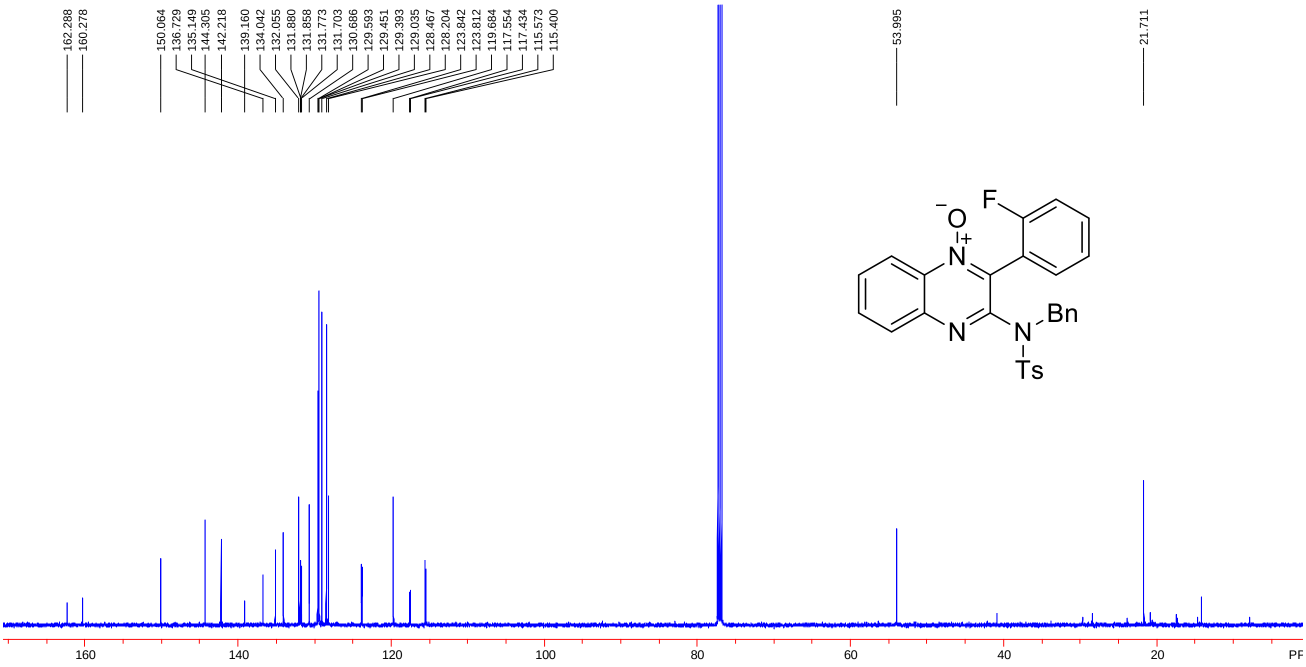

Supplementary Figure 113. ^{13}C NMR of compound **4v**

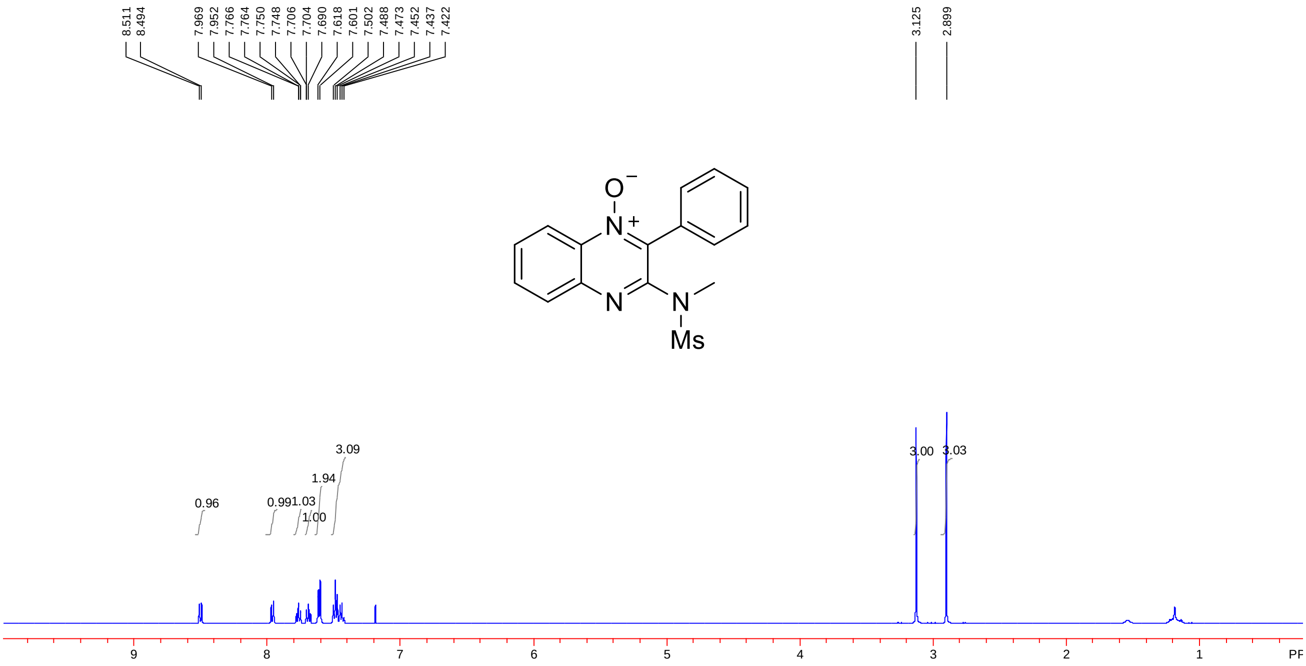

Supplementary Figure 114. ^{1}H NMR of compound **4w**

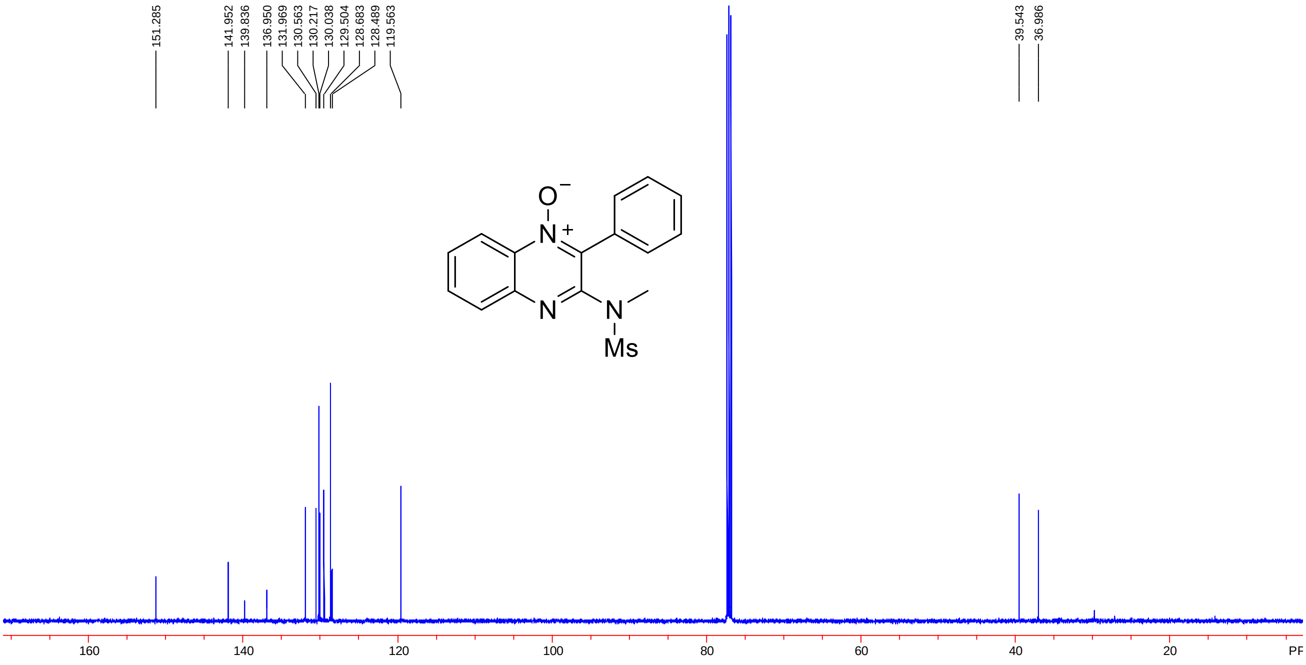

Supplementary Figure 115. ^{13}C NMR of compound **4w**

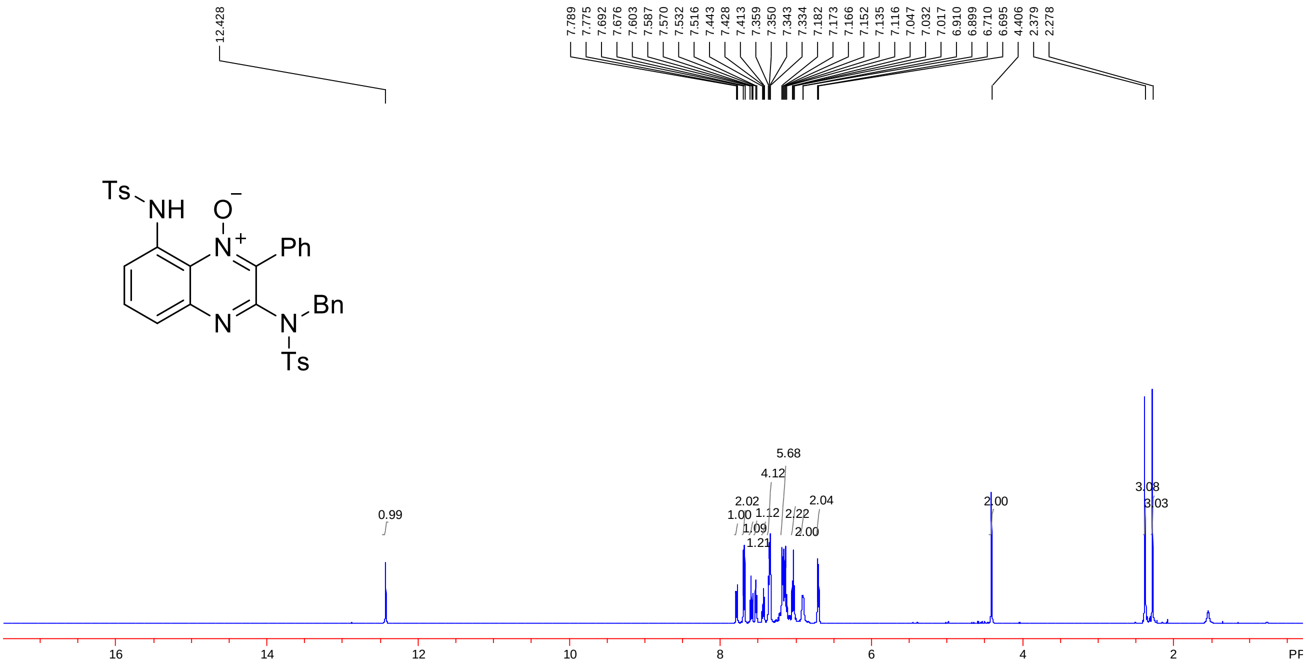

Supplementary Figure 116. ^{1}H NMR of compound **4i-1**

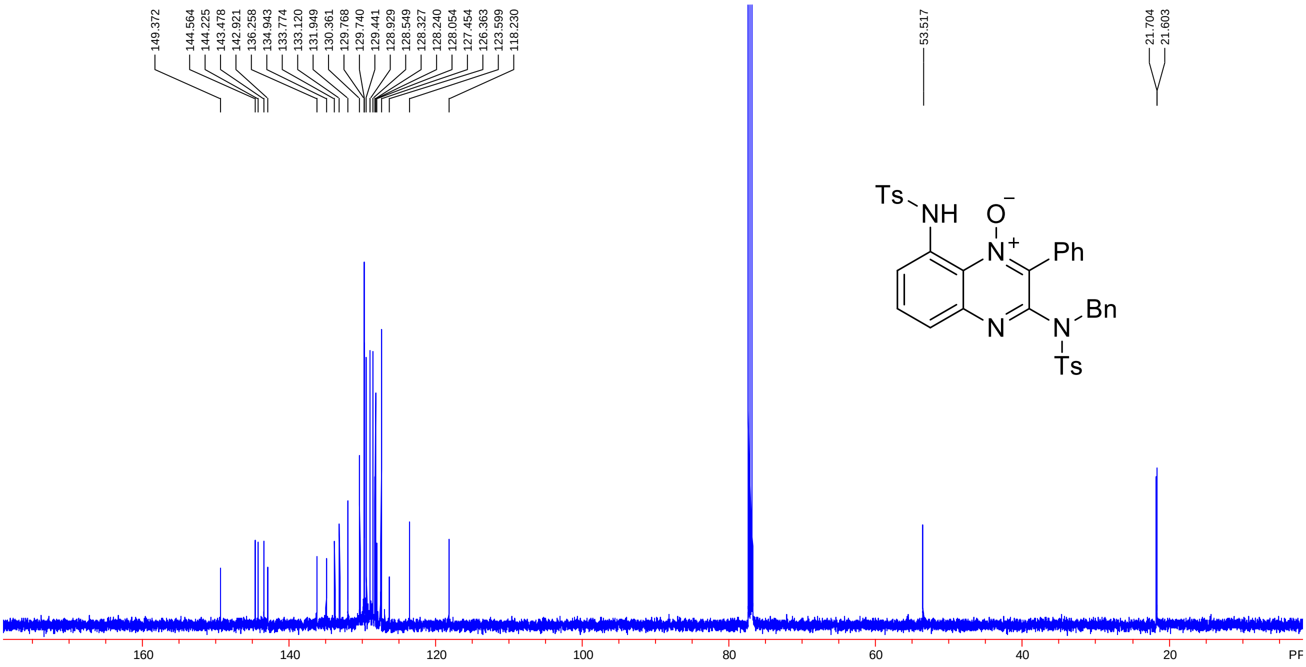

Supplementary Figure 117. ^{13}C NMR of compound **4i-1**

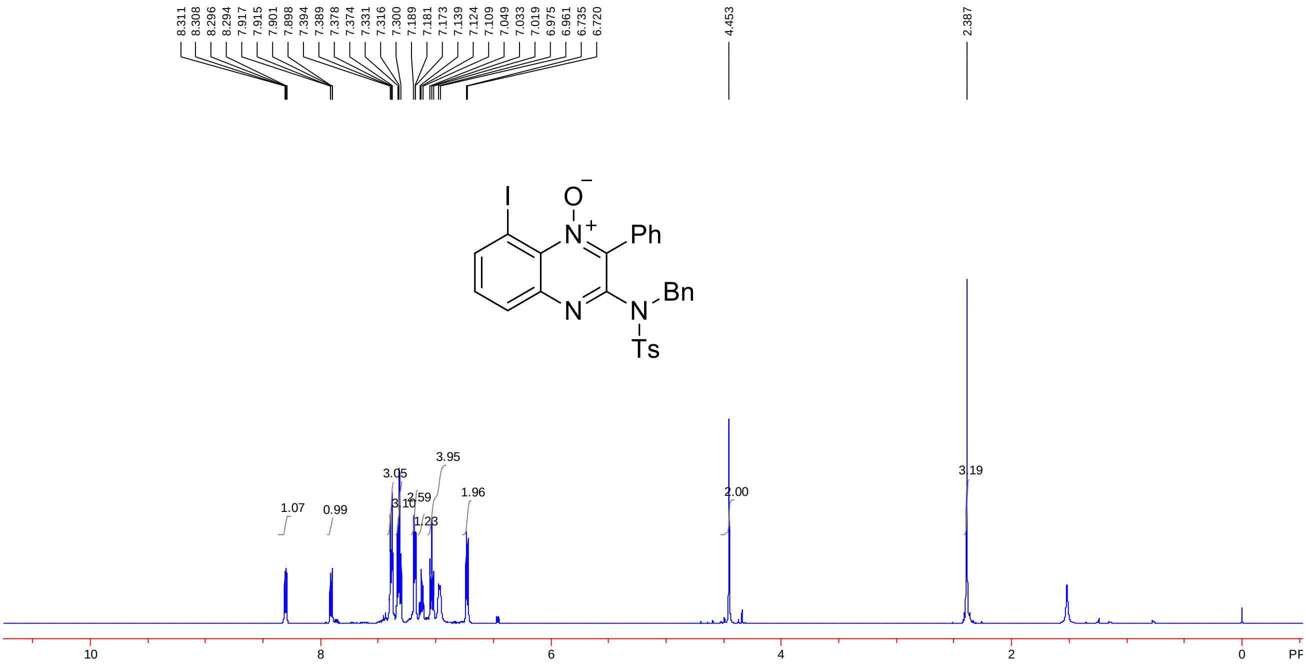

Supplementary Figure 118. ^{1}H NMR of compound **4i-2**

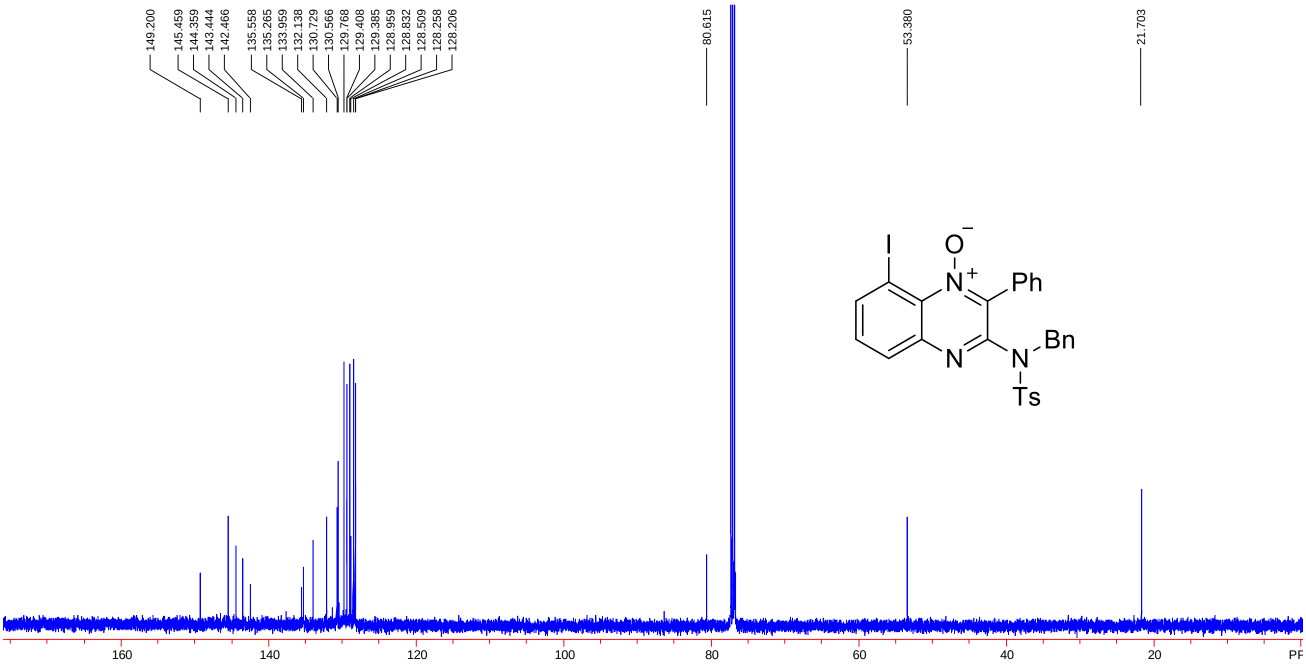

Supplementary Figure 119. ^{13}C NMR of compound **4i-2**

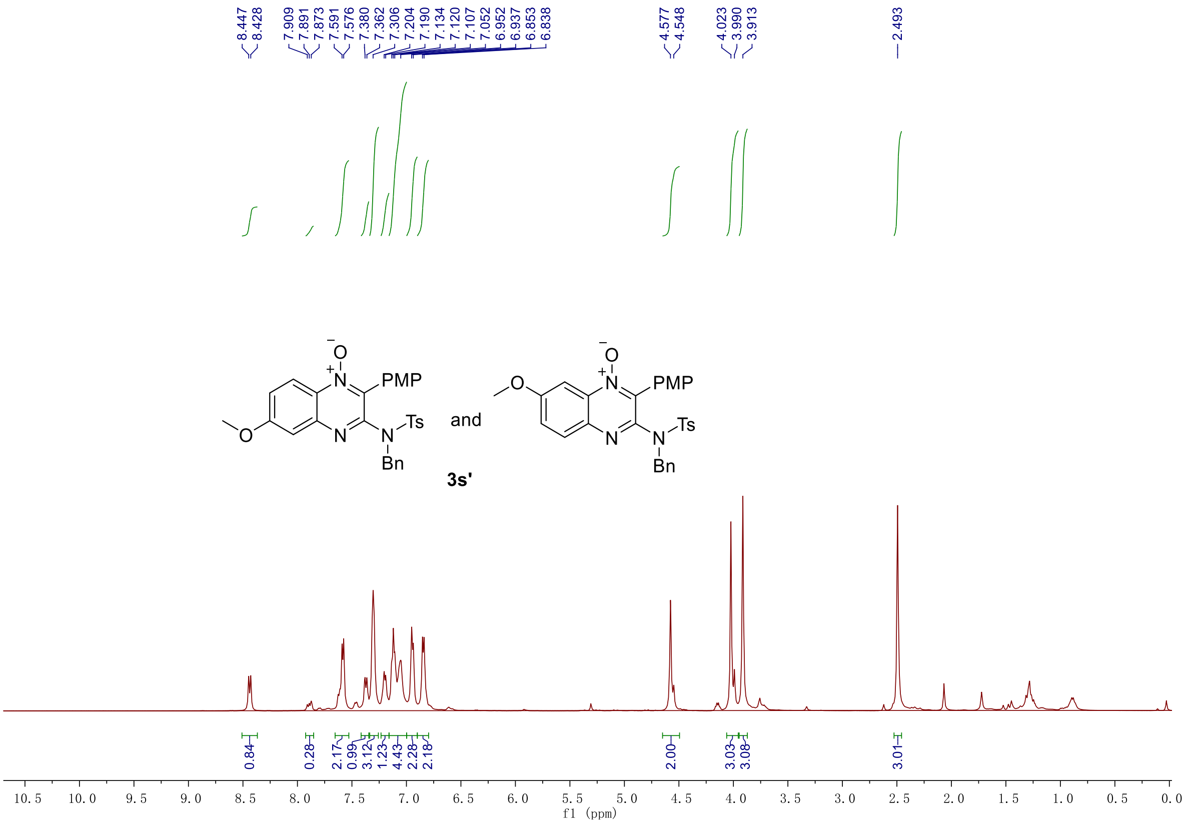

Supplementary Figure 120. ^{1}H NMR of compound **3s'**

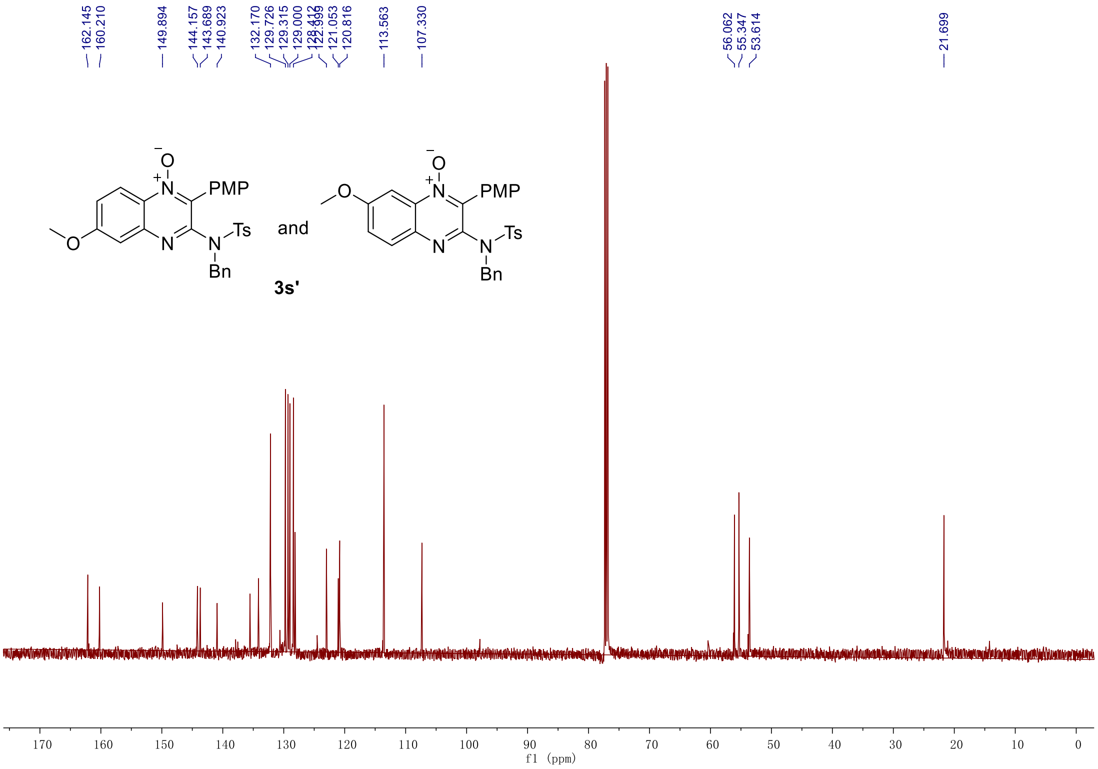

Supplementary Figure 121. ^{13}C NMR of compound **3s'**

2.5 X-Ray Crystal Structure Analyses

The crystallographic data (CCDC 1954158) can be obtained free of charge from The Cambridge Crystallographic Data Centre via www.ccdc.cam.ac.uk/data_request/cif.

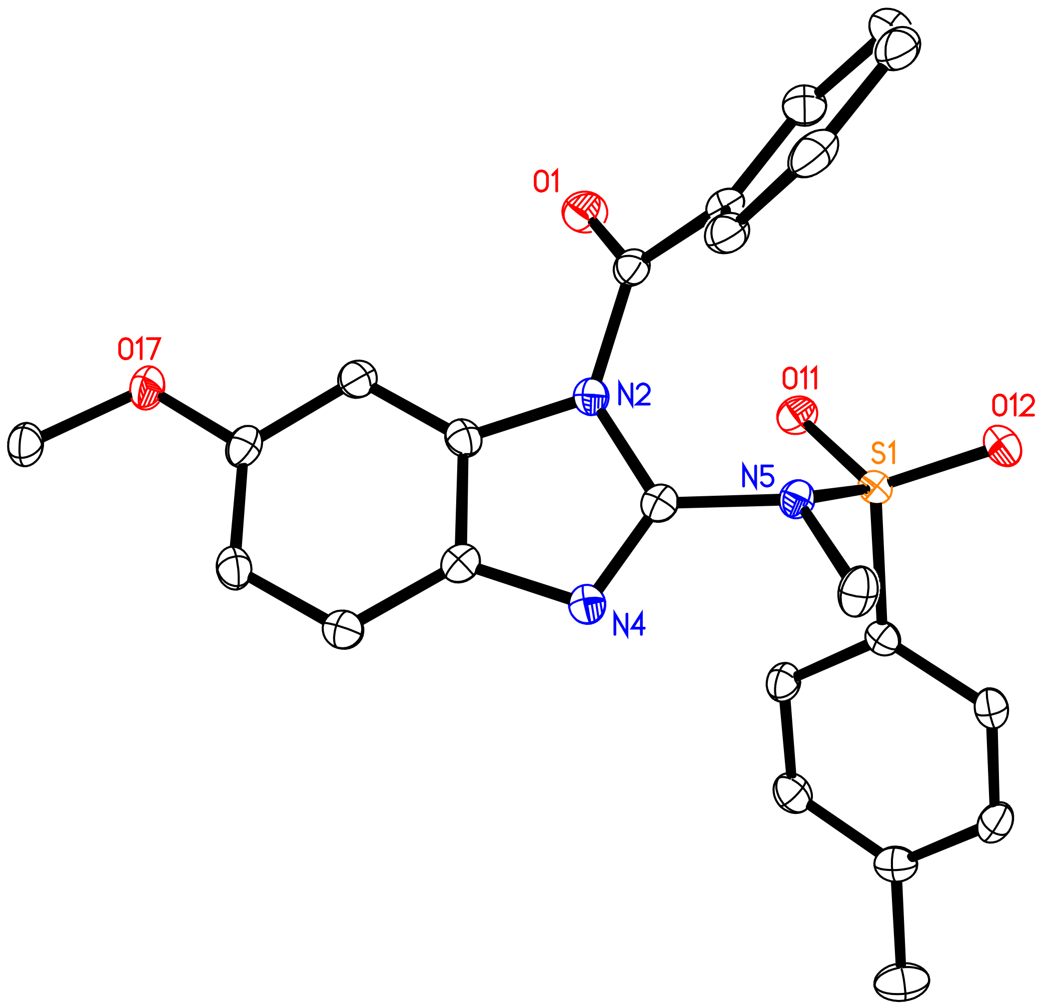

Supplementary Figure 122. X-Ray Crystal Structure of **3i**

The crystallographic data (CCDC 1570451) can be obtained free of charge from The Cambridge Crystallographic Data Centre via www.ccdc.cam.ac.uk/data_request/cif.

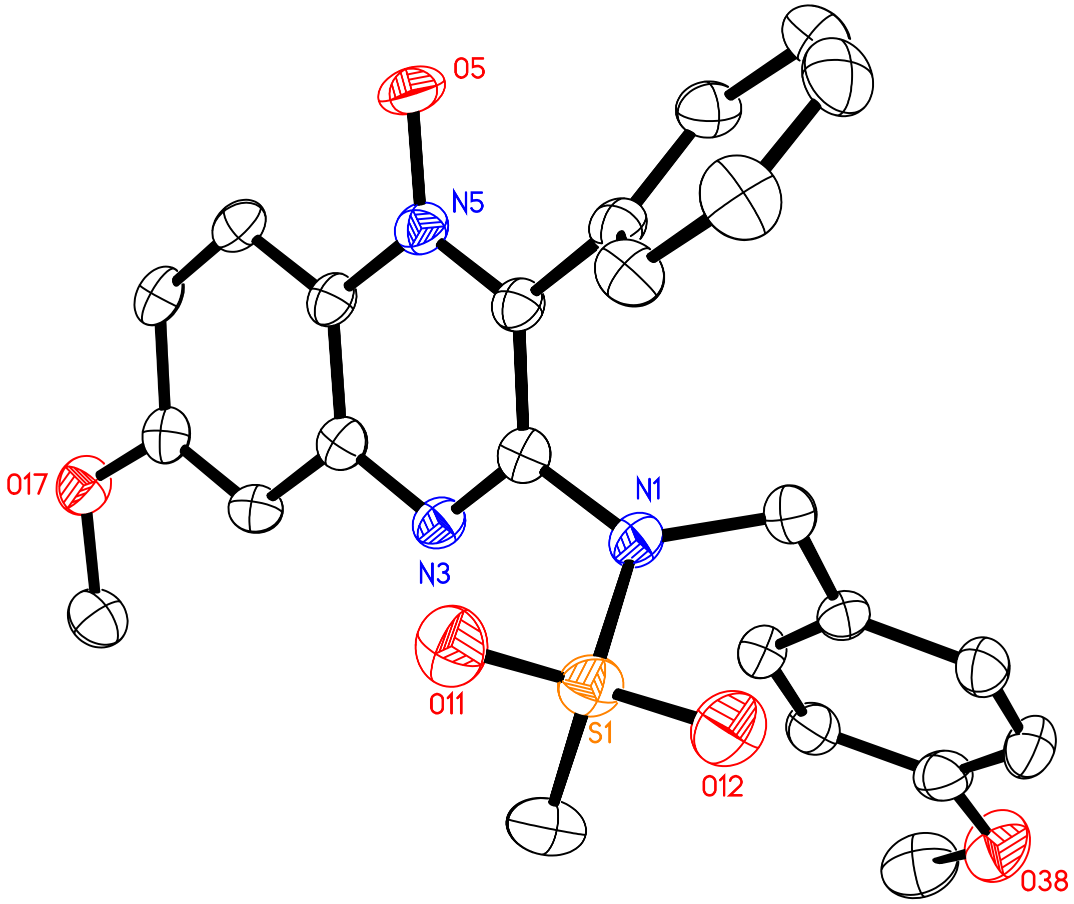

Supplementary Figure 123. X-Ray Crystal Structure of **4b**

3. Supplementary References

(1) Jouvin, K.; Coste, A.; Bayle, A.; Legrand, F.; Karthikeyan, G.; Tadiparthi, K.; Evano, G. Copper-Mediated Selective Cross-Coupling of 1,1-Dibromo-1-alkenes and Heteronucleophiles: Development of General Routes to Heterosubstituted Alkynes and Alkenes. *Organometallics* **2012**, *31*, 7933-7947.

(2) Kim, Y.; Dateer, R. B.; Chang, S. Borane-Catalyzed Selective Hydrosilylation of Internal Ynamides Leading to β-Silyl (Z)-Enamides. *Org. Lett.* **2017**, *19*, 190-193.

(3) Nobuo, S.; Hiroaki, S.; Shinichi, M.; Tohru, T. Synthesis of Quinoxaline 1,4-Dioxides from 5,6-Diethylbenzofuroxan on Silica Gel. *Heterocycles* **2005**, *65,* 1589-1600.

(4) Deghati, P. Y. F.; Borghini, A.; van den Nieuwendijk, A. M. C. H; Dissen-de Groote, M. Inhibition of Nucleoside Transport By New Analogues of Nitrobenzylthioinosine. *Bioorg. Med. Chem.* **2003** *11*, 899-908.

(5) a) Sheng, J.; He, R.; Xue, J.; Wu, C.; Qiao, J.; Chen, C. Cu-Catalyzed π-Core Evolution of Benzoxadiazoles with Diaryliodonium Salts for Regioselective Synthesis of Phenazine Scaffolds. *Org. Lett.* **2018**, *20*, 4458-4461; b) Dyall, L. Pyrolysis of Aryl Azides. VII. Interpretation of Hammett Correlations of Rates of Pyrolysis of Substituted 2-Nitroazidobenzenes. *Aust. J. Chem.*, **1986**, *39*, 89-101.

(6) Tricas, H.; Diebolt, O.; van Leeuwen, P. W. N. M. Bulky Monophosphite Ligands for Ethene Hydroformylation. *J. Catal*. **2013**, *298*, 198-205.

(7) Warżajtis, B.; Glišić, B. Đ.; Radulović, N. S.; Rychlewska, U.; Djuran, M. I. Gold(III) Complexes with Monodentate Coordinated Diazines: An Evidence for Strong Electron-Withdrawing Effect of Au(III) Ion *Polyhedron* **2014**, *79*, 221-228.

(8) a) Lee, T. C.; Yang, W. T.; Parr, R. G. Density-functional crystal orbital study on the structures and energetics of polyacetylene isomers. *Phys. Rev. B* **1988**, *37*, 785-789;

b) Miehlich, B.; Savin, A.; Stoll, H.; Preuss, H. Results obtained with the correlation energy density functionals ofBecke and Lee, Yang and Parr. *Chem. Phys. Lett.* **1989**, *157*, 200-206;

c) Stephens, P. J.; Devlin, F. J.; Chabalowski, C. F.; Frisch, M. J. Ab initio calculation of vibrational absorption and circular dichroism spectra using density functional force fields. *J. Phys. Chem.* **1994**, *98*, 11623-11627.

(9) Grimme, S.; Ehrlich, S.; Goerigk, L. Effect of the damping function in dispersion corrected density functional theory. *J. Comput. Chem.* **2011**, *32*, 1456-1465.

(10) Gaussian 16, Revision A.03, Frisch, M. J.; Trucks, G. W.; Schlegel, H. B.; Scuseria, G. E.; Robb, M. A.; Cheeseman, J. R.; Scalmani, G.; Barone, V.; Petersson, G. A.; Nakatsuji, H.; Li, X.; Caricato, M.; Marenich, A. V.; Bloino, J.; Janesko, B. G.; Gomperts, R.; Mennucci, B.; Hratchian, H. P.; Ortiz, J. V.; Izmaylov, A. F.; Sonnenberg, J. L.; Williams-Young, D.; Ding, F.; Lipparini, F.; Egidi, F.; Goings, J.; Peng, B.; Petrone, A.; Henderson, T.; Ranasinghe, D.; Zakrzewski, V. G.; Gao, J.; Rega, N.; Zheng, G.; Liang, W.; Hada, M.; Ehara, M.; Toyota, K.; Fukuda, R.; Hasegawa, J.; Ishida, M.; Nakajima, T.; Honda, Y.; Kitao, O.; Nakai, H.; Vreven, T.; Throssell, K.; Montgomery, J. A., Jr.; Peralta, J. E.; Ogliaro, F.; Bearpark, M. J.; Heyd, J. J.; Brothers, E. N.; Kudin, K. N.; Staroverov, V. N.; Keith, T. A.; Kobayashi, R.; Normand, J.; Raghavachari, K.; Rendell, A. P.; Burant, J. C.; Iyengar, S. S.; Tomasi, J.; Cossi, M.; Millam, J. M.; Klene, M.; Adamo, C.; Cammi, R.; Ochterski, J. W.; Martin, R. L.; Morokuma, K.; Farkas, O.; Foresman, J. B.; Fox, D. J. Gaussian, Inc., Wallingford CT, 2016.

(11) a) Roy, L. E.; Hay, P. J.; Martin, R. L. Revised basis sets for the LANL effective core potentials. *J. Chem. Theory Comput.* **2008**, *4*, 1029-1031;

b) Andrae, D.; Haeussermann, U.; Dolg, M.; Stoll, H.; Preuss, H. Energy-adjusted ab initio pseudopotentials for the second and third row transition elements. *Theor. Chem. Acc.* **1990**, *77*, 123-141.

(12) a) Hehre, W. J.; Ditchfield, R.; Pople, J. A. Self—Consistent Molecular Orbital Methods. XII. Further Extensions of Gaussian—Type Basis Sets for Use in Molecular Orbital Studies of Organic Molecules. *J. Chem. Phys.* **1972**, *56*, 2257-2261;

b) Francl, M. M.; Pietro, W. J.; Hehre, W. J.; Binkley, J. S.; Gordon, M. S.; DeFrees, D. J.; Pople, J. A. Self-consistent molecular orbital methods. XXIII. A polarizationtype basis set for secondrow elements. *J. Chem. Phys.* **1982**, *77*, 3654-3665.

(13) Ribeiro, R. F.; Marenich, A. V.; Cramer, C. J.; Truhlar, D. G. Use of solution-phase vibrational frequencies in continuum models for the free energy of solvation. *The Journal of Physical Chemistry B* **2011**, *115*, 14556-14562.

(14) a) Zhao, Y.; Truhlar, D. G. Density functionals with broad applicability in chemistry. *Acc. Chem. Res.* **2008**, *41*, 157-167;

b) Zhao, Y.; Truhlar, D. G. Benchmark energetic data in a model system for Grubbs II metathesis catalysis and their use for the development, assessment, and validation of electronic structure methods. *J. Chem. Theory Comput.* **2009**, *5*, 324-333.

(15) a) Wachters, A. J. H. Gaussian basis set for molecular wavefunctions containing third‒row atoms. *J. Chem. Phys.* **1970**, *52*, 1033-1036;

b) Francl, M. M.; Pietro, W. J.; Hehre, W. J.; Binkley, J. S.; Gordon, M. S.; DeFrees, D. J.; Pople, J. A. Self‒consistent molecular orbital methods. XXIII. A polarization‒type basis set for second‒row elements. *J. Chem. Phys.* **1982**, *77*, 3654-3665.

(16) Marenich, A. V.; Cramer, C. J.; Truhlar, D. G. Universal solvation model based on solute electron density and on a continuum model of the solvent defined by the bulk dielectric constant and atomic surface tensions. *J. Phys. Chem. B* **2009**, *113*, 6378-6396.

(17) Lu, T.; Chen, F. Multiwfn: a multifunctional wavefunction analyzer. *J. Comput. Chem.* **2012**, *33*, 580-592.
