# Supplementary material for: Dichotomy of platinum(II) and gold(III) carbene intermediates switching from N- to O-selectivity
Source: Nat Commun. 2022 Mar 30;13:1672. doi: 10.1038/s41467-022-29326-0 (PMC8967914; doi:10.1038/s41467-022-29326-0)
Supplement: Supplementary file 2 — Description of Additional Supplementary Files [file 41467_2022_29326_MOESM2_ESM.pdf]

Description of Additional Supplementary files

File name: Supplementary Data 1

Description: Energies and Cartesian coordinates
